# Chemodivergent transformations of amides using *gem*-diborylalkanes as pro-nucleophiles

**DOI:** 10.1038/s41467-020-16948-5

**Published:** 2020-06-19

**Authors:** Wei Sun, Lu Wang, Yue Hu, Xudong Wu, Chungu Xia, Chao Liu

**Affiliations:** 1 State Key Laboratory for Oxo Synthesis and Selective Oxidation, Suzhou Research Institute, Lanzhou Institute of Chemical Physics, Chinese Academy of Sciences, Lanzhou, 730000 PR China; 2 University of Chinese Academy of Sciences, Beijing, 100049 PR China; 3 Department of Chemistry and Chemical Engineering, Yibin University, Yibin, 644007 PR China; 4 Key Laboratory of Organosilicon Chemistry and Material Technology of Ministry of Education, Hangzhou Normal University, Hangzhou, 311121 PR China

**Keywords:** Organometallic chemistry, Synthetic chemistry methodology

## Abstract

Amides are versatile synthetic building blocks and their selective transformations into highly valuable functionalities are much desirable in the chemical world. However, the diverse structure and generally high stability of amides make their selective transformations challenging. Here we disclose a chemodivergent transformation of primary, secondary and tertiary amides by using 1,1-diborylalkanes as pro-nucleophiles. In general, selective B-O elimination occurs for primary, secondary amides and tertiary lactams to generate enamine intermediate, while tertiary amides undergo B-N elimination to generate enolate intermediate. Various in situ electrophilic trapping of those intermediates allows the chemoselective synthesis of α-functionalized ketones, β-aminoketones, enamides, β-ketoamides, γ-aminoketones, and cyclic amines from primary, secondary, tertiary amides and lactams. The key for these transformations is the enolization effect after the addition of α-boryl carbanion to amides.

## Abstract (web summary)

Amides are versatile synthetic building blocks, however the general stability of the amide bond makes its selective transformation challenging. Here, the authors report a chemodivergent transformation of primary, secondary and tertiary amides by using 1,1-diborylalkanes as pro-nucleophiles.

## Introduction

Amides are one of the fundamental structural units in tremedous natural products^1^, pharmaceuticals^2^, and agrochemicals^3,4^. Both the synthesis and transformation of amides are important for the modification of amides and the utilization of organic nitrogen resources, which have attracted tremedous attention in synthetic community. However, the activation of amides is challenging, majorly devoted to the resonance stability of N−C(O) bond (15–20 kcal/mol)^3^. Therefore, high activation energy is generally required for the transformation of amides.

**Fig. 1:**
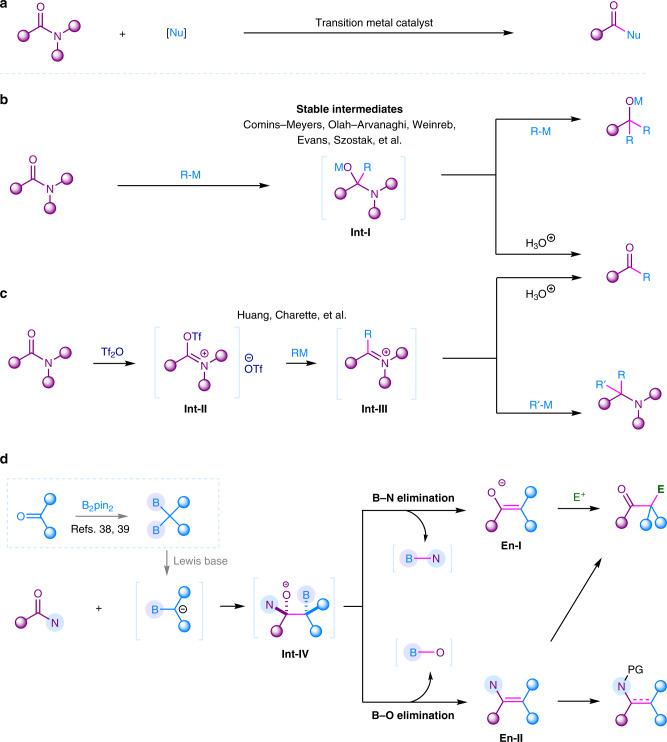
Strategies for the direct transformations of simple amides. **a** Transition metal catalyzed C–N activation/functionalization of amides. **b** Direct addition of [R–M] reagents to tertiary amides. **c** Transformation of amides via electrophilic activation using Tf_2_O. **d** This work: chemodivergent transformations of amides using 1,1-diborylalkanes as pro-nucleophiles.

Although a few transition metal catalyzed C–N activation of some activated amides have been achieved in cross-couplings (Fig. 1a)^5–7^, the major transformation of amides was initiated by the addition of highly active organometallic reagents, such as organolithiums and Grignard reagents onto their carbonyl groups to generate a tetrahedral intermediate **Int-I** (Fig. 1b). The transformation of this **Int-I** intermediate is vital for the following chemoselective synthesis. Intermediate **Int-I** is generally easy to eliminate amino group to produce more reactive ketone, in which the addition of the organometallic reagent to ketone would in situ occur to give overaddition product tertiary alcohols, although the elimination of oxyl group might also occur to finally generate tertiary amines in some special cases^8,9^. To chemoselectively obtain ketones, efforts have been devoted to stablize the **Int-I** intermediate to overcome the overaddition obstacle^8,10–17^. For example, Evans and Szostak attempted to form a stable tetrahedral intermediates upon addition of organometallics by tuning the *N*-substituent groups^10,11,18^; Weinreb developped a variety of *N*-methoxy-*N*-methyl amides which formed stable five-membered-ring metal chelated intermediate after the addition of organometallic reagents, in which acidic quenching provided a diverse synthesis of ketones (Weinreb ketone synthesis)^12–14^. Obviously, those strategies required structurally special tertiary amides to achieve chemoselective ketone synthesis from amides. Another powerful activation approach is the pre-*O*-acylation of amides using Tf_2_O (trifluoromethanesulfonic anhydride) to generate highly electrophilic intermediate **Int-II**^8,19–21^. The Huang group has done a significant contribution in chemodivergent transformations of amides using this activation method^22–28^. The Charette group has also used this activation method to convert a series of amides to ketones^29^. In this mode, reactive organometallic reagents R–M were required to react with **Int-II** to form intermediate **Int-III**, which gave ketones by acidic work-up or produced amines by another nucleophilic attack (Fig. 1c). This strategy was limited to secondary and tertiary amides. Therefore, the development of strategies for chemodivergent transformation of simple amides, including primiary amides, secondary amides, and tertiary amides, remains undeveloped.

1,1-Diborylalkanes as a special type of *gem*-dimetallic compounds have drawn much attention in organic synthesis in recent years, and many efforts on the synthesis of this type of compounds make them easily accessible^30–37^. Generally, 1,1-diborylalkanes could provide α-boryl carbanion synthons via deprotonation or Lewis base coordination. The addition of α-boryl carbanion to amides may give tetrahedral intermediate **Int-IV**. Differently, this intermediate bears a β-boryl group, which may result in chemodivergent B–O or B–N eliminations to generate enamine **En-I** or enolate **En-II**. This enolization effect will provide advantages in blocking second nucleophilic attack. Moreover, enamine and enolate are newly generated nucleophiles, and further electrophilic functionalization will provide more functionalized ketones or enamine derivatives from widely accesible amides.

Herein, we report a chemodivergent transformation of simple amides including primary, secondary, and tertiary amides using 1,1-diborylalkanes to access various functionalized ketones, enamides, α-alkylated cyclic amines, and β-ketoamides (Fig. 1d).

## Results and discussion

### Initial study

The 1,1-diborylalkanes could be facilely prepared from the direct borylation of aldehydes, ketones, or esters by using our recently developed methods^38,39^. α-Boryl carbanions from 1,1-diborylalkanes have previsouly been applied to add to carboxylic esters^40–42^. Recently, our group successfully achieved the transformation of carboxylic acids by using α-monoboryl carbanion^43,44^. Later, Xu and Zhao reported the transformation of special tertiary amide DMF (used as solvent) through a similar strategy to generate aldehydes^45,46^, in which a B–N elimination was proposed. Based on our previous study on carboxylic acids, we envisioned that a large number of more challenging primary, secondary, and tertiary amides might be chemodivergently transformed by using 1,1-diborylalkanes, because of that those diboron compounds may play dual roles: (1) the C–B moiety promotes the enolization process after the first nucleophilic addition; (2) the Lewis acidity of boryl group may promote the activation of amides by the coordination of oxygen or nitrogen atom with boron-center. Importantly, as shown in Fig. 1d, there are two possible eliminations for the generated intermediate **Int-IV**, making the chemistry more diverse.

**Fig. 2:**
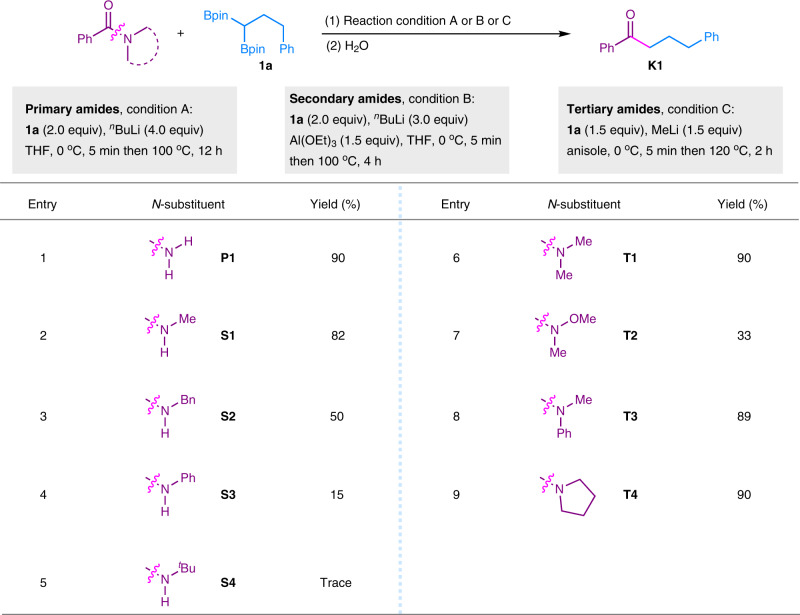
Reactivity study of different amides. Condition A (primary amides): **1a** (0.50 mmol), **P** (0.25 mmol), ^*n*^BuLi (1.0 mmol), THF (2.0 mL), 0 °C, 5 min then 100 °C for 12 h. Condition B (secondary aides): **1a** (0.50 mmol), **S** (0.25 mmol), ^*n*^BuLi (0.75 mmol), Al(OEt)_3_ (0.375 mmol), THF (2.0 mL), 0 °C, 5 min then 100 °C for 4 h. Condition C (tertiary amides): **1a** (0.375 mmol), **T** (0.25 mmol), MeLi (0.375 mmol), anisole (2.0 mL), 0 °C, 5 min then 120 °C for 2 h. Yields were determined by GC analysis using naphthalene as an internal standard. THF: tetrahydrofuran.

Our investigations began with the reaction of 3-phenyl-1,1-diborylpropane (**1a**) and a variety of typical primary, secondary, and tertiary amides as the model substrates (Fig. 2). We have shown that MeLi was an effective base for the activation of 1,1-diborylalkanes and quenching the acidci proton of carboxylic acids^43^. Then, MeLi was first applied as the base in the transformation of primary amide (**P1**, Fig. 2). However, it was less efficient and ^*n*^BuLi was showed to be the optimal base. Simple primary benzamide (**P1**) was selectively transformed to the desired ketone (**K1**) in 90% GC yield with H_2_O as the final quenching reagent (Fig. 2, entry 1). The reaction was conducted by adding ^*n*^BuLi (4.0 equiv) to the mixture of **1a** (2.0 equiv) and **P1** (1.0 equiv) at 0 °C for 5 min, followed by heating the mixture at 100 °C for 12 h (See detailed condition optimization in Supplementary Table 1). We assumed that two equivalents of *n*-BuLi was used to deprotonate the two protons of primary amide and the other two equivalents was used to coordinate to 1,1-diborylalkane to promote the generation of α-monoboryl carbanion. To our knowledge, only one successful report with limited examples has been demonstrated on the selective transformation of primary amides to generate ketones by using benzylic Grignard reagents over 80-years ago (1933)^47^. Encourged by this result, we then attempted to activate amides with different *N*-substitutents. Surprisingly, no desired product was observed when *N*-methylbenzamide (**S1**) was used as the substrate under the condition for **P1**. The transformation of secondary amides has been a long-time challenging topic and only the *O*-esterification or *O*-etherification strategy using Tf_2_O or TMSCl has been succeeded the activation of secondary amides. Lewis acids have been generally used to activate carbonyl groups. In this case, Al(OEt)_3_ was found to be effective for promoting the activation of secondary amide **S1**^48^, and 82% GC yield of **K1** was obtained under the optimized condition (Fig. 2, entry 2, see detailed condition optimizations in Supplementary Table 2). The reactivity of secondary amides varied with different *N*-substituents (Fig. 2, **S2**, **S3** and **S4**). *N*-Bn (**S2**) gave moderate yield of **K1**. *N*-Ph (**S3**) and *N*-^*t*^Bu (**S4**) gave low conversions of amides, which might be attributed to the steric hindrance. In the case of tertiary amide, no acid proton presented in the substrates, and equal amount of MeLi was found to be sufficient enough for the activation of 1,1-diborylalkane. **K1** was obtained in 90% GC yield from substrate **T1** (-NMe_2_). Although a good yield could be obtained by using THF as the solvent, anisole provided a better result. The temperature could be lowered to 100 °C with a slightly decreased yield (See detailed condition optimization in Supplementary Table 3). Subsequently, substrates bearing different *N*-substituents were also evaluated. Weinreb amide (**T2**) reacted to give low yield of ketone **K1**. The reactions proceeded smoothly for tertiary amides bearing *N*-Me-*N*-Ph (**T3**) and pyrrolidin-1-yl (**T4**) groups, affording the desired **K1** in excellent yields.

### Mechanistic consideration

**Fig. 3:**
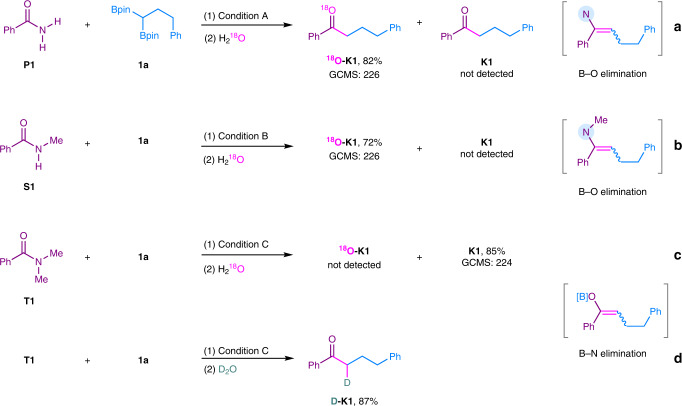
Isotopic labeling experiments. **a** ^18^O-labeling experiment of primary amide **P1**. **b** ^18^O-labeling experiment of secondary amide **S1**. **c** ^18^O-labeling experiment of tertiary amide **T1**. **d** Deuterium-labeling experiment of tertiary amide **T1**.

It has been demonstrated that **Int-IV** could undergo two possible eliminations (See above). To verify the eliminations, ^18^O-labeling experiments with primiary (**P1**), secondary (**S1**) and tertiary (**T1**) amides as the models were carried out by using H_2_^18^O as the quenching reagent (Fig. 3, eqs. a–c). Obviously, B–O elimination would give ^18^O-labeling product ^**18**^**O-K1**, and non-labeled ketone **K1** would be obtained if B–N elimination occurs. As a result, only the ^**18**^**O-K1** was observed in the cases of primary and secondary amides (Fig. 3, eqs. a–b). In the case of tertiary amide **T1**, only non-labeled **K1** was isolated (Fig. 3, eq. c). These results indicated that B–O elimination selectively ocurred in the reactions of primary and secondary amides, and B–N elimination selectively ocurred for tertiary amides. Furthermore, when D_2_O was used as the quenching reagent, the α-deuterated ketone **D-K1** was obtained in 87% yield (Fig. 3, eq. d). These results showed that an enolate species was generated in the case of tertiary amide.

### Scope of the methodology

**Fig. 4:**
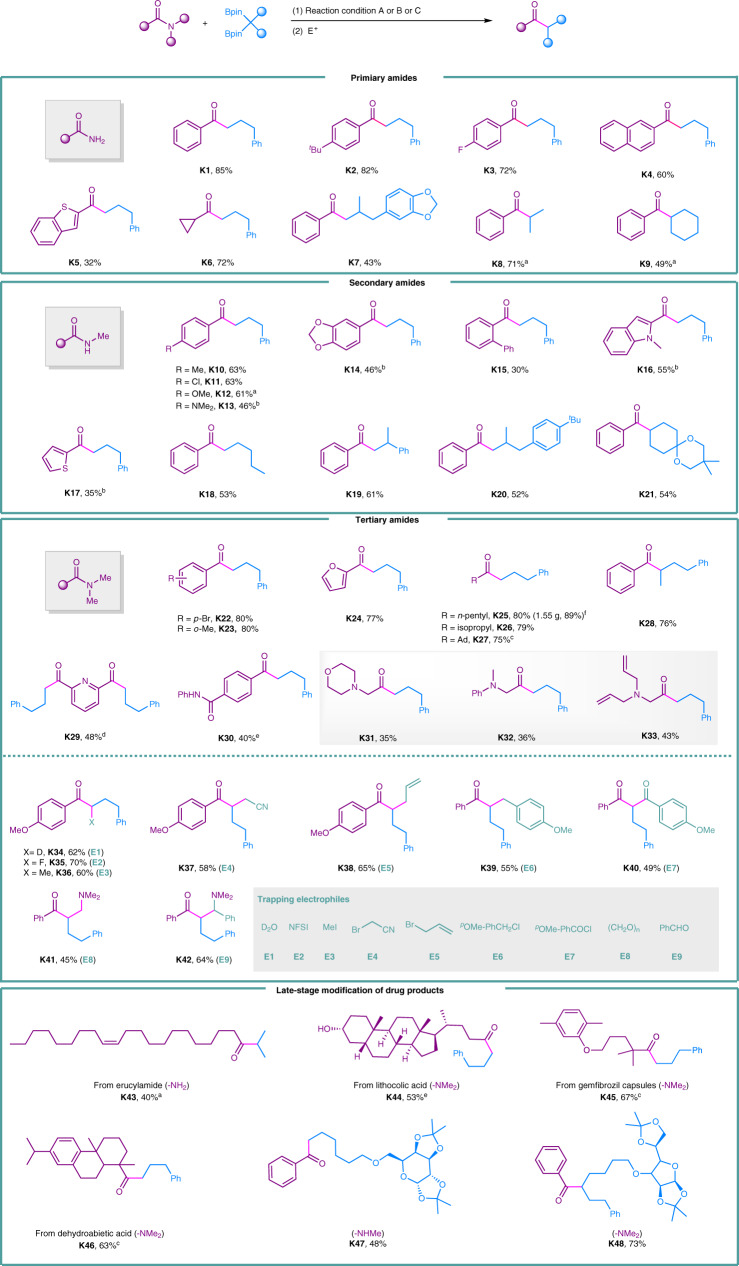
Exploration of substrate scope for the synthesis of ketones from amides. Reaction condition A for primary amide: **1** (0.50 mmol), primiary amide **P** (0.25 mmol), ^*n*^BuLi (1.0 mmol), THF (2.0 mL), 0 °C, 5 min then 100 °C for 12 h; Reaction condition B for secondary amide: **1** (0.50 mmol), secondary amide **S** (0.25 mmol), ^*n*^BuLi (0.75 mmol), Al(OEt)_3_ (0.375 mmol), THF (2.0 mL), 0 °C, 5 min then 100 °C for 4 h. Reaction condition C for tertiary amide: **1** (0.375 mmol), tertiary amide **T** (0.25 mmol), MeLi (0.375 mmol), anisole (2.0 mL), 0 °C, 5 min then 120 °C for 2 h. For detailed conditions of trapping electrophiles, see Supplementary Information. Yields based on isolated products. ^*a*^24 h. ^*b*^12 h. ^*c*^THF was used instead of anisole. ^*d*^**1** (0.75 mmol), MeLi (0.75 mmol). ^*e*^MeLi (0.625 mmol). ^*f*^ 8.0 mmol scale. Ad: adamantyl.

With the understanding of this amide transformation process, the substrate scope was then explored. Primary amides (–CONH_2_) were initially investigated to react with **1a** (Fig. 4, **K1**–**K9**). The benzamides bearing *p*-^*t*^Bu and *p*-F afforded their corresponding ketones **K2** and **K3** in good yields. The 2-naphthamide gave the corresponding product **K4** in 60% yield. Benzothienyl-derived heterocycle was also suitable for this transformation, albeit providing the desired product **K5** in a relatively low yield. Moreover, aliphatic amide bearing cyclopropyl was also successfully transformed to the desired product (**K6**) in high yield. With several different 1,1-diborylalkanes, the reactions also proceeded well with benzamide to afford the desired ketones (**K7**–**K9**). Subsequently, a series of secondary amides were assessed with **1a** as the standard 1,1-diborylalkane. Several substituted *N*-Me benzamides were subjected to give their desired products in moderate to good yields (**K10–K14**). The tolerance of *para*-Cl substituent would allow for further functionalization (**K11**). The *ortho*-Ph substrate showed relatively lower reactivity, indicating that steric hindrance might has negative effect for this transformation (**K15**). Heteroaromatic secondary amides also gave the desired products smoothly (**K16** and **K17**). Furthermore, the scope of 1,1-diborylalkanes was also explored (**K18**-**K21**) with **S1** as the coupling partner. Remarkably, non-terminal 1,1-diborylalkane was well tolerated in this transformation, affording the product in moderate yield (**K21**). In the cases of tertiary amides, the -NMe_2_ amides were next evaluated with **1a** as the model 1,1-diborylalkane. A variety of aromatic and aliphatic tertiary amides were well performed under the standard conditions for tertiary amides (**K22–K27**). Functional groups, such as *p*-Br and *o*-Me were all well compatible to release the corresponding ketones in good to excellent yields (**K22** and **K23**). Furyl-derived substrate was also subjected to this transformation, affording the corresponding ketone in good yield (**K24**). Several aliphatic tertiary amides (**K25**–**K27**) were also successfully converted into the desired ketones in good yields. In the case of **K25**, a gram-scale (8.0 mmol) reaction was conducted and 1.55 g (89%) of **K25** was isolated, demonstrating the possibility to scale up this reaction. The substrate with steric-hindered group (Ad-) was well tolerated (**K27**). Non-terminal 1,1-diborylalkanes reacted with **T1** to give the corresponding product **K28** in good yield. The diketone **K29** was generated from pyridyl diamide in 48% yield. Since 1,3-diacyl pyridines are suitable precursors for NNN-type diimine ligands, this method may provide an alternative approach for the diverse synthesis of these ligands. Secondary amide was well tolerated during the chemoselective transformation of tertiary amide in this process to deliver the corresponding **K30** in 40% yield, demonstrating that secondary amide was less reactive than that of tertiary amide. Peptides were generally connected by amide bond. The transformation of α-amino amide derivatives may provide a solution for peptides modification. Three α-amino amides were successfully converted to the corresponding α-aminoketones (**K31**–**K33**) by using **1a**. As mentioned above, boron enolates were generated from the reaction between 1,1-diborylalkanes and tertiary amides via B–N elimination. Various electrophiles (**E**) were applied to trap these boron enolates to achieve the synthesis of various functionalized ketones. The desired α-deuterated ketone (**K34)** was isolated in 62% yield by using D_2_O (**E1**) as the trapping electrophile. When NFSI (**E2**) was used, α-fluoro ketone (**K35)** was obtained in 70% yield. Subsequently, a series of alkyl halides, such as iodomethane (**E3**), bromoacetonitrile (**E4**), allyl bromide (**E5**), and benzylic halide (**E6**) were used, the corresponding products (**K36**–**K39**) were generated in moderate to good yields. When 4-methoxybenzoyl chloride (**E7**) was used, the desired unsymmetrical 1,3-diketone (**K40**) was obtained in 49% yield. Surprisingly, using paraformaldehyde (**E8**) and benzaldehyde (**E9**) as the trapping electrophiles, the released amine moieties were in situ captured by aldehyde to generate iminium intermediates which reacted with boron enolate to give β-aminoketones in moderate yields (**K41** and **K42**) without the observation of aldol products. These results indicated that the formation of iminium from aldehyde and released dimethylamino part was fast enough to overcome the direct addition of boron enolate to aldehyde^49^.

To further demonstrate the utility of this transformation, the late-stage modification of amides from natural products and drugs were carried out under standard reaction conditions. Erucylamide, as one kind of surface active agents, was transformed to the desired ketone with the well reservation of olefinic bond (**K43**). The derivatives of gemfibrozil capsules, dehydroabietic acid, and lithocolic acid were also applied in this transformation to afford the corresponding ketones in moderate yields (**K44**–**K46**). The success of lithoclic acid derivative highlighted the tolerance of free hydroxyl group. 1,1-Diborylalkanes bearing galactose functionality were well conducted with both secondary and tertiary amides to give their corresponding ketones in moderate to good yields (**K47** and **K48**).

**Fig. 5:**
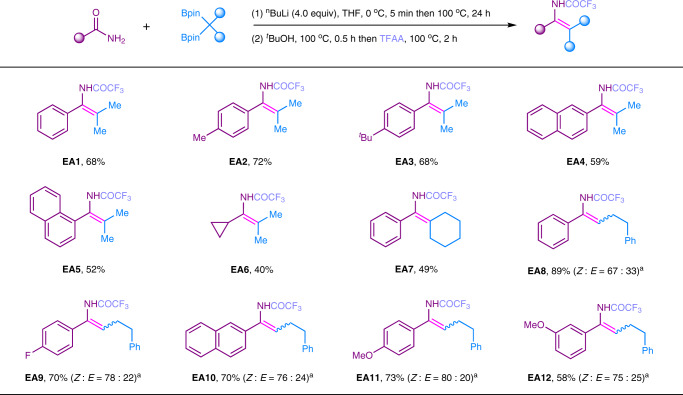
Synthesis of enamides via transformation of primary amides using 1,1-diborylalkanes. Reaction conditions: 1) **1** (0.50 mmol), primiary amide (0.25 mmol), ^*n*^BuLi (1.0 mmol), THF (2.0 mL), 0 °C, 5 min then 100 °C for 24 h. 2) ^*t*^BuOH (0.50 mL), 100 °C, 0.5 h then TFAA (1.0 mL), 100 °C, 2 h. Yields based on isolated products. The geometry and ratios of Z/E were determined by NMR analysis and shown in parenthesis. ^a^12 h. TFAA: trifluoroacetic anhydride.

With the insightful understanding of these transformation, the enamine intermediates were generated by B–O elimination process when primary and secondary amides were used (See above). To keep the nitrogen in the final products, we attempted to in situ capture those enamines to generate isolable enamides. Enamides are important compounds, which have been extensively used in the synthesis of chiral amines via enantioselective hydrogenation^50–52^. However, efficient synthesis of those motifs still remain challenging. In this case, after the completion of the reaction between primary amides and 1,1-diborylalkanes, the addition of tertiary butanol followed by TFAA successfully captured enamines to afford various enamides (Fig. 5). We assumed that tertiary butanol was functioned as an activator to release reactive enamines from boron-coordinated enamines. As a result, TFAA could efficiently capture those reactive enamies to give enamides. Substrates with different functional groups, such as *p*-Me, *p*-^*t*^Bu were converted to the corresponding enamides in good yields (**EA2, EA3**). 1-Naphthamide and 2-naphthamide afforded their enamides in moderate yields (**EA4**, **EA5**). Cyclopropyl amide was tolerated to give the product (**EA6**). Moreover, 1,1-diborylalkane from cyclohexanone was compatible and generated the desired enamide (**EA7**). Subsequently, *p*-F, *p*-OMe, *m*-OMe functionalities were all tolerated and generated the corresponding products in moderate to good yields (**EA8**–**EA12**). However, in the case of 1,1-diborylalkane **1a**, only moderate stereoselectivities were obtained. Unfortunately, secondary amides were failed to be captured to give enamides.

**Fig. 6:**
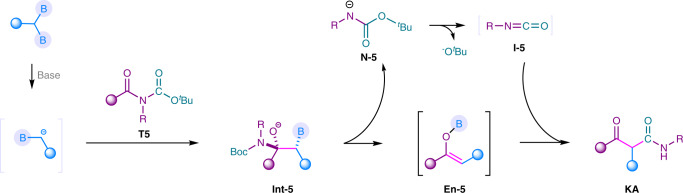
Mechanistic hypothesis. Proposed pathway for the transformation of *N*-Boc amides.

**Fig. 7:**
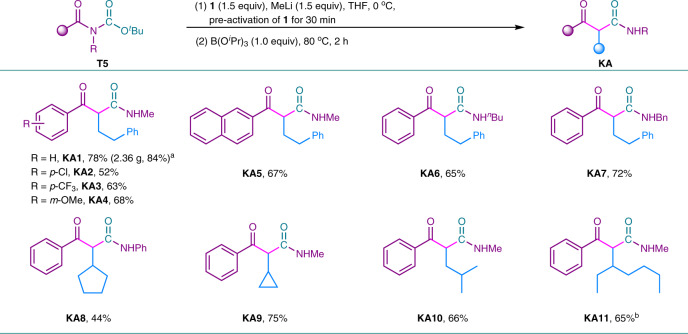
Synthesis of *β*-ketoamides via transformation of *N*-Boc amides using 1,1-diborylalkanes. Reaction conditions: **1** (0.45 mmol), MeLi (0.45 mmol), THF (3.0 mL), 0 °C, 30 min then *N*-Boc amides **T5** (0.30 mmol), B(O^*i*^Pr)_3_ (0.30 mmol), 80 °C for 2 h. ^*a*^10.0 mmol scale. Yields based on isolated products. ^*b*^The d.r. value was not determined.

As stated above, tertiary amides occured B–N elimination to generate boron enolate. If the released amino part could in situ generate a *N*-containing elelctrophile, an in situ reaction of this *N*-containing electrophile with boron enolate may occur. We noticed that the reaction of *N*-Boc tertiary amide **T5** with α-monoboryl carbanion might generate boron enolate **En-5** and release *N*-containing intermediate **N-5**. **N-5** might eliminate to generate isocyanate **I-5**, which could react with boron enolate to give β-ketoamides (Fig. 6)^53^. Those *N*-Boc and *N*-(Boc)_2_ tertiary amides are relative reactive and twisted^54,55^. When *N*-Boc **T5** was subjected to the reaction with 1,1-diborylalkanes, the corresponding β-ketoamides were obtained in good to high yields, in which B(O^*i*^Pr)_3_ was used as an effective additive (Fig. 7, See detailed reaction parameters in Supplementary Table 4). This reaction was also carried out in a 10.0 mmol scale to show the possibility of gram-scale synthesis. As a result, 2.36 g of **KA1** was obtained with 84% yield. Functional groups, such as Cl (**KA2**), CF_3_ (**KA3**) and OMe (**KA4**) were well tolerated. The naphthyl-derived *N*-Boc tertiary amide was also successfully converted to the desired product in 67% yield. Moreover, different *N*-substitutents, such as butyl, benzyl, and phenyl groups were well transformed to the corresponding products in moderate to good yields (**KA6**, **KA7**, **KA8**). We have also tested the *N*-(Boc)_2_ substrate, which only afforded trace amount of the desired β-ketoamide. Subsequently, different 1,1-diborylalkanes also afforded the desired β-ketoamides with satisfied yields (**KA8**-**KA11**). β-Ketoamides are useful skeloton in photographic compositions and organic synthesis. Moreover, they have also been used as photochemical precursors for isocyanates^56^. This approach provided an alternative for the synthesis of β-ketoamides.

**Fig. 8:**
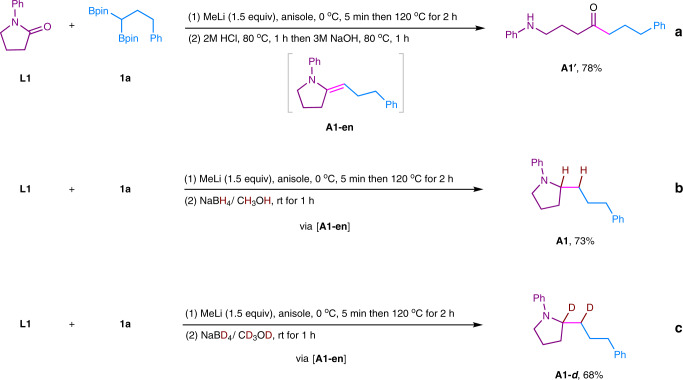
Investigation for the transformation of lactam. **a** Hydrolysis for the reaction of lactam **L1** with **1a**. **b** Reduction for the reaction of lactam **L1** with **1a** using NaBH_4_. **c** Reduction for the reaction of lactam **L1** with **1a** using NaBD_4_.

Next, according to the B–N elimination of tertiary amides, when tertiary lactam 1-phenylpyrrolidin-2-one was investigated, γ-aminoketone should be easily obtained as the product. However, to our surprise, the γ-aminoketone **A1′** was only obtained (78%) after the reaction mixture being treated by HCl followed by NaOH at 80 °C (Fig. 8, eq. a). We suspected that a B–O elimination between **1a** and **L1** might occur to give enamine **A1-en** as the intermediate. The **A1-en** was unstable and its hydrolysis afforded γ-aminoketone **A1′**. To prove this hypothesis, an appropriate reducant (NaBH_4_/MeOH) was added to reduce the double bond. As a result, saturated cyclic amine **A1** was obtained in 73% yield (Fig. 8, eq. b). When NaBD_4_/ CD_3_OD was used as the reductant, the desired fully D-labeled **A1-*d*** was obtained in 68% yield (Fig. 8, eq. c), demonstrating that the B–O elimination occurred for lactams, and enamine **A1-en** was generated as an unstable product. Overall, the deoxygenative alkylation of lactams was achieved by using 1,1-diborylalkanes to synthesis α-functionalized cyclic amines, which are potentially key units in numerous pharmaceutical drugs^57,58^.

**Fig. 9:**
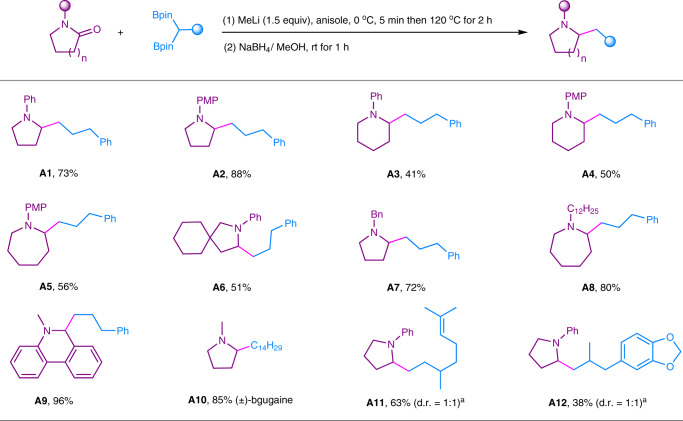
Deoxygenative alkylation of lactams using 1,1-diborylalkanes. Reaction conditions: 1) tertiary lactam (0.25 mmol), 1,1-diborylalkane (0.375 mmol), MeLi (0.375 mmol), anisole (2.0 mL), 0 °C, 5 min then 120 °C, 2 h; 2) NaBH_4_ (0.75 mmol), MeOH (1.0 mL), rt for 1 h. Yields based on isolated products. ^*a*^The d.r. value was determined by ^1^H NMR analysis. PMP: *p*-methoxyphenyl; Bn: benzyl.

We then turned our attention to investigate the scope of tertiary lactams, and the results were shown in Fig. 9. Five to seven-membered rings of *N*-aryl lactam were all compatible under current reaction condition, affording the desired α-alkylated cyclic amines in moderate to excellent yields (**A1**–**A5**). In addition, spiramide was also successfully transformed to the corresponding amine in 51% yield (**A6**). Different *N*-alkyl substituents were well tolerated (**A7**, **A8**). Phenanthridinone, which is widely found in natural products and bioactive molecules^1,59^, also furnished the desired product in excellent yield (**A9**). Two other 1,1-diborylalkanes were also tested and the desired cyclic amines **A10**–**A12** were well obtained. The alkaloids (±)-bgugaine (**A10**) was well synthesized from a common solvent *N*-methyl pyrrolidone in an excellent yield.

In summary, we have demonstrated a chemodivergent transformation of primary, secondary, tertiary amides and lactams by using 1,1-diborylalkanes as pro-nucleophiles. B–O elimination occurred for primary and secondary amides and tertiary lactams to generate enamine intermediate, and B–N elimination occurs for tertiary amides to generate enolate intermediate. The in situ hydrolysis of these intermediates achieved the synthesis of ketones from various amides. The in situ trapping of enolates with electrophiles generated α-functionalized ketones, in which β-aminoketones were well obtained by using aldehydes as the trapping electrophiles. The in situ trapping of those enamine intermediates by using TFAA allowed the synthesis of enamides from primary amides. When *N*-Boc tertiary amides were used, β-ketoamides were synthesized via the in situ generation of isocyanate as the trapping electrophiles. The deoxygenative alkylation of lactams was achieved by using 1,1-diborylalkanes to synthesis α-functionalized cyclic amines via in situ reduction of the enamine intermediates. Moreover, these methods have been applied to exhibit the late-stage functionalization of natural products, drug products.

## Methods

### Synthesis of ketones from primary amides

To a 25 mL resealable reaction tube of solvent flask equipped with a magnetic stirring bar, primary amide (0.25 mmol), 1,1-diborylalkane (0.50 mmol) were added and charged with N_2_ three times. Then, THF (2.0 mL) was added. The mixture was cooled down to 0 °C under nitrogen protection. Subsequently, ^*n*^BuLi (1.0 mmol, 2.5 mol/L in ^*n*^hexane) was added to the mixture at 0 °C and the resulting mixture was stirred for 5 min. Then the mixture was heated at 100 °C with stirring for 12–24 h. Upon completion, the reaction was quenched by H_2_O and heated at 80 °C for 2 h, then extracted by ethyl acetate. The organic layer was dried over anhydrous Na_2_SO_4_ and concentrated under vacuo. The pure product was obtained by flash column chromatography on silica gel.

### Synthesis of ketones from secondary amides

To a 25 mL resealable reaction tube of solvent flask equipped with a magnetic stirring bar, secondary amide (0.25 mmol), 1,1-diborylalkane (0.50 mmol), and Al(OEt)_3_ (0.375 mmol) were added and charged with N_2_ three times. Then THF (2.0 mL) was added. The mixture was cooled down to 0 °C under nitrogen protection. Subsequently, ^*n*^BuLi (0.75 mmol, 2.5 mol/L in ^*n*^hexane) was added to the mixture at 0 °C and the resulting mixture was stirred for 5 min. Then the mixture was heated at 100 °C with stirring for 4–12 h. Upon completion, the reaction was quenched by water and was extracted by ethyl acetate. The organic layer was dried over anhydrous Na_2_SO_4_ and concentrated under vacuo. The pure product was obtained by flash column chromatography on silica gel.

### Synthesis of ketones from tertiary amides

To a 25 mL resealable reaction tube of solvent flask equipped with a magnetic stirring bar, tertiary amide (0.25 mmol), 1,1-diborylalkane (0.375 mmol) were added and charged with N_2_ three times. Then, anisole or THF (2.0 mL) was added. The mixture was cooled down to 0 °C under nitrogen protection. Subsequently, MeLi (0.375 mmol, 1.6 mol/L in Et_2_O) was added to the mixture at 0 °C and the resulting mixture was stirred for 5 min. Then the mixture was heated at 120 °C with stirring for 2–6 h. Upon completion, the reaction was quenched by water and was extracted by ethyl acetate. The organic layer was dried over anhydrous Na_2_SO_4_ and concentrated under vacuo. The pure product was obtained by flash column chromatography on silica gel.

### Synthesis of enamides from primary amides

To a 25 mL resealable reaction tube of solvent flask equipped with a magnetic stirring bar, primary amide (0.25 mmol), 1,1-diborylalkane (0.50 mmol) were added and charged with N_2_ three times. Then, THF (2.0 mL) was added. The mixture was cooled down to 0 °C under nitrogen protection. Subsequently, ^*n*^BuLi (1.0 mmol, 2.5 mol/L in ^*n*^hexane) was added to the mixture at 0 °C and the resulting mixture was stirred for 5 min. Then the mixture was heated at 100 °C with stirring for 12–24 h. When the mixture was allowed to cool to rt, ^*t*^BuOH (0.5 mL) was added in one portion. After being stirred for 0.5 h at 100 °C, TFAA (1.0 mL) was also added in one portion. The mixture was heated at 100 °C with stirring for 2 h. The reaction was quenched by careful addition of saturated NaHCO_3_ solution and was extracted by ethyl acetate. The organic layer was dried over anhydrous Na_2_SO_4_ and concentrated under vacuo. The pure product was obtained by flash column chromatography on silica gel.

### Synthesis of β-ketoamide from *N*-Boc amides

To a 25 mL resealable reaction tube of solvent flask equipped with a magnetic stirring bar, 1,1-diborylalkane (0.45 mmol) was added and charged with N_2_ three times. Then, THF (3.0 mL) was added. The mixture was cooled down to 0 °C under nitrogen protection. Subsequently, MeLi (0.45 mmol, 1.6 mol/L in Et_2_O) was added to the mixture at 0 °C and the resulting mixture was stirred for 30 min. Then *N*-Boc amide (0.30 mmol) and B(O^*i*^Pr)_3_ (0.30 mmol) were added and the mixture was heated at 80 °C with stirring for 2 h. Upon completion, the reaction was quenched by water and extracted by ethyl acetate. The organic layer was dried over anhydrous Na_2_SO_4_ and concentrated under vacuo. The pure product was obtained by flash column chromatography on silica gel.

### Synthesis of *tert*-alkylamines from tertiary lactams

To a 25 mL resealable reaction tube of solvent flask equipped with a magnetic stirring bar, tertiary lactam (0.25 mmol), 1,1-diborylalkane (0.375 mmol) were added and charged with N_2_ three times. Then, anisole (2.0 mL) was added. The mixture was cooled down to 0 °C under nitrogen protection. Subsequently, MeLi (0.375 mmol, 1.6 mol/L in Et_2_O) was added to the mixture at 0 °C and the resulting mixture was stirred for 5 min. Then the mixture was heated at 120 °C with stirring for 2 h. When the mixture was allowed to cool to room temperature, NaBH_4_ (0.75 mmol), MeOH (1.0 mL) were added in one portion. After being stirred for 1 h, the reaction was quenched by careful addition of water and was extracted by ethyl acetate. The organic layer was dried over anhydrous Na_2_SO_4_ and concentrated under vacuo. The pure product was obtained by flash column chromatography on silica gel.

## Supplementary information

Supplementary Information

## Data Availability

Data supporting the findings of this study are available within the article and its Supplementary Information files. All other relevant data are available from the corresponding author upon reasonable request.
