# Supplementary material for: Chemodivergent transformations of amides using gem-diborylalkanes as pro-nucleophiles
Source: Nat Commun. 2020 Jun 19;11:3113. doi: 10.1038/s41467-020-16948-5 (PMC7305144; doi:10.1038/s41467-020-16948-5)
Supplement: Supplementary file 1 — Supplementary Information [file 41467_2020_16948_MOESM1_ESM.pdf]

Supplementary Information

Chemodivergent Transformations of Amides using *gem*-Diborylalkanes as Pro-nucleophiles

Sun et al.

Supplementary Methods

General Information

All reactions were isolated from moisture and oxygen by a nitrogen atmosphere with a resealable reaction tube of solvent flask. All glassware was oven dried at 110 °C for hours and cooled down under vacuum. THF was purified using Pure Solv 7-SDS solvent drying system. Anisole (Superdry, water ≤ 20 ppm) was purchased from J&K without further purification. ^{n}BuLi (2.5 M in hexane) was purchased from Acros Organics. MeLi (1.6 M in Et_2O) was purchased from J&K. Unless otherwise noted, chemicals were purchased from Acros Organics, Alfa Aesar, TCI, Adamas-beta®, J&K without further purification. Thin layer chromatography (TLC) employed glass 0.25 mm silica gel plates. Flash chromatography columns were packed with 100-200 mesh silica gel or through SepaBeamTM Machine SPB-3006012. Gas chromatographic analysis were performed on GC-2010 Plus gas chromatography instrument with an FID detector. GC-MS spectra were recorded on a GCMS-QP2010 SE. The High Resolution MS analyses were performed on Agilent 6530 Accurate-Mass Q-TOF LC/MS with ESI mode. The ^{1}H (400 MHz), ^{13}C (101 MHz) and ^{19}F NMR (376 MHz) data were recorded on 400 MHz spectrometer using $CDCl_3$ or DMSO-d_6 as solvent. For $CDCl_3$, ^{1}H NMR spectra was recorded with tetramethylsilane (δ = 0.00 ppm) as the internal reference; ^{13}C NMR spectra was recorded with $CDCl_3$ (δ = 77.00 ppm) as the internal reference. For DMSO-d_6, ^{1}H NMR spectra was recorded with DMSO (δ = 2.50 ppm) as the internal reference; ^{13}C NMR spectra was recorded with DMSO (δ = 39.50 ppm) as the internal reference.

General Procedures for the Preparation of 1,1-Diborylalkanes

Synthesis of 1,1-diborylalkanes from Aldehydes or Ketones:

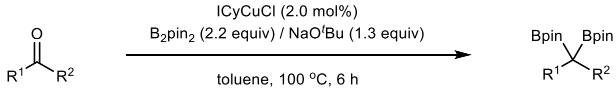

1,1-Diborylalkanes were prepared according to our previous report.[1] In a glove box, a 100 mL resealable reaction tube of solvent flask equipped with a stirrer bar was charged with B_2pin_2 (5.59 g, 22.0 mmol), NaO^{t}Bu (1.25 g, 13.0 mmol) and ICyCuCl (66.0 mg, 0.2 mmol). The tube was sealed and taken out of the glove box. Aldehyde or ketone (10.0 mmol) and toluene (20 mL) were added under the protection of nitrogen atmosphere. The tube was sealed and heated at 100 ºC with stirring for 6 h. Upon completion, the reaction was quenched by ethyl acetate. The pure product was obtained by flash column chromatography on silica gel. The isolated 1,1-diborylalkanes were analyzed by ^{1}H NMR, ^{13}C NMR and the spectrums were in accordance with the reported data to verify the identity and purity of the products.

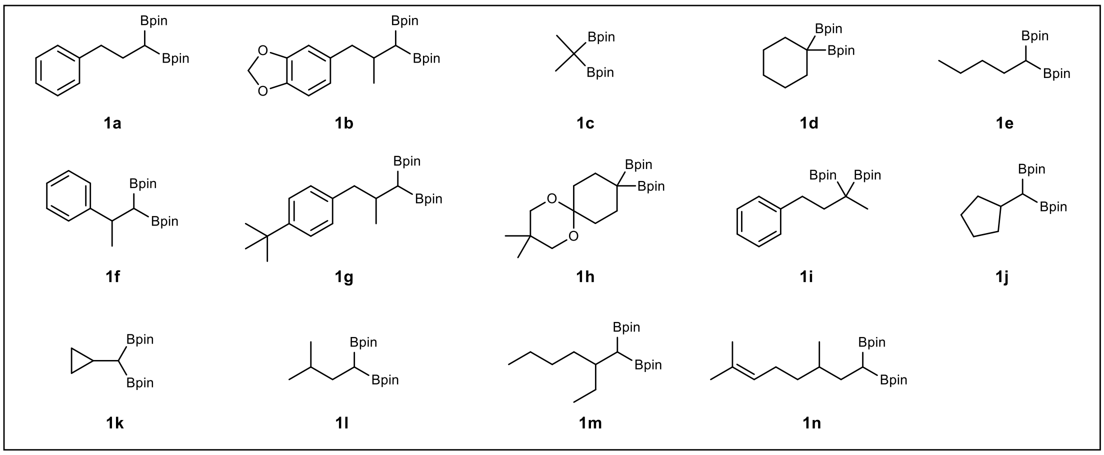

Synthesis of 1,1-diborylalkane 1o from aliphatic ester:

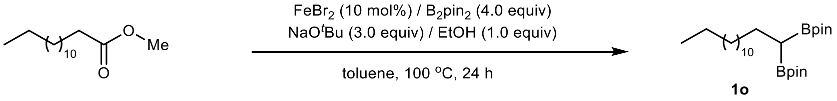

Compound **1o** was made according to our previous report.[2] In a glove box, a 100 mL resealable reaction tube of solvent flask equipped with a stirrer bar was charged with B_2pin_2 (5.08 g, 20.0 mmol), NaO^{t}Bu (1.44 g, 15.0 mmol) and $FeBr_2$ (107.8 mg, 0.5

mmol). The tube was sealed and taken out of the glove box. Methyl myristate (1.21 g, 5.0 mmol), EtOH (230.0 mg, 5.0 mmol) and toluene (20 mL) were added under nitrogen atmosphere. The tube was sealed and heated at 100 ºC with stirring for 24 h. Upon completion, the reaction mixture was cooled to room temperature, then quenched by water and extracted by ethyl acetate. The organic layer was dried over anhydrous Na_2SO_4 and concentrated under *vacuo*. The crude reaction mixture was purified on silica gel (PE: EA = 30:1) to afford **1o**. The isolated **1o** was analyzed by ^{1}H NMR, ^{13}C NMR, HRMS to verify the identity and purity of the products.

Preparation of compound 1p and 1q from the alkylation of simple 1,1-diborylalkanes:

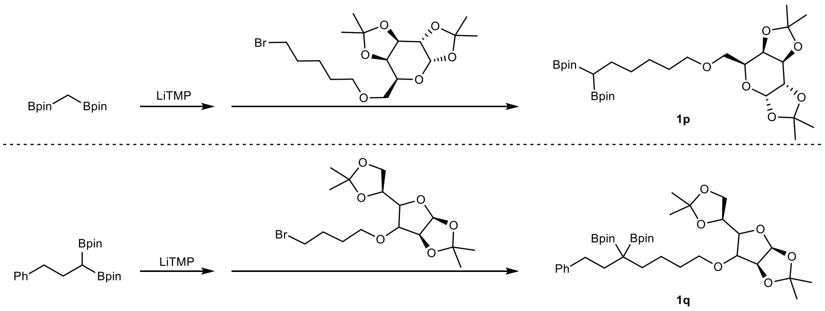

Compound **1p** and **1q** were made from the alkylation of simple 1,1-diborylalkanes according to literature report.[3] In a glove box, LiTMP (463.6 mg, 3.15 mmol for **1p**; 441.0 mg, 3.0 mmol for **1q**) was added to a 100 mL resealable reaction tube of solvent flask equipped with a magnetic stirring bar. The flask was sealed and removed from the glove box, followed by the addition of THF (15 mL) under N_2. The reaction mixture was cooled to 0 ºC, and a solution of 1,1-diborylalkane (3.0 mmol) in THF (3 mL) was added via syringe and the mixture was allowed to stir at 0 °C for 10 minutes. Alkyl bromide (3.30 mmol in the case of **1p**; 3.0 mmol in the case **1q**) in THF (3 mL) was added dropwise and the reaction was allowed to stir at 0 ºC for 15 min. Upon completion, the reaction mixture was warmed to room temperature, then quenched by water and extracted by ethyl acetate. The organic layer was dried over anhydrous Na_2SO_4 and concentrated under *vacuo*. The crude reaction mixture was purified on silica gel (PE: EA = 5:1) to afford **1p** or **1q**. The isolated **1p** and **1q** were analyzed by ^{1}H NMR, ^{13}C NMR, HRMS to verify the identity and purity of the products.

General Information for the Preparation of Amides

Amides were generally made from carboxylic acids according to literature reports by using CDI as the condensation reagent.[4] Tertiary amides bearing α-bulkiness were made according to the literature procedure by using oxalyl chloride as the condensation reagent.[5] *N*-Boc Amides were prepared from secondary amides according to the literature procedure.[6] *N*-aryl lactams were made according to the literature procedure.[7] 1-Benzylpyrrolidin-2-one was made according to the literature procedure.[8] 5-Methylphenanthridin-6(5*H*)-one was made according to the literature procedure.[9] The isolated product was analyzed by ^{1}H NMR, ^{13}C NMR and spectrums were in accord with reported data to verify the identity and purity of the products.[10-14] Other amides without noted were obtained from commercially available sources. Unknown compounds were analyzed by ^{1}H NMR, ^{13}C NMR and HRMS and the data were presented in the "detailed descriptions for unknown substrates and products" section.

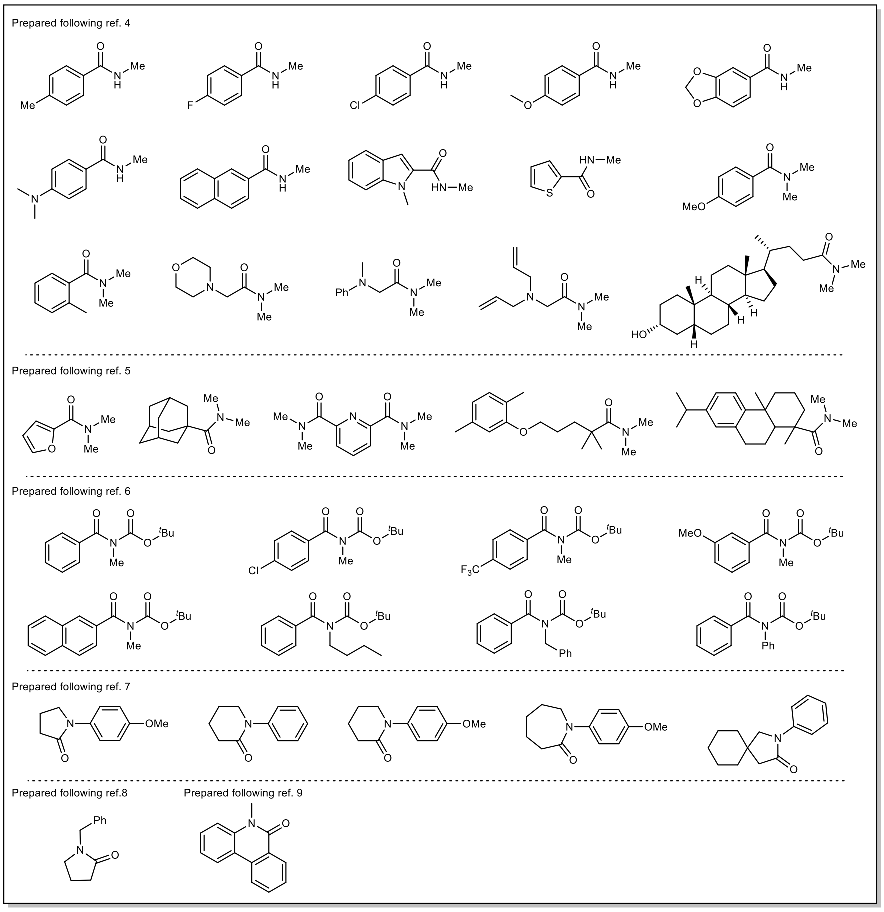

Reaction Parameters for the Transformation of Amides

Supplementary Table 1. Reaction parameters for the transformation of primary amides [a]

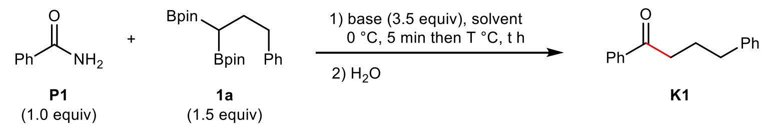

Entry	Base	Solvent	T	t	Yield (%)[b]
1	MeLi	Toluene	100	8	trace
2	MeLi	Anisole	100	8	13
3	MeLi	THF	100	8	45
4	MeLi	THF	80	8	42
5	MeLi	THF	60	8	38
6	NaO^{t}Bu	THF	100	8	0
7	^{n}BuLi	THF	100	8	59
8[c]	^{n}BuLi	THF	100	8	77
**9[c]**	**^{n}BuLi**	**THF**	**100**	**12**	**90 (85[d])**
10[c]	^{n}BuLi	THF	100	24	89

[a] Reaction conditions: **P1** (0.25 mmol), **1a** (0.375 mmol), base (3.5 equiv), solvent (2.0 mL), 0 °C, 5 min then T °C for t h. [b] Yields were determined by GC analysis using naphthalene as an internal standard. [c] **1a** (0.50 mmol, 2.0 equiv), base (4.0 equiv). [d] Isolated yield.

Supplementary Table 2. Reaction parameters for the transformation of secondary amides [a]

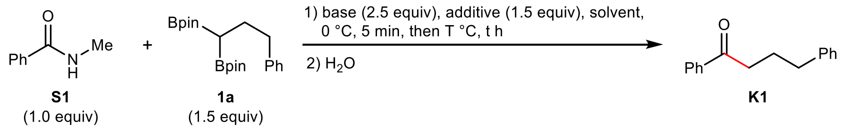

Entry	Base	Additive	Solvent	T	t	Yield (%)[b]
1	MeLi	none	THF	120	8	trace
2	MeLi	$B(OEt)_3$	THF	120	8	45
3	MeLi	$Mg(OEt)_2$	THF	120	8	25
4	MeLi	$Al(OEt)_3$	THF	120	8	67
5[c]	MeLi	$Al(OEt)_3$	THF	120	8	36
6	^{n}BuLi	$Al(OEt)_3$	THF	120	8	78
7	^{n}BuLi	$Al(OEt)_3$	DME	120	8	68
8	^{n}BuLi	$Al(OEt)_3$	Anisole	120	8	24
9	^{n}BuLi	$Al(OEt)_3$	THF	100	8	76
10	^{n}BuLi	$Al(OEt)_3$	THF	80	8	48
11	^{n}BuLi	$Al(OEt)_3$	THF	100	4	78
**12[d]**	**^{n}BuLi**	**$Al(OEt)_3$**	**THF**	**100**	**4**	**82 (78[f])**
13[e]	^{n}BuLi	$Al(OEt)_3$	THF	100	4	83

[a] Reaction conditions: **S1** (0.25 mmol), **1a** (0.375 mmol), base (2.5 equiv), additive (1.5 equiv), solvent (2.0 mL), 0 °C, 5 min then T °C for t h. [b] The yields were determined by GC analysis using naphthalene as an internal standard. [c] $Al(OEt)_3$ (0.5 equiv). [d] **1a** (2.0 equiv), base (3.0 equiv). [e] **1a** (2.5 equiv), base (3.5 equiv). [f] Isolated yield. THF = Tetrahydrofuran, DME = 1,2-Dimethoxyethane.

Supplementary Table 3. Reaction parameters for the transformation of tertiary amides [a]

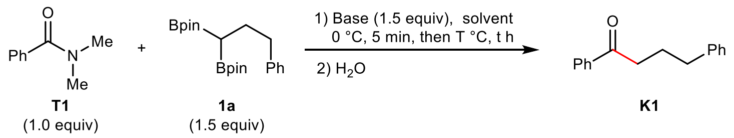

Entry	Base	Solvent	T	t	Yield (%)[b]
1	^{n}BuLi	THF	100	8	40
2	MeLi	THF	100	8	53
3	MeLi	THF	120	8	72
4	MeLi	Toluene	120	8	72
5	MeLi	DME	120	8	58
6	MeLi	^{c}Hexane	120	8	89
7	MeLi	Anisole	120	8	90
8	MeLi	Anisole	100	8	87
**9**	**MeLi**	**Anisole**	**120**	**2**	**90 (87[c])**

[a] Reaction conditions: **T1** (0.25 mmol), **1a** (0.375 mmol), MeLi (1.5 equiv), solvent (2.0 mL), 0 °C, 5 min then T °C for t h. [b] The yields were determined by GC analysis using naphthalene as an internal standard. [c] Isolated yield. THF = Tetrahydrofuran, DME = 1,2-Dimethoxyethane, ^{c}Hexane = Cyclohexane.

Supplementary Table 4. Reaction parameters of transformation of *N*-Boc amides using 1,1-diborylalkanes[a]

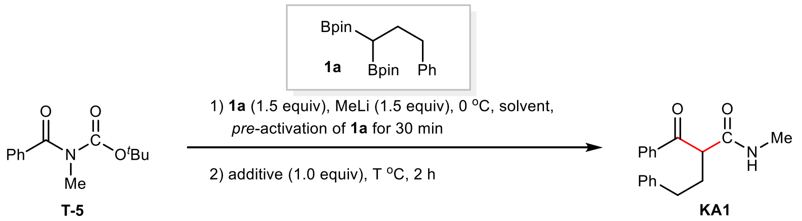

Entry	solvent	additive	T	Yield (%)[b]
1	THF	none	80	19
2	THF	$Mg(O^tBu)_2$	80	61
3	THF	$Al(OEt)_3$	80	63
4	THF	$B(O^nPr)_3$	80	55
5	THF	$B(O^iPr)_3$	80	78
**6[c]**	**THF**	$\mathbf{B(O^iPr)_3}$	**80**	**86 (78[d])**
7	THF	$B(O^iPr)_3$	100	61
8	THF	$B(O^iPr)_3$	120	53
9	DME	$B(O^iPr)_3$	80	24
10	Toluene	$B(O^iPr)_3$	80	16
11	Anisole	$B(O^iPr)_3$	80	16

[a] Reaction conditions: **1a** (0.30 mmol), MeLi (1.5 equiv), solvent (2.0 mL), 0 °C, 30 min then **T-5** (0.20 mmol), additive (1.0 equiv), T °C for 2 h. [b] The yields were determined by GC analysis using naphthalene as an internal standard. [c] **T-5** (0.30 mmol), **1a** (0.45 mmol), THF (3.0 mL). [d] Isolated yield. THF = Tetrahydrofuran, DME = 1,2-Dimethoxyethane.

General Procedure for the Transformation of Amides using 1,1-Diborylalkanes as Pro-nucleophiles

Synthesis of Ketones from Primary Amides

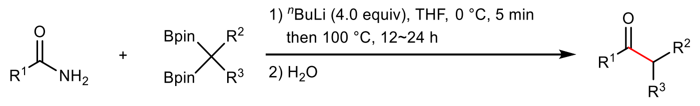

To a 25 mL resealable reaction tube of solvent flask equipped with a magnetic stirring bar, primary amide (0.25 mmol), 1,1-diborylalkane (0.50 mmol) were added and charged with N_2 three times. Then, THF (2.0 mL) was added. The mixture was cooled down to 0 ºC under nitrogen protection. Subsequently, ^{n}BuLi (1.0 mmol, 2.5 mol/L in ^{n}hexane) was added to the mixture at 0 ºC and the resulting mixture was stirred for 5 minutes. Then the mixture was heated at 100 ºC with stirring for 12 – 24 hours. Upon completion, the reaction was quenched by H_2O and heated at 80 ºC for 2 hours, then extracted by ethyl acetate. The organic layer was dried over anhydrous Na_2SO_4 and concentrated under *vacuo*. The pure product was obtained by flash column chromatography on silica gel.

Synthesis of Ketones from Secondary Amides

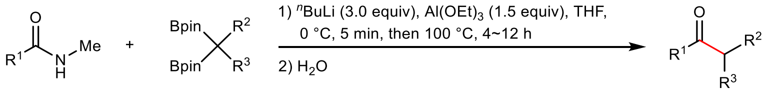

To a 25 mL resealable reaction tube of solvent flask equipped with a magnetic stirring bar, secondary amide (0.25 mmol), 1,1-diborylalkane (0.50 mmol) and $Al(OEt)_3$ (0.375 mmol) were added and charged with N_2 three times. Then THF (2.0 mL) was added. The mixture was cooled down to 0 ºC under nitrogen protection. Subsequently, ^{n}BuLi (0.75 mmol, 2.5 mol/L in ^{n}hexane) was added to the mixture at 0 ºC and the resulting mixture was stirred for 5 minutes. Then the mixture was heated at 100 ºC with stirring for 4 – 12 hours. Upon completion, the reaction was quenched by water and was extracted by ethyl acetate. The organic layer was dried over anhydrous Na_2SO_4 and concentrated under *vacuo*. The pure product was obtained by flash column chromatography on silica gel.

Synthesis of Ketones from Tertiary Amides

H_2O as the Trapping Electrophile

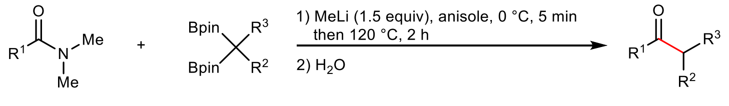

To a 25 mL resealable reaction tube of solvent flask equipped with a magnetic stirring bar, tertiary amide (0.25 mmol), 1,1-diborylalkane (0.375 mmol) were added and charged with N_2 three times. Then, anisole or THF (2.0 mL) was added. The mixture was cooled down to 0 °C under nitrogen protection. Subsequently, MeLi (0.375 mmol, 1.6 mol/L in Et_2O) was added to the mixture at 0 °C and the resulting mixture was stirred for 5 minutes. Then the mixture was heated at 120 °C with stirring for 2 – 6 hours. Upon completion, the reaction was quenched by water and was extracted by ethyl acetate. The organic layer was dried over anhydrous Na_2SO_4 and concentrated under *vacuo*. The pure product was obtained by flash column chromatography on silica gel.

D_2O, NFSI, Alkyl halides, Acyl chlorides and Aldehydes as the Trapping Electrophiles

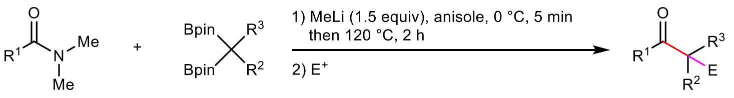

To a 25 mL resealable reaction tube of solvent flask equipped with a magnetic stirring bar, tertiary amide (0.25 mmol), 1,1-diborylalkane (0.375 mmol) were added and charged with N_2 three times. Then, anisole (2.0 mL) was added. The mixture was cooled down to 0 °C under nitrogen protection. Subsequently, MeLi (0.375 mmol, 1.6 mol/L in Et_2O) was added to the mixture at 0 °C and the resulting mixture was stirred for 5 minutes. Then the mixture was heated at 120 °C with stirring for 2 hours. Then, the reaction mixture was added with 2.0 equivalents of alkyl halides or acyl chlorides under N_2 atmosphere, then it was heated at 100 °C for 6 hours. For NFSI and MeI, the reaction mixture was stirred at room temperature for 12 hours. For aldehydes, the reaction mixtures were stirred at 40 °C for 5 hours or 12 hours. Upon completion, the reaction was quenched by water and was extracted by ethyl acetate. The organic layer was dried over anhydrous Na_2SO_4 and concentrated under *vacuo*. The pure product was obtained by flash column chromatography on silica gel.

Synthesis of Enamides from Primary Amides

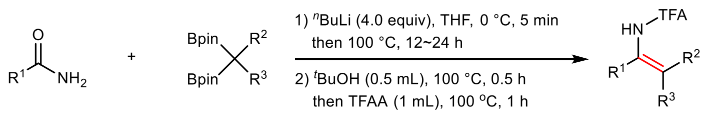

To a 25 mL resealable reaction tube of solvent flask equipped with a magnetic stirring bar, primary amide (0.25 mmol), 1,1-diborylalkane (0.50 mmol) were added and charged with N_2 three times. Then, THF (2.0 mL) was added. The mixture was cooled down to 0 ºC under nitrogen protection. Subsequently, ^{n}BuLi (1.0 mmol, 2.5 mol/L in ^{n}hexane) was added to the mixture at 0 ºC and the resulting mixture was stirred for 5 minutes. Then the mixture was heated at 100 ºC with stirring for 12~24 hours. When the mixture was allowed to cool to rt, ^{t}BuOH (0.5 mL) was added in one portion. After being stirred for 0.5 hour at 100 ºC, TFAA (1.0 mL) was also added in one portion. The mixture was heated at 100 ºC with stirring for 2 hours. The reaction was quenched by careful addition of saturated $NaHCO_3$ solution and was extracted by ethyl acetate. The organic layer was dried over anhydrous Na_2SO_4 and concentrated under *vacuo*. The pure product was obtained by flash column chromatography on silica gel.

Synthesis of *β*-Ketoamide from *N*-Boc Amides

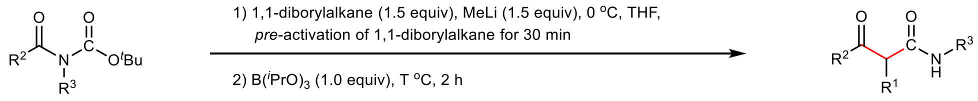

To a 25 mL resealable reaction tube of solvent flask equipped with a magnetic stirring bar, 1,1-diborylalkane (0.45 mmol) was added and charged with N_2 three times. Then, THF (3.0 mL) was added. The mixture was cooled down to 0 ºC under nitrogen protection. Subsequently, MeLi (0.45 mmol, 1.6 mol/L in Et_2O) was added to the mixture at 0 ºC and the resulting mixture was stirred for 30 minutes. Then *N*-Boc amide (0.30 mmol) and B(O^{i}Pr)$_3$ (0.30 mmol) were added and the mixture was heated at 80 ºC with stirring for 2 hours. Upon completion, the reaction was quenched by water and extracted by ethyl acetate. The organic layer was dried over anhydrous Na_2SO_4 and concentrated under *vacuo*. The pure product was obtained by flash column chromatography on silica gel.

Synthesis of *tert*-Alkylamines from Tertiary Lactams

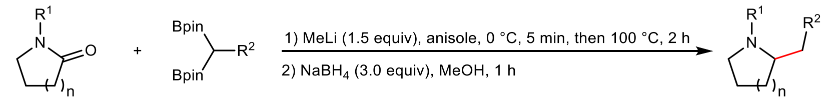

To a 25 mL resealable reaction tube of solvent flask equipped with a magnetic stirring bar, tertiary lactam (0.25 mmol), 1,1-diborylalkane (0.375 mmol) were added and charged with N_2 three times. Then, anisole (2.0 mL) was added. The mixture was cooled down to 0 ºC under nitrogen protection. Subsequently, MeLi (0.375 mmol, 1.6 mol/L in Et_2O) was added to the mixture at 0 ºC and the resulting mixture was stirred for 5 minutes. Then the mixture was heated at 120 ºC with stirring for 2 hours. When the mixture was allowed to cool to room temperature, $NaBH_4$ (0.75 mmol), MeOH (1.0 mL) were added in one portion. After being stirred for 1 hour, the reaction was quenched by careful addition of water and was extracted by ethyl acetate. The organic layer was dried over anhydrous Na_2SO_4 and concentrated under *vacuo*. The pure product was obtained by flash column chromatography on silica gel.

Gram Scale Experiments

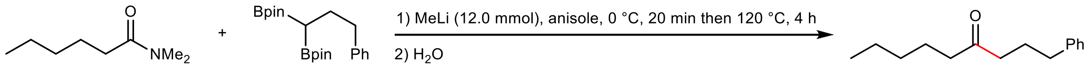

T25, 8.0 mmol　**1a**, 12.0 mmol　**K25** 1.55 g, 89% yield

To a 250 mL resealable reaction tube of solvent flask equipped with a magnetic stirring bar, **T25** (1.14 g, 8.0 mmol), **1a** (4.46 g, 12.0 mmol) were added and charged with N_2 three times. Then, anisole (50 mL) was added. The mixture was cooled down to 0 ºC under nitrogen protection. Subsequently, MeLi (12.0 mmol, 1.6 mol/L in Et_2O) was added to the mixture at 0 ºC and the resulting mixture was stirred for 20 minutes. Then the mixture was heated at 120 ºC with stirring for 4 hours. Upon completion, the reaction was quenched by water and extracted by ethyl acetate. The organic layer was dried over anhydrous Na_2SO_4 and concentrated under *vacuo*. The pure product was obtained by flash column chromatography on silica gel and give the desired product as a colorless oil (1.55 g, 89% yield).

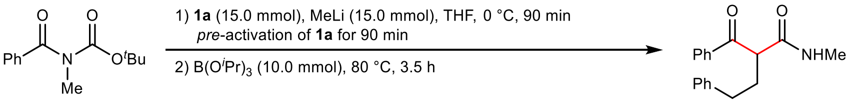

T-5 10.0 mmol　**KA1** 2.36 g, 84% yield

To a 250 mL resealable reaction tube of solvent flask equipped with a magnetic stirring bar, **1a** (5.58 g, 15.0 mmol) were added and charged with N_2 three times. Then, THF (80 mL) was added. The mixture was cooled down to 0 ºC under nitrogen protection. Subsequently, MeLi (15.0 mmol, 1.6 mol/L in Et_2O) was added to the mixture at 0 ºC and the resulting mixture was stirred for 90 minutes. Then **T5** (2.35 g, 10.0 mmol) and $B(O^iPr)_3$ (1.88 g, 10.0 mmol) were added and the mixture was heated at 80 ºC with stirring for 3.5 hours. Upon completion, the reaction was quenched by water and extracted by ethyl acetate. The organic layer was dried over anhydrous Na_2SO_4 and concentrated under *vacuo*. The pure product was obtained by flash column chromatography on silica gel and give the desired product as a white solid (2.36 g, 84% yield).

Supplementary Discussion

$H_2{}^{18}O$ Labeling Experiments

According to the general procedure for the synthesis of ketones from primary amides, secondary amides and tertiary amides. Upon completion, the reaction was quenched by ethyl acetate. Then the reaction was detected by GC-MS, and the yield was based on isolated product.

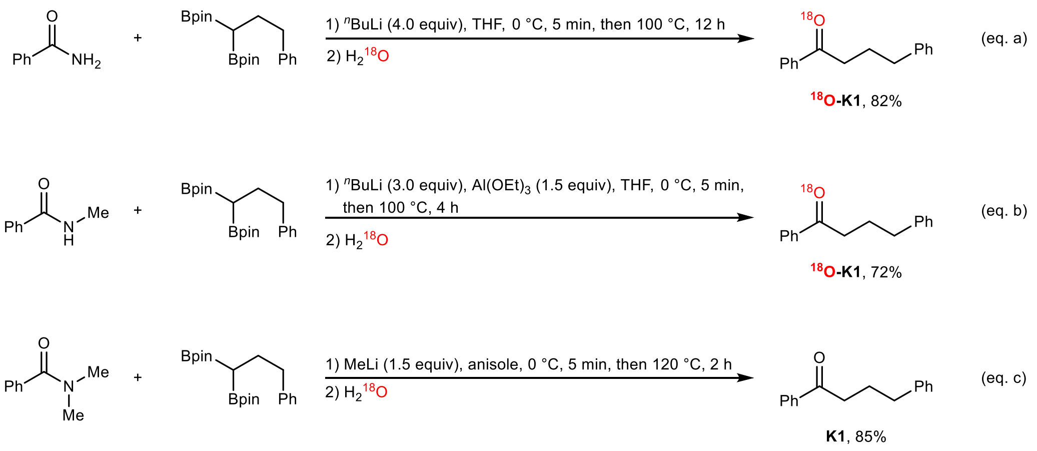

Supplementary Figure 1. MS spectra of eq. (a):

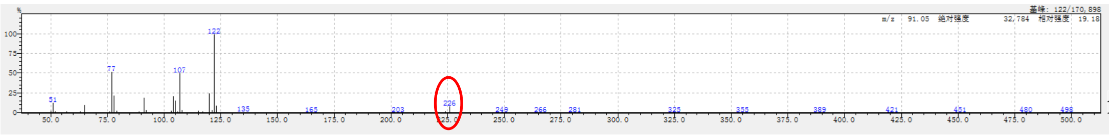

Supplementary Figure 2. MS spectra of eq. (b):

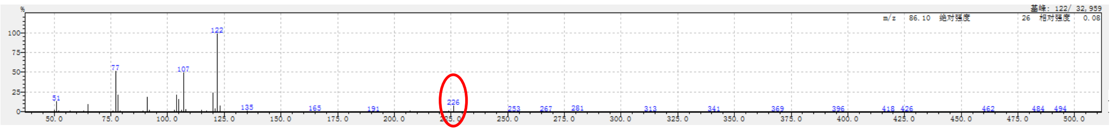

Supplementary Figure 3. MS spectra of eq. (c):

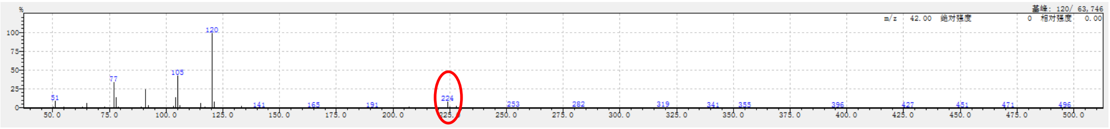

Isotopic Labeling Experiments

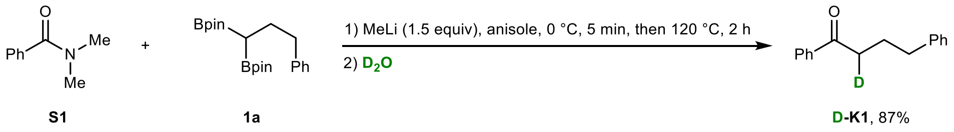

In a 25 mL resealable reaction tube of solvent flask equipped with a magnetic stirring bar, **S1** (0.25 mmol, 37.3 mg), **1a** (0.375 mmol, 139.8 mg) were added and charged with N_2 three times. Then, anisole (2.0 mL) was added. The mixture was cooled down to 0 °C under nitrogen protection. Subsequently, MeLi (0.375 mmol, 1.6 mol/L in Et_2O) was added to the mixture at 0 °C and the resulting mixture was stirred for 5 minutes. Then the mixture was heated at 120 °C with stirring for 2 hours. When the mixture was allowed to cool to room temperature, D_2O (100 µL, 5.0 mmol) was added. After being stirred for 1 hour, the reaction was quenched by water and extracted by ethyl acetate. The pure product was obtained by flash column chromatography on silica gel and give the desired product as a colorless oil (48.8 mg, 87% yield).

1,4-Diphenylbutan-1-one-2-*d* (D-K1).[15] **^{1}H NMR** (400 MHz, $CDCl_3$) δ 7.92 (d, J = 8.4 Hz, 2H), 7.58 – 7.50 (m, 1H), 7.48 – 7.40 (m, 2H), 7.32 – 7.26 (m, 2H), 7.23 – 7.16 (m, 3H), 2.99 – 2.92 (m, 1H), 2.72 (t, J = 7.6 Hz, 2H), 2.12 – 2.04 (m, 2H) ppm; **^{13}C NMR** (101 MHz, $CDCl_3$) δ 200.2, 141.7, 137.1, 132.9, 128.5(4), 128.5, 128.4, 128.0, 125.9, 37.5, 37.4, 37.2, 35.2, 25.7 ppm;

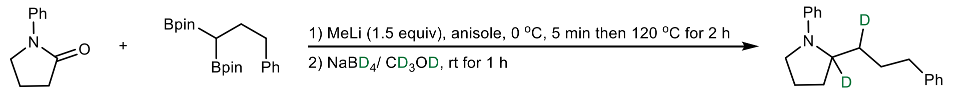

In a 25 mL resealable reaction tube of solvent flask equipped with a magnetic stirring bar, **L1** (0.25 mmol, 40.3 mg), **1a** (0.375 mmol, 139.8 mg) were added and charged with N_2 three times. Then, anisole (2.0 mL) was added. The mixture was cooled down to 0 ºC under nitrogen protection. Subsequently, MeLi (0.375 mmol, 1.6 mol/L in Et_2O) was added to the mixture at 0 ºC and the resulting mixture was stirred for 5 minutes. Then the mixture was heated at 120 ºC with stirring for 2 hours. When the mixture was allowed to cool to room temperature, $NaBD_4$ (0.75 mmol, 31.5 mg), CD_3OD (1.0 mL) were added in one portion. After being stirred for 1 hour, the reaction was quenched by careful addition of water and was extracted by ethyl acetate. The pure product was obtained by flash column chromatography on silica gel and give the desired product as a colorless oil (45.3 mg, 68% yield).

1-Phenyl-2-(3-phenylpropyl-1-*d*)pyrrolidine-2-*d* (A1-*d*). ^{1}H NMR (400 MHz, $CDCl_3$) δ 7.38 – 7.34 (m, 2H), 7.31 – 7.24 (m, 5H), 6.71 (t, J = 7.2 Hz, 1H), 6.60 (d, J = 8.0 Hz, 2H), 3.49 – 3.45 (m, 1H), 3.23 – 3.17 (m, 1H), 2.80 – 2.65 (m, 2H), 2.11 – 1.97 (m, 3H), 1.89 – 1.83 (m, 1H), 1.78 – 1.72 (m, 2H), 1.41 – 1.37 (m, 1H) ppm; **^{13}C NMR** (101 MHz, $CDCl_3$) δ 147.3, 142.4, 129.1, 128.3(3), 128.2(9), 125.7, 115.2, 111.7, 48.2, 36.0, 32.4, 32.2, 32.0, 30.1, 28.3, 23.5 ppm; **HRMS** (ESI) calcd for $C_{19}H_{21}D_2N$ $[M+H]^+$: 268.2034; found: 268.2030.

Proposed Mechanisms for Chemodivergent Transformation of Amides using 1,1-Diborylalkanes as Pro-nucleophiles

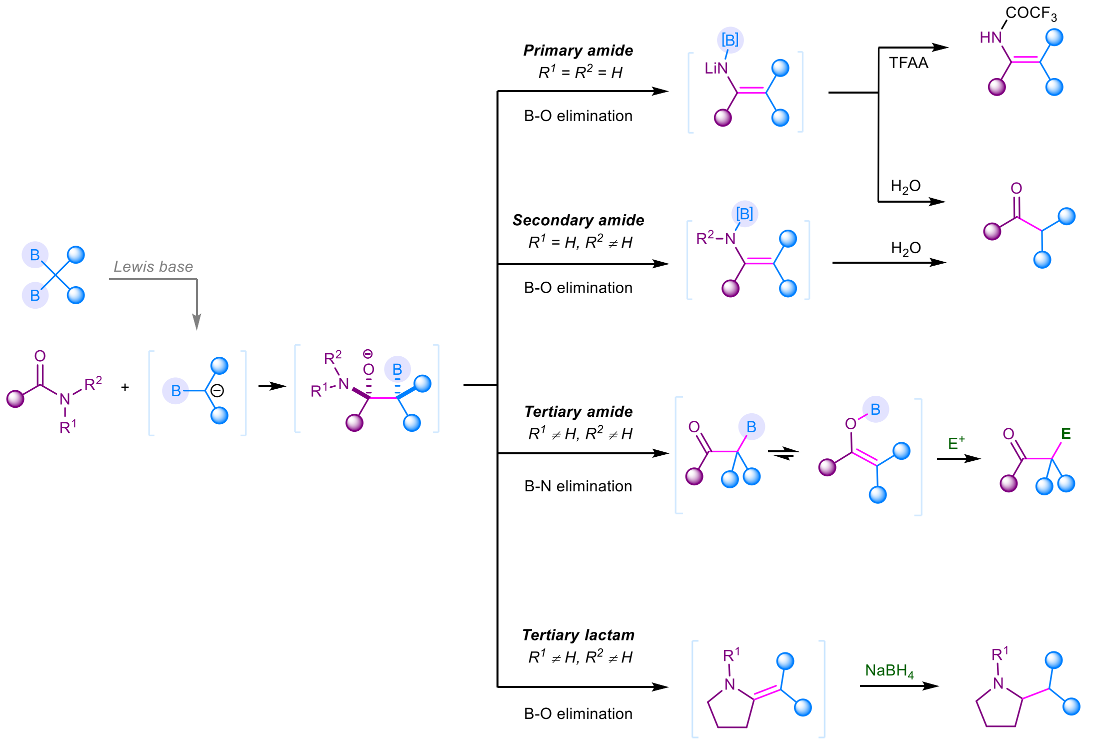

Supplementary Figure 4. Proposed mechanisms for chemodivergent transformation of amides.

Supplementary Note 1

Detailed Descriptions for Unknown Substrates and Products

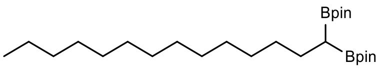

2,2'-(Tetradecane-1,1-diyl)bis(4,4,5,5-tetramethyl-1,3,2-dioxaborolane) (1o):

The title compound was obtained as a colorless oil in 1.31 g, 58% yield. **^{1}H NMR** (400 MHz, $CDCl_3$) δ 1.58 – 1.48 (m, 2H), 1.37 – 1.10 (m, 46H), 0.88 (t, *J* = 6.8 Hz, 3H), 0.88 (t, *J* = 8.0 Hz, 1H) ppm. **^{13}C NMR** (101 MHz, $CDCl_3$) δ 82.8, 32.5, 31.9, 29.7, 29.6(2), 29.6(1), 29.5(6), 29.5, 29.3, 25.6, 24.8, 24.5, 22.6, 14.1 ppm. **HRMS** (ESI) calcd for $C_{26}H_{52}B_2O_4$ $[M+H]^+$: 451.4130; found: 451.4125.

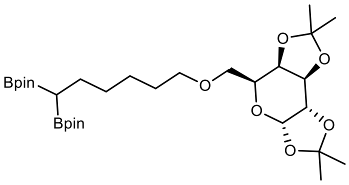

2,2'-(6-(((3aS,5S,5aR,8aR,8bS)-2,2,7,7-tetramethyltetrahydro-5H-bis([1,3]dioxolo)[4,5-b:4',5'-d]pyran-5-yl)methoxy)hexane-1,1-diyl)bis(4,4,5,5-tetramethyl-1,3,2-dioxaborolane) (1n):

The title compound was obtained as a white solid in 1.58 g, 89% yield. mp: 61-63 ºC. **^{1}H NMR** (400 MHz, $CDCl_3$) δ 5.57 – 5.48 (m, 1H), 4.64 – 4.54 (m, 1H), 4.33 – 4.22 (m, 2H), 4.00 – 3.90 (m, 1H), 3.66 – 3.59 (m, 1H), 3.58 – 3.51 (m, 1H), 3.50 – 3.38 (m, 2H), 1.59 – 1.49 (m, 7H), 1.47 – 1.42 (m, 3H), 1.36 – 1.26 (m, 10H), 1.22 (s, 24H), 0.70 (t, *J* = 7.2 Hz, 1H) ppm. **^{13}C NMR** (101 MHz, $CDCl_3$) δ 109.1, 108.5, 96.3, 82.8, 71.6, 71.1, 70.6, 69.2, 66.6, 32.4, 29.5, 26.1, 25.9(8), 25.9(6), 25.6, 24.9, 24.8, 24.5, 24.4 ppm. **HRMS** (ESI) calcd for $C_{30}H_{54}B_2O_{10}$ $[M+Na]^+$: 619.3801; found: 619.3795.

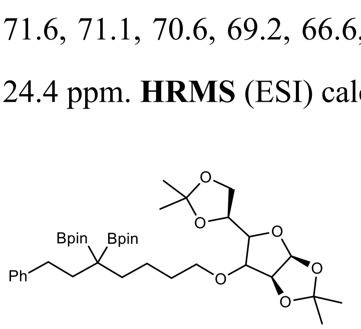

2,2'-(7-(((3aS,6aS)-5-((S)-2,2-dimethyl-1,3-dioxolan-4-yl)-2,2-dimethyltetrahydrofuro[2,3-d][1,3]dioxol-6-yl)oxy)-1-phenylheptane-3,3-diyl)bis(4,4,5,5-tetramethyl-1,3,2-dioxaborolane) (1o):

The title compound was obtained as a colorless oil in 1.68 g, 82% yield. **^{1}H NMR** (400

MHz, $CDCl_3$) δ 7.26 – 7.18 (m, 4H), 7.15 – 7.10 (m, 1H), 4.99 (s, 1H), 4.75 (dd, *J* = 6.0, 3.6 Hz, 1H), 4.57 (d, *J* = 6.0 Hz, 1H), 4.42 – 4.36 (m, 1H), 4.11 (dd, *J* = 8.8, 6.4 Hz, 1H), 4.03 (dd, *J* = 8.4, 4.4 Hz, 1H), 3.92 (dd, *J* = 7.6, 3.6 Hz, 1H), 3.68 – 3.58 (m, 1H), 3.45 – 3.35 (m, 1H), 2.55 – 2.46 (m, 2H), 1.94 – 1.86 (m, 2H), 1.75 – 1.68 (m, 2H), 1.63 – 1.55 (m, 2H), 1.46 (s, 3H), 1.44 (s, 3H), 1.37 (s, 3H), 1.34 – 1.29 (m, 5H), 1.23 (s, 24H) ppm; **^{13}C NMR** (101 MHz, $CDCl_3$) δ 143.5, 128.3, 128.0, 125.2, 112.3, 109.0, 106.1, 85.0, 82.8, 80.1, 79.4, 73.0, 67.3, 66.9, 33.7, 31.7, 29.9, 28.6, 26.8, 25.7, 25.1, 24.6(4), 24.5(6), 24.4, 23.4 ppm. **HRMS** (ESI) calcd for $C_{37}H_{60}B_2O_{10}$ $[M+Na]^+$: 709.4270; found: 709.4265.

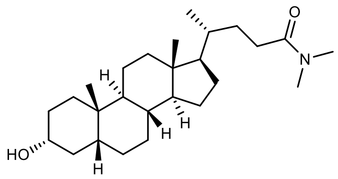

(*R*)-4-((3*R*,5*R*,8*R*,9*S*,10*S*,13*R*,14*S*,17*R*)-3-hydroxy-10,13-dimethylhexadecahydro-1H-cyclopenta[a]phenanthren-17-yl)-*N*, *N*-dimethylpentanamide

The synthesis was done in 5.0 mmol scale following literature report,[4] and the title compound was obtained as a white solid in 1.09 g, 54% yield. mp: 163-166 °C. **^{1}H NMR** (400 MHz, $CDCl_3$) 3.66 – 3.59 (m, 1H), 3.01 (s, 3H), 2.94 (s, 3H), 2.40 – 2.32 (m, 1H), 2.24 – 2.16 (m, 1H), 1.98 – 0.92 (m, 33H), 0.65 (s, 3H) ppm; **^{13}C NMR** (101 MHz, $CDCl_3$) δ 173.7, 71.8, 56.5, 56.1, 42.7, 42.1, 40.4, 40.2, 37.3, 36.5, 35.8, 35.6, 35.4, 35.3, 34.6, 31.2, 30.5, 30.3, 28.2, 27.2, 26.4, 24.2, 23.4, 20.8, 18.5, 12.0 ppm. **HRMS** (ESI) calcd for $C_{26}H_{45}NO_2$ $[M+H]^+$: 404.3529; found: 404.3519.

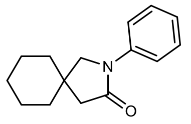

2-Phenyl-2-azaspiro[4.5]decan-3-one

The synthesis was done in 10.0 mmol scale following literature report,[7] and the title compound was obtained as a white solid in 1.72 g, 75% yield. mp: 106-108 °C. **^{1}H NMR** (400 MHz, $CDCl_3$) 7.62 – 7.59 (m, 2H), 7.38 – 7.34 (m, 2H), 7.15 – 7.11 (m, 1H), 3.59 (s, 2H), 2.46 (s, 2H), 1.61 – 1.42 (m, 10H) ppm; **^{13}C NMR** (101 MHz, $CDCl_3$) δ 173.3, 139.5, 128.7, 124.3, 119.8, 59.8, 45.3, 36.6, 35.7, 25.5, 22.7 ppm. **HRMS** (ESI)

calcd for $C_{15}H_{19}NO$ $[M+H]^+$: 230.1545; found: 230.1539.

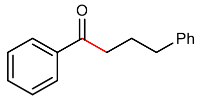

1,4-Diphenylbutan-1-one (K1).[15] Prepared according to the ***General Procedure for the Synthesis of Ketones from Primary Amides*** using benzamide (30.3 mg, 0.25 mmol), $PhCH_2CH_2CH(Bpin)_2$ (186.0 mg, 0.50 mmol), ^{n}BuLi (0.40 mL, 1.0 mmol, 2.5 M in hexane) and THF (2.0 mL), 0 °C, 5 min then 100 °C, 12 h. Upon completion, the reaction mixture was quenched with H_2O and purified by column chromatography (PE : EA = 50 : 1) to give 47.5 mg (85% yield) of product as a colorless oil. **^{1}H NMR** (400 MHz, $CDCl_3$) δ 7.93 – 7.91 (m, 2H), 7.56 – 7.52 (m, 1H), 7.46 – 7.42 (m, 2H), 7.31 – 7.27 (m, 2H), 7.22 – 7.17 (m, 3H), 2.98 (t, *J* = 7.2 Hz, 2H), 2.72 (t, *J* = 7.6 Hz, 2H), 2.15 – 2.02 (m, 2H) ppm; **^{13}C NMR** (101 MHz, $CDCl_3$) δ 200.1, 141.7, 137.0, 132.9, 128.5(3), 128.5(0), 128.4, 128.0, 125.9, 37.7, 35.2, 25.7 ppm.

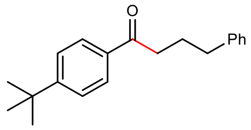

1-(4-(Tert-butyl)phenyl)-4-phenylbutan-1-one (K2).[16] Prepared according to the ***General Procedure for the Synthesis of Ketones from Primary Amides*** using 4-(*tert*-butyl)benzamide (44.3 mg, 0.25 mmol), $PhCH_2CH_2CH(Bpin)_2$ (186.0 mg, 0.50 mmol), ^{n}BuLi (0.40 mL, 1.0 mmol, 2.5 M in hexane) and THF (2.0 mL), 0 °C, 5 min then 100 °C, 12 h. Upon completion, the reaction mixture was quenched with H_2O and purified by column chromatography (PE : EA = 50 : 1) to give 57.3 mg (82% yield) of product as a colorless oil. **^{1}H NMR** (400 MHz, $CDCl_3$) δ 7.86 (d, *J* = 8.4 Hz, 2H), 7.45 (d, *J* = 8.4 Hz, 2H), 7.31 – 7.24 (m, 2H), 7.23 – 7.15 (m, 3H), 2.95 (t, *J* = 7.2 Hz, 2H), 2.71 (t, *J* = 7.6 Hz, 2H), 2.13 – 2.03 (m, 2H), 1.33 (s, 9H) ppm; **^{13}C NMR** (101 MHz, $CDCl_3$) δ 199.7, 156.6, 141.7, 134.5, 128.5, 128.3, 128.0, 125.9, 125.4, 37.6, 35.2, 35.0, 31.1, 25.8 ppm.

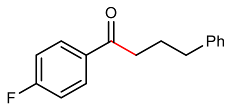

1-(4-Fluorophenyl)-4-phenylbutan-1-one (K3).[15] Prepared according to the ***General Procedure for the Synthesis of Ketones from Primary Amides*** using 4-fluorobenzamide (34.8 mg, 0.25 mmol), $PhCH_2CH_2CH(Bpin)_2$ (186.0 mg, 0.50 mmol), ^{n}BuLi (0.40 mL, 1.0 mmol, 2.5 M in hexane) and THF (2.0 mL), 0 °C, 5 min then 100 °C, 12 h. Upon completion, the reaction mixture was quenched with H_2O and purified by column chromatography (PE : EA = 50 : 1) to give 43.6 mg (72% yield) of product as a white solid, mp: 51-54 °C. **^{1}H NMR** (400 MHz, $CDCl_3$) δ 7.95 – 7.92 (m, 2H), 7.31 – 7.27 (m, 2H), 7.21 – 7.19 (m, 3H), 7.13 – 7.08 (m, 2H), 2.94 (t, *J* = 7.2 Hz, 2H), 2.72 (t, *J* = 7.6 Hz, 2H), 2.17 – 2.00 (m, 2H) ppm; **^{13}C NMR** (101 MHz, $CDCl_3$) δ 198.4, 165.6 (d, *J* = 254.4 Hz), 141.5, 133.4, 130.6 (d, *J* = 9.1 Hz), 128.5, 128.4, 126.0, 115.6 (d, *J* = 22.2 Hz), 37.5, 35.1, 25.6 ppm;

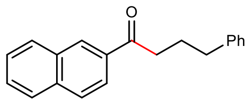

1-(Naphthalen-2-yl)-4-phenylbutan-1-one (K4).[15] Prepared according to the ***General Procedure for the Synthesis of Ketones from Primary Amides*** using 2-naphthamide (42.8 mg, 0.25 mmol), $PhCH_2CH_2CH(Bpin)_2$ (186.0 mg, 0.50 mmol), ^{n}BuLi (0.40 mL, 1.0 mmol, 2.5 M in hexane) and THF (2.0 mL), 0 °C, 5 min then 100 °C, 12 h. Upon completion, the reaction mixture was quenched with H_2O and purified by column chromatography (PE : EA = 50 : 1) to give 41.0 mg (60% yield) of product as a white solid, mp: 77-80 °C. **^{1}H NMR** (400 MHz, $CDCl_3$) δ 8.38 (s, 1H), 7.99 (d, *J* = 8.8 Hz, 1H), 7.91 (d, *J* = 8.8 Hz, 1H), 7.88 – 7.81 (m, 2H), 7.62 – 7.48 (m, 2H), 7.33 – 7.26 (m, 2H), 7.25 – 7.15 (m, 3H), 3.08 (t, *J* = 7.2 Hz, 2H), 2.75 (t, *J* = 7.6 Hz, 2H), 2.21 – 2.06 (m, 2H) ppm; **^{13}C NMR** (101 MHz, $CDCl_3$) δ 200.0, 141.7, 135.5, 134.3, 132.5, 129.6, 129.5, 128.5, 128.4, 128.3(3), 128.3(0), 127.7, 126.7, 125.9, 123.8, 37.7, 35.2, 25.8 ppm;

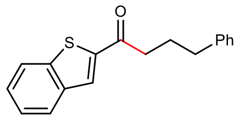

1-(Benzo[b]thiophen-2-yl)-4-phenylbutan-1-one (K5). Prepared according to the ***General Procedure for the Synthesis of Ketones from Primary Amides*** using benzo[*b*]thiophene-2-carboxamide (44.3 mg, 0.25 mmol), $PhCH_2CH_2CH(Bpin)_2$ (186.0 mg, 0.50 mmol), ^{n}BuLi (0.40 mL, 1.0 mmol, 2.5 M in hexane) and THF (2.0 mL), 0 °C, 5 min then 100 °C, 12 h. Upon completion, the reaction mixture was quenched with H_2O and purified by column chromatography (PE : EA = 20 : 1) to give 22.3 mg (32% yield) of product as a colorless oil. **^{1}H NMR** (400 MHz, $CDCl_3$) δ 7.94 – 7.74 (m, 3H), 7.51 – 7.34 (m, 2H), 7.33 – 7.17 (m, 5H), 3.01 (t, J = 7.2 Hz, 2H), 2.75 (t, J = 7.6 Hz, 2H), 2.20 – 2.05 (m, 2H) ppm; **^{13}C NMR** (101 MHz, $CDCl_3$) δ 194.6, 143.7, 142.4, 141.4, 139.1, 128.8, 128.5, 128.4, 127.3, 126.0, 125.9, 124.9, 123.0, 38.3, 35.1, 26.0 ppm. **HRMS** (ESI) calcd for $C_{18}H_{16}OS$ $[M+H]^+$: 281.1000; found: 281.0993.

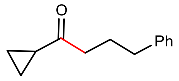

1-Cyclopropyl-4-phenylbutan-1-one (K6).[15] Prepared according to the ***General Procedure for the Synthesis of Ketones from Primary Amides*** using cyclopropanecarboxamide (21.3 mg, 0.25 mmol), $PhCH_2CH_2CH(Bpin)_2$ (186.0 mg, 0.50 mmol), ^{n}BuLi (0.40 mL, 1.0 mmol, 2.5 M in hexane) and THF (2.0 mL), 0 °C, 5 min then 100 °C, 12 h. Upon completion, the reaction mixture was quenched with H_2O and purified by column chromatography (PE : EA = 50 : 1) to give 33.8 mg (72% yield) of product as a colorless oil. **^{1}H NMR** (400 MHz, $CDCl_3$) δ 7.31 – 7.24 (m, 2H), 7.21 – 7.14 (m, 3H), 2.63 (t, J = 7.6 Hz, 2H), 2.55 (t, J = 7.6 Hz, 2H), 2.00 – 1.85 (m, 3H), 1.04 – 0.97 (m, 2H), 0.87 – 0.80 (m, 2H) ppm; **^{13}C NMR** (101 MHz, $CDCl_3$) δ 210.7, 141.7, 128.5, 128.3, 125.9, 42.6, 35.1, 25.4, 20.4, 10.5 ppm.

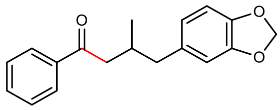

4-(Benzo[d][1,3]dioxol-5-yl)-3-methyl-1-phenylbutan-1-one (K7). Prepared

according to the ***General Procedure for the Synthesis of Ketones from Primary Amides*** using benzamide (30.3 mg, 0.25 mmol), 2,2'-(3-(benzo[d][1,3]dioxol-5-yl)-2-methylpropane-1,1-diyl)bis(4,4,5,5-tetramethyl-1,3,2-dioxaborolane) (215.0 mg, 0.50 mmol), ^{n}BuLi (0.40 mL, 1.0 mmol, 2.5 M in hexane) and THF (2.0 mL), 0 ºC, 5 min then 100 ºC, 12 h. Upon completion, the reaction mixture was quenched with H_2O and purified by column chromatography (PE : EA = 50 : 1) to give 30.4 mg (43% yield) of product as a colorless oil. **^{1}H NMR** (400 MHz, $CDCl_3$) δ 7.90 – 7.88 (m, 2H), 7.56 – 7.52 (m, 1H), 7.46 – 7.41 (m, 2H), 6.74 – 6.69 (m, 2H), 6.64 – 6.62 (m, 1H), 5.92 (s, 2H), 2.96 (dd, *J* = 16.4, 5.2 Hz, 1H), 2.74 (dd, *J* = 16.4, 7.6 Hz, 1H), 2.59 (dd, *J* = 13.2, 6.8 Hz, 1H), 2.51 – 2.38 (m, 2H), 0.96 (d, *J* = 6.4 Hz, 3H) ppm; **^{13}C NMR** (101 MHz, $CDCl_3$) δ 200.1, 147.5, 145.7, 137.3, 134.3, 132.9, 128.5, 128.0, 122.0, 109.5, 108.0, 100.7, 44.9, 43.0, 31.9, 19.9 ppm; **HRMS** (ESI) calcd for $C_{18}H_{18}O_3$ $[M+Na]^+$: 305.1154; found: 305.1149.

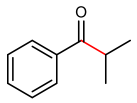

2-Methyl-1-phenylpropan-1-one (K8).[13] Prepared according to the ***General Procedure for the Synthesis of Ketones from Primary Amides*** using benzamide (30.3 mg, 0.25 mmol), 2,2'-(propane-2,2-diyl)bis(4,4,5,5-tetramethyl-1,3,2-dioxaborolane) (148.0 mg, 0.50 mmol), ^{n}BuLi (0.40 mL, 1.0 mmol, 2.5 M in hexane) and THF (2.0 mL), 0 ºC, 5 min then 100 ºC, 24 h. Upon completion, the reaction mixture was quenched with H_2O and purified by column chromatography (PE : EA = 50 : 1) to give 26.3 mg (71% yield) of product as a colorless oil. **^{1}H NMR** (400 MHz, $CDCl_3$) δ 7.99 – 7.92 (m, 2H), 7.57 – 7.50 (m, 1H), 7.49 – 7.41 (m, 2H), 3.63 – 3.48 (m, 1H), 1.22 (d, *J* = 6.8 Hz, 6H) ppm; **^{13}C NMR** (101 MHz, $CDCl_3$) δ 204.4, 136.2, 132.7, 128.5, 128.2, 35.3, 19.1 ppm.

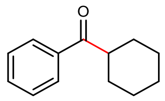

Cyclohexyl(phenyl)methanone (K9).[17] Prepared according to the ***General Procedure***

for the Synthesis of Ketones from Primary Amides using benzamide (30.3 mg, 0.25 mmol), 2,2'-(cyclohexylmethylene)bis(4,4,5,5-tetramethyl-1,3,2-dioxaborolane) (175.0 mg, 0.50 mmol), ^{n}BuLi (0.40 mL, 1.0 mmol, 2.5 M in hexane) and THF (2.0 mL), 0 °C, 5 min then 100 °C, 24 h. Upon completion, the reaction mixture was quenched with H_2O and purified by column chromatography (PE : EA = 50 : 1) to give 34.7 mg (49% yield) of product as a white solid, mp: 54 -57 °C. **^{1}H NMR** (400 MHz, $CDCl_3$) δ 7.99 – 7.86 (m, 2H), 7.58 – 7.49 (m, 1H), 7.49 – 7.40 (m, 2H), 3.33 – 3.20 (m, 1H), 1.95 – 1.71 (m, 5H), 1.57 – 1.24 (m, 5H) ppm; **^{13}C NMR** (101 MHz, $CDCl_3$) δ 203.8, 136.4, 132.7, 128.5, 128.2, 45.6, 29.4, 25.9, 25.8 ppm.

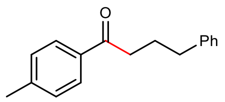

4-Phenyl-1-(p-tolyl)butan-1-one (K10).[16] Prepared according to the ***General Procedure for the Synthesis of Ketones from Secondary Amides*** using *N,* 4-dimethylbenzamide (37.3 mg, 0.25 mmol), $PhCH_2CH_2CH(Bpin)_2$ (186.0 mg, 0.50 mmol), $Al(OEt)_3$ (60.8 mg, 0.375 mmol), ^{n}BuLi (0.30 mL, 0.75 mmol, 2.5 M in hexane) and THF (2.0 mL), 0 °C, 5 min then 100 °C, 4 h. Upon completion, the reaction mixture was quenched with H_2O and purified by column chromatography (PE : EA = 50 : 1) to give 37.2 mg (63% yield) of product as a colorless oil. **^{1}H NMR** (400 MHz, $CDCl_3$) δ 7.81 (d, J = 7.6 Hz, 2H), 7.32 – 7.13 (m, 7H), 2.94 (t, J = 7.2 Hz, 2H), 2.71 (t, J = 7.6 Hz, 2H), 2.39 (s, 3H), 2.13 – 2.00 (m, 2H) ppm; **^{13}C NMR** (101 MHz, $CDCl_3$) δ 199.7, 143.6, 141.7, 134.5, 129.2, 128.5, 128.3, 128.1, 125.9, 37.5, 35.2, 25.8, 21.6 ppm.

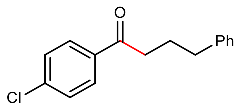

1-(4-chlorophenyl)-4-phenylbutan-1-one (K11).[16] Prepared according to the ***General Procedure for the Synthesis of Ketones from Secondary Amides*** using 4-chloro-*N*-methylbenzamide (42.3 mg, 0.25 mmol), $PhCH_2CH_2CH(Bpin)_2$ (186.0 mg, 0.50 mmol), $Al(OEt)_3$ (60.8 mg, 0.375 mmol), MeLi (0.83 mL, 0.75 mmol, 1.6 M in Et_2O) and THF (2.0 mL), 0 °C, 5 min then 100 °C, 4 h. Upon completion, the reaction mixture was

quenched with H_2O and purified by column chromatography (PE : EA = 30 : 1) to give 40.8 mg (63% yield) of product as a white solid, mp: 58-60 °C. **^{1}H NMR** (400 MHz, $CDCl_3$) δ 7.84 (d, *J* = 8.4 Hz, 2H), 7.40 (d, *J* = 8.4 Hz, 2H), 7.33 – 7.25 (m, 2H), 7.23 – 7.14 (m, 3H), 2.93 (t, *J* = 7.2 Hz, 2H), 2.71 (t, *J* = 7.6 Hz, 2H), 2.16 – 2.00 (m, 2H) ppm; **^{13}C NMR** (101 MHz, $CDCl_3$) δ 198.8, 141.5, 139.3, 135.2, 129.4, 128.8, 128.5, 128.4, 126.0, 37.6, 35.1, 25.5 ppm.

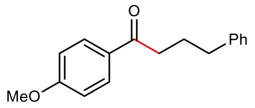

1-(4-Methoxyphenyl)-4-phenylbutan-1-one (K12).[15] Prepared according to the ***General Procedure for the Synthesis of Ketones from Secondary Amides*** using 4-methoxy-*N*-methylbenzamide (41.3 mg, 0.25 mmol), $PhCH_2CH_2CH(Bpin)_2$ (186.0 mg, 0.50 mmol), $Al(OEt)_3$ (60.8 mg, 0.375 mmol), ^{n}BuLi (0.30 mL, 0.75 mmol, 2.5 M in hexane) and THF (2.0 mL), 0 °C, 5 min then 100 °C, 24 h. Upon completion, the reaction mixture was quenched with H_2O and purified by column chromatography (PE : EA = 10 : 1) to give 38.8 mg (61% yield) of product as a white solid, mp: 56-58 °C. **^{1}H NMR** (400 MHz, $CDCl_3$) δ 7.90 (d, *J* = 8.8 Hz, 2H), 7.30 – 7.24 (m, 2H), 7.21 – 7.17 (m, 3H), 6.90 (d, *J* = 8.8 Hz, 2H), 3.8 (s, 3H), 2.92 (t, *J* = 7.2 Hz, 2H), 2.71 (t, *J* = 7.6 Hz, 2H), 2.13 – 2.01 (m, 2H) ppm; **^{13}C NMR** (101 MHz, $CDCl_3$) δ 198.7, 163.3, 141.8, 130.2, 130.1, 128.5, 128.3, 125.9, 113.7, 55.4, 37.3, 35.2, 25.9 ppm.

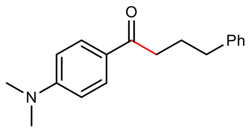

1-(4-(dimethylamino)phenyl)-4-phenylbutan-1-one (K13).[15] Prepared according to the ***General Procedure for the Synthesis of Ketones from Secondary Amides*** using 4-(dimethylamino)-*N*-methylbenzamide (44.5 mg, 0.25 mmol), $PhCH_2CH_2CH(Bpin)_2$ (186.0 mg, 0.50 mmol), $Al(OEt)_3$ (60.8 mg, 0.375 mmol), ^{n}BuLi (0.30 mL, 0.75 mmol, 2.5 M in hexane) and THF (2.0 mL), 0 °C, 5 min then 100 °C, 12 h. Upon completion, the reaction mixture was quenched with H_2O and purified by column chromatography (PE : EA = 10 : 1) to give 30.7 mg (46% yield) of product as a white solid, mp: 98-100

°C. ^{1}H NMR (400 MHz, $CDCl_3$) δ 7.84 (d, *J* = 8.8 Hz, 2H), 7.31 – 7.24 (m, 2H), 7.22 – 7.14 (m, 3H), 6.62 (d, *J* = 9.2 Hz, 2H), 3.02 (s, 6H), 2.88 (t, *J* = 7.2 Hz, 2H), 2.70 (t, *J* = 7.6 Hz, 2H), 2.11 – 2.00 (m, 2H) ppm; ^{13}C NMR (101 MHz, $CDCl_3$) δ 198.2, 153.3, 142.0, 130.2, 128.5, 128.3, 125.8, 125.1, 110.6, 39.9, 37.0, 35.4, 26.3 ppm.

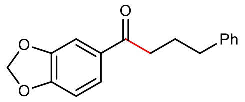

1-(Benzo[*d*][1,3]dioxol-5-yl)-4-phenylbutan-1-one (K14). Prepared according to the ***General Procedure for the Synthesis of Ketones from Secondary Amides*** using *N*-methylbenzo[*d*][1,3]dioxole-5-carboxamide (44.8 mg, 0.25 mmol), $PhCH_2CH_2CH(Bpin)_2$ (186.0 mg, 0.50 mmol), $Al(OEt)_3$ (60.8 mg, 0.375 mmol), ^{n}BuLi (0.30 mL, 0.75 mmol, 2.5 M in hexane) and THF (2.0 mL), 0 °C, 5 min then 100 °C, 12 h. Upon completion, the reaction mixture was quenched with H_2O and purified by column chromatography (PE : EA = 10 : 1) to give 30.3 mg (46% yield) of product as a colorless oil. **^{1}H NMR** (400 MHz, $CDCl_3$) δ 7.50 (dd, *J* = 8.0, 2.0 Hz, 1H), 7.40 (d, *J* = 2.0 Hz, 1H), 7.31 – 7.25 (m, 2H), 7.23 – 7.14 (m, 3H), 6.81 (d, *J* = 8.4 Hz, 1H), 6.01 (s, 2H), 2.88 (t, *J* = 7.2 Hz, 2H), 2.70 (t, *J* = 7.6 Hz, 2H), 2.11 – 2.00 (m, 2H) ppm; **^{13}C NMR** (101 MHz, $CDCl_3$) δ 198.1, 151.6, 148.1, 141.7, 131.9, 128.5, 128.4, 125.9, 124.2, 107.9, 107.8, 101.7, 37.4, 35.2, 25.9 ppm. **HRMS** (ESI) calcd for $C_{17}H_{16}O_3$ $[M+H]^+$: 269.1178; found: 269.1173.

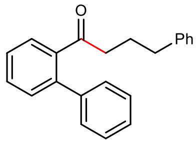

1-([1,1'-Biphenyl]-2-yl)-4-phenylbutan-1-one (K15). Prepared according to the ***General Procedure for the Synthesis of Ketones from Secondary Amides*** using *N*-methyl-[1,1'-biphenyl]-2-carboxamide (52.8 mg, 0.25 mmol), $PhCH_2CH_2CH(Bpin)_2$ (186.0 mg, 0.50 mmol), $Al(OEt)_3$ (60.8 mg, 0.375 mmol), ^{n}BuLi (0.30 mL, 0.75 mmol, 2.5 M in hexane) and THF (2.0 mL), 0 °C, 5 min then 100 °C, 4 h. Upon completion, the reaction mixture was quenched with H_2O and purified by column chromatography

(PE : EA = 30 : 1) to give 22.5 mg (30% yield) of product as a colorless oil. **^{1}H NMR** (400 MHz, $CDCl_3$) δ 7.52 – 7.43 (m, 2H), 7.42 – 7.34 (m, 5H), 7.32 – 7.28 (m, 2H), 7.23 – 7.17 (m, 2H), 7.16 – 7.10 (m, 1H), 7.02 – 6.95 (m, 2H), 2.37 (t, *J* = 7.6 Hz, 2H), 2.24 (t, *J* = 7.2 Hz, 2H), 1.79 – 1.69 (m, 2H) ppm; **^{13}C NMR** (101 MHz, $CDCl_3$) δ 207.7, 141.5, 141.1, 140.6, 140.1, 130.4, 130.1, 128.8, 128.6, 128.3, 128.2, 127.8, 127.6, 127.4, 125.8, 42.3, 35.0, 26.1 ppm. **HRMS** (ESI) calcd for $C_{22}H_{20}O$ $[M+Na]^+$: 323.1412; found: 323.1410.

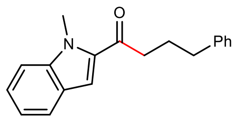

1-(1-Methyl-1H-indol-2-yl)-4-phenylbutan-1-one (K16).[15] Prepared according to the ***General Procedure for the Synthesis of Ketones from Secondary Amides*** using *N*,1-dimethyl-1*H*-indole-2-carboxamide (47.0 mg, 0.25 mmol), $PhCH_2CH_2CH(Bpin)_2$ (186.0 mg, 0.50 mmol), $Al(OEt)_3$ (60.8 mg, 0.375 mmol), ^{n}BuLi (0.30 mL, 0.75 mmol, 2.5 M in hexane) and THF (2.0 mL), 0 °C, 5 min then 100 °C, 12 h. Upon completion, the reaction mixture was quenched with H_2O and purified by column chromatography (PE : EA = 20 : 1) to give 38.1 mg (55% yield) of product as a colorless oil. **^{1}H NMR** (400 MHz, $CDCl_3$) δ 7.66 (d, *J* = 8.4 Hz, 1H), 7.36 (d, *J* = 4.0 Hz, 2H), 7.32 – 7.26 (m, 2H), 7.24 – 7.17 (m, 4H), 7.17 – 7.12 (m, 1H), 4.06 (s, 3H), 2.97 (t, *J* = 7.2 Hz, 2H), 2.73 (t, *J* = 7.6 Hz, 2H), 2.15 – 2.05 (m, 2H) ppm; **^{13}C NMR** (101 MHz, $CDCl_3$) δ 194.2, 141.6, 140.0, 134.8, 128.5, 128.4, 125.9, 125.8, 125.7, 122.8, 120.6, 111.2, 110.3, 39.1, 35.3, 32.2, 26.5 ppm.

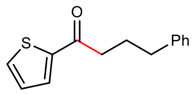

4-Phenyl-1-(thiophen-2-yl)butan-1-one (K17). Prepared according to the ***General Procedure for the Synthesis of Ketones from Secondary Amides*** using *N*-methylthiophene-2-carboxamide (35.3 mg, 0.25 mmol), $PhCH_2CH_2CH(Bpin)_2$ (186.0 mg, 0.50 mmol), $Al(OEt)_3$ (60.8 mg, 0.375 mmol), ^{n}BuLi (0.30 mL, 0.75 mmol, 2.5 M in hexane) and THF (2.0 mL), 0 °C, 5 min then 100 °C, 12 h. Upon completion, the

reaction mixture was quenched with H_2O and purified by column chromatography (PE : EA = 20 : 1) to give 20.1 mg (35% yield) of product as a colorless oil. **^{1}H NMR** (400 MHz, $CDCl_3$) δ 7.63 (dd, *J* = 2.8, 1.2 Hz, 1H), 7.60 (dd, *J* = 4.8, 1.2 Hz, 1H), 7.33 – 7.25 (m, 2H), 7.23 – 7.16 (m, 3H), 7.10 (dd, *J* = 4.8, 3.6 Hz, 1H), 2.90 (t, *J* = 7.2 Hz, 2H), 2.71 (t, *J* = 7.6 Hz, 2H), 2.15 – 2.03 (m, 2H) ppm; **^{13}C NMR** (101 MHz, $CDCl_3$) δ 193.0, 144.4, 141.5, 133.3, 131.7, 128.5, 128.4, 128.0, 126.0, 38.5, 35.1, 26.1 ppm. **HRMS** (ESI) calcd for $C_{14}H_{14}OS$ $[M+Na]^+$: 253.0663; found: 253.0660.

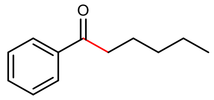

1-Phenylhexan-1-one (K18).[18] Prepared according to the ***General Procedure for the Synthesis of Ketones from Secondary Amides*** using *N*-methylbenzamide (33.8 mg, 0.25 mmol), 2,2'-(pentane-1,1-diyl)bis(4,4,5,5-tetramethyl-1,3,2-dioxaborolane) (162.0 mg, 0.75 mmol), $Al(OEt)_3$ (60.8 mg, 0.375 mmol), ^{n}BuLi (0.30 mL, 0.50 mmol, 2.5 M in hexane) and THF (2.0 mL), 0 °C, 5 min then 100 °C, 4 h. Upon completion, the reaction mixture was quenched with H_2O and purified by column chromatography (PE : EA = 30 : 1) to give 23.4 mg (53% yield) of product as a colorless oil. **^{1}H NMR** (400 MHz, $CDCl_3$) δ 8.00 – 7.93 (m, 2H), 7.60 – 7.51 (m, 1H), 7.50 – 7.41 (m, 2H), 2.96 (t, *J* = 7.2 Hz, 2H), 1.80 – 1.70 (m, 2H), 1.41 – 1.31 (m, 4H), 0.97 – 0.87 (m, 3H) ppm; **^{13}C NMR** (101 MHz, $CDCl_3$) δ 200.6, 137.1, 132.8, 128.5, 128.0, 38.6, 31.5, 24.1, 22.5, 13.9 ppm.

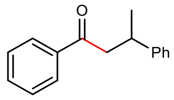

1,3-Diphenylbutan-1-one (K19).[19] Prepared according to the ***General Procedure for the Synthesis of Ketones from Secondary Amides*** using *N*-methylbenzamide (33.8 mg, 0.25 mmol), 2,2'-(2-phenylpropane-1,1-diyl)bis(4,4,5,5-tetramethyl-1,3,2-dioxaborolane) (186.0 mg, 0.50 mmol), $Al(OEt)_3$ (60.8 mg, 0.375 mmol), ^{n}BuLi (0.30 mL, 0.75 mmol, 2.5 M in hexane) and THF (2.0 mL), 0 °C, 5 min then 100 °C, 4 h. Upon completion, the reaction mixture was quenched with H_2O and purified by column

chromatography (PE : EA = 30 : 1) to give 34.4 mg (61% yield) of product as a white solid, mp: 26-28 ºC. **^{1}H NMR** (400 MHz, $CDCl_3$) δ 7.96 – 7.88 (m, 2H), 7.57 – 7.50 (m, 1H), 7.46 – 7.39 (m, 2H), 7.34 – 7.25 (m, 4H), 7.22 – 7.14 (m, 1H), 3.57 – 3.45 (m, 1H), 3.29 (dd, *J* = 16.4, 5.6 Hz, 1H), 3.18 (dd, *J* = 16.4, 8.4 Hz, 1H), 1.34 (d, *J* = 6.8 Hz, 3H) ppm; **^{13}C NMR** (101 MHz, $CDCl_3$) δ 199.0, 146.6, 137.2, 132.9, 128.5(2), 128.5(0), 128.0, 126.8, 126.2, 47.0, 35.6, 21.8 ppm.

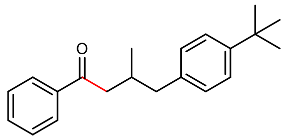

4-(4-(*tert*-butyl)phenyl)-3-methyl-1-phenylbutan-1-one (K20). Prepared according to the ***General Procedure for the Synthesis of Ketones from Secondary Amides*** using *N*-methylbenzamide (33.8 mg, 0.25 mmol), 2,2'-(3-(4-(tert-butyl)phenyl)-2-methylpropane-1,1-diyl)bis(4,4,5,5-tetramethyl-1,3,2-dioxaborolane) (221.1 mg, 0.50 mmol), $Al(OEt)_3$ (60.8 mg, 0.375 mmol), ^{n}BuLi (0.30 mL, 0.75 mmol, 2.5 M in hexane) and THF (2.0 mL), 0 ºC, 5 min then 100 ºC, 4 h. Upon completion, the reaction mixture was quenched with H_2O and purified by column chromatography (PE : EA = 30 : 1) to give 37.9 mg (52% yield) of product as a white solid, mp: 59-61 ºC. **^{1}H NMR** (400 MHz, $CDCl_3$) δ 7.89 – 7.81 (m, 2H), 7.56 – 7.48 (m, 1H), 7.45 – 7.37 (m, 2H), 7.33 – 7.26 (m, 2H), 7.14 – 7.08 (m, 2H), 2.98 (dd, *J* = 16.0, 5.2 Hz, 1H), 2.73 (dd, *J* = 16.0, 8.0 Hz, 1H), 2.66 – 2.52 (m, 2H), 2.52 – 2.42 (m, 1H), 1.31 (s, 9H), 0.98 (d, *J* = 6.8 Hz, 3H) ppm; **^{13}C NMR** (101 MHz, $CDCl_3$) δ 200.1, 148.8, 137.4, 137.3, 132.8, 128.9, 128.5, 128.1, 125.1, 45.0, 42.9, 34.3, 31.8, 31.4, 20.1 ppm. **HRMS** (ESI) calcd for $C_{21}H_{26}O$ $[M+Na]^+$: 317.1881; found: 317.1878.

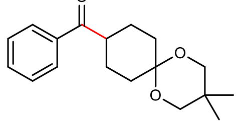

(3,3-dimethyl-1,5-dioxaspiro[5.5]undecan-9-yl)(phenyl)methanone (K21). Prepared according to the ***General Procedure for the Synthesis of Ketones from***

Secondary Amides using *N*-methylbenzamide (33.8 mg, 0.25 mmol), 2,2'-(3,3-dimethyl-1,5-dioxaspiro[5.5]undecane-9,9-diyl)bis(4,4,5,5-tetramethyl-1,3,2-dioxaborolane) (218.0 mg, 0.50 mmol), $Al(OEt)_3$ (60.8 mg, 0.375 mmol), ^{n}BuLi (0.30 mL, 0.75 mmol, 2.5 M in hexane) and THF (2.0 mL), 0 °C, 5 min then 100 °C, 4 h. Upon completion, the reaction mixture was quenched with H_2O and purified by column chromatography (PE : EA = 10 : 1) to give 39.1 mg (54% yield) of product as a white solid, mp: 94-97 °C. **^{1}H NMR** (400 MHz, $CDCl_3$) δ 7.94 (d, *J* = 7.2 Hz, 2H), 7.55 (t, *J* = 7.2 Hz, 1H), 7.46 (t, *J* = 7.6 Hz, 2H), 3.55 (s, 2H), 3.49 (s, 2H), 3.35 – 3.23 (m, 1H), 2.38 – 2.29 (m, 2H), 1.88 – 1.78 (m, 4H), 1.57 – 1.45 (m, 2H), 0.97 (s, 6H) ppm; **^{13}C NMR** (101 MHz, $CDCl_3$) δ 203.0, 136.3, 132.8, 128.6, 128.2, 96.9, 70.1, 69.8, 44.7, 31.5, 30.2, 25.4, 22.7 ppm. **HRMS** (ESI) calcd for $C_{18}H_{24}O_3$ $[M+H]^+$: 289.1804; found: 289.1799.

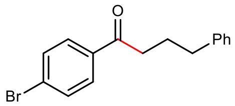

1-(4-Bromophenyl)-4-phenylbutan-1-one (K22). Prepared according to the ***General Procedure for the Synthesis of Ketones from Tertiary Amides*** using 4-bromo-*N*, *N*-dimethylbenzamide (57.0 mg, 0.25 mmol), $PhCH_2CH_2CH(Bpin)_2$ (139.8 mg, 0.375 mmol), MeLi (0.24 mL, 0.375 mmol, 1.6 M in Et_2O) and anisole (2.0 mL), 0 °C, 5 min then 120 °C, 2 h. Upon completion, the reaction mixture was quenched with H_2O and purified by column chromatography (PE : EA = 50 : 1) to give 59.9 mg (80% yield) of product as a white solid, mp: 61-64 °C. **^{1}H NMR** (400 MHz, $CDCl_3$) δ 7.76 (d, *J* = 8.4 Hz, 2H), 7.57 (d, *J* = 8.4 Hz, 2H), 7.31 – 7.27 (m, 2H), 7.21 – 7.18 (m, 3H), 2.92 (t, *J* = 7.2 Hz, 2H), 2.71 (t, *J* = 7.6 Hz, 2H), 2.12 – 2.02 (m, 2H) ppm; **^{13}C NMR** (101 MHz, $CDCl_3$) δ 199.0, 141.5, 135.6, 131.8, 129.5, 128.5, 128.4, 128.0, 126.0, 37.5, 35.0, 25.5 ppm; **HRMS** (ESI) calcd for $C_{16}H_{15}BrO$ $[M+Na]^+$: 325.0204; found: 325.0200.

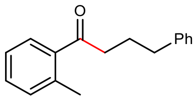

4-Phenyl-1-(o-tolyl)butan-1-one (K23). Prepared according to the ***General***

Procedure for the Synthesis of Ketones from Tertiary Amides using *N*, *N*, 2-trimethylbenzamide (40.8 mg, 0.25 mmol), $PhCH_2CH_2CH(Bpin)_2$ (139.8 mg, 0.375 mmol), MeLi (0.24 mL, 0.375 mmol, 1.6 M in Et_2O) and anisole (2.0 mL), 0 °C, 5 min then 120 °C, 2 h. Upon completion, the reaction mixture was quenched with H_2O and purified by column chromatography (PE : EA = 50 : 1) to give 48.0 mg (80% yield) of product as a colorless oil. **^{1}H NMR** (400 MHz, $CDCl_3$) δ 7.72 – 7.70 (m, 2H), 7.36 – 7.17 (m, 7H), 2.96 (t, *J* = 7.2 Hz, 2H), 2.72 (t, *J* = 7.6 Hz, 2H), 2.39 (s, 3H), 2.14 – 2.03 (m, 2H) ppm; **^{13}C NMR** (101 MHz, $CDCl_3$) δ 200.3, 141.7, 138.7, 137.0, 133.7, 128.5(1), 128.4(9), 128.3(8), 128.3(5), 125.9, 125.2, 37.7, 35.2, 25.7, 21.3 ppm; **HRMS** (ESI) calcd for $C_{17}H_{18}O$ $[M+Na]^+$: 261.1255; found: 261.1256.

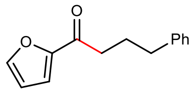

1-(Furan-2-yl)-4-phenylbutan-1-one (K24).[15] Prepared according to the ***General Procedure for the Synthesis of Ketones from Tertiary Amides*** using *N, N*-dimethylhexanamide (37.8 mg, 0.25 mmol), $PhCH_2CH_2CH(Bpin)_2$ (139.8 mg, 0.375 mmol), MeLi (0.24 mL, 0.375 mmol, 1.6 M in Et_2O) and anisole (2.0 mL), 0 °C, 5 min then 120 °C, 2 h. Upon completion, the reaction mixture was quenched with H_2O and purified by column chromatography (PE : EA = 20 : 1) to give 41.0 mg (77% yield) of product as a white solid, mp: 63-67 °C. **^{1}H NMR** (400 MHz, $CDCl_3$) δ 7.60 – 7.50 (m, 1H), 7.32 – 7.24 (m, 2H), 7.23 – 7.15 (m, 3H), 7.12 (t, *J* = 3.6 Hz, 1H), 6.55 – 6.43 (m, 1H), 2.83 (t, *J* = 7.2 Hz, 2H), 2.70 (t, *J* = 7.6 Hz, 2H), 2.13 – 1.99 (m, 2H) ppm; **^{13}C NMR** (101 MHz, $CDCl_3$) δ 189.3, 152.7, 146.2, 141.5, 128.4, 128.3, 125.9, 116.8, 112.1, 37.6, 35.1, 25.6 ppm.

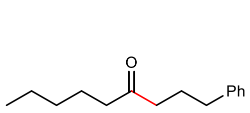

Phenylnonan-4-one (K25).[15] Prepared according to the ***General Procedure for the Synthesis of Ketones from Tertiary Amides*** using *N, N*-dimethylhexanamide (35.8 mg, 0.25 mmol), $PhCH_2CH_2CH(Bpin)_2$ (139.8 mg, 0.375 mmol), MeLi (0.24 mL, 0.375 mmol, 1.6 M in Et_2O) and anisole (2.0 mL), 0 °C, 5 min then 120 °C, 2 h. Upon

completion, the reaction mixture was quenched with H_2O and purified by column chromatography (PE : EA = 50 : 1) to give 43.5 mg (80% yield) of product as a colorless oil. **^{1}H NMR** (400 MHz, $CDCl_3$) δ 7.30 – 7.25 (m, 2H), 7.20 – 7.16 (m, 3H), 2.61 (t, *J* = 7.2 Hz, 2H), 2.42 – 2.34 (m, 4H), 1.96 – 1.83 (m, 2H), 1.62 – 1.48 (m, 2H), 1.33 – 1.22 (m, 4H), 0.88 (t, *J* = 7.2 Hz, 3H) ppm; **^{13}C NMR** (101 MHz, $CDCl_3$) δ 211.2, 141.6, 128.4, 128.3, 125.9, 42.8, 41.8, 35.1, 31.4, 25.2, 23.5, 22.4, 13.9 ppm.

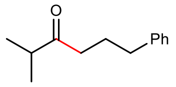

2-methyl-6-phenylhexan-3-one (K26).[20] Prepared according to the ***General Procedure for the Synthesis of Ketones from Tertiary Amides*** using *N*, *N*-dimethylisobutyramide (28.8 mg, 0.25 mmol), $PhCH_2CH_2CH(Bpin)_2$ (139.8 mg, 0.375 mmol), MeLi (0.24 mL, 0.375 mmol, 1.6 M in Et_2O) and anisole (2.0 mL), 0 °C, 5 min then 120 °C, 2 h. Upon completion, the reaction mixture was quenched with H_2O and purified by column chromatography (PE : EA = 50 : 1) to give 37.7 mg (79% yield) of product as a colorless oil. **^{1}H NMR** (400 MHz, $CDCl_3$) δ 7.30 – 7.26 (m, 2H), 7.20 – 7.16 (m, 3H), 2.63 – 2.53 (m, 3H), 2.46 (t, *J* = 7.2 Hz, 2H), 1.97 – 1.85 (m, 2H), 1.07 (d, *J* = 6.8 Hz, 6H) ppm; **^{13}C NMR** (101 MHz, $CDCl_3$) δ 214.6, 141.7, 128.4, 128.3, 125.9, 40.8, 39.4, 35.1, 25.2, 18.2 ppm.

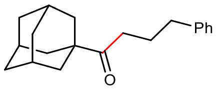

1-((3r,5r,7r)-Adamantan-1-yl)-4-phenylbutan-1-one (K27). Prepared according to the ***General Procedure for the Synthesis of Ketones from Tertiary Amides*** using (*3r*,*5r*,*7r*)-*N*, *N*-dimethyladamantane-1-carboxamide (51.8 mg, 0.25 mmol), $PhCH_2CH_2CH(Bpin)_2$ (139.8 mg, 0.375 mmol), MeLi (0.24 mL, 0.375 mmol, 1.6 M in Et_2O) and THF (2.0 mL), 0 °C, 5 min then 100 °C, 6 h. Upon completion, the reaction mixture was quenched with H_2O and purified by column chromatography (PE : EA = 50 : 1) to give 53.0 mg (75% yield) of product as a colorless oil. **^{1}H NMR** (400 MHz, $CDCl_3$) δ 7.29 – 7.25 (m, 2H), 7.19 – 7.16 (m, 3H), 2.61 – 2.57 (m, 2H), 2.45 (t, *J* = 7.2

Hz, 2H), 2.04 – 2.01 (m, 3H), 1.91 – 1.83 (m, 2H), 1.78 – 1.65 (m, 12H) ppm; **^{13}C NMR** (101 MHz, $CDCl_3$) δ 215.4, 141.9, 128.4, 128.3, 125.8, 46.3, 38.2, 36.5, 35.2, 35.1, 27.9, 25.1 ppm; **HRMS** (ESI) calcd for $C_{20}H_{26}O$ $[M+Na]^+$: 305.1881; found: 305.1880.

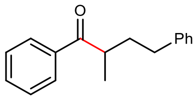

2-Methyl-1,4-diphenylbutan-1-one (K28).[21] Prepared according to the ***General Procedure for the Synthesis of Ketones from Tertiary Amides*** using *N*, *N*-dimethylbenzamide (37.5 mg, 0.25 mmol), 2,2'-(4-phenylbutane-2,2-diyl)bis(4,4,5,5-tetramethyl-1,3,2-dioxaborolane) (144.8 mg, 0.375 mmol), MeLi (0.24 mL, 0.375 mmol, 1.6 M in Et_2O) and anisole (2.0 mL), 0 °C, 5 min then 120 °C, 2 h. Upon completion, the reaction mixture was quenched with H_2O and purified by column chromatography (PE : EA = 50 : 1) to give 45.4 mg (76% yield) of product as a colorless oil. **^{1}H NMR** (400 MHz, $CDCl_3$) δ 7.87 – 7.85 (m, 2H), 7.56 – 7.52 (m, 1H), 7.45 – 7.41 (m, 2H), 7.29 – 7.24 (m, 2H), 7.20 – 7.13 (m, 3H), 3.51 – 3.42 (m, 1H), 2.70 – 2.59 (m, 2H), 2.22 – 2.13 (m, 1H), 1.79 – 1.66 (m, 1H), 1.23 (d, *J* = 7.2 Hz, 3H) ppm; **^{13}C NMR** (101 MHz, $CDCl_3$) δ 204.1, 141.7, 136.5, 132.9, 128.6, 128.4, 128.3, 128.2, 125.9, 39.7, 35.1, 33.4, 17.3 ppm.

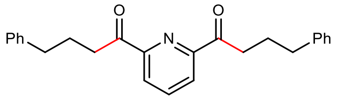

1,1'-(pyridine-2,6-diyl)bis(4-phenylbutan-1-one) (K29). Prepared according to the ***General Procedure for the Synthesis of Ketones from Tertiary Amides*** using N^2, N^2, N^6, N^6-tetramethylpyridine-2,6-dicarboxamide (55.3 mg, 0.25 mmol), $PhCH_2CH_2CH(Bpin)_2$ (279.0 mg, 0.75 mmol), MeLi (0.47 mL, 0.75 mmol, 1.6 M in Et_2O) and anisole (2.0 mL), 0 °C, 5 min then 120 °C, 4 h. Upon completion, the reaction mixture was quenched with H_2O and purified by column chromatography (PE : EA = 10 : 1) to give 41.3 mg (48% yield) of product as a colorless oil. **^{1}H NMR** (400 MHz, $CDCl_3$) δ 8.17 (t, *J* = 7.6 Hz, 2H), 7.98 – 7.93 (m, 1H), 7.31 – 7.26 (m, 4H), 7.23 – 7.18

(m, 6H), 3.24 (t, *J* = 7.2 Hz, 4H), 2.77 – 2.71 (m, 4H), 2.14 – 2.05 (m, 4H) ppm; ^{13}**C NMR** (101 MHz, $CDCl_3$) δ 201.1, 152.3, 141.7, 138.0, 128.5, 128.3, 125.9, 124.7, 36.9, 35.4, 25.7 ppm; **HRMS** (ESI) calcd for $C_{25}H_{25}NO_2$ $[M+H]^+$: 372.1964; found: 372.1959.

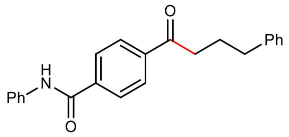

N*-phenyl-4-(4-phenylbutanoyl)benzamide (K30).** Prepared according to the ***General Procedure for the Synthesis of Ketones from Tertiary Amides using N^1, N^1-dimethyl-N^4-phenylterephthalamide (67.0 mg, 0.25 mmol), $PhCH_2CH_2CH(Bpin)_2$ (139.8 mg, 0.375 mmol), MeLi (0.39 mL, 0.625 mmol, 1.6 M in Et_2O) and anisole (2.0 mL), 0 °C, 5 min then 120 °C, 2 h. Upon completion, the reaction mixture was quenched with H_2O and purified by column chromatography (PE : EA = 3 : 1) to give 34.3 mg (40% yield) of product as a colorless oil. ^{1}**H NMR** (400 MHz, DMSO-*d6*) δ 10.40 (s, 1H), 8.08 – 8.04 (m, 4H), 7.82 – 7.76 (m, 2H), 7.40 – 7.33 (m, 2H), 7.32 – 7.26 (m, 2H), 7.25 – 7.16 (m, 3H), 7.15 – 7.09 (m, 1H), 3.10 (t, *J* = 7.2 Hz, 2H), 2.66 (t, *J* = 7.6 Hz, 2H), 2.00 – 1.88 (m, 2H) ppm; ^{13}**C NMR** (101 MHz, DMSO-*d6*) δ 199.6, 164.8, 141.7, 138.9, 138.7(3), 138.6(6), 128.6, 128.3, 128.0, 127.8, 125.8, 123.9, 120.4, 37.7, 34.4, 25.5 ppm; **HRMS** (ESI) calcd for $C_{23}H_{21}NO_2$ $[M+H]^+$: 344.1651; found: 344.1633.

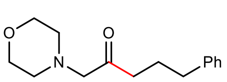

1-Morpholino-5-phenylpentan-2-one (K31). Prepared according to the ***General Procedure for the Synthesis of Ketones from Tertiary Amides*** using *N*, *N*-diethyl-2-morpholinoacetamide (50.0 mg, 0.25 mmol), $PhCH_2CH_2CH(Bpin)_2$ (139.8 mg, 0.375 mmol), MeLi (0.24 mL, 0.375 mmol, 1.6 M in Et_2O) and anisole (2.0 mL), 0 °C, 5 min then 120 °C, 2 h. Upon completion, the reaction mixture was quenched with H_2O and purified by column chromatography (DCM : MeOH = 20 : 1) to give 21.5 mg (35% yield) of product as yellow oil. ^{1}**H NMR** (400 MHz, $CDCl_3$) δ 7.30 – 7.24 (m, 2H), 7.23 – 7.11 (m, 3H), 3.72 (t, *J* = 4.4 Hz, 4H), 3.16 (s, 2H), 2.63 (t, *J* = 7.6 Hz, 2H), 2.54

– 2.37 (m, 6H), 1.98 – 1.87 (m, 2H) ppm; **^{13}C NMR** (101 MHz, $CDCl_3$) δ 207.9, 141.4, 128.4(2), 128.3(6), 126.0, 67.8, 66.7, 53.7, 39.5, 35.0, 25.1 ppm. **HRMS** (ESI) calcd for $C_{15}H_{21}NO_2$ $[M+Na]^+$: 270.1470; found: 270.1465.

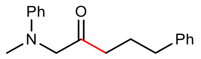

1-(Methyl(phenyl)amino)-5-phenylpentan-2-one (K32). Prepared according to the ***General Procedure for the Synthesis of Ketones from Tertiary Amides*** using *N*, *N*-dimethyl-2-(methyl(phenyl)amino)acetamide (48.0 mg, 0.25 mmol), $PhCH_2CH_2CH(Bpin)_2$ (139.8 mg, 0.375 mmol), MeLi (0.24 mL, 0.375 mmol, 1.6 M in Et_2O) and anisole (2.0 mL), 0 °C, 5 min then 120 °C, 2 h. Upon completion, the reaction mixture was quenched with H_2O and purified by column chromatography (PE : EA = 5 : 1) to give 24.0 mg (36% yield) of product as yellow oil. **^{1}H NMR** (400 MHz, $CDCl_3$) δ 7.27 – 7.11 (m, 7H), 6.74 (t, *J* = 7.2 Hz, 1H), 6.59 (d, *J* = 8.0 Hz, 2H), 3.98 (s, 2H), 3.02 (s, 3H), 2.59 (t, *J* = 7.6 Hz, 2H), 2.43 (t, *J* = 7.2 Hz, 2H), 1.95 – 1.86 (m, 2H) ppm; **^{13}C NMR** (101 MHz, $CDCl_3$) δ 210.2, 148.7, 141.4, 129.3, 128.4(4), 128.3(6), 125.9, 117.2, 111.9, 62.7, 39.7, 38.6, 35.0, 24.7 ppm. **HRMS** (ESI) calcd for $C_{18}H_{21}NO$ $[M+H]^+$: 268.1701; found: 268.1965.

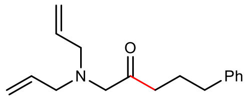

1-(diallylamino)-5-phenylpentan-2-one (K33). Prepared according to the ***General Procedure for the Synthesis of Ketones from Tertiary Amides*** using 2-(diethylamino)-*N*, *N*-dimethylacetamide (39.5 mg, 0.25 mmol), $PhCH_2CH_2CH(Bpin)_2$ (139.8 mg, 0.375 mmol), MeLi (0.24 mL, 0.375 mmol, 1.6 M in Et_2O) and anisole (2.0 mL), 0 °C, 5 min then 120 °C, 2 h. Upon completion, the reaction mixture was quenched with H_2O and purified by column chromatography (DCM : MeOH = 20 : 1) to give 27.7 mg (43% yield) of product as yellow oil. **^{1}H NMR** (400 MHz, $CDCl_3$) δ 7.29 – 7.25 (m, 2H), 7.22 – 7.14 (m, 3H), 5.92 – 5.73 (m, 2H), 5.22 – 5.08 (m, 4H), 3.22 (s, 2H), 3.12 (d, *J* = 6.4 Hz, 4H), 2.61 (t, *J* = 7.6 Hz, 2H), 2.43 (t, *J* = 7.2 Hz, 2H), 1.95 – 1.84 (m, 2H)

ppm; **^{13}C NMR** (101 MHz, $CDCl_3$) δ 210.2, 141.6, 135.1, 128.4, 128.3, 125.9, 118.3, 62.4, 57.6, 39.4, 35.1, 25.1 ppm; **HRMS** (ESI) calcd for $C_{17}H_{23}NO$ $[M+H]^+$: 258.1858; found: 258.1854.

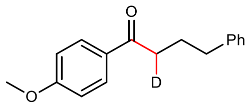

1-(4-Methoxyphenyl)-4-phenylbutan-1-one-2-*d* (K34). Prepared according to the ***General Procedure for the Synthesis of Ketones from Tertiary Amides*** using 4-methoxy-*N*, *N*-dimethylbenzamide (44.8 mg, 0.25 mmol), $PhCH_2CH_2CH(Bpin)_2$ (139.8 mg, 0.375 mmol), MeLi (0.24 mL, 0.375 mmol) and anisle (2.0 mL), 0 ºC, 5 min then 120 ºC, 2 h. Upon completion, the reaction mixture was quenched with D_2O (100 μL, 5.0 mmol) and purified by column chromatography (PE : EA = 10 : 1) to give 44.9 mg (70% yield) of product as a white solid, mp: 53-56 ºC. **^{1}H NMR** (400 MHz, $CDCl_3$) δ 7.90 (d, J = 9.2 Hz, 2H), 7.32 – 7.25 (m, 2H), 7.23 – 7.16 (m, 3H), 6.91 (d, J = 9.2 Hz, 2H), 3.85 (s, 3H), 2.93 – 2.85 (m, 1H), 2.71 (t, J = 7.6 Hz, 2H), 2.11 – 2.00 (m, 2H) ppm; **^{13}C NMR** (101 MHz, $CDCl_3$) δ 198.7, 163.3, 141.7, 130.2, 128.5, 128.3, 125.9, 113.6, 55.4, 37.0 (t, J = 191.9 Hz), 35.2, 25.9 ppm. **HRMS** (ESI) calcd for $C_{17}H_{17}DO_2$ $[M+H]^+$: 256.1448; found: 256.1441.

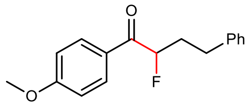

2-Fluoro-1-(4-methoxyphenyl)-4-phenylbutan-1-one (K35). Prepared according to the ***General Procedure for the Synthesis of Ketones from Tertiary Amides*** using 4-methoxy-*N*, *N*-dimethylbenzamide (44.8 mg, 0.25 mmol), $PhCH_2CH_2CH(Bpin)_2$ (139.8 mg, 0.375 mmol), MeLi (0.24 mL, 0.375 mmol) and anisole (2.0 mL), 0 ºC, 5 min then 120 ºC. After 2 h, NFSI (158.0 mg, 0.50 mmol) was added under N_2 atmosphere, then stirred at room temperature for another 12 h. The title compound was purified by column chromatography (PE : EA = 10 : 1) to give 42.3 mg (62% yield) of product as a colorless oil. **^{1}H NMR** (400 MHz, $CDCl_3$) δ 7.90 – 7.84 (m, 2H), 7.35 – 7.28 (m, 2H), 7.24 – 7.18 (m, 3H), 6.95 – 6.87 (m, 2H), 5.60 – 5.40 (m, 1H), 3.86 (s,

3H), 2.96 – 2.77 (m, 2H), 2.35 – 2.16 (m, 2H) ppm; **^{13}C NMR** (101 MHz, $CDCl_3$) δ 194.8 (d, *J* = 191.9 Hz), 164.0, 140.3, 131.2(1), 131.1(7), 128.5, 127.1, 126.3, 113.9, 92.5 (d, *J* = 183.8 Hz), 55.5, 34.4 (d, *J* = 21.2 Hz), 30.8 (d, *J* = 3.0 Hz) ppm. **HRMS** (ESI) calcd for $C_{17}H_{17}FO_2$ $[M+H]^+$: 273.1291; found: 273.1284.

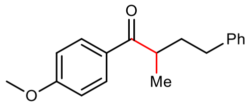

1-(4-Methoxyphenyl)-2-methyl-4-phenylbutan-1-one (K36). Prepared according to the ***General Procedure for the Synthesis of Ketones from Tertiary Amides*** using 4-methoxy-*N*, *N*-dimethylbenzamide (44.8 mg, 0.25 mmol), $PhCH_2CH_2CH(Bpin)_2$ (139.8 mg, 0.375 mmol), MeLi (0.24 mL, 0.375 mmol) and anisole (2 mL), 0 °C, 5 min then 120 °C. After 2 h, MeI (71.0 mg, 0.50 mmol) was added under N_2 atmosphere, then stirred at room temperature for another 12 h. The title compound was purified by column chromatography (PE : EA = 10 : 1) to give 40.0 mg (60% yield) of product as a colorless oil. **^{1}H NMR** (400 MHz, $CDCl_3$) δ 7.86 (d, *J* = 8.4 Hz, 2H), 7.32 – 7.23 (m, 2H), 7.21 – 7.10 (m, 3H), 6.91 (d, *J* = 8.8 Hz, 2H), 3.86 (s, 3H), 3.49 – 3.36 (m, 1H), 2.69 – 2.57 (m, 2H), 2.22 – 2.08 (m, 1H), 1.82 – 1.67 (m, 1H), 1.22 (d, *J* = 6.8 Hz, 3H) ppm; **^{13}C NMR** (101 MHz, $CDCl_3$) δ 202.6, 163.3, 141.8, 130.5, 129.4, 128.5, 128.3, 125.8, 113.7, 55.4, 39.2, 35.3, 33.5, 17.5 ppm. **HRMS** (ESI) calcd for $C_{18}H_{20}O_2$ $[M+H]^+$: 269.1542; found: 269.1549.

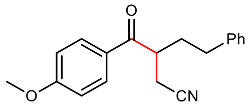

3-(4-Methoxybenzoyl)-5-phenylpentanenitrile (K37). Prepared according to the ***General Procedure for the Synthesis of Ketones from Tertiary Amides*** using 4-methoxy-*N*, *N*-dimethylbenzamide (44.8 mg, 0.25 mmol), $PhCH_2CH_2CH(Bpin)_2$ (139.8 mg, 0.375 mmol), MeLi (0.24 mL, 0.375 mmol) and anisole (2.0 mL), 0 °C, 5 min then 120 °C. After 2 h, 2-bromoacetonitrile (60.0 mg, 0.50 mmol) was added under N_2 atmosphere, then heated at 100 °C for another 6 h. The title compound was purified by column chromatography (PE : EA = 10 : 1) to give the yellow oil, 42.5 mg, 58%

yield. ^{1}**H NMR** (400 MHz, $CDCl_3$) δ 7.80 (d, *J* = 8.4 Hz, 2H), 7.32 – 7.25 (m, 2H), 7.25 – 7.19 (m, 1H), 7.16 – 7.07 (m, 2H), 6.97 – 6.86 (m, 2H), 3.87 (s, 3H), 3.78 – 3.64 (m, 1H), 2.80 – 2.59 (m, 4H), 2.26 – 2.12 (m, 1H), 2.07 – 1.93 (m, 1H) ppm; ^{13}**C NMR** (101 MHz, $CDCl_3$) δ 198.0, 164.0, 140.1, 130.8, 128.6, 128.4, 128.2, 126.4, 118.5, 114.0, 55.5, 41.3, 33.8, 32.4, 18.7 ppm. **HRMS** (ESI) calcd for $C_{19}H_{19}NO_2$ $[M+H]^+$: 294.1494; found: 294.1485.

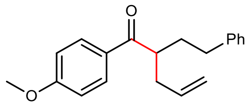

1-(4-Methoxyphenyl)-2-phenethylpent-4-en-1-one (K38). Prepared according to the ***General Procedure for the Synthesis of Ketones from Tertiary Amides*** using 4-methoxy-*N*, *N*-dimethylbenzamide (44.8 mg, 0.25 mmol), $PhCH_2CH_2CH(Bpin)_2$ (139.8 mg, 0.375 mmol), MeLi (0.24 mL, 0.375 mmol) and anisole (2.0 mL), 0 °C, 5 min then 120 °C. After 2 h, 3-bromoprop-1-ene (60.0 mg, 0.50 mmol) was added under N_2 atmosphere, then heated at 100 °C for another 6 h. The title compound was purified by column chromatography (PE : EA = 10 : 1) to give 47.7 mg (65% yield) of product as a colorless oil. ^{1}**H NMR** (400 MHz, $CDCl_3$) δ 7.87 (d, *J* = 8.4 Hz, 2H), 7.33 – 7.06 (m, 5H), 6.92 (d, *J* = 8.8 Hz, 2H), 5.82 – 5.65 (m, 1H), 5.13 – 4.88 (m, 2H), 3.87 (s, 3H), 3.55 – 3.36 (m, 1H), 2.72 – 2.44 (m, 3H), 2.35 – 2.24 (m, 1H), 2.22 – 2.07 (m, 1H), 1.93 – 1.76 (m, 1H) ppm; ^{13}**C NMR** (101 MHz, $CDCl_3$) δ 201.7, 163.4, 141.7, 135.7, 130.5, 130.1, 128.4, 128.3, 125.9, 116.7, 113.7, 55.4, 44.5, 36.6, 33.5, 33.4 ppm. **HRMS** (ESI) calcd for $C_{20}H_{22}O_2$ $[M+H]^+$: 295.1698; found: 295.1694.

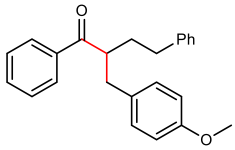

2-(4-Methoxybenzyl)-1,4-diphenylbutan-1-one (K39).[15] Prepared according to the ***General Procedure for the Synthesis of Ketones from Tertiary Amides*** using *N*, *N*-dimethylbenzamide (37.3 mg, 0.25 mmol), $PhCH_2CH_2CH(Bpin)_2$ (139.8 mg, 0.375 mmol), MeLi (0.24 mL, 0.375 mmol) and anisole (2.0 mL), 0 °C, 5 min then 120 °C. After 2 h, 1-(chloromethyl)-4-methoxybenzene (78.3 mg, 0.50 mmol) was added under

N_2 atmosphere, then heated at 100 °C for another 6 h. The title compound was purified by column chromatography (PE : EA = 10 : 1) to give 51.0 mg (55% yield) of product as a colorless oil. **^{1}H NMR** (400 MHz, $CDCl_3$) δ 7.78 (d, J = 7.6 Hz, 2H), 7.55 – 7.45 (m, 1H), 7.44 – 7.33 (m, 2H), 7.26 – 7.21 (m, 2H), 7.19 – 7.14 (m, 1H), 7.11 – 6.99 (m, 4H), 6.75 (d, J = 8.0 Hz, 2H), 3.77 – 3.64 (m, 4H), 3.13 – 3.01 (m, 1H), 2.82 – 2.71 (m, 1H), 2.68 – 2.58 (m, 1H), 2.57 – 2.47 (m, 1H), 2.20 – 2.06 (m, 1H), 1.91 – 1.78 (m, 1H) ppm; **^{13}C NMR** (101 MHz, $CDCl_3$) δ 203.6, 157.9, 141.5, 137.2, 132.9, 131.6, 129.9, 128.5, 128.4, 128.3, 128.2, 125.9, 113.7, 55.2, 47.6, 37.3, 33.5, 33.4 ppm.

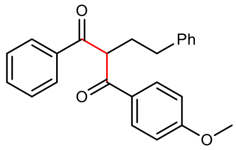

1-(4-Methoxyphenyl)-2-phenethyl-3-phenylpropane-1,3-dione (K40).[15] Prepared according to the ***General Procedure for the Synthesis of Ketones from Tertiary Amides*** using *N*, *N*-dimethylbenzamide (37.3 mg, 0.25 mmol), $PhCH_2CH_2CH(Bpin)_2$ (139.8 mg, 0.375 mmol), MeLi (0.24 mL, 0.375 mmol) and anisole (2.0 mL), 0 °C, 5 min then 120 °C. After 2 h, 4-methoxybenzoyl chloride (85.3 mg, 0.50 mmol) was added under N_2 atmosphere, then heated at 100 °C for another 6 h. The title compound was purified by column chromatography (PE : EA = 10 : 1) to give 47.4 mg (49% yield) of product as a colorless oil. **^{1}H NMR** (400 MHz, $CDCl_3$) δ 7.88 – 7.76 (m, 4H), 7.56 – 7.47 (m, 1H), 7.43 – 7.35 (m, 2H), 7.34 – 7.28 (m, 2H), 7.27 – 7.21 (m, 1H), 7.21 – 7.14 (m, 2H), 6.87 (d, J = 8.4 Hz, 2H), 5.12 (t, J = 6.4 Hz, 1H), 3.83 (s, 3H), 2.81 – 2.71 (m, 2H), 2.51 – 2.33 (m, 2H) ppm; **^{13}C NMR** (101 MHz, $CDCl_3$) δ 196.1, 194.5, 163.7, 141.0, 135.9, 133.4, 130.9, 128.8, 128.7, 128.5, 128.4, 126.3, 114.0, 55.5, 55.2, 34.0, 30.8 ppm.

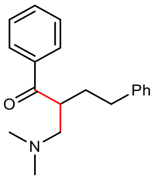

2-((Dimethylamino)methyl)-1,4-diphenylbutan-1-one (K41).[15] Prepared according

to the ***General Procedure for the Synthesis of Ketones from Tertiary Amides*** using *N*, *N*-dimethylbenzamide (37.3 mg, 0.25 mmol), $PhCH_2CH_2CH(Bpin)_2$ (139.8 mg, 0.375 mmol), MeLi (0.24 mL, 0.375 mmol) and anisole (2.0 mL), 0 °C, 5 min then 120 °C. After 2 h, paraformaldehyde (7.5 mg, 0.25 mmol) was added under N_2 atmosphere, then stirred at 50 °C for another 5 h. The title compound was purified by column chromatography (DCM : MeOH = 20 : 1) to give 31.5 mg (45% yield) of product as yellow oil. **^{1}H NMR** (400 MHz, $CDCl_3$) δ 7.88 (d, *J* = 7.6 Hz, 2H), 7.59 – 7.53 (m, 1H), 7.48 – 7.39 (m, 2H), 7.28 – 7.22 (m, 2H), 7.21 – 7.15 (m, 1H), 7.15 – 7.09 (m, 2H), 3.72 – 3.62 (m, 1H), 2.87 – 2.77 (m, 1H), 2.68 – 2.52 (m, 2H), 2.45 – 2.36 (m, 1H), 2.19 (s, 6H), 2.11 – 2.01 (m, 1H), 1.94 – 1.84 (m, 1H) ppm; **^{13}C NMR** (101 MHz, $CDCl_3$) δ 203.3, 141.5, 137.3, 133.0, 128.6, 128.4, 128.3, 128.2, 125.9, 61.7, 45.9, 43.7, 33.4, 32.7 ppm.

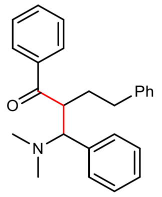

2-((Dimethylamino)(phenyl)methyl)-1,4-diphenylbutan-1-one (K42). Prepared according to the ***General Procedure for the Synthesis of Ketones from Tertiary Amides*** using *N*, *N*-dimethylbenzamide (37.3 mg, 0.25 mmol), $PhCH_2CH_2CH(Bpin)_2$ (139.8 mg, 0.375 mmol), MeLi (0.24 mL, 0.375 mmol) and anisole (2.0 mL), 0 °C, 5 min then 120 °C. After 2 h, benzaldehyde (53.1 mg, 0.50 mmol) was added under N_2 atmosphere, then heated at 40 °C for another 12 h. The title compound was purified by column chromatography (PE : EA = 3 : 1) to give 57.2 mg (64% yield) of product as a colorless oil. *This compound was a signal isomer. It is worth noting that the product was unstable when exposed to air atmosphere.* **^{1}H NMR** (400 MHz, $CDCl_3$) δ 8.04 – 7.93 (m, 2H), 7.60 – 7.54 (m, 1H), 7.52 – 7.45 (m, 2H), 7.37 – 7.28 (m, 3H), 7.20 – 7.11 (m, 5H), 6. 95 – 6.88 (m, 2H), 4.25 – 4.14 (m, 1H), 3.98 (d, *J* = 10.8 Hz, 1H), 2.57 – 2.47 (m, 1H), 2.40 – 2.29 (m, 1H), 2.04 – 1.89 (m, 7H), 1.68 – 1.58 (m, 1H) ppm; **^{13}C NMR** (101 MHz, $CDCl_3$) δ 204.3, 141.4, 139.0, 134.2, 132.6, 129.4, 128.5, 128.4, 128.2, 128.1, 127.8, 127.4, 125.8, 72.6, 46.3, 41.6, 33.3, 31.6 ppm. **HRMS** (ESI) calcd for $C_{25}H_{27}NO$

[M+H]$^+$: 358.2171; found: 358.2164.

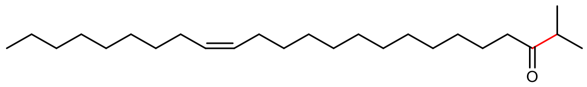

(Z)-2-methyltetracos-15-en-3-one (K43). Prepared according to the ***General Procedure for the Synthesis of Ketones from Primary Amides*** using erucylamide (84.4 mg, 0.25 mmol), 2,2'-(propane-2,2-diyl)bis(4,4,5,5-tetramethyl-1,3,2-dioxaborolane) (148.0 mg, 0.50 mmol), ^{n}BuLi (0.40 mL, 1.0 mmol, 2.5 M in hexane) and THF (2.0 mL), 0 °C, 5 min then 100 °C, 24 h. Upon completion, the reaction mixture was quenched with H_2O and purified by column chromatography (PE : EA = 50 : 1) to give 36.4 mg (40% yield) of product as a colorless oil. **^{1}H NMR** (400 MHz, $CDCl_3$) δ 5.39 – 5.30 (m, 2H), 2.65 – 2.53 (m, 1H), 2.43 (t, J = 7.2 Hz, 2H), 2.05 – 1.98 (m, 4H), 1.10 – 1.05 (m, 30H), 1.08 (d, J = 6.8 Hz, 6H), 0.91 – 0.85 (m, 3H) ppm; **^{13}C NMR** (101 MHz, $CDCl_3$) δ 215.0, 129.8(9), 129.8(7), 40.8, 40.4, 31.9, 29.8, 29.7, 29.6(4), 29.6(0), 29.5(4), 29.5(1), 29.4(9), 29.4(5), 29.3(4), 29.3(1), 27.2, 23.8, 22.7, 18.3, 14.1 ppm. **HRMS** (ESI) calcd for $C_{25}H_{48}O$ [M+H]$^+$: 365.3783; found: 365.3779.

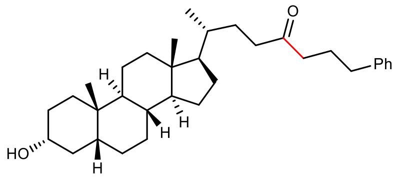

(*R*)-7-((3*R*,5*R*,8*R*,9*S*,10*S*,13*R*,14*S*,17*R*)-3-hydroxy-10,13-dimethylhexadecahydro-1*H*-cyclopenta[a]phenanthren-17-yl)-1-phenyloctan-4-one (K44). Prepared according to the ***General Procedure for the Synthesis of Ketones from Tertiary Amides*** using *(R)-4-((3R,5R,8R,9S,10S,13R,14S,17R)*-3-hydroxy-10,13-dimethylhexadecahydro-1*H*-cyclopenta[a]phenanthren-17-yl)-*N*, *N*-dimethylpentanamide (100.8 mg, 0.25 mmol), $PhCH_2CH_2CH(Bpin)_2$ (139.8 mg, 0.375 mmol), MeLi (0.39 mL, 0.625 mmol, 1.6 M in Et_2O) and anisole (2.0 mL), 0 °C, 5 min then 120 °C, 2 h. Upon completion, the reaction mixture was quenched with H_2O and purified by column chromatography (PE : EA = 5 : 1) to give 62.5 mg (53% yield) of product as light yellow oil. **^{1}H NMR** (400 MHz, $CDCl_3$) 7.30 – 7.26 (m, 2H), 7.20 –

7.16 (m, 3H), 3.67 – 3.58 (m, 1H), 2.61 (t, *J* = 7.6 Hz, 2H), 2.43 – 2.23 (m, 4H), 1.96 – 1.64 (m, 11H), 1.58 – 1.49 (m, 2H), 1.43 – 1.04 (m, 16H), 0.91 – 0.87 (m, 6H), 0.63 (s, 3H) ppm; **^{13}C NMR** (101 MHz, $CDCl_3$) δ 211.5, 141.6, 128.4, 128.3, 125.9, 71.8, 56.5, 56.0, 42.7, 42.1, 41.8, 40.4, 40.1, 39.7, 36.4, 35.8, 35.3, 35.3, 35.1, 34.5, 30.5, 29.8, 28.2, 27.2, 26.4, 25.2, 24.2, 23.3, 20.8, 18.4, 12.0 ppm. **HRMS** (ESI) calcd for $C_{33}H_{50}O_2$ $[M+Na]^+$: 501.3709; found: 501.3706.

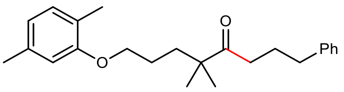

8-(2,5-dimethylphenoxy)-5,5-dimethyl-1-phenyloctan-4-one (K45). Prepared according to the ***General Procedure for the Synthesis of Ketones from Tertiary Amides*** using 5-(2,5-dimethylphenoxy)-*N*, *N*, 2, 2-tetramethylpentanamide (69.3 mg, 0.25 mmol), $PhCH_2CH_2CH(Bpin)_2$ (139.8 mg, 0.375 mmol), MeLi (0.24 mL, 0.375 mmol, 1.6 M in Et_2O) and THF (2.0 mL), 0 °C, 5 min then 100 °C, 6 h. Upon completion, the reaction mixture was quenched with H_2O and purified by column chromatography (PE : EA = 30 : 1) to give 58.8 mg (67% yield) of product as a colorless oil. **^{1}H NMR** (400 MHz, $CDCl_3$) δ 7.28 – 7.24 (m, 2H), 7.18 – 7.16 (m, 3H), 7.00 (d, *J* = 8.4 Hz, 1H), 6.65 (d, *J* = 7.6 Hz, 1H), 6.59 (s, 1H), 3.88 (t, *J* = 5.6 Hz, 2H), 2.60 (t, *J* = 7.6 Hz, 2H), 2.50 (t, *J* = 7.2 Hz, 2H), 2.30 (s, 3H), 2.17 (s, 3H), 1.96 – 1.84 (m, 2H), 1.74 – 1.58 (m, 4H), 1.13 (s, 6H) ppm; **^{13}C NMR** (101 MHz, $CDCl_3$) δ 215.2, 156.8, 141.8, 136.5, 130.3, 128.4, 128.3, 125.8, 123.4, 120.7, 111.9, 67.8, 47.3, 36.3, 35.9, 35.2, 25.3, 25.0, 24.4, 21.4, 15.8 ppm; **HRMS** (ESI) calcd for $C_{24}H_{32}O_2$ $[M+Na]^+$: 375.2300; found: 375.2297.

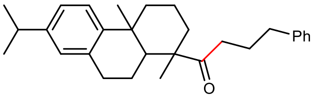

1-(7-Isopropyl-1,4a-dimethyl-1,2,3,4,4a,9,10,10a-octahydrophenanthren-1-yl)-4-phenylbutan-1-one (K46). Prepared according to the ***General Procedure for the Synthesis of Ketones from Tertiary Amides*** using 7-isopropyl-*N*, *N*,1,4a-tetramethyl-1,2,3,4,4a,9,10,10a-octahydrophenanthrene-1-carboxamide (81.8 mg, 0.25 mmol), $PhCH_2CH_2CH(Bpin)_2$ (139.8 mg, 0.375 mmol), MeLi (0.24 mL, 0.375 mmol, 1.6 M in

Et_2O) and THF (2.0 mL), 0 °C, 5 min then 100 °C, 6 h. Upon completion, the reaction mixture was quenched with H_2O and purified by column chromatography (PE : EA = 30 : 1) to give 63.4 mg (63% yield) of product as a colorless oil. **^{1}H NMR** (400 MHz, $CDCl_3$) δ 7.28 – 7.15 (m, 6H), 7.00 (d, *J* = 8.0 Hz, 1H), 6.88 (s, 1H), 2.86 – 2.79 (m, 3H), 2.60 – 2.41 (m, 4H), 2.32 (d, *J* = 12.4 Hz, 1H), 2.16 (d, *J* = 12.4 Hz, 1H), 1.93 – 1.68 (m, 5H), 1.56 – 1.43 (m, 3H), 1.23 – 1.21 (m, 13H) ppm; **^{13}C NMR** (101 MHz, $CDCl_3$) δ 215.7, 146.9, 145.8, 141.8, 134.6, 128.4, 128.3, 127.0, 125.8, 124.1, 123.9, 52.5, 43.7, 38.0, 36.9, 35.7, 35.6, 35.2, 33.4, 29.9, 25.8, 25.2, 24.0, 21.6, 18.5, 15.8 ppm; **HRMS** (ESI) calcd for $C_{29}H_{38}O$ $[M+Na]^+$: 425.2820; found: 425.2816.

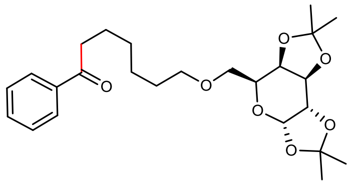

1-phenyl-7-(((3aS,5S,5aR,8aR,8bS)-2,2,7,7-tetramethyltetrahydro-5H-bis([1,3]dioxolo)[4,5-b:4',5'-d]pyran-5-yl)methoxy)heptan-1-one (K47). Prepared according to the ***General Procedure for the Synthesis of Ketones from Secondary Amides*** using *N*-methylbenzamide (33.8 mg, 0.25 mmol), 2,2'-(6-(((3aS,5S,5aR,8aR,8bS)-2,2,7,7-tetramethyltetrahydro-5H-bis([1,3]dioxolo)[4,5-b:4',5'-d]pyran-5-yl)methoxy)hexane-1,1-diyl)bis(4,4,5,5-tetramethyl-1,3,2-dioxaborolane) (298.0 mg, 0.50 mmol), $Al(OEt)_3$ (60.8 mg, 0.375 mmol), *ⁿ*BuLi (0.30 mL, 0.75 mmol, 2.5 M in hexane) and THF (2.0 mL), 0 °C, 5 min then 100 °C, 4 h. Upon completion, the reaction mixture was quenched with H_2O and purified by column chromatography (PE : EA = 5 : 1) to give 53.8 mg (48% yield) of product as a colorless oil. **^{1}H NMR** (400 MHz, $CDCl_3$) δ 7.96 (d, *J* = 8.0 Hz, 2H), 7.64 – 7.39 (m, 3H), 5.54 (d, *J* = 4.8 Hz, 1H), 4.60 (d, *J* = 6.8 Hz, 1H), 4.41 – 4.20 (m, 2H), 3.97 (t, *J* = 6.4 Hz, 1H), 3.71 – 3.39 (m, 4H), 2.96 (t, *J* = 7.2 Hz, 2H), 1.80 – 1.68 (m, 2H), 1.64 – 1.51 (m, 5H), 1.48 – 1.30 (m, 13H) ppm; **^{13}C NMR** (101 MHz, $CDCl_3$) δ 200.3, 136.9, 132.8, 128.4, 127.9, 109.0, 108.4, 96.2, 71.3, 71.1, 70.4(9), 70.4(5), 69.2, 66.6, 38.4, 29.3, 29.0, 26.0, 25.9, 25.8, 24.8, 24.3, 24.2 ppm; **HRMS** (ESI) calcd for $C_{25}H_{36}O_7$ $[M+Na]^+$:

471.2359; found: 471.2354.

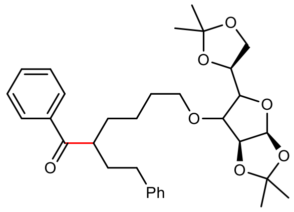

6-(((3aS,6aS)-5-((S)-2,2-dimethyl-1,3-dioxolan-4-yl)-2,2-dimethyltetrahydrofuro[2,3-*d*][1,3]dioxol-6-yl)oxy)-2-phenethyl-1-phenylhexan-1-one (K48). Prepared according to the ***General Procedure for the Synthesis of Ketones from Tertiary Amides*** using *N, N*-dimethylbenzamide (37.3 mg, 0.25 mmol), 2,2'-(7-(((3a*S*,6a*S*)-5-((*S*)-2,2-dimethyl-1,3-dioxolan-4-yl)-2,2-dimethyltetrahydrofuro[2,3-d][1,3]dioxol-6-yl)oxy)-1-phenylheptane-3,3-diyl)bis(4,4,5,5-tetramethyl-1,3,2-dioxaborolane) (257.3 mg, 0.375 mmol), MeLi (0.39 mL, 0.625 mmol, 1.6 M in Et_2O) and anisole (2.0 mL), 0 ºC, 5 min then 120 ºC, 2 h. Upon completion, the reaction mixture was quenched with H_2O and purified by column chromatography (PE : EA = 5 : 1) to give 98.2 mg (73% yield) of product as a colorless oil. **^{1}H NMR** (400 MHz, $CDCl_3$) δ 7.88 – 7.83 (m, 2H), 7.59 – 7.51 (m, 1H), 7.44 (t, *J* = 7.6 Hz, 2H), 7.27 – 7.21 (m, 2H), 7.21 – 7.15 (m, 1H), 7.13 – 7.06 (m, 2H), 5.81 (t, *J* = 4.0 Hz, 1H), 4.46 (d, *J* = 3.6 Hz, 1H), 4.28 – 4.20 (m, 1H), 4.12 – 4.06 (m, 1H), 4.06 – 4.00 (m, 1H), 3.99 – 3.92 (m, 1H), 3.80 (d, *J* = 3.2 Hz, 1H), 3.55 – 3.39 (m, 3H), 2.19 – 2.07 (m, 2H), 2.19 – 2.07 (m, 1H), 1.87 – 1.76 (m, 2H), 1.57 – 1.47 (m, 6H), 1.41 – 1.38 (m, 3H), 1.34 – 1.27 (m, 8H) ppm; **^{13}C NMR** (101 MHz, $CDCl_3$) δ 203.8(4), 203.8, 141.6, 137.3(4), 137.3(2), 132.9, 128.6, 128.4, 128.3, 128.1, 125.9, 111.6, 108.8, 105.2, 82.5, 82.4, 82.1, 82.0, 81.1(0), 81.0(6), 72.4(4), 72.4(2), 70.3, 70.1, 67.1, 45.1(4), 45.1(1), 33.9, 33.8, 33.5, 32.0, 29.8(3), 29.7(8), 26.8, 26.7, 26.2, 25.3(3), 25.3(0), 24.0, 23.9 ppm; **HRMS** (ESI) calcd for $C_{32}H_{42}O_7$ $[M+Na]^+$: 561.2828; found: 561.2819.

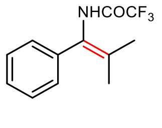

2,2,2-Trifluoro-*N*-(2-methyl-1-phenylprop-1-en-1-yl)acetamide (EA1). Prepared

according to the ***General Procedure for the Synthesis of Enamides from Primary Amides*** using benzamide (30.3 mg, 0.25 mmol), 2,2'-(propane-2,2-diyl)bis(4,4,5,5-tetramethyl-1,3,2-dioxaborolane) (148.0 mg, 0.50 mmol), ^{n}BuLi (0.40 mL, 1.0 mmol, 2.5 M in hexane) and THF (2 mL), 0 °C, 5 min then 100 °C, 24 h. Upon completion, ^{t}BuOH (0.50 mL) were added under N_2 atmosphere, then stirred at 100 °C for another 0.5 h. TFAA (1.0 mL) was then added under N_2 atmosphere, stirred at 100 °C for 2 h. The reaction mixture was quenched with saturated sodium bicarbonate solution and purified by column chromatography (PE : EA = 10 : 1) to give 41.3 mg (68% yield) of product as a white solid, mp: 114-116 °C. **^{1}H NMR** (400 MHz, $CDCl_3$) δ 7.37 – 7.24 (m, 6H), 1.83 – 1.82 (m, 6H) ppm; **^{13}C NMR** (101 MHz, $CDCl_3$) δ 154.9 (q, J = 36.3 Hz), 136.8, 132.7, 129.1, 128.4, 128.0, 125.2, 116.1 (q, J = 288.8 Hz), 21.2, 20.5 ppm; **^{19}F NMR** (376 MHz, $CDCl_3$) δ -75.48 ppm; **HRMS** (ESI) calcd for $C_{12}H_{12}F_3NO$ $[M+Na]^+$: 266.0769; found: 266.0766.

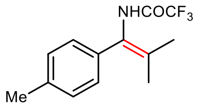

2,2,2-Trifluoro-N-(2-methyl-1-(p-tolyl)prop-1-en-1-yl)acetamide (EA2). Prepared according to the ***General Procedure for the Synthesis of Enamides from Primary Amides*** using 4-methylbenzamide (33.8 mg, 0.25 mmol), 2,2'-(propane-2,2-diyl)bis(4,4,5,5-tetramethyl-1,3,2-dioxaborolane) (148.0 mg, 0.50 mmol), ^{n}BuLi (0.40 mL, 1.0 mmol, 2.5 M in hexane) and THF (2 mL), 0 °C, 5 min then 100 °C, 24 h. Upon completion, ^{t}BuOH (0.50 mL) were added under N_2 atmosphere, then stirred at 100 °C for another 0.5 h. TFAA (1.0 mL) was then added under N_2 atmosphere, stirred at 100 °C for 2 h. The reaction mixture was quenched with saturated sodium bicarbonate solution and purified by column chromatography (PE : EA = 10 : 1) to give 46.0 mg (72% yield) of product as a white solid, mp: 104-106 °C. **^{1}H NMR** (400 MHz, $CDCl_3$) δ 7.31 (br, 1H), 7.17 – 7.12 (m, 4H), 2.35 (s, 3H), 1.82 (s, 3H), 1.80 (s, 3H) ppm; **^{13}C NMR** (101 MHz, $CDCl_3$) δ 154.9 (q, J = 36.5 Hz), 137.9, 133.8, 132.1, 129.0, 128.9, 125.1, 116.1 (q, J = 289.0 Hz), 21.2, 20.5 ppm; **^{19}F NMR** (376 MHz, $CDCl_3$) δ -75.50 ppm; **HRMS** (ESI) calcd for $C_{13}H_{14}F_3NO$ $[M+H]^+$: 258.1106; found: 258.1100.

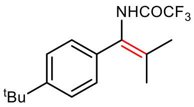

N*-(1-(4-(tert-butyl)phenyl)-2-methylprop-1-en-1-yl)-2,2,2-trifluoroacetamide (EA3).** Prepared according to the ***General Procedure for the Synthesis of Enamides from Primary Amides using 4-(*tert*-butyl)benzamide (44.3 mg, 0.25 mmol), 2,2'-(propane-2,2-diyl)bis(4,4,5,5-tetramethyl-1,3,2-dioxaborolane) (148.0 mg, 0.50 mmol), ^{n}BuLi (0.40 mL, 1.0 mmol, 2.5 M in hexane) and THF (2 mL), 0 °C, 5 min then 100 °C, 24 h. Upon completion, ^{t}BuOH (0.50 mL) were added under N_2 atmosphere, then stirred at 100 °C for another 0.5 h. TFAA (1.0 mL) was then added under N_2 atmosphere, stirred at 100 °C for 2 h. The reaction mixture was quenched with saturated sodium bicarbonate solution and purified by column chromatography (PE : EA = 10 : 1) to give 50.9 mg (68% yield) of product as a white solid, mp: 114-116 °C. **^{1}H NMR** (400 MHz, $CDCl_3$) δ 7.38 – 7.36 (m, 2H), 7.28 (br, 1H), 7.19 – 7.17 (m, 2H), 1.84 (s, 3H), 1.80 (s, 3H), 1.32 (s, 9H) ppm; **^{13}C NMR** (101 MHz, $CDCl_3$) δ 154.9 (q, J = 36.3 Hz), 151.0, 133.8, 132.3, 128.7, 125.2, 124.9, 116.1 (q, J = 288.9 Hz), 34.6, 31.3, 21.3, 20.6 ppm; **^{19}F NMR** (376 MHz, $CDCl_3$) δ -75.45 ppm; **HRMS** (ESI) calcd for $C_{16}H_{20}F_3NO$ $[M]^+$: 299.1497; found: 299.1502.

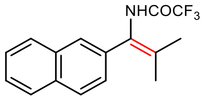

2,2,2-Trifluoro-N-(2-methyl-1-(naphthalen-2-yl)prop-1-en-1-yl)acetamide (EA4). Prepared according to the ***General Procedure for the Synthesis of Enamides from Primary Amides*** using 2-naphthamide (42.8 mg, 0.25 mmol), 2,2'-(propane-2,2-diyl)bis(4,4,5,5-tetramethyl-1,3,2-dioxaborolane) (148.0 mg, 0.50 mmol), ^{n}BuLi (0.40 mL, 1.0 mmol, 2.5 M in hexane) and THF (2 mL), 0 °C, 5 min then 100 °C, 24 h. Upon completion, ^{t}BuOH (0.50 mL) were added under N_2 atmosphere, then stirred at 100 °C for another 0.5 h. TFAA (1.0 mL) was then added under N_2 atmosphere, stirred at 100 °C for 2 h. The reaction mixture was quenched with saturated sodium bicarbonate solution and purified by column chromatography (PE : EA = 10 : 1) to give 42.9 mg

(59% yield) of product as a white solid, mp: 128-131 °C. **^{1}H NMR** (400 MHz, $CDCl_3$) δ 7.84 – 7.80 (m, 3H), 7.72 (m, 1H), 7.51 – 7.47 (m, 2H), 7.39 – 7.34 (m, 2H), 1.88 (s, 3H), 1.87 (s, 3H) ppm; **^{13}C NMR** (101 MHz, $CDCl_3$) δ 155.2, 154.9, 134.1, 133.2, 133.1, 132.8, 128.4, 128.1(2), 128.0(6), 127.7, 126.6, 126.5, 126.41, 125.2, 117.5, 114.7, 21.3, 20.6 ppm; **^{19}F NMR** (376 MHz, $CDCl_3$) δ -75.40 ppm; **HRMS** (ESI) calcd for $C_{16}H_{14}F_3NO$ $[M+Na]^+$: 316.0926; found: 316.0919.

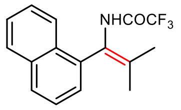

2,2,2-trifluoro-N-(2-methyl-1-(naphthalen-1-yl)prop-1-en-1-yl)acetamide (EA5). Prepared according to the ***General Procedure for the Synthesis of Enamides from Primary Amides*** using 1-naphthamide (42.8 mg, 0.25 mmol), 2,2'-(propane-2,2-diyl)bis(4,4,5,5-tetramethyl-1,3,2-dioxaborolane) (148.0 mg, 0.50 mmol), ^{n}BuLi (0.40 mL, 1.0 mmol, 2.5 M in hexane) and THF (2 mL), 0 °C, 5 min then 100 °C, 24 h. Upon completion, ^{t}BuOH (0.50 mL) were added under N_2 atmosphere, then stirred at 100 °C for another 0.5 h. TFAA (1.0 mL) was then added under N_2 atmosphere, stirred at 100 °C for 2 h. The reaction mixture was quenched with saturated sodium bicarbonate solution and purified by column chromatography (PE : EA = 10 : 1) to give 37.5 mg (51% yield) of product as a white solid, mp: 89 -91 °C. **^{1}H NMR** (400 MHz, $CDCl_3$) δ 7.89 – 7.84 (m, 3H), 7.52 – 7.39 (m, 4H), 7.30 (br, 1H), 1.92 (s, 3H), 1.60 (s, 3H) ppm; **^{13}C NMR** (101 MHz, $CDCl_3$) δ 154.5 (q, J = 36.6 Hz), 134.7, 134.4, 133.8, 131.6, 128.9, 128.5, 127.7, 126.8, 126.2, 125.4, 125.0, 117.4.1 (q, J = 289.9 Hz), 21.2, 20.2 ppm; **^{19}F NMR** (376 MHz, $CDCl_3$) δ -75.35 ppm; **HRMS** (ESI) calcd for $C_{16}H_{14}F_3NO$ $[M+Na]^+$: 316.0926; found: 316.0923.

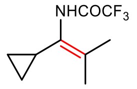

N*-(1-cyclopropyl-2-methylprop-1-en-1-yl)-2,2,2-trifluoroacetamide (EA6).** Prepared according to the ***General Procedure for the Synthesis of Enamides from Primary Amides using cyclopropanecarboxamide (21.3 mg, 0.25 mmol), 2,2'-(propane-

2,2-diyl)bis(4,4,5,5-tetramethyl-1,3,2-dioxaborolane) (148.0 mg, 0.50 mmol), ^{n}BuLi (0.40 mL, 1.0 mmol, 2.5 M in hexane) and THF (2 mL), 0 °C, 5 min then 100 °C, 24 h. Upon completion, ^{t}BuOH (0.50 mL) were added under N_2 atmosphere, then stirred at 100 °C for another 0.5 h. TFAA (1.0 mL) was then added under N_2 atmosphere, stirred at 100 °C for 2 h. The reaction mixture was quenched with saturated sodium bicarbonate solution and purified by column chromatography (PE : EA = 10 : 1) to give 20.7 mg (40% yield) of product as a white solid, mp: 58-61 °C. **^{1}H NMR** (400 MHz, $CDCl_3$) δ 6.85 (br, 1H), 1.90 (s, 3H), 1.74 – 1.66 (m, 1H), 1.63 (s, 3H), 0.77 – 0.70 (m, 2H), 0.45 – 0.39 (m, 2H) ppm; **^{13}C NMR** (101 MHz, $CDCl_3$) δ 155.3 (q, J = 36.0 Hz), 131.9, 124.5, 116.1 (q, J = 288.8 Hz), 20.2, 19.7, 12.1, 5.3 ppm; **^{19}F NMR** (376 MHz, $CDCl_3$) δ -75.42 ppm; **HRMS** (ESI) calcd for $C_9H_{12}F_3NO$ $[M+H]^+$: 208.0949; found: 208.0942.

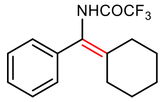

N*-(cyclohexylidene(phenyl)methyl)-2,2,2-trifluoroacetamide (EA7).** Prepared according to the ***General Procedure for the Synthesis of Enamides from Primary Amides using benzamide (30.3 mg, 0.25 mmol), 2,2'-(cyclohexylmethylene)bis(4,4,5,5-tetramethyl-1,3,2-dioxaborolane) (175.0 mg, 0.50 mmol), ^{n}BuLi (0.40 mL, 1.0 mmol, 2.5 M in hexane) and THF (2 mL), 0 °C, 5 min then 100 °C, 24 h. Upon completion, ^{t}BuOH (0.50 mL) were added under N_2 atmosphere, then stirred at 100 °C for another 0.5 h. TFAA (1.0 mL) was then added under N_2 atmosphere, stirred at 100 °C for 2 h. The reaction mixture was quenched with saturated sodium bicarbonate solution and purified by column chromatography (PE : EA = 10 : 1) to give 34.7 mg (49% yield) of product as a white solid, mp: 147-149 °C. **^{1}H NMR** (400 MHz, $CDCl_3$) δ 7.38 – 7.20 (m, 6H), 2.28 – 2.17 (m, 4H), 1.72 – 1.65 (m, 2H), 1.63 – 1.55 (m, 4H) ppm; **^{13}C NMR** (101 MHz, $CDCl_3$) δ 155.3 (q, J = 36.3 Hz), 140.0, 136.8, 129.1, 128.4, 128.0, 122.4, 116.1 (q, J = 288.9 Hz), 31.1, 30.7, 27.9, 27.1, 26.3 ppm; **^{19}F NMR** (376 MHz, $CDCl_3$) δ -75.47 ppm; **HRMS** (ESI) calcd for $C_{15}H_{16}F_3NO$ $[M+Na]^+$: 306.1082; found: 306.1075.

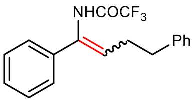

N*-(1,4-diphenylbut-1-en-1-yl)-2,2,2-trifluoroacetamide (*Z*:*E* = 67:33) (EA8).** Prepared according to the ***General Procedure for the Synthesis of Enamides from Primary Amides using benzamide (30.3 mg, 0.25 mmol), $PhCH_2CH_2CH(Bpin)_2$ (186.0 mg, 0.50 mmol), ^{n}BuLi (0.40 mL, 1.0 mmol, 2.5 M in hexane) and THF (2 mL), 0 °C, 5 min then 100 °C, 12 h. Upon completion, ^{t}BuOH (0.50 mL) were added under N_2 atmosphere, then stirred at 100 °C for another 0.5 h. TFAA (1.0 mL) was then added under N_2 atmosphere, stirred at 100 °C for 2 h. The reaction mixture was quenched with saturated sodium bicarbonate solution and purified by column chromatography (PE : EA = 10 : 1) to give 71.0 mg (89% yield) of product as a white solid, mp: 63-85 °C. **^{1}H NMR** (400 MHz, $CDCl_3$) δ 7.38 – 6.92 (m, 11H), 6.46 (t, *J* = 7.6 Hz, 0.33H), 5.95 (t, *J* = 7.6 Hz, 0.67H), 2.83 – 2.67 (m, 2H), 2.50 – 2.35 (m, 2H) ppm; **^{13}C NMR** (101 MHz, $CDCl_3$) δ 155.3 (q, *J* = 37.0 Hz), 141.1(3), 141.0(6), 136.1, 135.0, 132.1, 131.7, 128.8, 128.7, 128.6(2), 128.5(8), 128.5(5), 128.5(2), 128.4(8), 128.4, 128.3, 126.9, 126.3, 126.0, 125.4, 122.5, 115.8 (q, *J* = 289.9 Hz), 115.7 (q, *J* = 289.9 Hz), 35.9, 34.8, 30.2(3), 30.2(1) ppm. **^{19}F NMR** (376 MHz, $CDCl_3$) δ -75.28, -75.82 ppm; **HRMS** (ESI) calcd for $C_{18}H_{16}F_3NO$ $[M+Na]^+$: 342.1082; found: 342.1079.

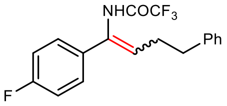

2,2,2-Trifluoro-*N*-(1-(4-fluorophenyl)-4-phenylbut-1-en-1-yl)acetamide (*Z*:*E* = 78:22) (EA9). Prepared according to the ***General Procedure for the Synthesis of Enamides from Primary Amides*** using 4-fluorobenzamide (34.8 mg, 0.25 mmol), $PhCH_2CH_2CH(Bpin)_2$ (186.0 mg, 0.50 mmol), ^{n}BuLi (0.40 mL, 1.0 mmol, 2.5 M in hexane) and THF (2 mL), 0 °C, 5 min then 100 °C, 12 h. Upon completion, ^{t}BuOH (0.50 mL) were added under N_2 atmosphere, then stirred at 100 °C for another 0.5 h. TFAA (1.0 mL) was then added under N_2 atmosphere, stirred at 100 °C for 2 h. The reaction mixture was quenched with saturated sodium bicarbonate solution and purified by column chromatography (PE : EA = 10 : 1) to give 59.0 mg (70% yield) of product as

a white solid, mp: 70-84 °C. **^{1}H NMR** (400 MHz, $CDCl_3$) δ 7.36 – 7.28 (m, 2H), 7.25 – 7.17 (m, 4H), 7.12 – 6.90 (m, 4H), 6.39 (t, *J* = 7.6 Hz, 0.22H), 5.86 (t, *J* = 7.6 Hz, 0.78H), 2.84 – 2.66 (m, 2H), 2.49 – 2.32 (m, 2H) ppm; **^{13}C NMR** (101 MHz, $CDCl_3$) δ 162.8 (d, *J* = 249.5 Hz), 162.7 (d, *J* = 249.5 Hz), 155.4 (q, *J* = 37.4 Hz), 141.0, 132.3(3), 132.3(0), 131.2, 131.1, 130.9(1), 130.8(8), 130.5, 130.4, 128.7, 128.6(4), 128.5(5), 128.5, 128.3(9), 128.3(6), 127.2, 127.1, 126.6, 126.4, 126.0, 123.1, 120.0, 117.2, 117.1, 115.9, 115.8, 115.7, 115.6, 115.5, 114.3, 111.4, 35.8, 34.8, 30.1(9), 30.1(5) ppm. **^{19}F NMR** (376 MHz, $CDCl_3$) δ -75.33, -75.84, -111.82, -113.15 ppm; **HRMS** (ESI) calcd for $C_{18}H_{15}F_4NO_2$ $[M+Na]^+$: 360.0987; found: 360.0987.

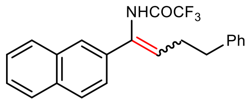

2,2,2-Trifluoro-*N*-(1-(naphthalen-2-yl)-4-phenylbut-1-en-1-yl)acetamide (*Z*:*E* = 76:24) (EA10). Prepared according to the ***General Procedure for the Synthesis of Enamides from Primary Amides*** using 2-naphthamide (42.8 mg, 0.25 mmol), $PhCH_2CH_2CH(Bpin)_2$ (186.0 mg, 0.50 mmol), ^{n}BuLi (0.40 mL, 1.0 mmol, 2.5 M in hexane) and THF (2 mL), 0 °C, 5 min then 100 °C, 12 h. Upon completion, ^{t}BuOH (0.50 mL) were added under N_2 atmosphere, then stirred at 100 °C for another 0.5 h. TFAA (1.0 mL) was then added under N_2 atmosphere, stirred at 100 °C for 2 h. The reaction mixture was quenched with saturated sodium bicarbonate solution and purified by column chromatography (PE : EA = 10 : 1) to give 64.6 mg (70% yield) of product as a white solid, mp: 100-110 °C. **^{1}H NMR** (400 MHz, $CDCl_3$) δ 7.84 – 7.61 (m, 4H), 7.53 – 7.42 (m, 2H), 7.39 – 6.93 (m, 7H), 6.53 (t, *J* = 7.6 Hz, 0.24H), 6.08 (t, *J* = 7.6 Hz, 0.76H), 2.81 (t, *J* = 7.2 Hz, 1.52H), 2.72 (t, *J* = 7.2 Hz, 0.48 Hz), 2.54 – 2.39 (m, 2H) ppm; **^{13}C NMR** (101 MHz, $CDCl_3$) δ 155.4 (q, *J* = 37.1 Hz), 141.1(2), 141.0(7), 133.4, 133.2(0), 133.1(7), 133.1, 133.0, 132.3, 132.1, 131.7, 128.6(2), 128.6, 128.5, 1284, 128.2(3), 128.1(6), 128.1, 127.7, 127.6, 127.5, 126.9, 126.7, 126.5, 126.4, 126.3, 126.0, 125.8, 123.2, 122.9, 120.2, 117.3, 114.4, 111.6, 35.9, 34.8, 30.3(8), 30.3(6) ppm. **^{19}F NMR** (376 MHz, $CDCl_3$) δ -75.17, -75.76 ppm; **HRMS** (ESI) calcd for $C_{22}H_{18}F_3NO_2$ $[M+Na]^+$: 392.1238; found: 392.1234.

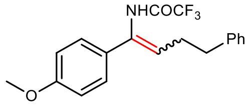

2,2,2-Trifluoro-*N*-(1-(4-methoxyphenyl)-4-phenylbut-1-en-1-yl)acetamide (*Z*:*E* = 80:20) (EA11). Prepared according to the ***General Procedure for the Synthesis of Enamides from Primary Amides*** using 4-methoxybenzamide (35.3 mg, 0.25 mmol), $PhCH_2CH_2CH(Bpin)_2$ (186.0 mg, 0.50 mmol), ^{n}BuLi (0.40 mL, 1.0 mmol, 2.5 M in hexane) and THF (2 mL), 0 °C, 5 min then 100 °C, 12 h. Upon completion, ^{t}BuOH (0.50 mL) were added under N_2 atmosphere, then stirred at 100 °C for another 0.5 h. TFAA (1.0 mL) was then added under N_2 atmosphere, stirred at 100 °C for 2 h. The reaction mixture was quenched with saturated sodium bicarbonate solution and purified by column chromatography (PE : EA = 10 : 1) to give 63.6 mg (73% yield) of product as a white solid, mp: 64-81 °C. **^{1}H NMR** (400 MHz, $CDCl_3$) δ 7.32 – 7.30 (m, 1H), 7.25 – 7.16 (m, 5H), 7.12 – 7.05 (m, 1H), 6.91 – 6.81 (m, 3H), 6.41 (t, *J* = 7.6 Hz, 0.2H), 5.84 (t, *J* = 7.6 Hz, 0.8H), 3.80 – 3.78 (m, 3H), 2.79 (t, *J* = 7.2 Hz, 1.6H), 2.72 (t, *J* = 7.2 Hz, 0.4H), 2.46 – 2.37 (m, 2H) ppm; **^{13}C NMR** (101 MHz, $CDCl_3$) δ 159.9, 155.3 (q, *J* = 36.7 Hz), 141.2(4), 141.1(7), 131.7, 131.3, 129.8, 128.7, 128.6, 128.5, 128.3, 127.3, 126.7, 126.2, 126.0, 125.1, 122.0, 117.3, 114.4, 114.1(2), 114.0(7), 55.3, 36.0, 34.9, 30.3, 30.2 ppm. **^{19}F NMR** (376 MHz, $CDCl_3$) δ -75.31, -75.86 ppm; **HRMS** (ESI) calcd for $C_{19}H_{18}F_3NO_2$ $[M+Na]^+$: 372.1187; found: 372.1183.

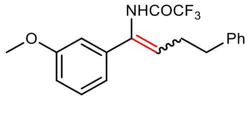

2,2,2-Trifluoro-*N*-(1-(3-methoxyphenyl)-4-phenylbut-1-en-1-yl)acetamide (*Z*:*E* = 75:25) (EA12). Prepared according to the ***General Procedure for the Synthesis of Enamides from Primary Amides*** using 3-methoxybenzamide (35.3 mg, 0.25 mmol), $PhCH_2CH_2CH(Bpin)_2$ (186.0 mg, 0.50 mmol), ^{n}BuLi (0.40 mL, 1.0 mmol, 2.5 M in hexane) and THF (2 mL), 0 °C, 5 min then 100 °C, 12 h. Upon completion, ^{t}BuOH (0.50 mL) were added under N_2 atmosphere, then stirred at 100 °C for another 0.5 h. TFAA (1.0 mL) was then added under N_2 atmosphere, stirred at 100 °C for 2 h. The reaction

mixture was quenched with saturated sodium bicarbonate solution and purified by column chromatography (PE : EA = 10 : 1) to give 50.6 mg (58% yield) of product as a white solid, mp: 66-75 °C. **^{1}H NMR** (400 MHz, $CDCl_3$) δ 7.33 – 7.23 (m, 3H), 7.22 – 7.09 (m, 3H), 6.91 – 6.66 (m, 4H), 6.51 (t, *J* = 7.6 Hz, 0.25H), 5.97 (t, *J* = 7.6 Hz, 0.75H), 3.79 – 3.76 (m, 3H), 2.81 (t, *J* = 7.2 Hz, 1.5H), 2.72 (t, *J* = 7.2 Hz, 0.5H), 2.50 – 2.37 (m, 2H) ppm; **^{13}C NMR** (101 MHz, $CDCl_3$) δ 159.8, 155.3 (q, *J* = 37.2 Hz), 141.2, 141.0, 137.6, 136.4, 131.9, 131.6, 129.9, 129.7, 128.6(1), 128.5(7), 128.5, 128.3, 127.1, 126.3, 126.0, 122.4, 120.8, 120.1, 117.9, 117.2, 117.1, 114.4, 114.2, 113.8, 111.4, 55.3, 55.2, 36.0, 34.8, 30.3 ppm. **^{19}F NMR** (376 MHz, $CDCl_3$) δ -75.31, -75.84 ppm; **HRMS** (ESI) calcd for $C_{19}H_{18}F_3NO_2$ $[M+Na]^+$: 372.1187; found: 372.1187.

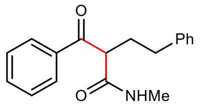

2-Benzoyl-*N*-methyl-4-phenylbutanamide (KA1). Prepared according to the ***General Procedure for the Synthesis of β-Ketoamide from N-Boc Amides*** using *tert*-Butyl benzoyl(methyl)carbamate (70.6 mg, 0.30 mmol), $PhCH_2CH_2CH(Bpin)_2$ (167.4 mg, 0.45 mmol), MeLi (0.28 mL, 0.45 mmol, 1.6 M in Et_2O), $B(O^iPr)_3$ (56.4 mg, 0.30 mmol) and THF (3.0 mL), 80 °C, 2 h. Upon completion, the reaction mixture was quenched with H_2O and purified by column chromatography (PE : EA = 2 : 1) to give 65.7 mg (78% yield) of product as a white solid, mp: 121-123 °C. **^{1}H NMR** (400 MHz, $CDCl_3$) δ 7.96 – 7.93 (m, 2H), 7.61 – 7.55 (m, 1H), 7.50 – 7.39 (m, 2H), 7.27 – 7.21 (m, 2H), 7.20 – 7.11 (m, 3H), 6.49 (s, br, 1H), 4.38 (t, *J* = 7.2 Hz, 1H), 2.78 (d, *J* = 4.8 Hz, 3H), 2.72 – 2.57 (m, 2H), 2.41 – 2.18 (m, 2H) ppm; **^{13}C NMR** (101 MHz, $CDCl_3$) δ 199.4, 169.2, 140.6, 136.3, 133.9, 128.8, 128.6, 128.5, 128.4, 126.2, 54.9, 33.8, 33.5, 26.5 ppm; **HRMS** (ESI) calcd for $C_{18}H_{19}NO_2$ $[M+Na]^+$: 304.1313; found:304.1305.

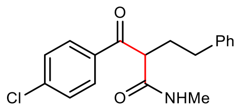

2-(4-Chlorobenzoyl)-*N*-methyl-4-phenylbutanamide (KA2). Prepared according to the ***General Procedure for the Synthesis of β-Ketoamide from N-Boc Amides*** using

tert-Butyl (4-chlorobenzoyl)(methyl)carbamate (80.7 mg, 0.30 mmol), $PhCH_2CH_2CH(Bpin)_2$ (167.4 mg, 0.45 mmol), MeLi (0.28 mL, 0.45 mmol, 1.6 M in Et_2O), $B(O^iPr)_3$ (56.4 mg, 0.30 mmol) and THF (3.0 mL), 80 ºC, 2 h. Upon completion, the reaction mixture was quenched with H_2O and purified by column chromatography (PE : EA = 2 : 1) to give 49.7 mg (52% yield) of product as a white solid, mp: 180-183 ºC. **^{1}H NMR** (400 MHz, $CDCl_3$) δ 7.91 (d, *J* = 8.4 Hz, 2H), 7.46 (d, *J* = 8.4 Hz, 2H), 7.35 – 7.29 (m, 2H), 7.26 – 7.13 (m, 3H), 6.41 (s, br, 1H), 4.35 (t, *J* = 7.2 Hz, 1H), 2.83 (d, *J* = 7.2 Hz, 3H), 2.74 – 2.64 (m, 2H), 2.46 – 2.20 (m, 2H) ppm; **^{13}C NMR** (101 MHz, $CDCl_3$) δ 198.2, 169.0, 140.6, 140.4, 134.6, 130.1, 129.2, 128.6, 128.5, 126.3, 55.0, 33.7, 33.5, 26.5 ppm; **HRMS** (ESI) calcd for $C_{18}H_{18}ClNO_2$ $[M+H]^+$: 316.1104; found: 316.1098.

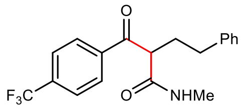

N*-Methyl-4-phenyl-2-(4-(trifluoromethyl)benzoyl)butanamide (KA3).** Prepared according to the ***General Procedure for the Synthesis of β-Ketoamide from N-Boc Amides using *tert*-Butyl methyl(4-(trifluoromethyl)benzoyl)carbamate (90.9 mg, 0.30 mmol), $PhCH_2CH_2CH(Bpin)_2$ (167.4 mg, 0.45 mmol), MeLi (0.28 mL, 0.45 mmol, 1.6 M in Et_2O), $B(O^iPr)_3$ (56.4 mg, 0.30 mmol) and THF (3.0 mL), 80 ºC, 2 h. Upon completion, the reaction mixture was quenched with H_2O and purified by column chromatography (PE : EA = 2 : 1) to give 66.4 mg (63% yield) of product as a white solid, mp: 170-173 ºC. **^{1}H NMR** (400 MHz, $CDCl_3$) δ 8.03 (d, *J* = 8.0 Hz, 2H), 7.71 (d, *J* = 8.0 Hz, 2H), 7.29 – 7.23 (m, 2H), 7.23 – 7.17 (m, 1H), 7.17 – 7.10 (m, 2H), 6.30 (s, br, 1H), 4.35 (t, *J* = 7.2 Hz, 1H), 2.80 (d, *J* = 4.8 Hz, 3H), 2.74 – 2.58 (m, 2H), 2.44 – 2.16 (m, 2H) ppm; **^{13}C NMR** (101 MHz, $CDCl_3$) δ 198.3, 168.6, 140.2, 138.8, 135.2, 134.8, 129.0, 128.5, 126.4, 125.8 (q, *J* = 3.8 Hz), 124.7, 122.0 ppm; **HRMS** (ESI) calcd for $C_{19}H_{18}F_3NO_2$ $[M+H]^+$: 350.1368; found: 350.1364.

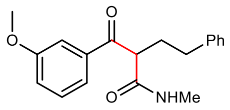

2-(3-Methoxybenzoyl)-*N*-methyl-4-phenylbutanamide (KA4). Prepared according to the ***General Procedure for the Synthesis of β-Ketoamide from N-Boc Amides*** using *tert*-Butyl (3-methoxybenzoyl)(methyl)carbamate (79.5 mg, 0.30 mmol), $PhCH_2CH_2CH(Bpin)_2$ (167.4 mg, 0.45 mmol), MeLi (0.28 mL, 0.45 mmol, 1.6 M in Et_2O), $B(O^iPr)_3$ (56.4 mg, 0.30 mmol) and THF (3.0 mL), 80 °C, 2 h. Upon completion, the reaction mixture was quenched with H_2O and purified by column chromatography (PE : EA = 1 : 1) to give 63.8 mg (68% yield) of product as a white solid, mp: 132-134 °C. **^{1}H NMR** (400 MHz, $CDCl_3$) δ 7.53 (d, *J* = 8.0 Hz, 1H), 7.49 – 7.43 (m, 1H), 7.36 (t, *J* = 8.0 Hz, 1H), 7.29 – 7.22 (m, 2H), 7.22 – 7.10 (m, 4H), 6.46 (s, br, 1H), 4.37 (t, *J* = 7.2 Hz, 1H), 3.84 (s, 3H), 2.79 (d, *J* = 5.2 Hz, 3H), 2.73 – 2.56 (m, 2H), 2.41 – 2.16 (m, 2H) ppm; **^{13}C NMR** (101 MHz, $CDCl_3$) δ 199.4, 169.2, 159.9, 140.5, 137.5, 129.8, 128.5, 128.4, 126.2, 121.4, 120.8, 112.4, 55.4, 54.9, 34.1, 33.5, 26.5 ppm; **HRMS** (ESI) calcd for $C_{19}H_{21}NO_3$ $[M+H]^+$: 312.1600; found: 312.1590.

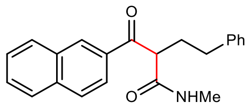

2-(2-Naphthoyl)-*N*-methyl-4-phenylbutanamide (KA5). Prepared according to the ***General Procedure for the Synthesis of β-Ketoamide from N-Boc Amides*** using *tert*-Butyl (2-naphthoyl)(methyl)carbamate (85.5 mg, 0.30 mmol), $PhCH_2CH_2CH(Bpin)_2$ (167.4 mg, 0.45 mmol), MeLi (0.28 mL, 0.45 mmol, 1.6 M in Et_2O), $B(O^iPr)_3$ (56.4 mg, 0.30 mmol) and THF (3.0 mL),80 °C, 2 h. Upon completion, the reaction mixture was quenched with H_2O and purified by column chromatography (PE : EA = 2 : 1) to give 66.5 mg (67% yield) of product as a white solid, mp: 166-169 °C. **^{1}H NMR** (400 MHz, $CDCl_3$) δ 8.42 (s, 1H), 8.00 (d, *J* = 8.4 Hz, 1H), 7.95 – 7.80 (m, 3H), 7.67 – 7.50 (m, 2H), 7.30 – 7.11 (m, 5H), 6.59 (s, br, 1H), 4.57 (t, *J* = 7.2 Hz, 1H), 2.81 (d, *J* = 4.8 Hz, 3H), 2.75 – 2.62 (m, 2H), 2.47 – 2.24 (m, 2H) ppm; **^{13}C NMR** (101 MHz, $CDCl_3$) δ 199.6, 169.4, 140.6, 135.9, 133.6, 132.4, 131.1, 129.9, 129.0, 128.7, 128.6, 128.5, 127.7, 127.0, 126.2, 123.8, 54.7, 34.2, 33.6, 26.5 ppm; **HRMS** (ESI) calcd for $C_{22}H_{21}NO_2$ $[M+H]^+$: 332.1651; found: 332.1645.

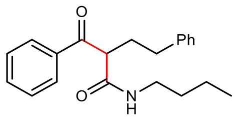

2-Benzoyl-*N*-butyl-4-phenylbutanamide (KA6). Prepared according to the ***General Procedure for the Synthesis of β-Ketoamide from N-Boc Amides*** using *tert*-Butyl benzoyl(butyl)carbamate (83.1 mg, 0.30 mmol), $PhCH_2CH_2CH(Bpin)_2$ (167.4 mg, 0.45 mmol), MeLi (0.28 mL, 0.45 mmol, 1.6 M in Et_2O), $B(O^iPr)_3$ (56.4 mg, 0.30 mmol) and THF (3.0 mL), 80 °C, 2 h. Upon completion, the reaction mixture was quenched with H_2O and purified by column chromatography (PE : EA = 2 : 1) to give 62.8 mg (65% yield) of product as a white solid, mp: 98-101 °C. **^{1}H NMR** (400 MHz, $CDCl_3$) δ 7.99 – 7.91 (m, 2H), 7.61 – 7.54 (m, 1H), 7.49 – 7.40 (m, 2H), 7.29 – 7.22 (m, 2H), 7.21 – 7.11 (m, 3H), 6.45 (t, *J* = 7.2 Hz, 1H), 4.36 (t, *J* = 7.2 Hz, 1H), 3.35 – 3.11 (m, 2H), 2.71 – 2.60 (m, 2H), 2.41 – 2.15 (m, 2H), 1.52 – 1.39 (m, 2H), 1.35 – 1.22 (m, 2H), 0.87 (t, *J* = 7.2 Hz, 3H) ppm; **^{13}C NMR** (101 MHz, $CDCl_3$) δ 199.4, 168.5, 140.6, 136.3, 133.8, 128.7, 128.6, 128.5, 128.4, 126.1, 55.2, 39.4, 33.8, 33.5, 31.4, 19.9, 13.6 ppm; **HRMS** (ESI) calcd for $C_{21}H_{25}NO_2$ $[M+H]^+$: 324.1964; found: 324.1957.

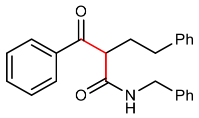

2-Benzoyl-*N*-benzyl-4-phenylbutanamide (KA7). Prepared according to the ***General Procedure for the Synthesis of β-Ketoamide from N-Boc Amides*** using *tert*-butyl benzoyl(benzyl)carbamate (93.3 mg, 0.30 mmol), $PhCH_2CH_2CH(Bpin)_2$ (167.4 mg, 0.45 mmol), MeLi (0.28 mL, 0.45 mmol, 1.6 M in Et_2O), $B(O^iPr)_3$ (56.4 mg, 0.30 mmol) and THF (3.0 mL), 80 °C, 2 h. Upon completion, the reaction mixture was quenched with H_2O and purified by column chromatography (PE : EA = 2 : 1) to give 77.3 mg (72% yield) of product as a white solid, mp: 130-133 °C. **^{1}H NMR** (400 MHz, $CDCl_3$) δ 7.93 (d, *J* = 7.2 Hz, 2H), 7.58 (d, *J* = 7.2 Hz, 1H), 7.44 (d, *J* = 7.6 Hz, 2H), 7.31 – 7.06 (m, 10H), 6.92 – 6.82 (m, 1H), 4.54 – 4.38 (m, 2H), 4.37 – 4.28 (m, 1H), 2.66 (d, *J* = 7.2 Hz, 2H), 2.40 – 2.20 (m, 2H) ppm; **^{13}C NMR** (101 MHz, $CDCl_3$) δ 199.0, 168.5, 140.5, 137.9, 136.1, 133.9, 128.8, 128.6(2), 128.6(0), 128.5, 128.4, 127.4(2), 127.3(7), 126.2, 54.9, 43.5, 33.7, 33.5 ppm; **HRMS** (ESI) calcd for $C_{24}H_{23}NO_2$ $[M+H]^+$:

358.1807; found: 358.1801.

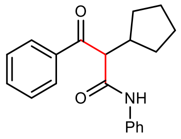

2-Cyclopentyl-3-oxo-N,3-diphenylpropanamide (KA8). Prepared according to the ***General Procedure for the Synthesis of β-Ketoamide from N-Boc Amides*** using *tert*-butyl benzoyl(phenyl)carbamate (89.1 mg, 0.30 mmol), 2,2'-(cyclopentylmethylene)bis(4,4,5,5-tetramethyl-1,3,2-dioxaborolane) (151.2 mg, 0.45 mmol), MeLi (0.28 mL, 0.45 mmol, 1.6 M in Et_2O), $B(O^iPr)_3$ (56.4 mg, 0.30 mmol) and THF (3.0 mL), 80 °C, 2 h. Upon completion, the reaction mixture was quenched with H_2O and purified by column chromatography (PE : EA = 2 : 1) to give 40.9 mg (44% yield) of product as a white solid, mp: 169-171 °C. **^{1}H NMR** (400 MHz, $CDCl_3$) δ 8.58 (s, br, 1H), 8.07 (t, *J* = 8.0 Hz, 2H), 7.62 (t, *J* = 7.2 Hz, 1H), 7.57 – 7.46 (m, 4H), 7.31 (t, *J* = 8.0 Hz, 2H), 7.09 (t, *J* = 7.6 Hz, 1H), 4.32 (d, *J* = 10.4 Hz, 1H), 2.68 – 2.52 (m, 1H), 1.96 – 1.81 (m, 1H), 1.78 – 1.69 (m, 2H), 1.65 – 1.48 (m, 4H), 1.17 – 1.03 (m, 1H) ppm; **^{13}C NMR** (101 MHz, $CDCl_3$) δ 200.9, 166.4, 137.6, 136.9, 134.2, 128.9, 128.8, 124.3, 119.8, 61.7, 44.6, 31.4, 30.4, 24.7, 24.3 ppm; **HRMS** (ESI) calcd for $C_{20}H_{21}NO_2$ $[M+H]^+$: 308.1651; found: 308.1645.

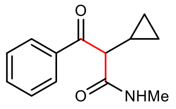

2-Cyclopropyl-*N*-methyl-3-oxo-3-phenylpropanamide (KA9). Prepared according to the ***General Procedure for the Synthesis of β-Ketoamide from N-Boc Amides*** using *tert*-Butyl benzoyl(methyl)carbamate (70.6 mg, 0.30 mmol), 2,2'-(cyclopropylmethylene)bis(4,4,5,5-tetramethyl-1,3,2-dioxaborolane) (138.6 mg, 0.45 mmol), MeLi (0.28 mL, 0.45 mmol, 1.6 M in Et_2O), $B(O^iPr)_3$ (56.4 mg, 0.30 mmol) and THF (3.0 mL), 80 °C, 2 h. Upon completion, the reaction mixture was quenched with H_2O and purified by column chromatography (PE : EA = 2 : 1) to give 49.1 mg (75% yield) of product as a white solid, mp: 174-177 °C. **^{1}H NMR** (400 MHz, $CDCl_3$) δ 8.02 – 7.96 (m, 2H), 7.64 – 7.56 (m, 1H), 7.52 – 7.45 (m, 2H), 6.66 (s, br, 1H), 3.65

(d, J = 9.6 Hz, 1H), 2.84 (d, J = 4.8 Hz, 3H), 1.47 – 1.35 (m, 1H), 0.71 – 0.63 (m, 1H), 0.62 – 0.49 (m, 2H), 0.28 – 0.21 (m, 1H) ppm; **^{13}C NMR** (101 MHz, $CDCl_3$) δ 199.5, 169.4, 136.6, 133.8, 128.8, 126.8, 59.4, 26.5, 13.3, 4.7, 3.6 ppm; **HRMS** (ESI) calcd for $C_{13}H_{15}NO_2$ $[M+Na]^+$: 240.1000; found: 240.0993.

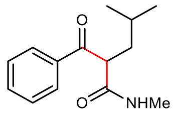

2-Benzoyl-*N*,4-dimethylpentanamide (KA10). Prepared according to the ***General Procedure for the Synthesis of β-Ketoamide from N-Boc Amides*** using *tert*-Butyl benzoyl(methyl)carbamate (70.6 mg, 0.30 mmol), 2,2'-(3-methylbutane-1,1-diyl)bis(4,4,5,5-tetramethyl-1,3,2-dioxaborolane) (145.8 mg, 0.45 mmol), MeLi (0.28 mL, 0.45 mmol, 1.6 M in Et_2O), $B(O^iPr)_3$ (56.4 mg, 0.30 mmol) and THF (3.0 mL), 80 °C, 2 h. Upon completion, the reaction mixture was quenched with H_2O and purified by column chromatography (PE : EA = 2 : 1) to give 46.0 mg (66% yield) of product as a white solid, mp: 129-132 °C. **^{1}H NMR** (400 MHz, $CDCl_3$) δ 8.12 – 7.98 (m, 2H), 7.64 – 7.57 (m, 1H), 7.52 – 7.45 (m, 2H), 6.38 (s, br, 1H), 4.48 (t, J = 7.2 Hz, 1H), 2.78 (d, J = 7.2 Hz, 3H), 2.00 – 1.73 (m, 2H), 1.69 – 1.52 (m, 1H), 0.93 (t, J = 6.4 Hz, 3H), 0.90 (t, J = 6.8 Hz, 3H) ppm; **^{13}C NMR** (101 MHz, $CDCl_3$) δ 199.9, 169.5, 136.5, 133.8, 128.8, 128.6, 54.3, 41.4, 26.6, 26.5, 22.5, 22.4 ppm; **HRMS** (ESI) calcd for $C_{14}H_{19}NO_2$ $[M+H]^+$: 234.1494; found: 234.1488.

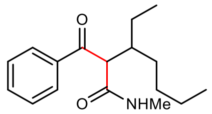

2-Benzoyl-4-ethyl-*N*-methyloctanamide (KA11). Prepared according to the ***General Procedure for the Synthesis of β-Ketoamide from N-Boc Amides*** using *tert*-Butyl benzoyl(methyl)carbamate (70.6 mg, 0.30 mmol), 2,2'-(3-ethylheptane-1,1-diyl)bis(4,4,5,5-tetramethyl-1,3,2-dioxaborolane) (171.0 mg, 0.45 mmol), MeLi (0.28 mL, 0.45 mmol, 1.6 M in Et_2O), $B(O^iPr)_3$ (56.4 mg, 0.30 mmol) and THF (3.0 mL), 80 °C, 2 h. Upon completion, the reaction mixture was quenched with H_2O and purified by column chromatography (PE : EA = 2 : 1) to give 53.5 mg (65% yield) of product

as a white solid, mp: 147-149 °C. The *d.r.* value was not determined. **^{1}H NMR** (400 MHz, $CDCl_3$) δ 8.03 (d, *J* = 7.6 Hz, 2H), 7.67 – 7.55 (m, 1H), 7.54 – 7.44 (m, 2H), 6.57 (s, br, 1H), 4.41 (d, *J* = 10.0 Hz, 1H), 2.78 (d, *J* = 4.4 Hz, 3H), 2.29 – 2.13 (m, 1H), 1.62 – 1.02 (m, 10H), 0.98 – 0.85 (m, 3H), 0.78 (t, *J* = 7.2 Hz, 3H) ppm; **^{13}C NMR** (101 MHz, $CDCl_3$) δ 201.1, 201.0, 168.7, 137.3, 137.2, 133.9, 128.8, 128.6, 59.6, 59.5, 42.7(1), 42.6(8), 30.0, 29.0, 28.1, 28.0, 26.4, 23.4, 22.9, 22.7, 22.4, 14.0, 13.9, 10.1, 9.8 ppm; **HRMS** (ESI) calcd for $C_{17}H_{25}NO_2$ $[M+H]^+$: 276.1964; found: 276.1955.

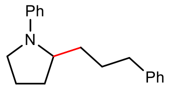

1-Phenyl-2-(3-phenylpropyl)pyrrolidine (A1). Prepared according to the ***General Procedure for the Synthesis of tert-Alkylamines from Tertiary Lactams*** using 1-phenylpyrrolidin-2-one (40.3 mg, 0.25 mmol), $PhCH_2CH_2CH(Bpin)_2$ (139.8 mg, 0.375 mmol), MeLi (0.24 mL, 0.375 mmol, 1.6 M in Et_2O) and anisole (2.0 mL), 0 °C, 5 min then 120 °C, 2 h. Upon completion, $NaBH_4$ (28.4 mg, 0.75 mmol), MeOH (1.0 mL) were added under N_2 atmosphere, then stirred at room temperature for another 1 h. The reaction mixture was quenched with H_2O and purified by column chromatography (PE : EA = 50 : 1) to give 48.3 mg (73% yield) of product as a colorless oil. **^{1}H NMR** (400 MHz, $CDCl_3$) δ 7.30 – 7.26 (m, 2H), 7.23 – 7.16 (m, 5H), 6.63 (t, *J* = 7.6 Hz, 1H), 6.52 (d, *J* = 8.0 Hz, 2H), 3.67 – 3.62 (m, 1H), 3.39 (t, *J* = 7.6 Hz, 1H), 3.17 – 3.07 (m, 1H), 2.72 – 2.58 (m, 2H), 2.01 – 1.91 (m, 3H), 1.82 – 1.74 (m, 2H), 1.71 – 1.64 (m, 2H), 1.38 – 1.30 (m, 1H) ppm; **^{13}C NMR** (101 MHz, $CDCl_3$) δ 147.2, 142.3, 129.1, 128.3(2), 128.2(9), 125.7, 115.2, 111.7, 58.5, 48.2, 36.0, 32.7, 30.2, 28.4, 23.5 ppm; **HRMS** (ESI) calcd for $C_{19}H_{23}N$ $[M+H]^+$: 266.1909; found: 266.1906.

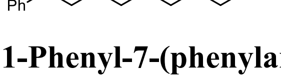

1-Phenyl-7-(phenylamino)heptan-4-one (A1'). Prepared according to the ***General Procedure for the Synthesis of tert-Alkylamines from Tertiary Lactams*** using 1-phenylpyrrolidin-2-one (40.3 mg, 0.25 mmol), $PhCH_2CH_2CH(Bpin)_2$ (139.8 mg, 0.375 mmol), MeLi (0.24 mL, 0.375 mmol, 1.6 M in Et_2O) and anisole (2.0 mL), 0 °C, 5 min

then 120 °C, 2 h. Upon completion, 2 M HCl (1.0 mL) was added, then heated at 80 °C for 1 h. Subsequently, 3 M NaOH (1.0 mL) was added. then heated at 80 °C for another 1 h. The reaction mixture was quenched with H_2O and purified by fast column chromatography (DCM : MeOH = 20 : 1) to give 54.7 mg (78% yield) of product as yellow oil. *It is worth noting that the product was unstable.* **^{1}H NMR** (400 MHz, $CDCl_3$) δ 7.29 – 7.25 (m, 2H), 7.20 – 7.13 (m, 5H), 6.69 (t, *J* = 7.2 Hz, 1H), 6.58 (d, *J* = 8.0 Hz, 2H), 3.72 – 3.61 (m, 1H), 3.10 (t, *J* = 6.8 Hz, 2H), 2.61 (t, *J* = 7.6 Hz, 2H), 2.50 (t, *J* = 7.2 Hz, 2H), 2.41 (t, *J* = 7.2 Hz, 2H), 1.94 – 1.83 (m, 4H) ppm; **^{13}C NMR** (101 MHz, $CDCl_3$) δ 210.6, 148.2, 141.5, 129.2, 128.4, 128.3, 125.9, 117.2, 112.6, 43.4, 42.0, 40.2, 35.0, 25.1, 23.3 ppm; **HRMS** (ESI) calcd for $C_{19}H_{23}NO$ $[M+H]^+$: 282.1858; found: 282.1849.

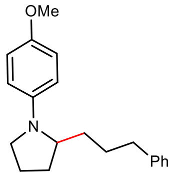

1-(4-Methoxyphenyl)-2-(3-phenylpropyl)pyrrolidine (A2). Prepared according to the ***General Procedure for the Synthesis of tert-Alkylamines from Tertiary Lactams*** using 1-(4-methoxyphenyl)pyrrolidin-2-one (47.8 mg, 0.25 mmol), $PhCH_2CH_2CH(Bpin)_2$ (139.8 mg, 0.375 mmol), MeLi (0.24 mL, 0.375 mmol, 1.6 M in Et_2O) and anisole (2.0 mL), 0 °C, 5 min then 120 °C, 2 h. Upon completion, $NaBH_4$ (28.4 mg, 0.75 mmol), MeOH (1.0 mL) were added under N_2 atmosphere, then stirred at room temperature for another 1 h. The reaction mixture was quenched with H_2O and purified by column chromatography (PE : EA = 30 : 1) to give 64.7 mg (88% yield) of product as a colorless oil. **^{1}H NMR** (400 MHz, $CDCl_3$) δ 7.27 (t, *J* = 7.6 Hz, 2H), 7.19 – 7.15 (m, 3H), 6.82 (d, *J* = 8.8 Hz, 2H), 6.48 (d, *J* = 8.4 Hz, 2H), 3.75 (s, 3H), 3.56 – 3.54 (m, 1H), 3.40 – 3.36 (m, 1H), 3.10 – 3.04 (m, 1H), 2.71 – 2.53 (m, 2H), 2.01 – 1.89 (m, 3H), 1.80 – 1.63 (m, 4H), 1.38 – 1.26 (m, 1H) ppm; **^{13}C NMR** (101 MHz, $CDCl_3$) δ 150.6, 142,38, 128.3(3), 128.2(8), 125.7, 115.0, 112.7, 59.0, 56.0, 49.1, 36.0, 33.1, 30.4, 28.4, 23.6 ppm; **HRMS** (ESI) calcd for $C_{20}H_{25}NO$ $[M+H]^+$: 296.2014; found:

296.2011.

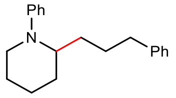

1-Phenyl-2-(3-phenylpropyl)piperidine (A3). Prepared according to the ***General Procedure for the Synthesis of tert-Alkylamines from Tertiary Lactams*** using 1-phenylpiperidin-2-one (43.8 mg, 0.25 mmol), $PhCH_2CH_2CH(Bpin)_2$ (139.8 mg, 0.375 mmol), MeLi (0.24 mL, 0.375 mmol, 1.6 M in Et_2O) and anisole (2.0 mL), 0 °C, 5 min then 120 °C, 2 h. Upon completion, $NaBH_4$ (28.4 mg, 0.75 mmol), MeOH (1.0 mL) were added under N_2 atmosphere, then stirred at room temperature for another 1 h. The reaction mixture was quenched with H_2O and purified by column chromatography (PE : EA = 50 : 1) to give 28.6 mg (41% yield) of product as a colorless oil. **^{1}H NMR** (400 MHz, $CDCl_3$) δ 7.26 – 7.20 (m, 4H), 7.17 – 7.10 (m, 3H), 6.87 (d, *J* = 8.0 Hz, 2H), 6.76 (d, *J* = 7.2 Hz, 1H), 3.82 – 3.77 (m, 1H), 3.35 – 3.30 (m, 1H), 3.00 – 2.93 (m, 1H), 2.62 – 2.47 (m, 2H), 1.81 – 1.50 (m, 10H) ppm; **^{13}C NMR** (101 MHz, $CDCl_3$) δ 151.2, 142.5, 129.1, 128.3, 128.2, 125.6, 118.1, 116.4, 55.7, 43.8, 35.9, 28.8, 27.8, 27.2, 25.4, 19.4 ppm; **HRMS** (ESI) calcd for $C_{20}H_{25}N$ $[M+H]^+$: 280.2065; found: 280.2063.

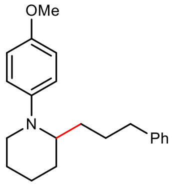

1-(4-Methoxyphenyl)-2-(3-phenylpropyl)piperidine (A4). Prepared according to the ***General Procedure for the Synthesis of tert-Alkylamines from Tertiary Lactams*** using 1-(4-methoxyphenyl)piperidin-2-one (51.3 mg, 0.25 mmol), $PhCH_2CH_2CH(Bpin)_2$ (139.8 mg, 0.375 mmol), MeLi (0.24 mL, 0.375 mmol, 1.6 M in Et_2O) and anisole (2.0 mL), 0 °C, 5 min then 120 °C, 2 h. Upon completion, $NaBH_4$ (28.4 mg, 0.75 mmol), MeOH (1.0 mL) were added under N_2 atmosphere, then stirred at room temperature for another 1 h. The reaction mixture was quenched with H_2O and purified by column chromatography (PE : EA = 30 : 1) to give 36.8 mg (50% yield) of product as a colorless

oil. ^{1}**H NMR** (400 MHz, $CDCl_3$) δ 7.25 – 7.21 (m, 2H), 7.16 – 7.12 (m, 1H), 7.09 – 7.07 (m, 2H), 6.92 – 6.88 (m, 2H), 6.83 – 6.79 (m, 2H), 3.77 (s, 3H), 3.40 – 3.36 (m, 1H), 3.01 – 2.93 (m, 2H), 2.56 – 2.41 (m, 2H), 1.84 – 1.77 (m, 1H), 1.68 – 1.40 (m, 9H) ppm; ^{13}**C NMR** (101 MHz, $CDCl_3$) δ 153.7, 145.8, 142.5, 128.3, 128.2, 125.6, 120.7, 114.3, 58.0, 55.5, 48.4, 35.9, 28.9, 28.4, 28.3, 25.9, 20.8 ppm; **HRMS** (ESI) calcd for $C_{21}H_{27}NO$ $[M+H]^+$: 310.2171; found: 310.2169.

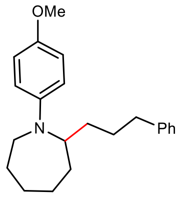

1-(4-Methoxyphenyl)-2-(3-phenylpropyl)azepane (A5). Prepared according to the ***General Procedure for the Synthesis of tert-Alkylamines from Tertiary Lactams*** using 1-(4-methoxyphenyl)azepan-2-one (54.8 mg, 0.25 mmol), $PhCH_2CH_2CH(Bpin)_2$ (139.8 mg, 0.375 mmol), MeLi (0.24 mL, 0.375 mmol, 1.6 M in Et_2O) and anisole (2.0 mL), 0 °C, 5 min then 120 °C, 2 h. Upon completion, $NaBH_4$ (28.4 mg, 0.75 mmol), MeOH (1.0 mL) were added under N_2 atmosphere, then stirred at room temperature for another 1 h. The reaction mixture was quenched with H_2O and purified by column chromatography (PE : EA = 50 : 1) to give 45.3 mg (56% yield) of product as a colorless oil. ^{1}**H NMR** (400 MHz, $CDCl_3$) δ 7.29 – 7.24 (m, 2H), 7.19 – 7.15 (m, 3H), 6.82 – 6.79 (m, 2H), 6.58 – 6.56 (m, 2H), 3.75 (s, 3H), 3.61 – 3.56 (m, 1H), 3.43 – 3.39 (m, 1H), 3.17 – 3.10 (m, 1H), 2.68 – 2.56 (m, 2H), 2.20 – 2.13 (m, 1H), 1.78 – 1.58 (m, 7H), 1.39 – 1.17 (m, 4H) ppm; ^{13}**C NMR** (101 MHz, $CDCl_3$) δ 149.6, 143.3, 142.4, 128.4, 128.3, 125.7, 115.1, 111.0, 56.7, 56.0, 43.3, 36.2, 35.0, 33.3, 30.0, 28.0, 27.0, 25.3 ppm; **HRMS** (ESI) calcd for $C_{22}H_{29}NO$ $[M+H]^+$: 324.2327; found: 324.2324.

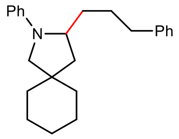

2-Phenyl-3-(3-phenylpropyl)-2-azaspiro[4.5]decane (A6). Prepared according to the ***General Procedure for the Synthesis of tert-Alkylamines from Tertiary Lactams*** using

2-phenyl-2-azaspiro[4.5]decan-3-one (57.3 mg, 0.25 mmol), $PhCH_2CH_2CH(Bpin)_2$ (139.8 mg, 0.375 mmol), MeLi (0.24 mL, 0.375 mmol, 1.6 M in Et_2O) and anisole (2.0 mL), 0 °C, 5 min then 120 °C, 2 h. Upon completion, $NaBH_4$ (28.4 mg, 0.75 mmol), MeOH (1.0 mL) were added under N_2 atmosphere, then stirred at room temperature for another 1 h. The reaction mixture was quenched with H_2O and purified by column chromatography (PE : EA = 30 : 1) to give 42.4 mg (51% yield) of product as a colorless oil. **^{1}H NMR** (400 MHz, $CDCl_3$) δ 7.29 – 7.25 (m, 2H), 7.3 – 7.15 (m, 5H), 6.66 – 6.59 (m, 1H), 6.52 (d, *J* = 8.0 Hz, 2H), 3.73 – 3.66 (m, 1H), 3.21 – 3.16 (m, 2H), 2.69 – 2.55 (m, 2H), 2.07 – 1.94 (m, 2H), 1.68 – 1.58 (m, 2H), 1.55 – 1.23 (m, 12H) ppm; **^{13}C NMR** (101 MHz, $CDCl_3$) δ 147.9, 142.4, 128.9, 128.3(2), 128.2(6), 125.7, 115.3, 112.5, 60.4, 57.0, 41.2, 37.1, 35.9, 33.3, 27.7, 26.2, 23.9, 23.2 ppm; **HRMS** (ESI) calcd for $C_{24}H_{31}N$ $[M+H]^+$: 334.2535; found: 334.2533.

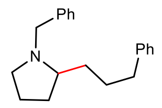

1-Benzyl-2-(3-phenylpropyl)pyrrolidine (A7). Prepared according to the ***General Procedure for the Synthesis of tert-Alkylamines from Tertiary Lactams*** using 1-benzylpyrrolidin-2-one (43.8 mg, 0.25 mmol), $PhCH_2CH_2CH(Bpin)_2$ (139.8 mg, 0.375 mmol), MeLi (0.24 mL, 0.375 mmol, 1.6 M in Et_2O) and anisole (2.0 mL), 0 °C, 5 min then 120 °C, 2 h. Upon completion, $NaBH_4$ (28.4 mg, 0.75 mmol), MeOH (1.0 mL) were added under N_2 atmosphere, then stirred at room temperature for another 1 h. The reaction mixture was quenched with H_2O and purified by column chromatography (PE : EA = 5 : 1) to give 50.2 mg (72% yield) of product as a colorless oil. **^{1}H NMR** (400 MHz, $CDCl_3$) δ 7.29 – 7.15 (m, 10H), 4.00 (d, *J* = 13.2 Hz, 1H), 3.12 (d, *J* = 12.8 Hz, 1H), 2.91 – 2.87 (m, 1H), 2.69 – 2.57 (m, 2H), 2.36 – 2.29 (m, 1H), 2.11 – 2.04 (m, 1H), 1.96 – 1.87 (m, 1H), 1.81 – 1.57 (m, 5H), 1.52 – 1.32 (m, 2H) ppm; **^{13}C NMR** (101 MHz, $CDCl_3$) δ 142.6, 139.6, 129.0, 128.4, 128.2, 128.1, 126.7, 125.6, 64.2, 58.4, 54.2, 36.3, 33.7, 30.3, 28.2, 21.9 ppm; **HRMS** (ESI) calcd for $C_{20}H_{25}N$ $[M+H]^+$: 280.2065; found: 280.2063.

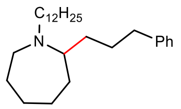

Dodecyl-2-(3-phenylpropyl)azepane (A8). Prepared according to the ***General Procedure for the Synthesis of tert-Alkylamines from Tertiary Lactams*** using 1-dodecylazepan-2-one (70.3 mg, 0.25 mmol), $PhCH_2CH_2CH(Bpin)_2$ (139.8 mg, 0.375 mmol), MeLi (0.24 mL, 0.375 mmol, 1.6 M in Et_2O) and anisole (2.0 mL), 0 °C, 5 min then 120 °C, 2 h. Upon completion, $NaBH_4$ (28.4 mg, 0.75 mmol), MeOH (1.0 mL) were added under N_2 atmosphere, then stirred at room temperature for another 1 h. The reaction mixture was quenched with H_2O and purified by column chromatography (DCM : MeOH = 10 : 1) to give 77.0 mg (80% yield) of product as a colorless oil. **^{1}H NMR** (400 MHz, $CDCl_3$) δ 7.34 – 7.31 (m, 2H), 7.25 – 7.20 (m, 3H), 2.97 – 2.91 (m, 1H), 2.74 – 2.49 (m, 6H), 1.70 – 1.30 (m, 32H), 0.95 (t, J = 6.8 Hz, 3H) ppm; **^{13}C NMR** (101 MHz, $CDCl_3$) δ 142.9, 128.4, 128.2, 125.5, 62.5, 51.2, 49.9, 36.3, 34.5, 33.1, 31.9, 29.7(4), 29.7(1), 29.6(7), 29.4, 29.1, 29.0, 28.7, 27.5, 27.1, 25.8, 22.7, 14.1 ppm; **HRMS** (ESI) calcd for $C_{27}H_{47}N$ $[M+H]^+$: 386.3787; found: 386.3787.

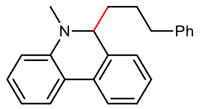

5-Methyl-6-(3-phenylpropyl)-5,6-dihydrophenanthridine (A9). Prepared according to the ***General Procedure for the Synthesis of tert-Alkylamines from Tertiary Lactams*** using 5-methylphenanthridin-6(5*H*)-one (52.3 mg, 0.25 mmol), $PhCH_2CH_2CH(Bpin)_2$ (139.8 mg, 0.375 mmol), MeLi (0.24 mL, 0.375 mmol, 1.6 M in Et_2O) and anisole (2.0 mL), 0 °C, 5 min then 120 °C, 2 h. Upon completion, $NaBH_4$ (28.4 mg, 0.75 mmol), MeOH (1.0 mL) were added under N_2 atmosphere, then stirred at room temperature for another 1 h. The reaction mixture was quenched with H_2O and purified by column chromatography (PE : EA = 30 : 1) to give 74.9 mg (96% yield) of product as a colorless oil. **^{1}H NMR** (400 MHz, $CDCl_3$) δ 7.74 – 7.71 (m, 2H), 7.36 – 7.27 (m, 1H), 7.27 – 7.12 (m, 5H), 7.06 – 7.02 (m, 3H), 6.87 – 6.78 (m, 1H), 6.65 (d, J = 8.0 Hz, 1H), 4.17 – 4.14 (m, 1H), 3.00 (s, 3H), 2.52 – 2.39 (m, 2H), 1.69 – 1.58 (m, 1H), 1.54 – 1.42 (m, 3H) ppm; **^{13}C NMR** (101 MHz, $CDCl_3$) δ 144.9, 142.2, 135.6, 130.9, 129.2, 128.3,

128.2, 127.4, 126.5, 126.4, 125.7, 123.1, 122.8, 122.4, 117.5, 112.6 ppm; **HRMS** (ESI) calcd for $C_{23}H_{23}N$ $[M+H]^+$: 314.1909; found: 314.1904.

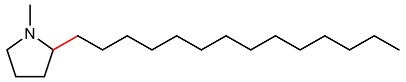

(±)-1-Methyl-2-tetradecylpyrrolidine ((±)-bgugaine) (A10).[22] Prepared according to the ***General Procedure for the Synthesis of tert-Alkylamines from Tertiary Lactams*** using 1-methylpyrrolidin-2-one (24.8 mg, 0.25 mmol), 2,2'-(tetradecane-1,1-diyl)bis(4,4,5,5-tetramethyl-1,3,2-dioxaborolane) (168.8 mg, 0.375 mmol), MeLi (0.24 mL, 0.375 mmol, 1.6 M in Et_2O) and anisole (2.0 mL), 0 °C, 5 min then 120 °C, 2 h. Upon completion, $NaBH_4$ (28.4 mg, 0.75 mmol), MeOH (1.0 mL) were added under N_2 atmosphere, then stirred at room temperature for another 1 h. The reaction mixture was quenched with H_2O and purified by column chromatography (EA : MeOH = 20 : 1) to give 59.0 mg (85% yield) of product as a colorless oil. **^{1}H NMR** (400 MHz, $CDCl_3$) δ 3.11 – 3.06 (m, 1H), 2.32 (s, 3H), 2.14 (dd, J = 18.0, 9.2 Hz, 1H), 2.00 – 1.89 (m, 2H), 1.80 – 1.63 (m, 3H), 1.48 – 1.40 (m, 1H), 1.30 – 1.21 (m, 25H), 0.88 (t, J = 6.8 Hz, 3H) ppm; **^{13}C NMR** (101 MHz, $CDCl_3$) δ 66.5, 57.3, 40.3, 33.7, 31.9, 30.7, 30.0, 29.6(4), 29.6(2), 29.5(8), 29.3, 26.7, 22.6, 21.7, 14.1 ppm.

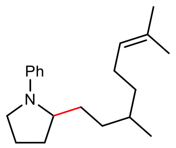

2-(3,7-Dimethyloct-6-en-1-yl)-1-phenylpyrrolidine (*d.r.* = 1:1) (A11). Prepared according to the ***General Procedure for the Synthesis of tert-Alkylamines from Tertiary Lactams*** using 1-phenylpyrrolidin-2-one (40.3 mg, 0.25 mmol), 2,2'-(3,7-dimethyloct-6-ene-1,1-diyl)bis(4,4,5,5-tetramethyl-1,3,2-dioxaborolane) (147.0 mg, 0.375 mmol), MeLi (0.24 mL, 0.375 mmol, 1.6 M in Et_2O) and anisole (2.0 mL), 0 °C, 5 min then 120 °C, 2 h. Upon completion, $NaBH_4$ (28.4 mg, 0.75 mmol), MeOH (1.0 mL) were added under N_2 atmosphere, then stirred at room temperature for another 1 h. The reaction mixture was quenched with H_2O and purified by column chromatography (PE : EA = 30 : 1) to give 44.9 mg (63% yield) of product as a colorless

oil,. **^{1}H NMR** (400 MHz, $CDCl_3$) δ 7.26 – 7.18 (m, 2H), 6.68 – 6.60 (m, 1H), 6.54 (d, *J* = 8.0, Hz, 2H), 5.14 – 5.05 (m, 1H), 3.63 – 3.54 (m, 1H), 3.45 – 3.37 (m, 1H), 3.20 – 3.10 (m, 1H), 2.07 – 1.90 (m, 5H), 1.68 (s, 3H), 1.61 (s, 3H), 1.46 – 1.07 (m, 8H), 0.89 (d, *J* = 2.8 Hz, 1.5 H), 0.88 (d, *J* = 2.8 Hz, 1.5 H), ppm; **^{13}C NMR** (101 MHz, $CDCl_3$) δ 147.3, 131.1, 129.2, 124.9, 115.1, 111.8, 59.1, 59.0, 48.2, 37.1, 36.9, 32.6(4), 32.5(6), 30.4, 30.3(3), 30.2(7), 25.7, 25.6, 23.5, 19.8, 19.7, 17.6 ppm; **HRMS** (ESI) calcd for $C_{20}H_{31}N$ $[M+H]^+$: 286.2535; found: 286.2533.

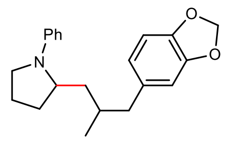

2-(3-(Benzo[d][1,3]dioxol-5-yl)-2-methylpropyl)-1-phenylpyrrolidine (*d.r.* = 1:1) (A12). Prepared according to the ***General Procedure for the Synthesis of tert-Alkylamines from Tertiary Lactams*** using 1-phenylpyrrolidin-2-one (40.3 mg, 0.25 mmol), 2,2'-(3-(benzo[d][1,3]dioxol-5-yl)-2-methylpropane-1,1-diyl)bis(4,4,5,5-tetramethyl-1,3,2-dioxaborolane) (161.3 mg, 0.375 mmol), MeLi (0.24 mL, 0.375 mmol, 1.6 M in Et_2O) and anisole (2.0 mL), 0 °C, 5 min then 120 °C, 2 h. Upon completion, $NaBH_4$ (28.4 mg, 0.75 mmol), MeOH (1.0 mL) were added under N_2 atmosphere, then stirred at room temperature for another 1 h. The reaction mixture was quenched with H_2O and purified by column chromatography (PE : EA = 30 : 1) to give 30.6 mg (38% yield) of product as a colorless oil. **^{1}H NMR** (400 MHz, $CDCl_3$) δ 7.23 – 7.16 (m, 2H), 6.75 – 6.54 (m, 5H), 6.46 (d, *J* = 8.4 Hz, 1H), 5.92 – 5.91 (m, 2H), 3.82 – 3.77 (m, 0.5H), 3.73 – 3.69 (m, 0.5H), 3.41 – 3.33 (m, 1H), 3.15 – 3.08 (m, 1H), 2.82 – 2.77 (m, 0.5H), 2.50 – 2.37 (m, 1H), 2.36 – 2.28 (m, 0.5H), 2.08 – 1.72 (m, 5H), 1.55 – 1.51 (m, 0.5H), 1.31 – 1.15 (m, 1.5H), 1.06 (d, *J* = 6.4 Hz, 1.5H), 0.90 (d, *J* = 6.4 Hz, 1.5H) ppm; **^{13}C NMR** (101 MHz, $CDCl_3$) δ 147.5, 147.4, 147.2, 147.1, 145.6, 145.5, 135.0, 134.8, 129.2, 129.1, 121.9, 115.2, 115.1, 111.8, 111.7, 109.4, 108.0, 107.9, 100.7, 100.7, 57.3, 56.4, 48.0, 44.5, 43.0, 40.1, 38.8, 33.6, 33.2, 30.9, 30.0, 23.3, 23.2, 21.0, 19.1 ppm; **HRMS** (ESI) calcd for $C_{21}H_{25}NO_2$ $[M+H]^+$: 324.1964; found: 324.1963.

Supplementary Note 2

Copies of Substrates and Products ^{1}H, ^{13}C, ^{19}F and NOESY spectra

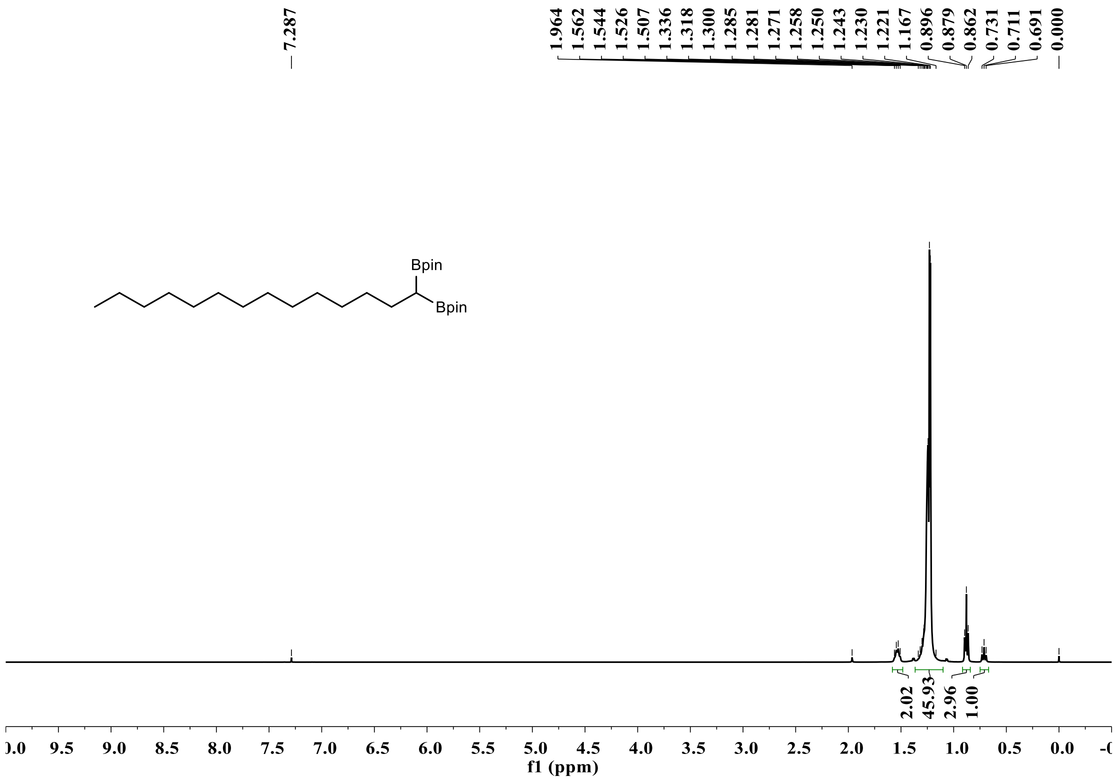

Supplementary Figure 5. ^{1}H NMR spectrum for **1o**

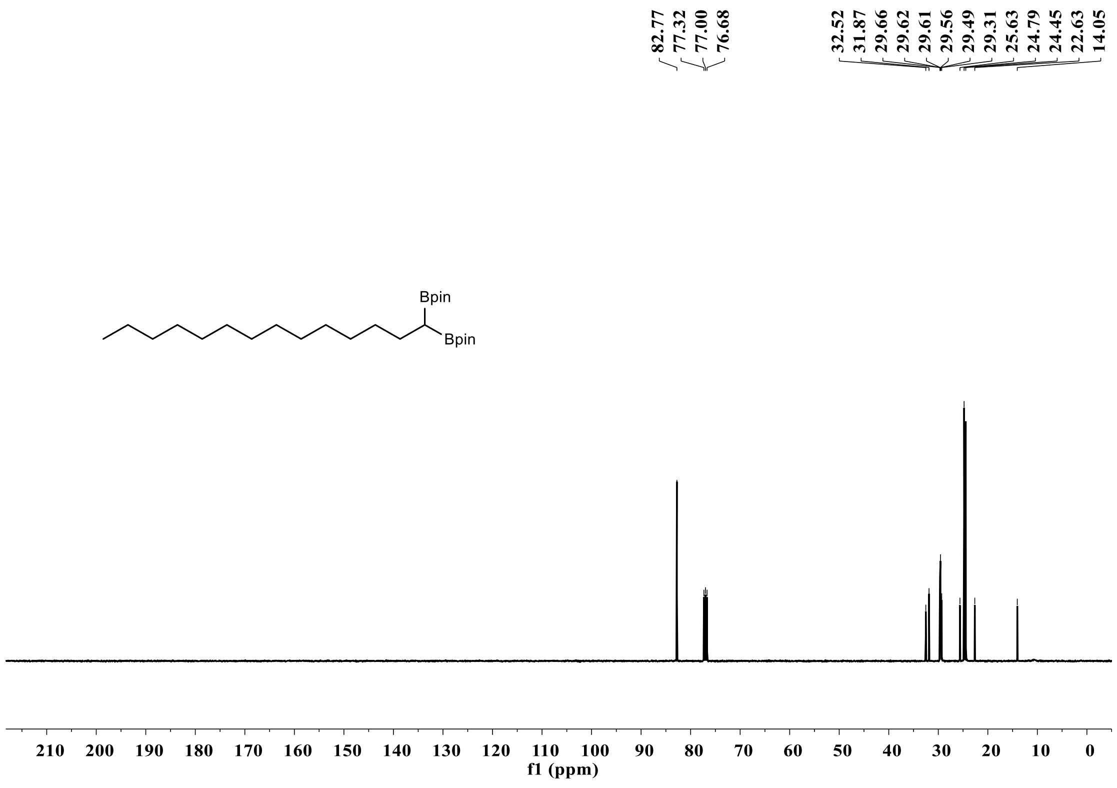

Supplementary Figure 6. ^{13}C NMR spectrum for **1o**

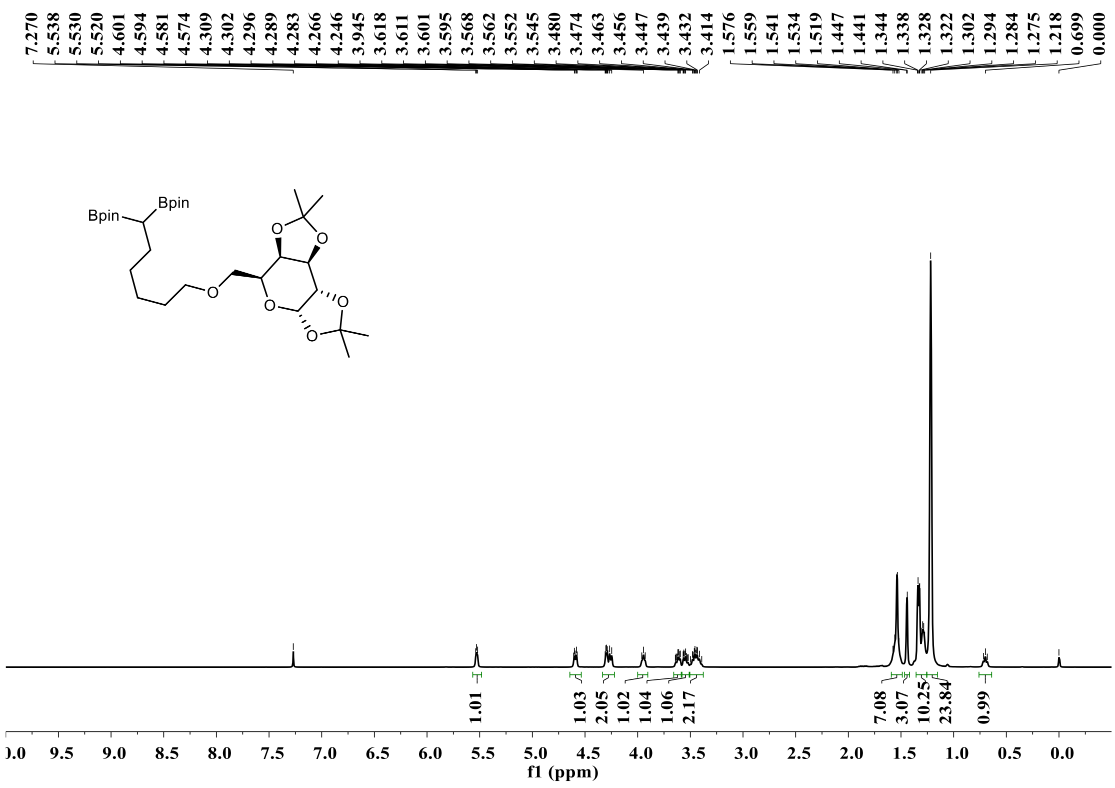

Supplementary Figure 7. ^{1}H NMR spectrum for **1p**

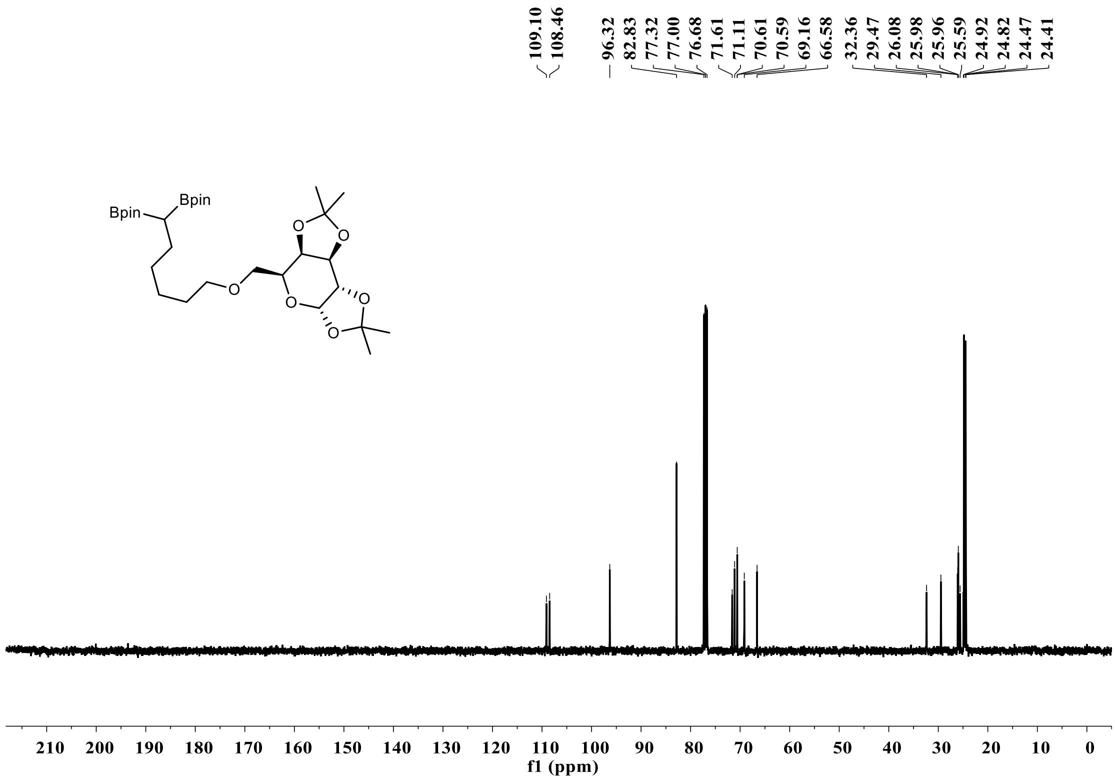

Supplementary Figure 8. ^{13}C NMR spectrum for **1p**

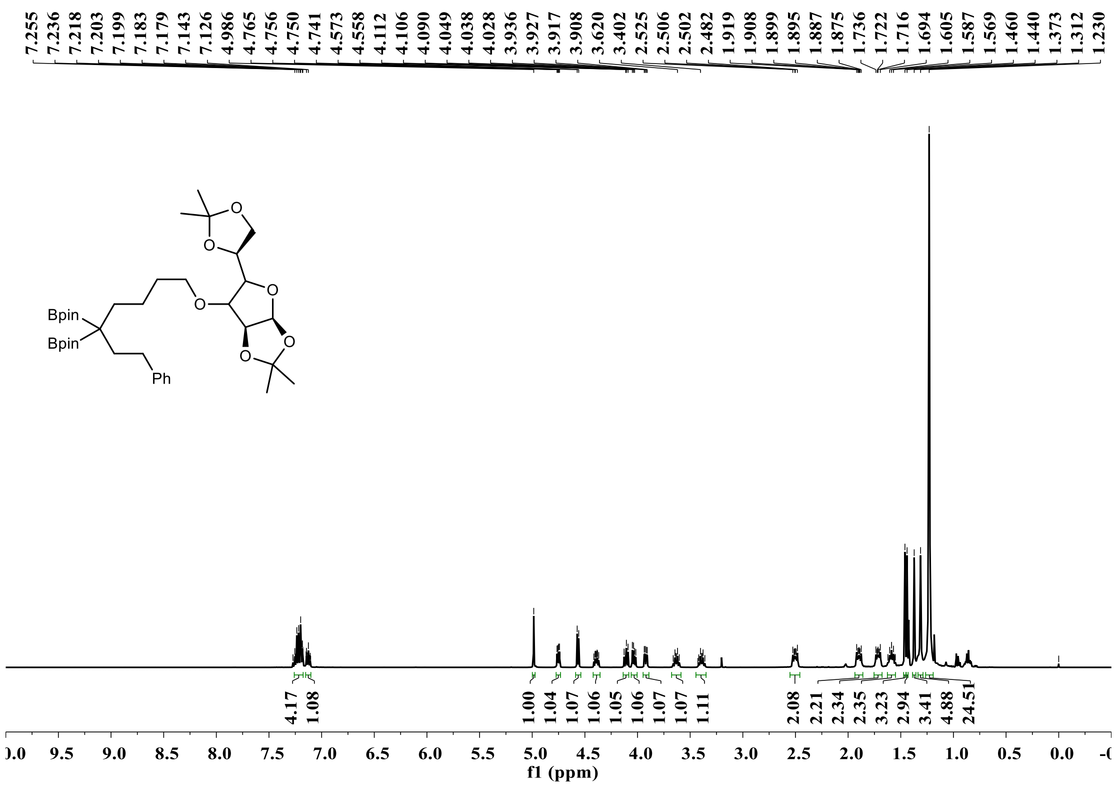

Supplementary Figure 9. ^{1}H NMR spectrum for **1q**

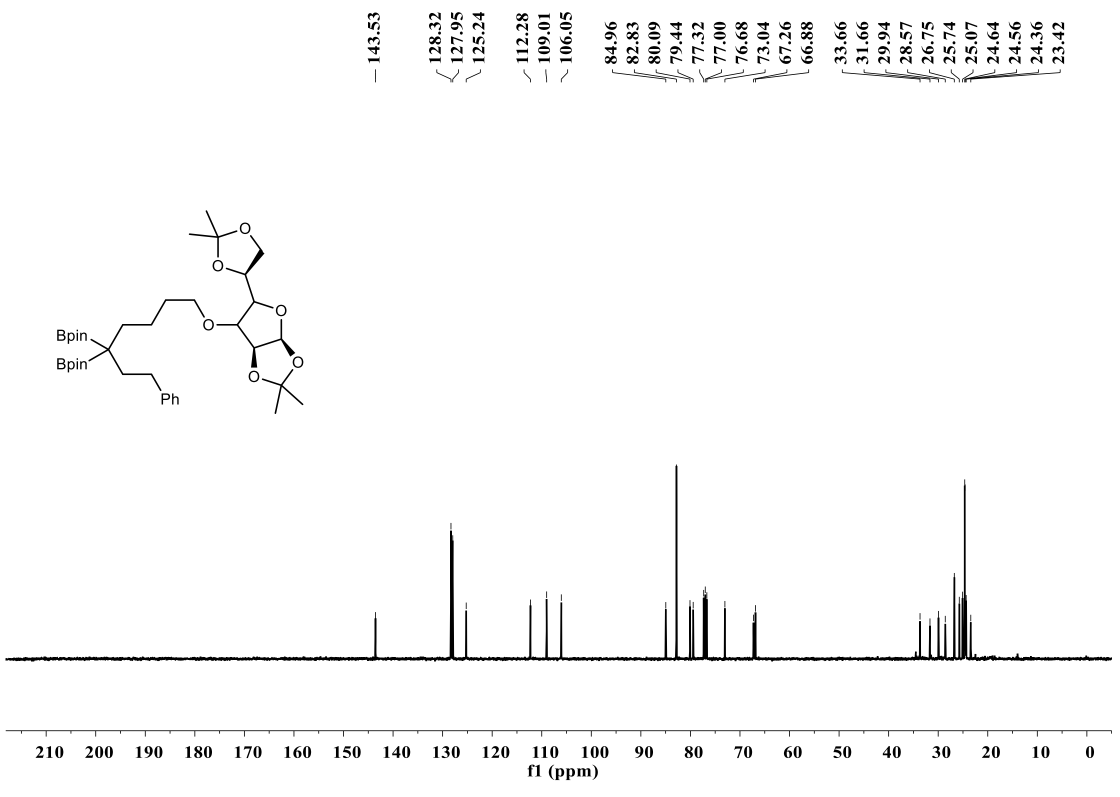

Supplementary Figure 10. ^{13}C NMR spectrum for **1q**

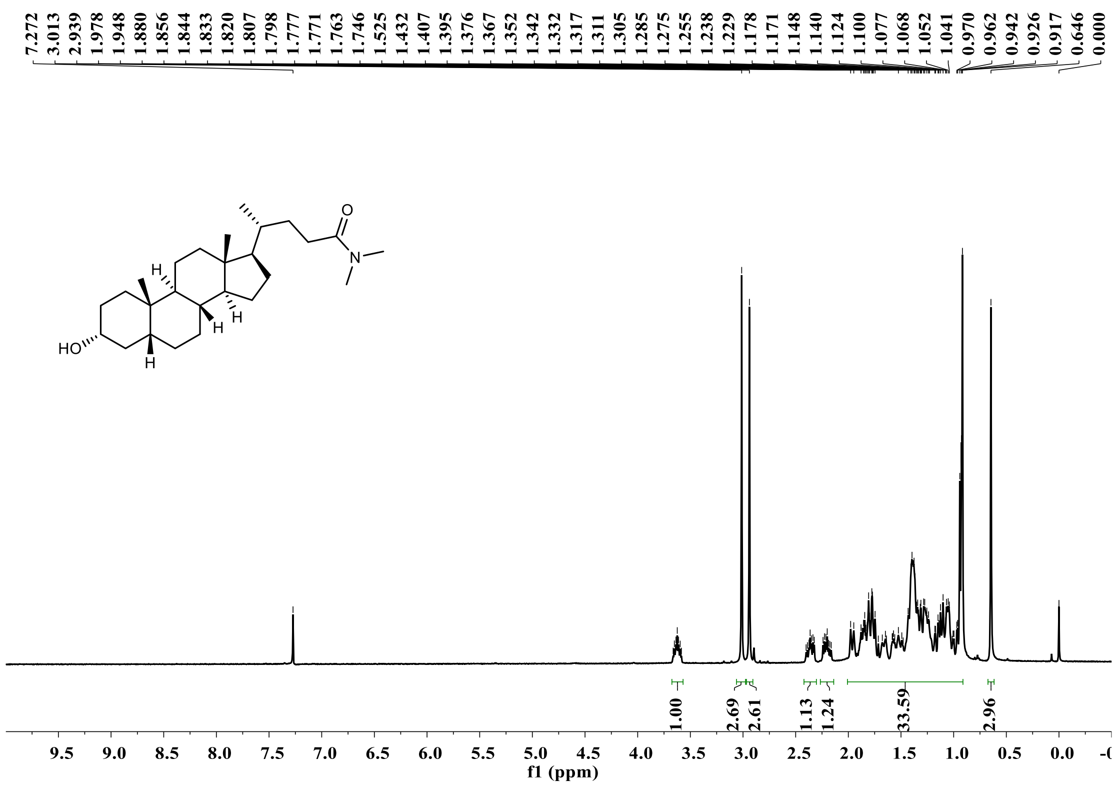

Supplementary Figure 11. ^{1}H NMR spectrum

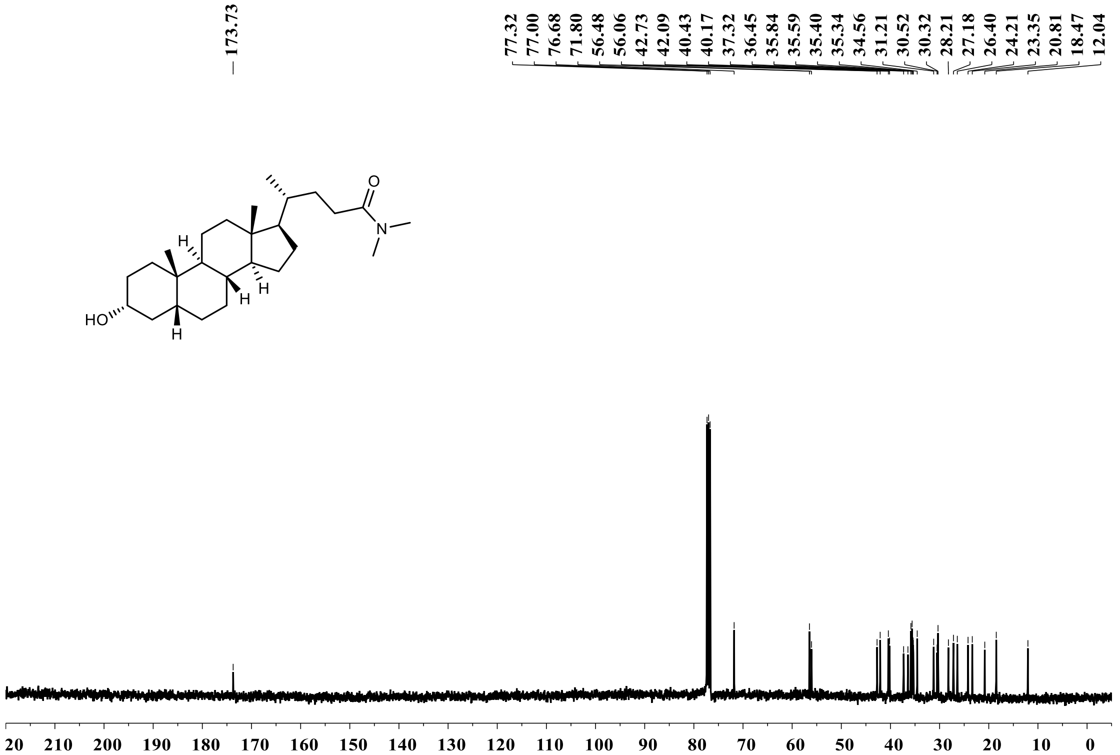

Supplementary Figure 12. ^{13}C NMR spectrum

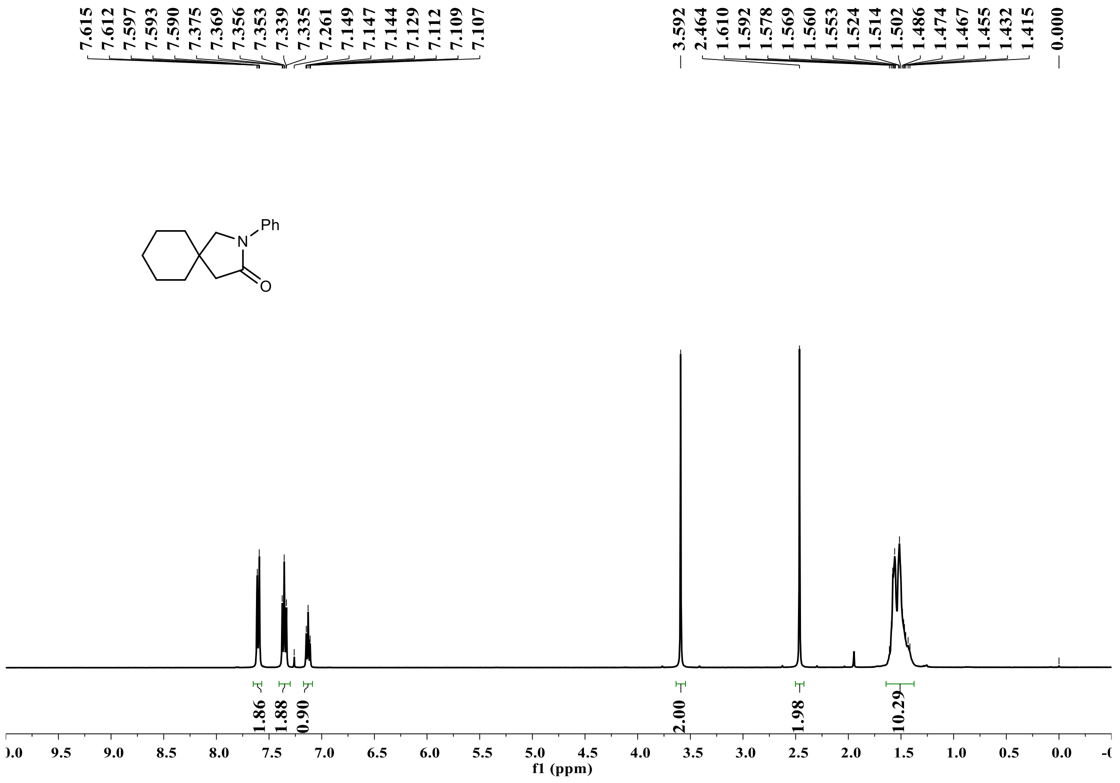

Supplementary Figure 13. ^{1}H NMR spectrum

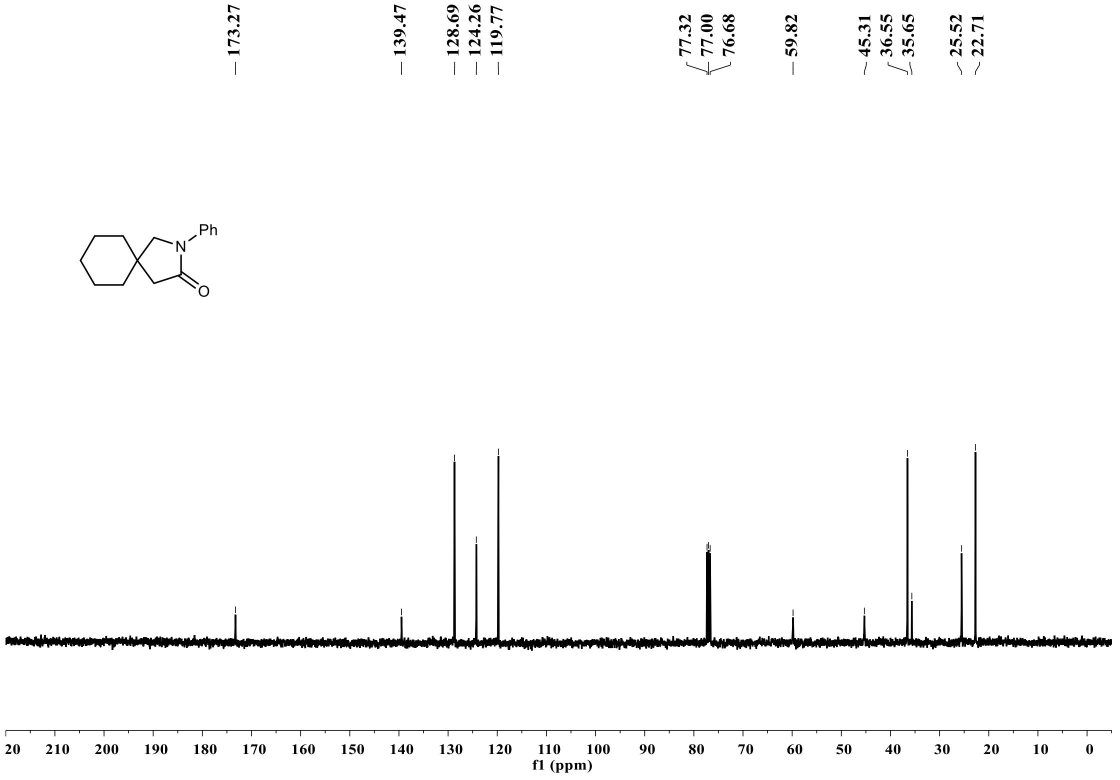

Supplementary Figure 14. ^{13}C NMR spectrum

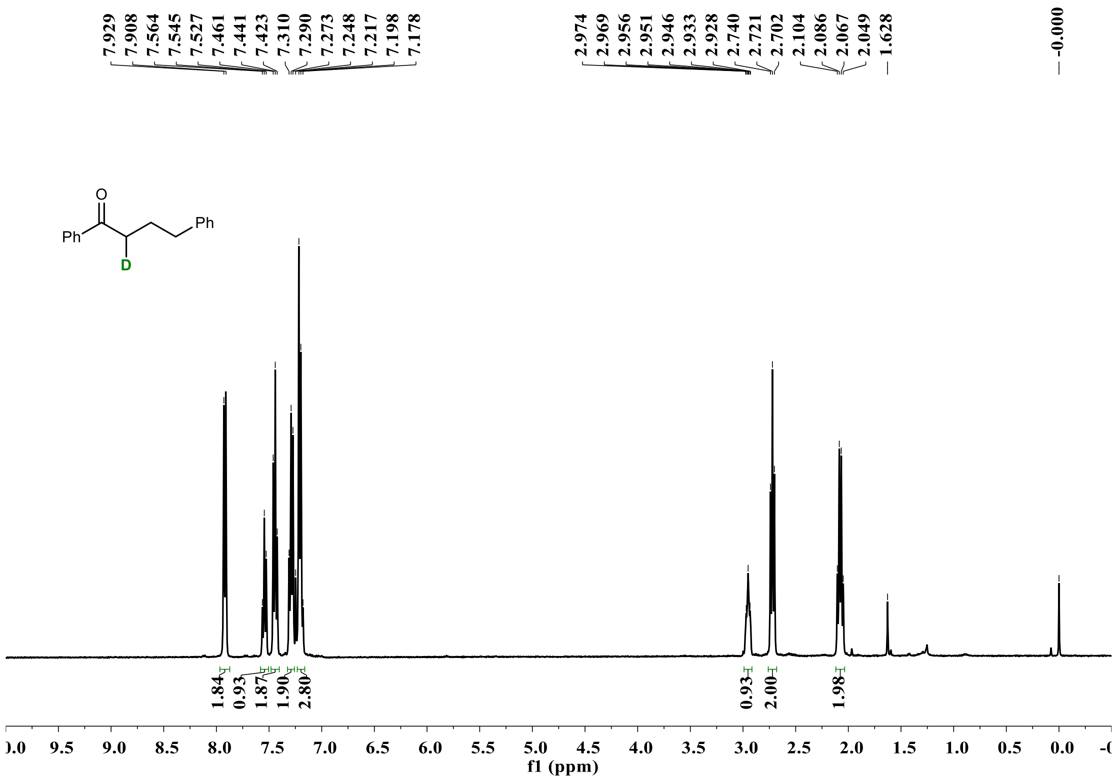

Supplementary Figure 15. ^{1}H NMR spectrum for **D-K1**

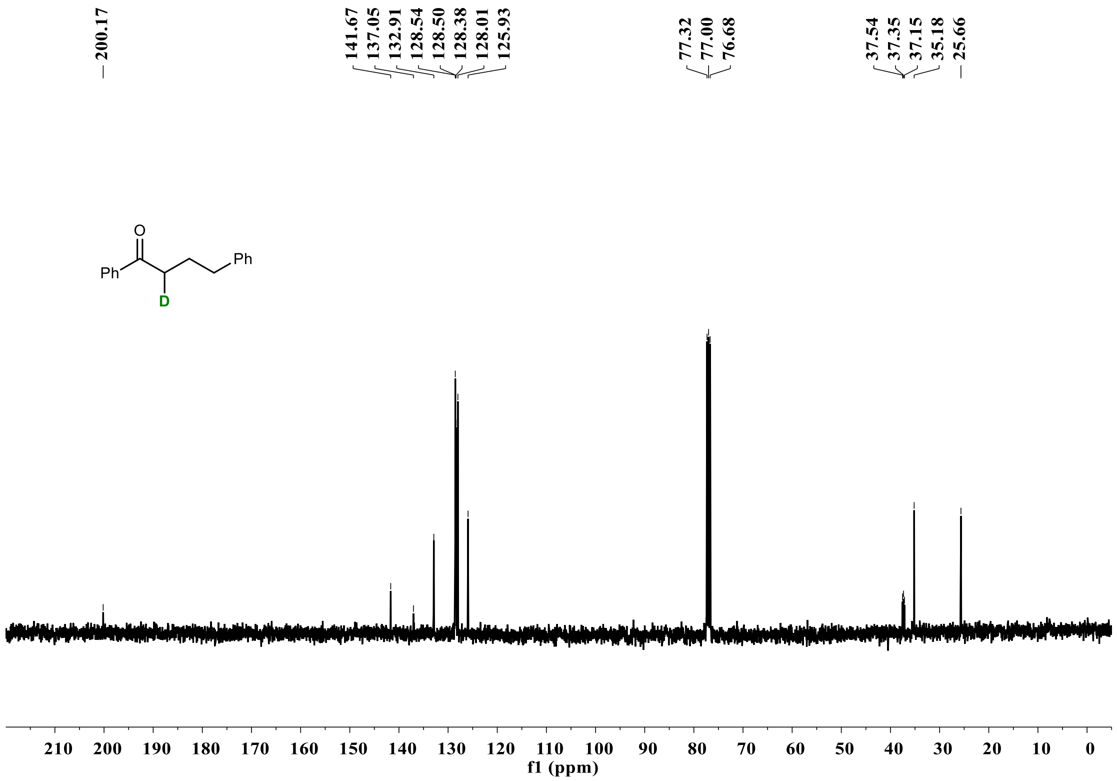

Supplementary Figure 16. ^{13}C NMR spectrum for **D-K1**

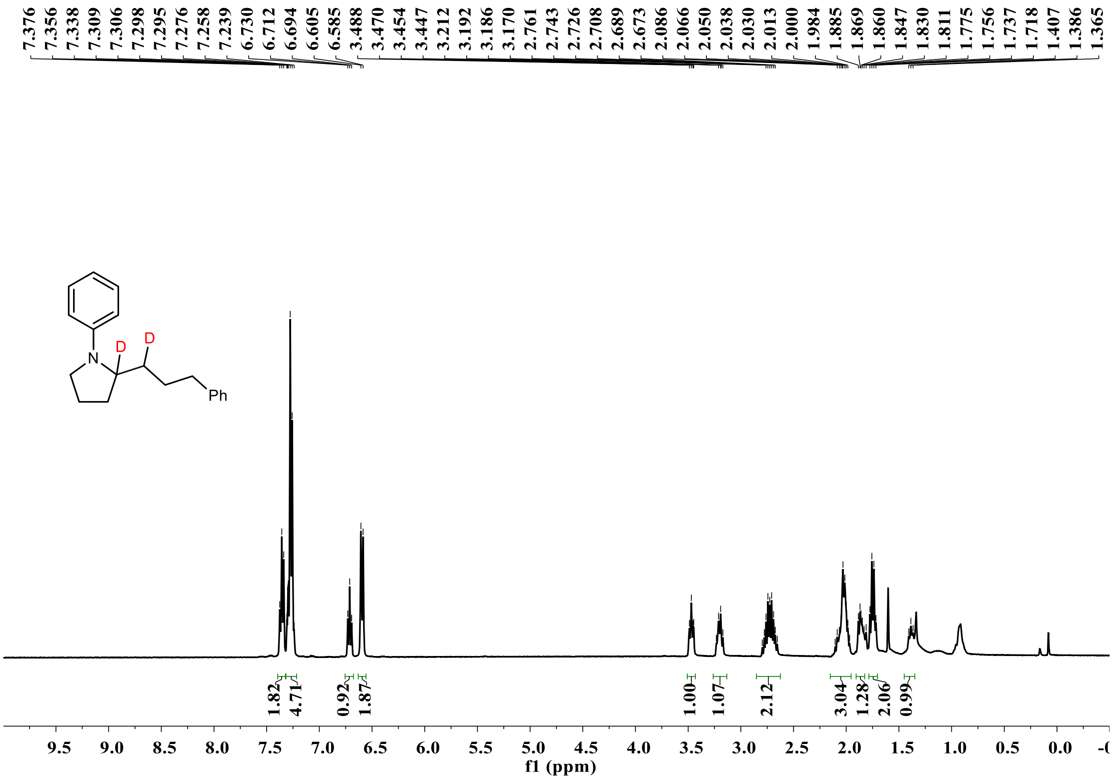

Supplementary Figure 17. ^{1}H NMR spectrum for **A1-*d***

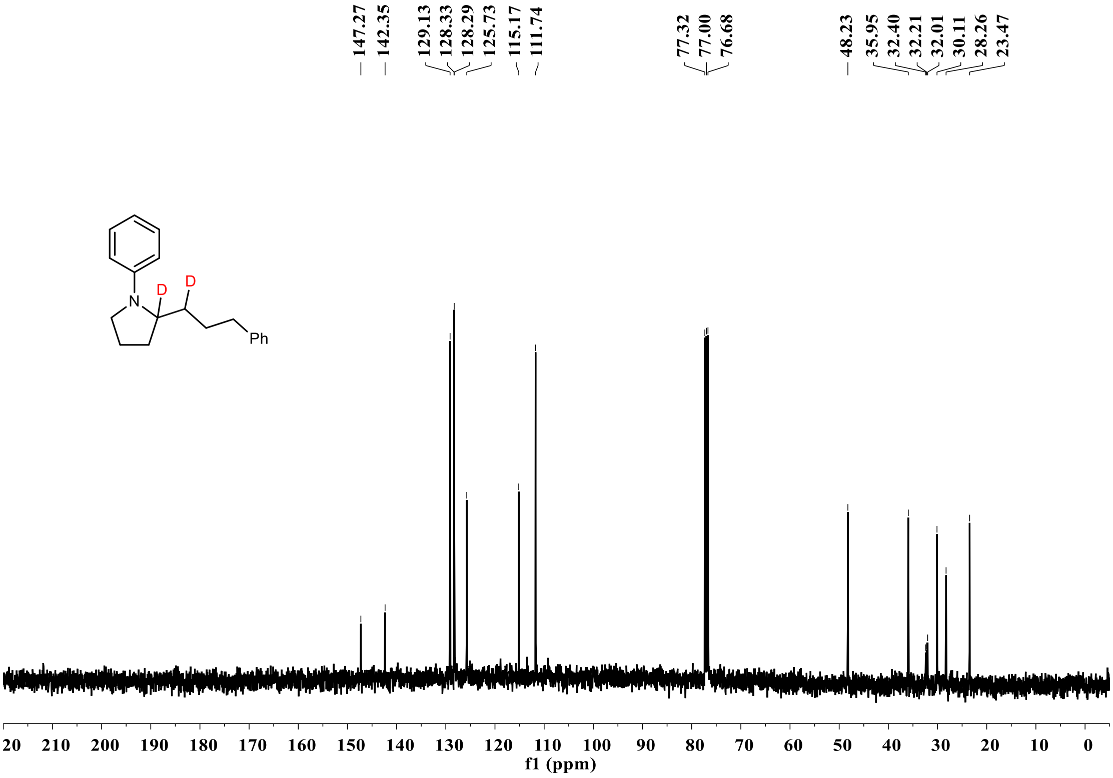

Supplementary Figure 18. ^{13}C NMR spectrum for **A1-*d***

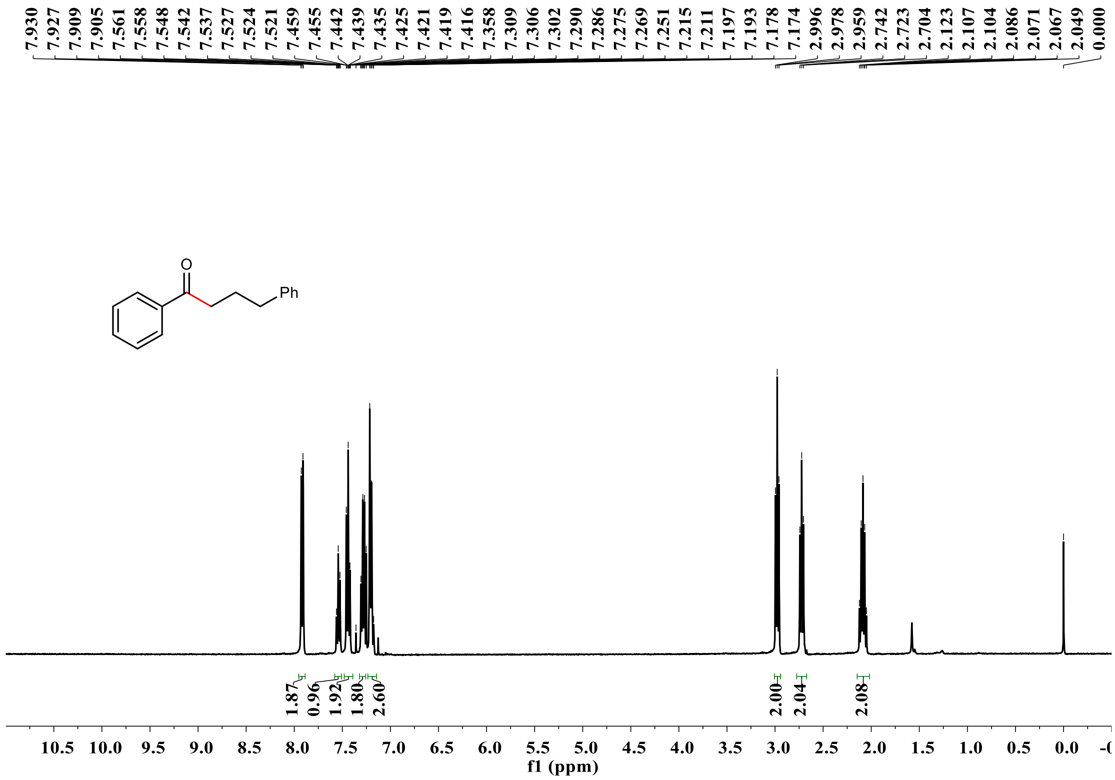

Supplementary Figure 19. ^{1}H NMR spectrum for **K1**

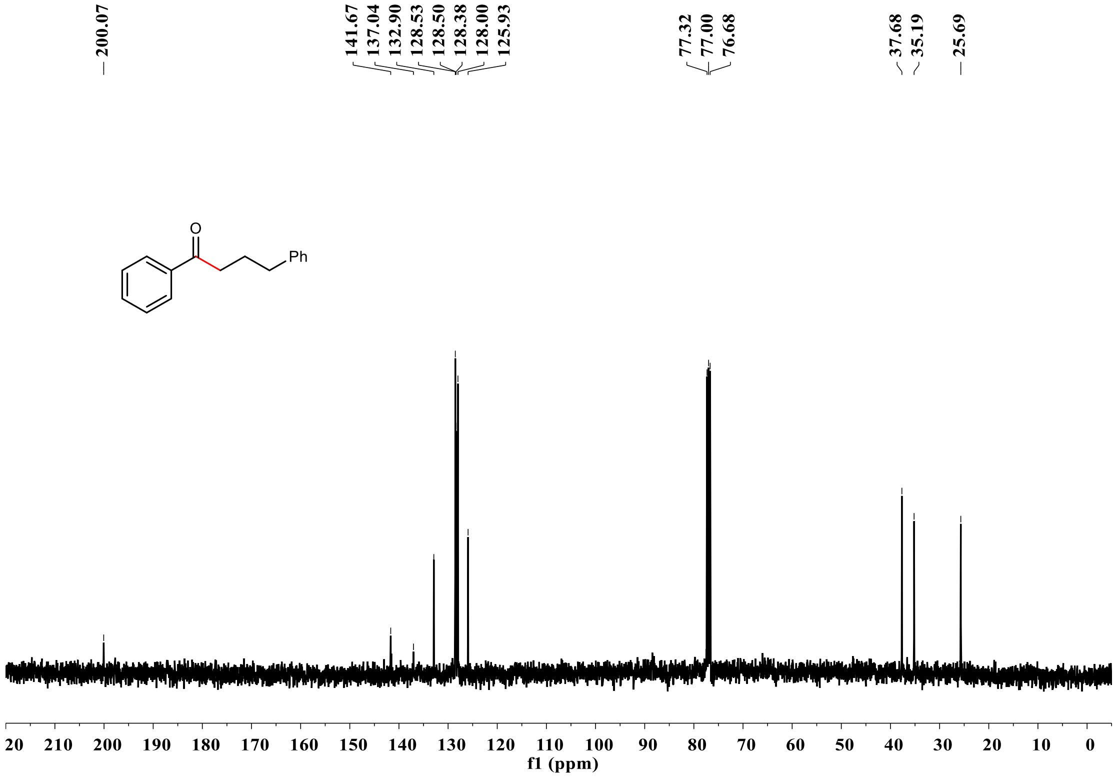

Supplementary Figure 20. ^{13}C NMR spectrum for **K1**

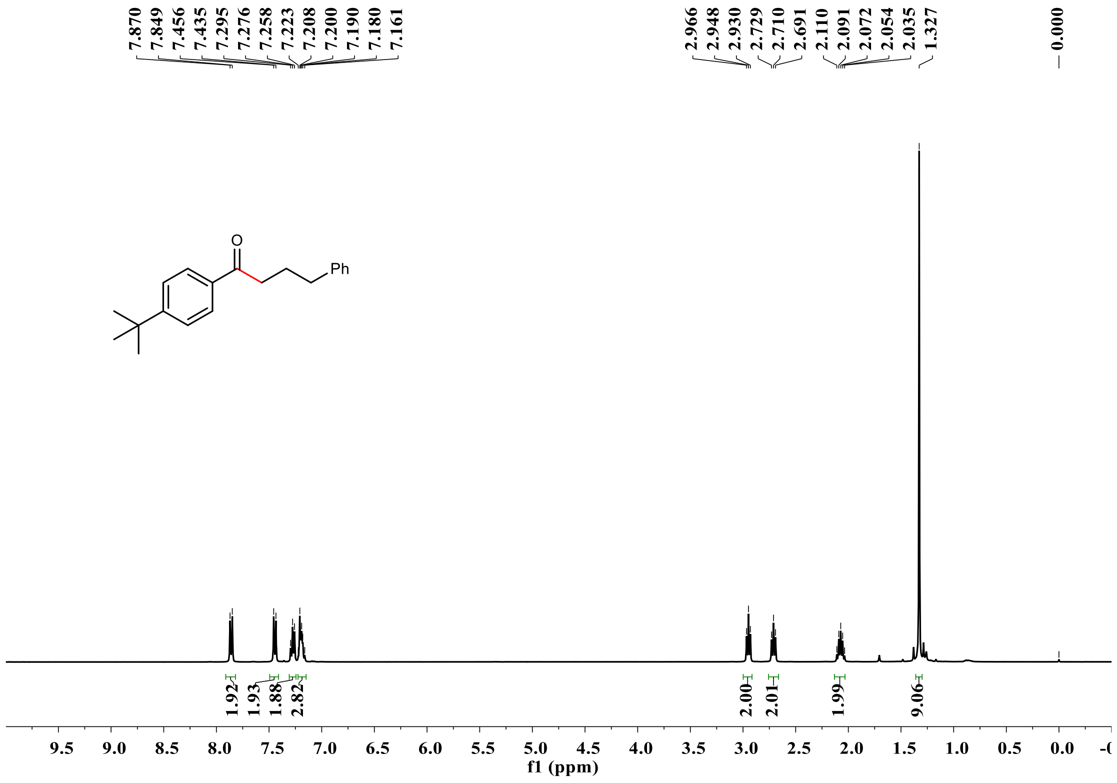

Supplementary Figure 21. ^{1}H NMR spectrum for **K2**

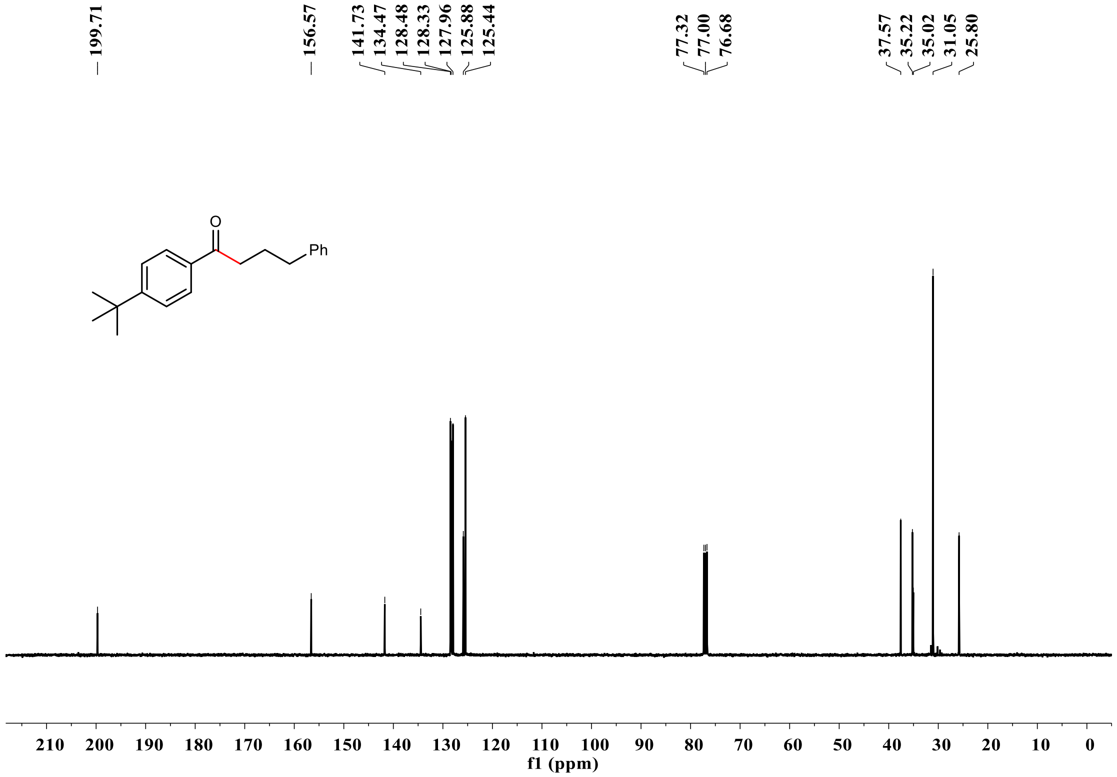

Supplementary Figure 22. ^{13}C NMR spectrum for **K2**

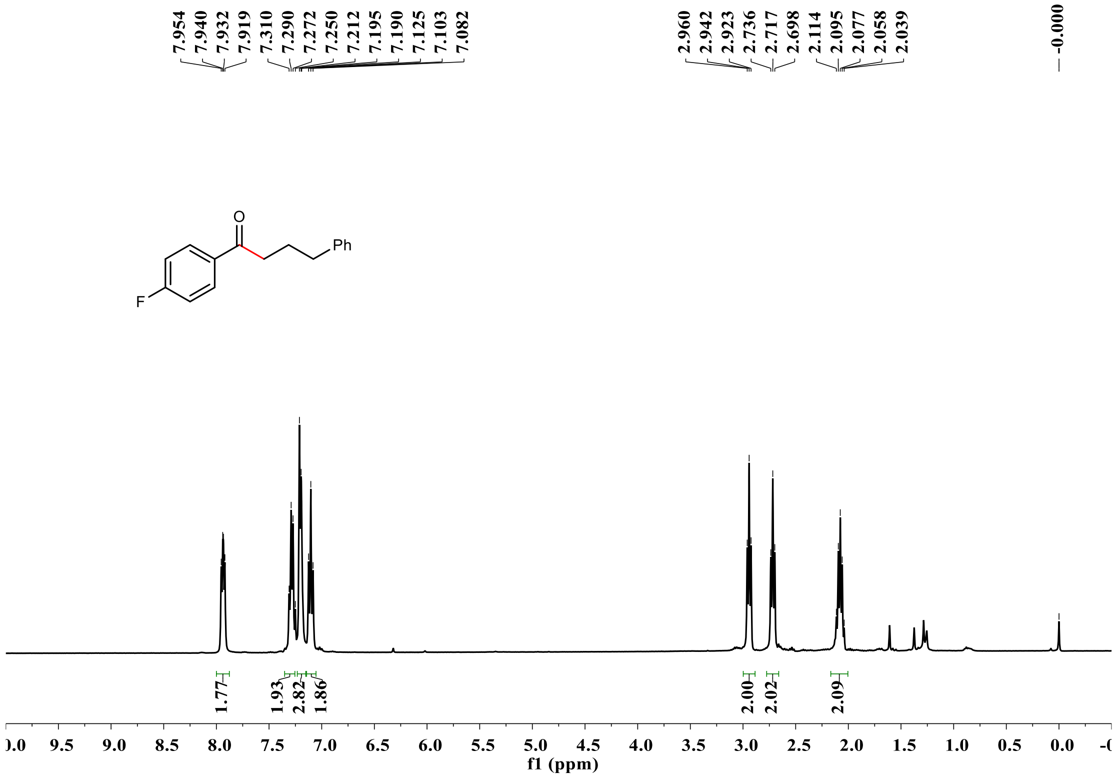

Supplementary Figure 23. ^{1}H NMR spectrum for **K3**

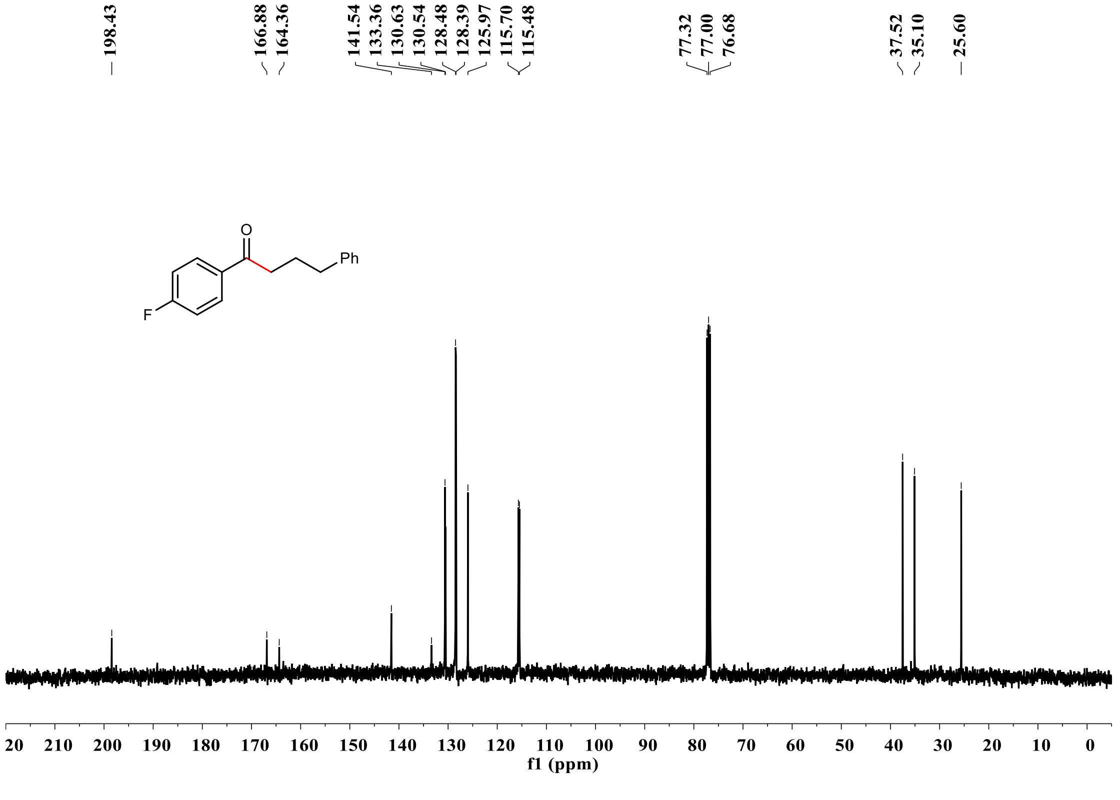

Supplementary Figure 24. ^{13}C NMR spectrum for **K3**

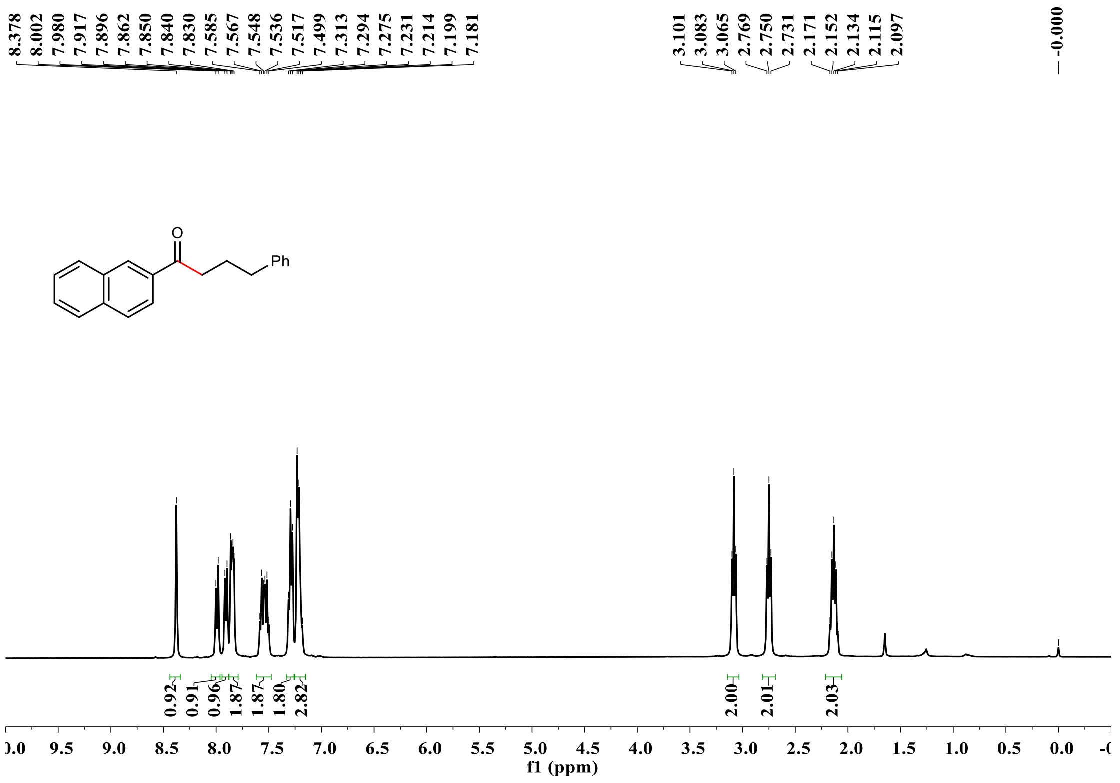

Supplementary Figure 25. ^{1}H NMR spectrum for **K4**

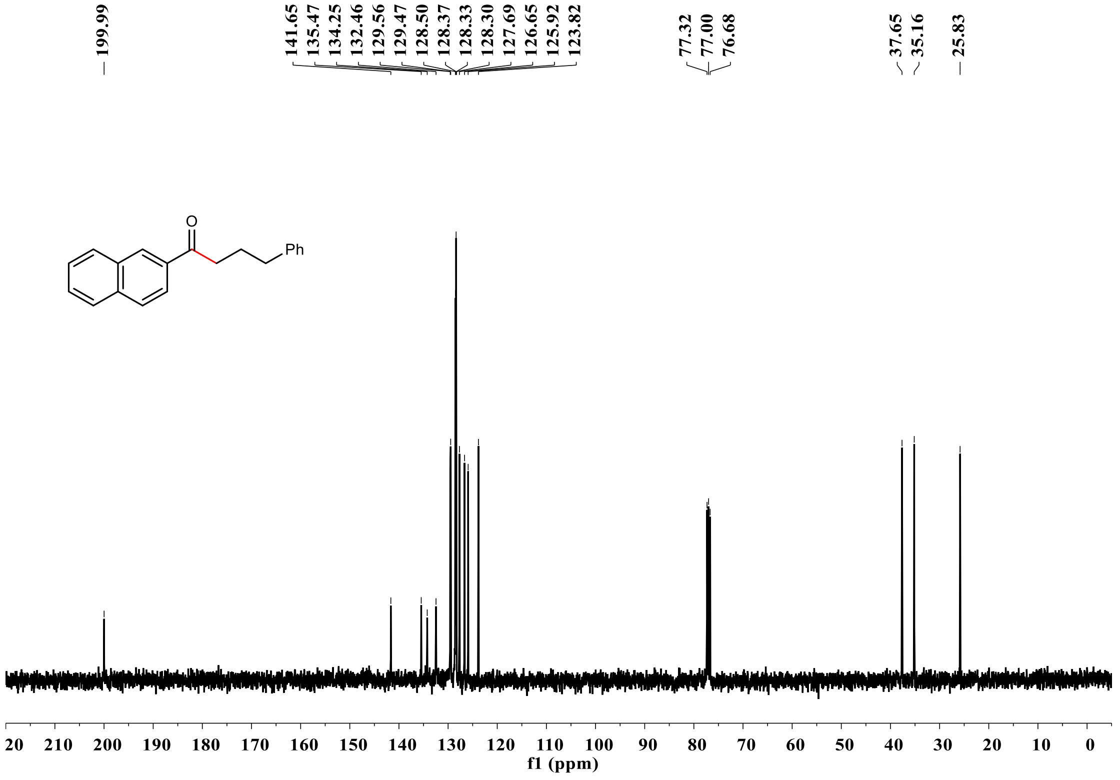

Supplementary Figure 26. ^{13}C NMR spectrum for **K4**

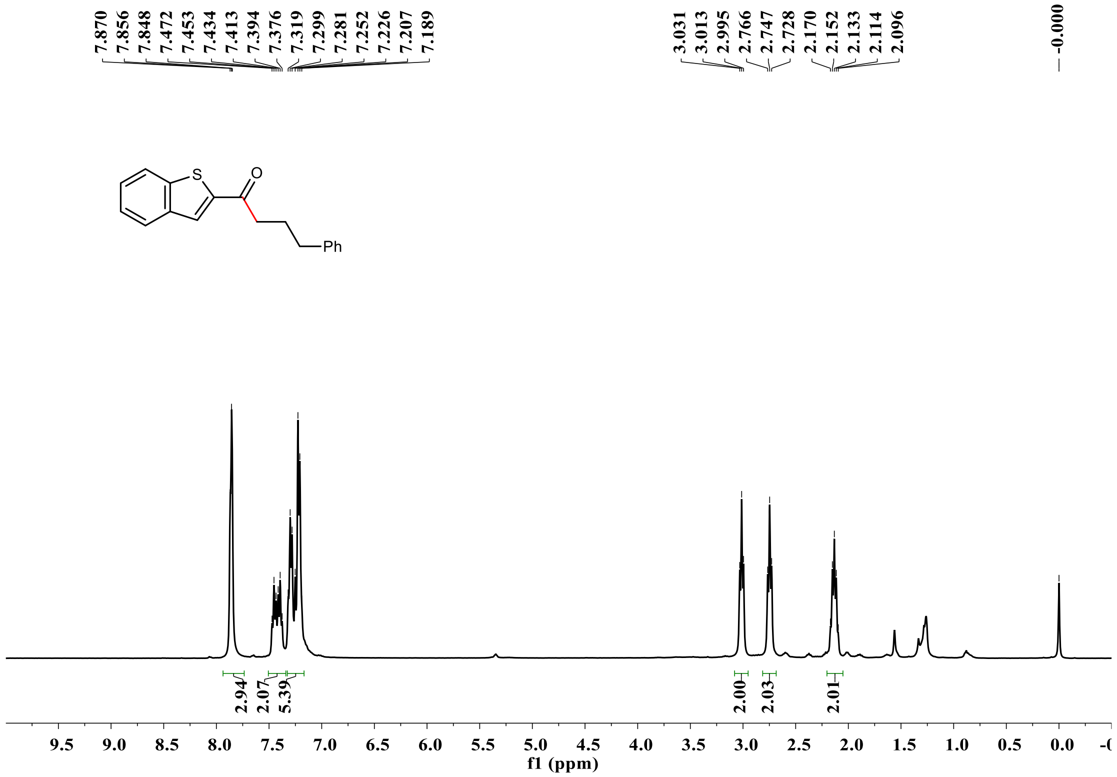

Supplementary Figure 27. ^{1}H NMR spectrum for **K5**

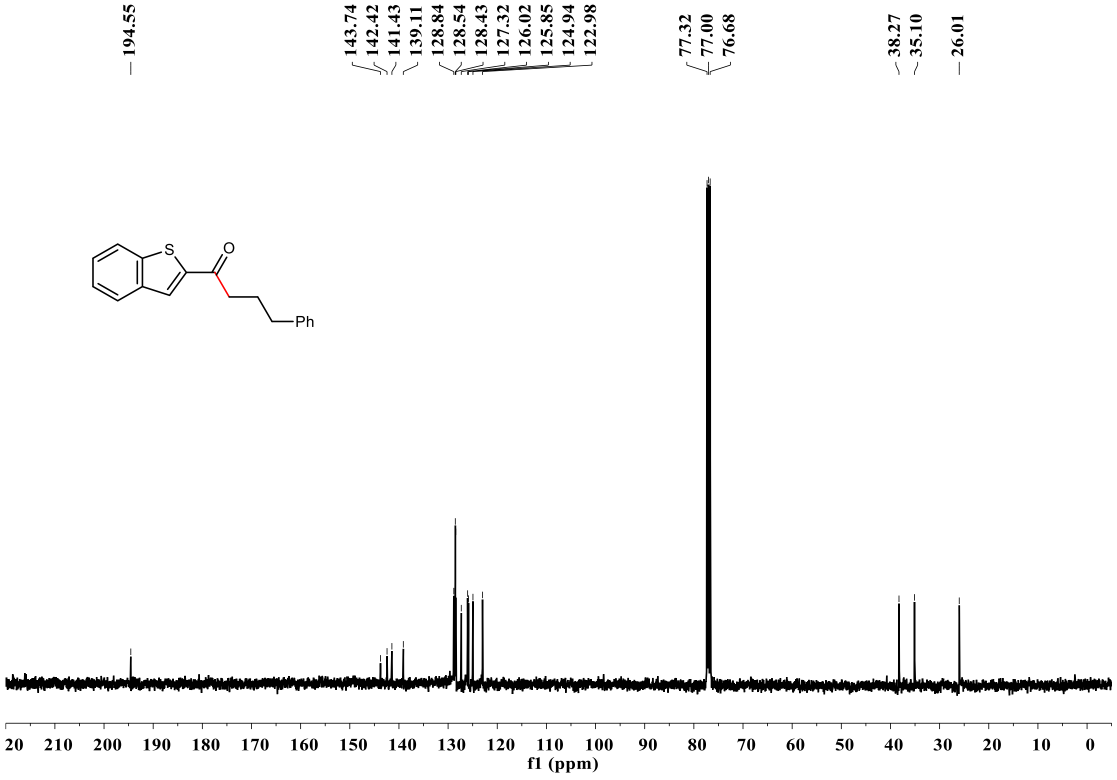

Supplementary Figure 28. ^{13}C NMR spectrum for **K5**

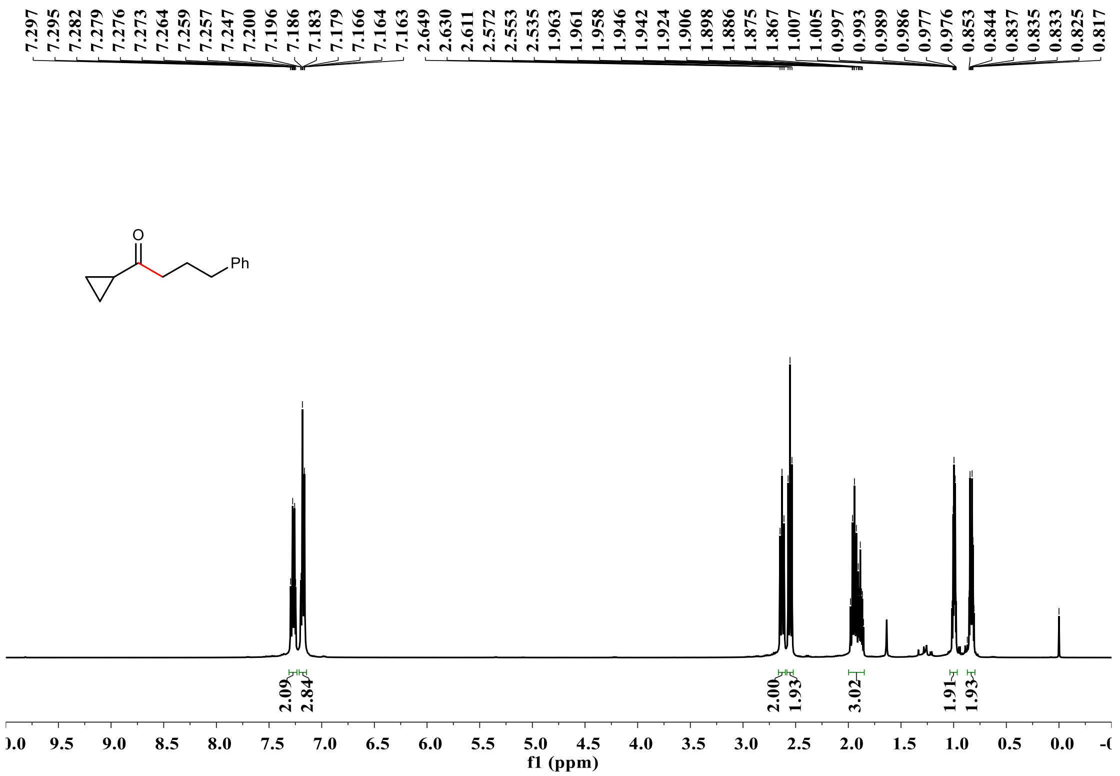

Supplementary Figure 29. ^{1}H NMR spectrum for **K6**

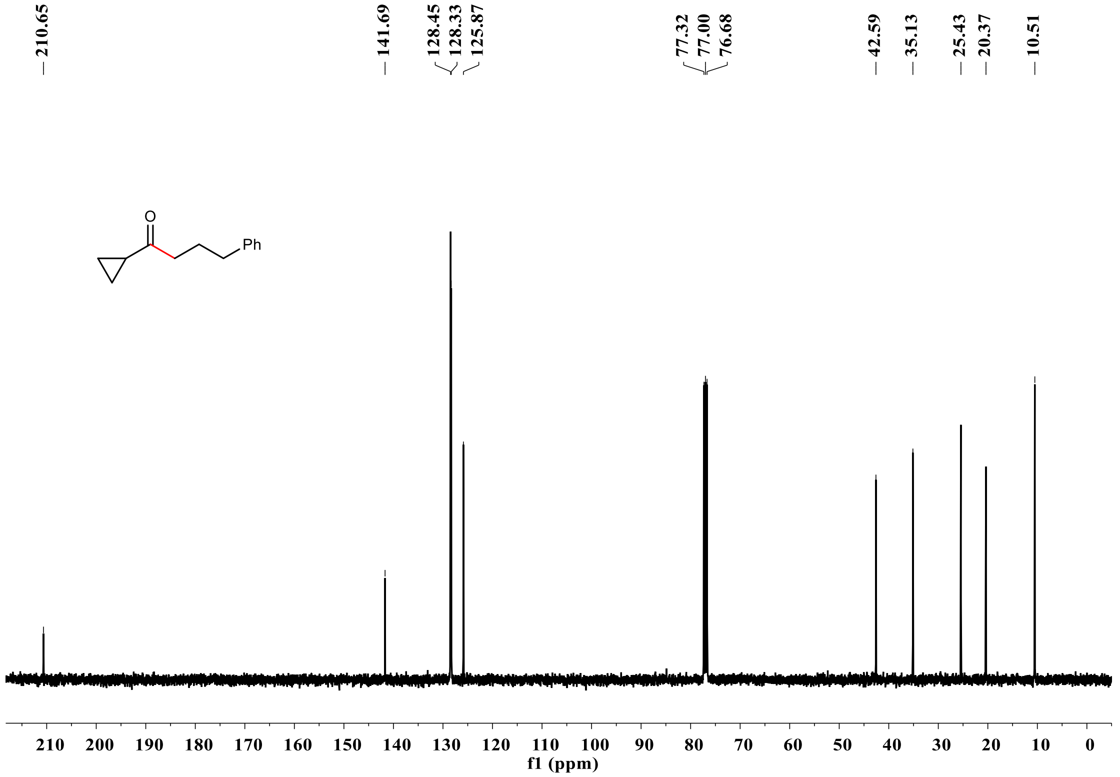

Supplementary Figure 30. ^{13}C NMR spectrum for **K6**

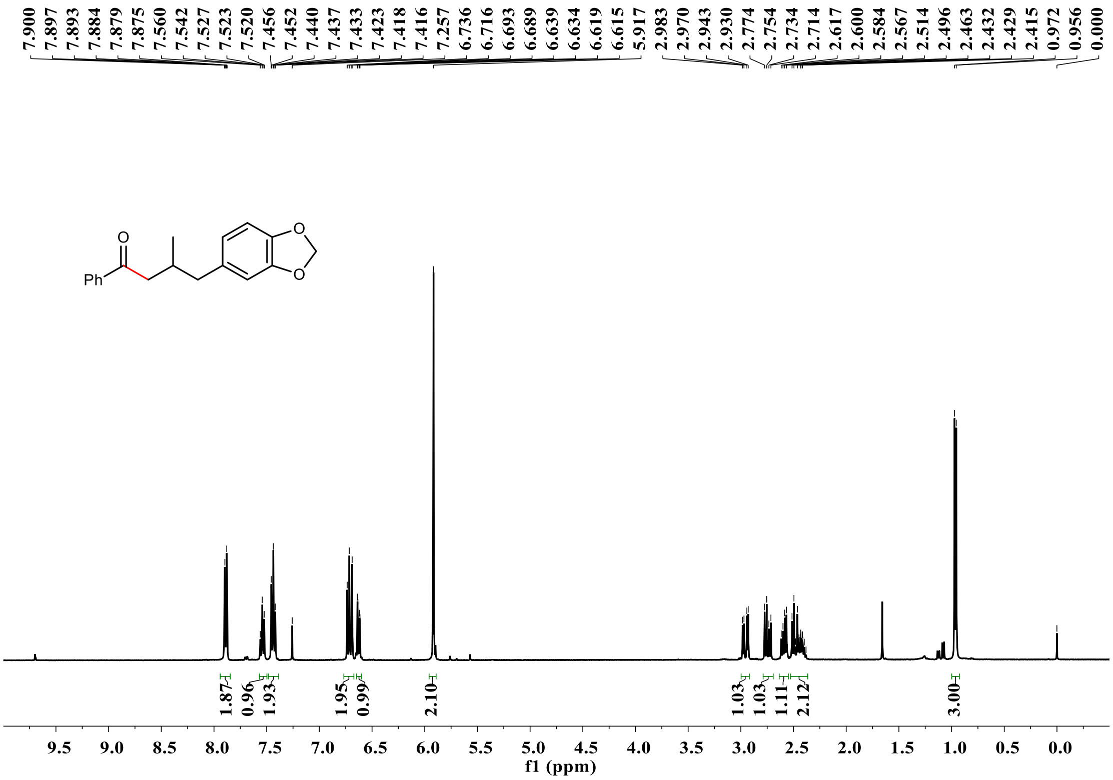

Supplementary Figure 31. ^{1}H NMR spectrum for **K7**

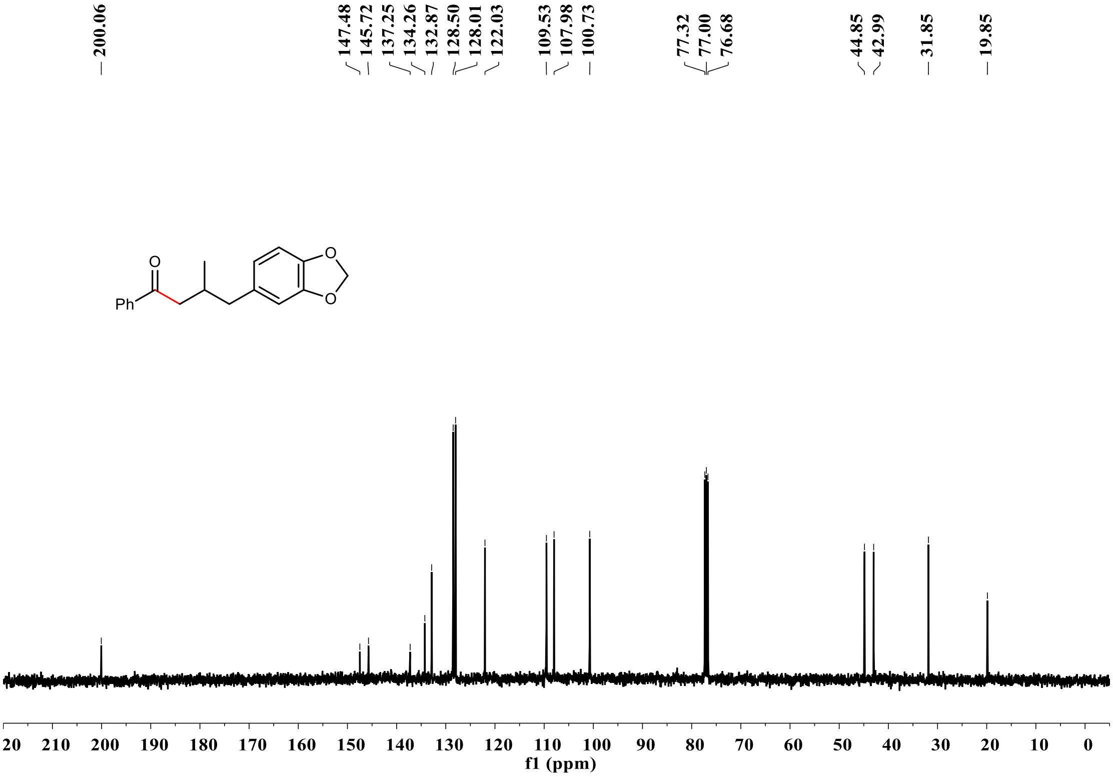

Supplementary Figure 32. ^{13}C NMR spectrum for **K7**

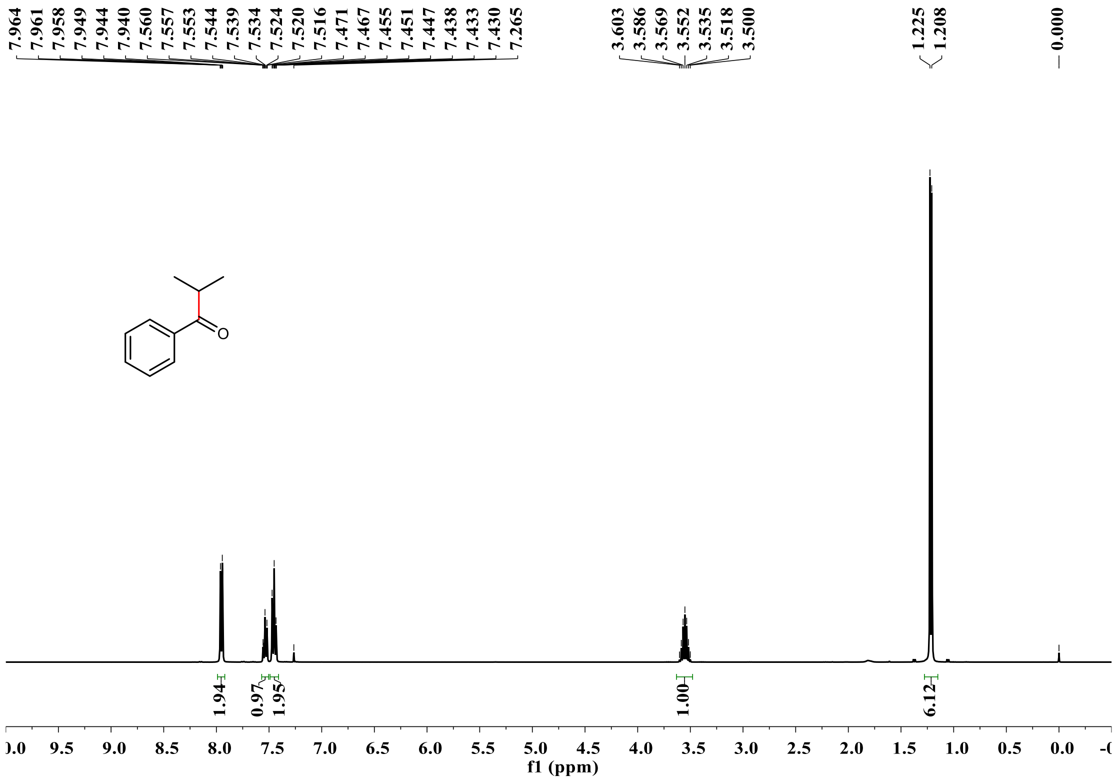

Supplementary Figure 33. ^{1}H NMR spectrum for **K8**

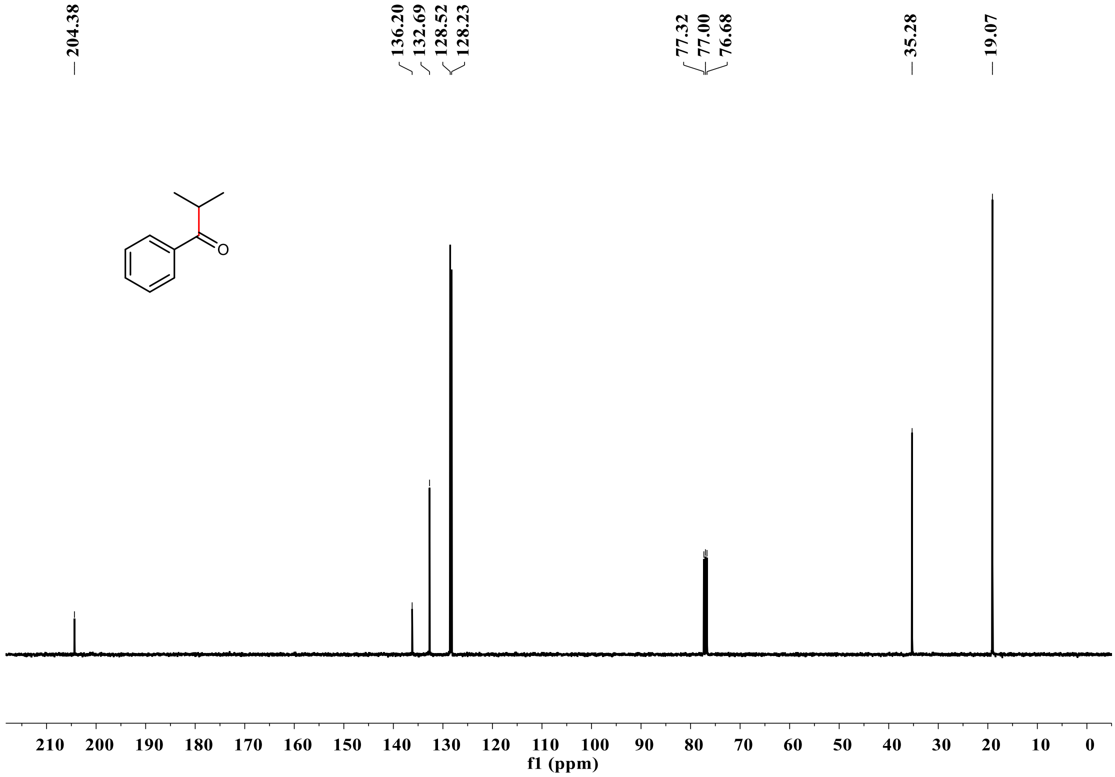

Supplementary Figure 34. ^{13}C NMR spectrum for **K8**

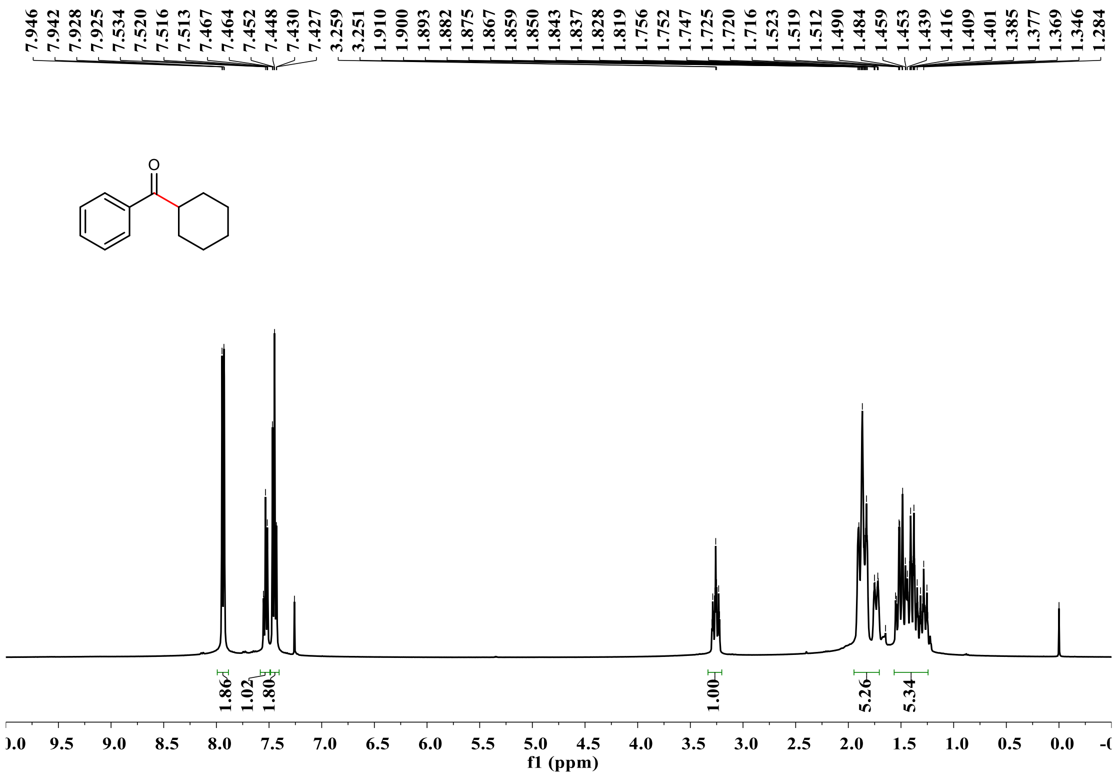

Supplementary Figure 35. ^{1}H NMR spectrum for **K9**

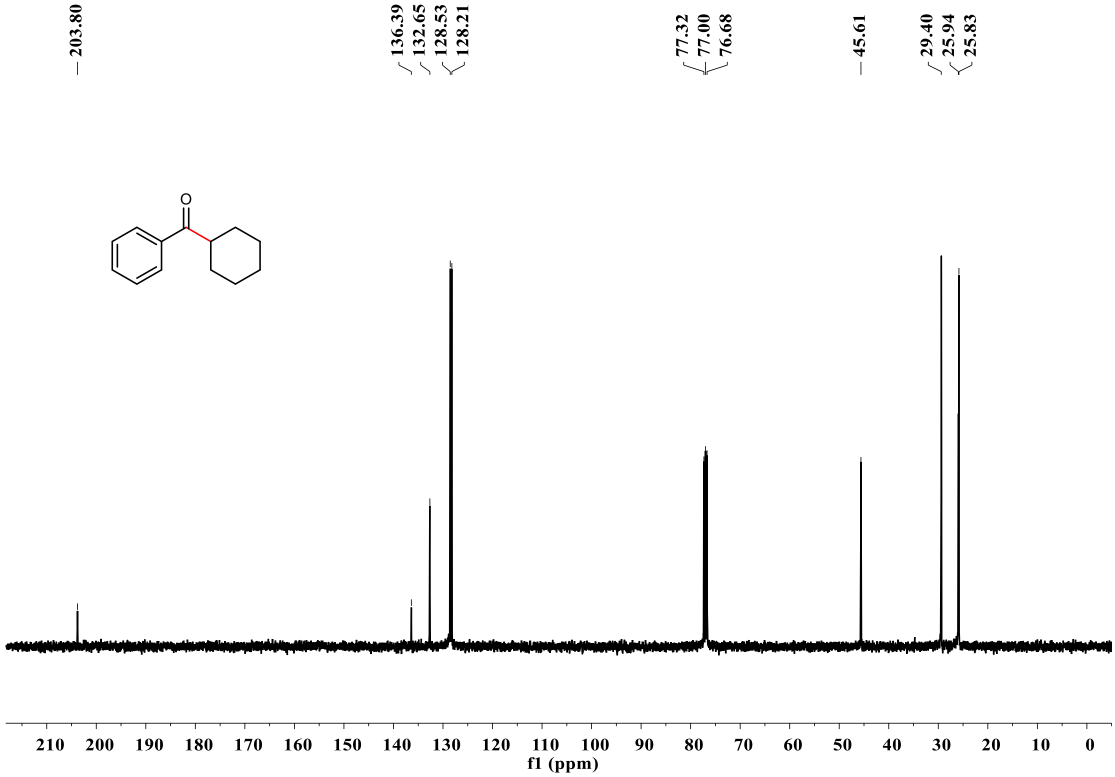

Supplementary Figure 36. ^{13}C NMR spectrum for **K9**

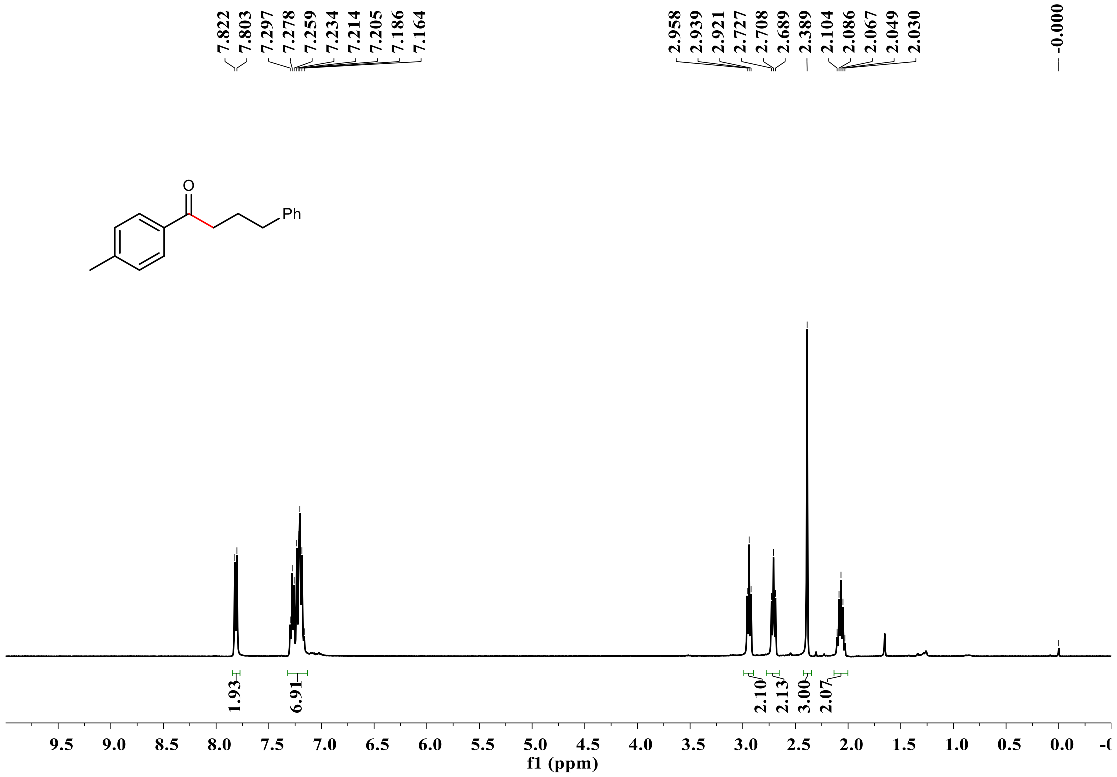

Supplementary Figure 37. ^{1}H NMR spectrum for **K10**

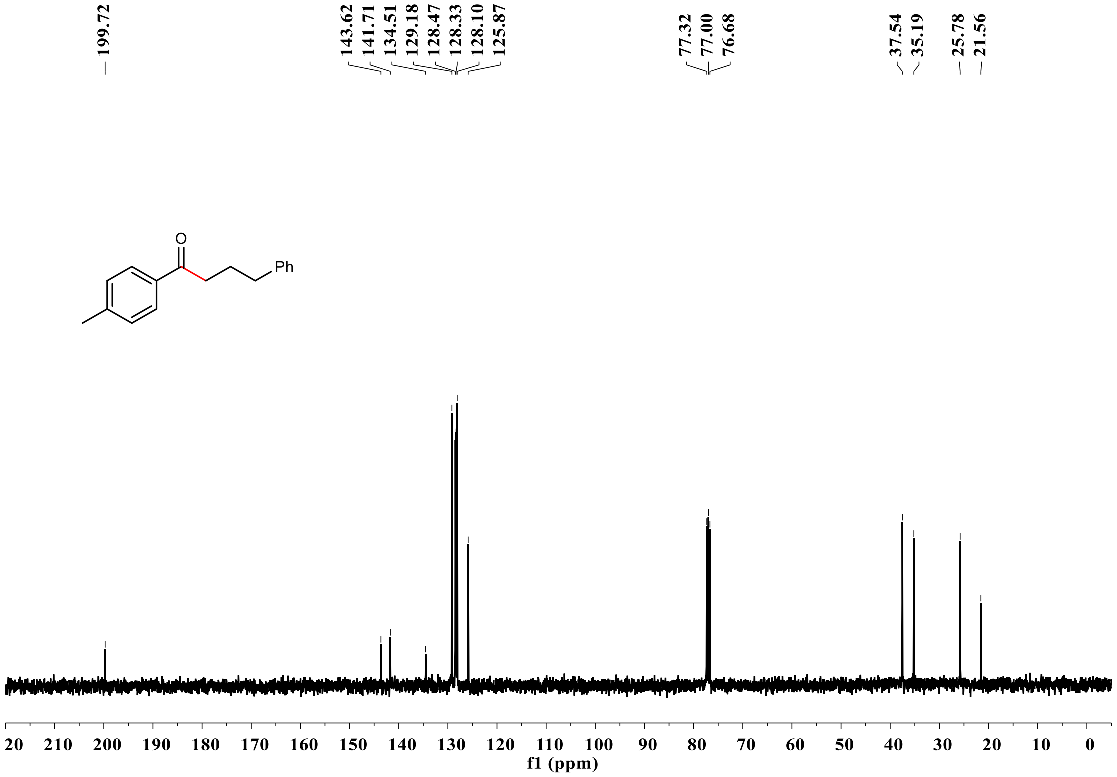

Supplementary Figure 38. ^{13}C NMR spectrum for **K10**

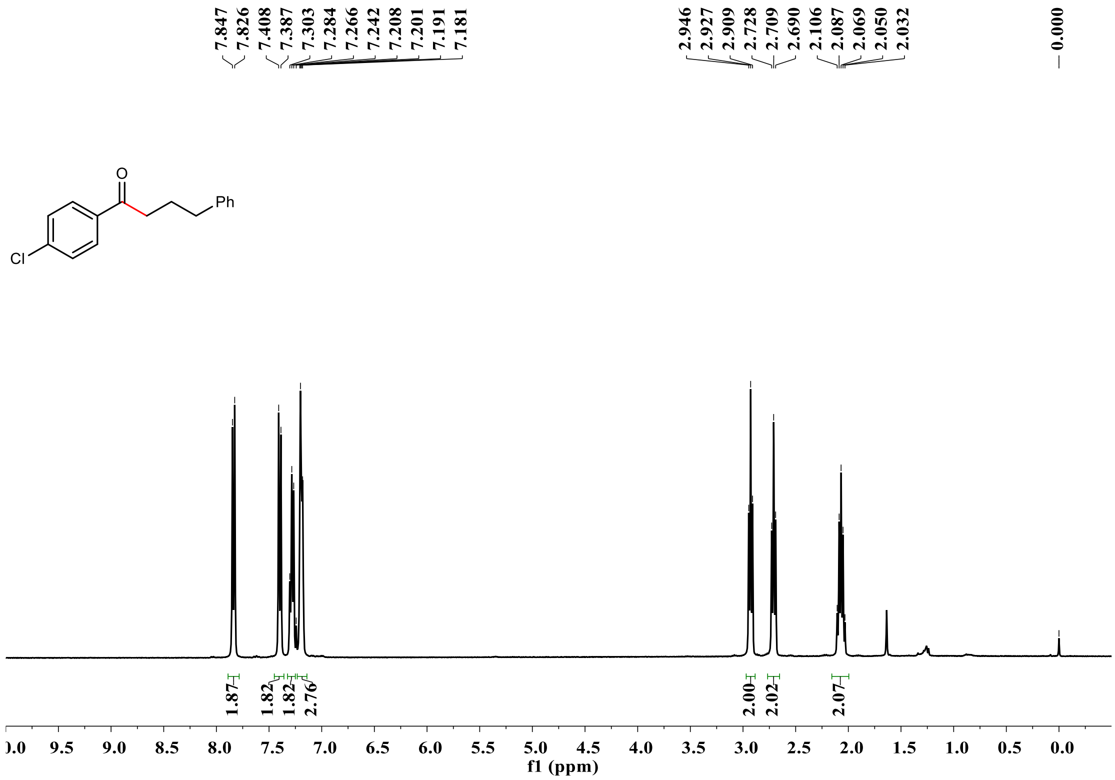

Supplementary Figure 39. ^{1}H NMR spectrum for **K11**

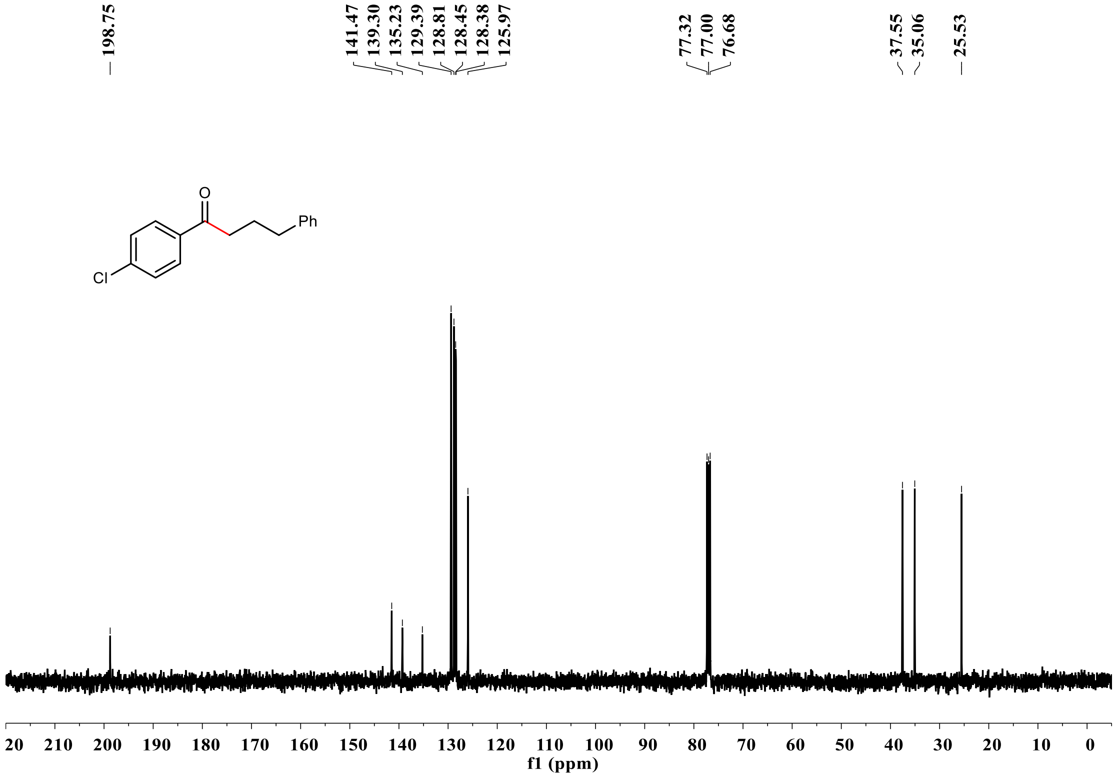

Supplementary Figure 40. ^{13}C NMR spectrum for **K11**

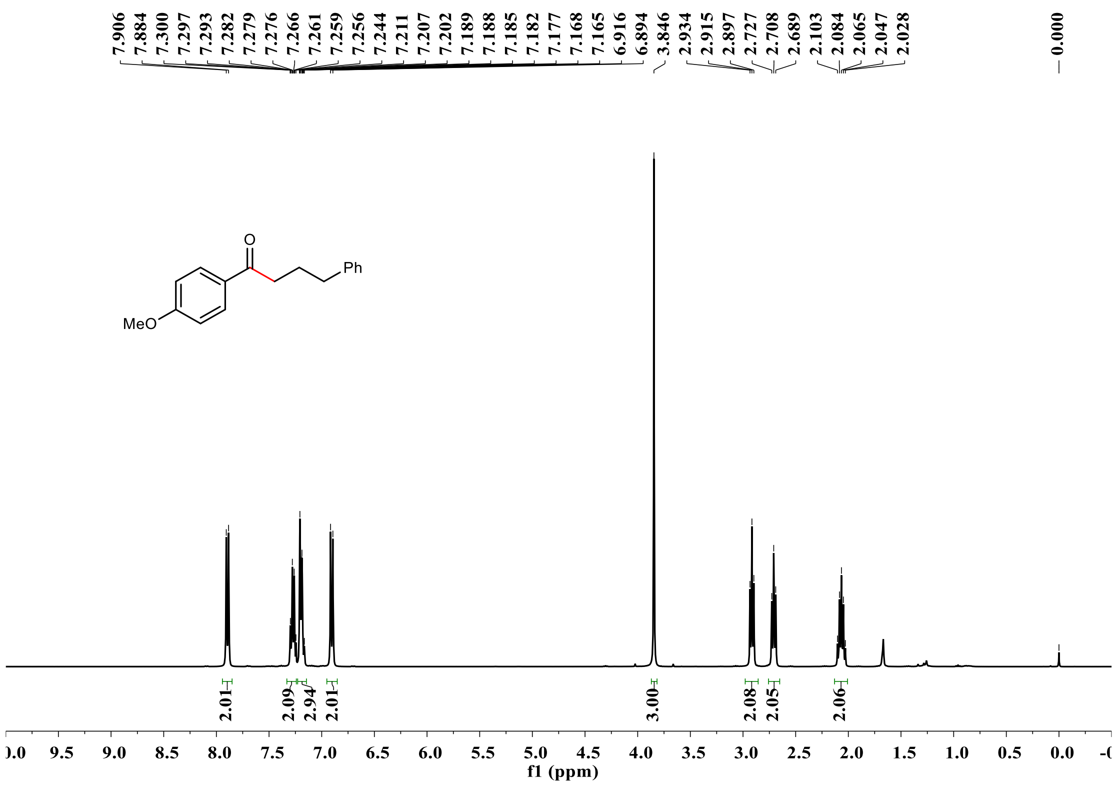

Supplementary Figure 41. ^{1}H NMR spectrum for **K12**

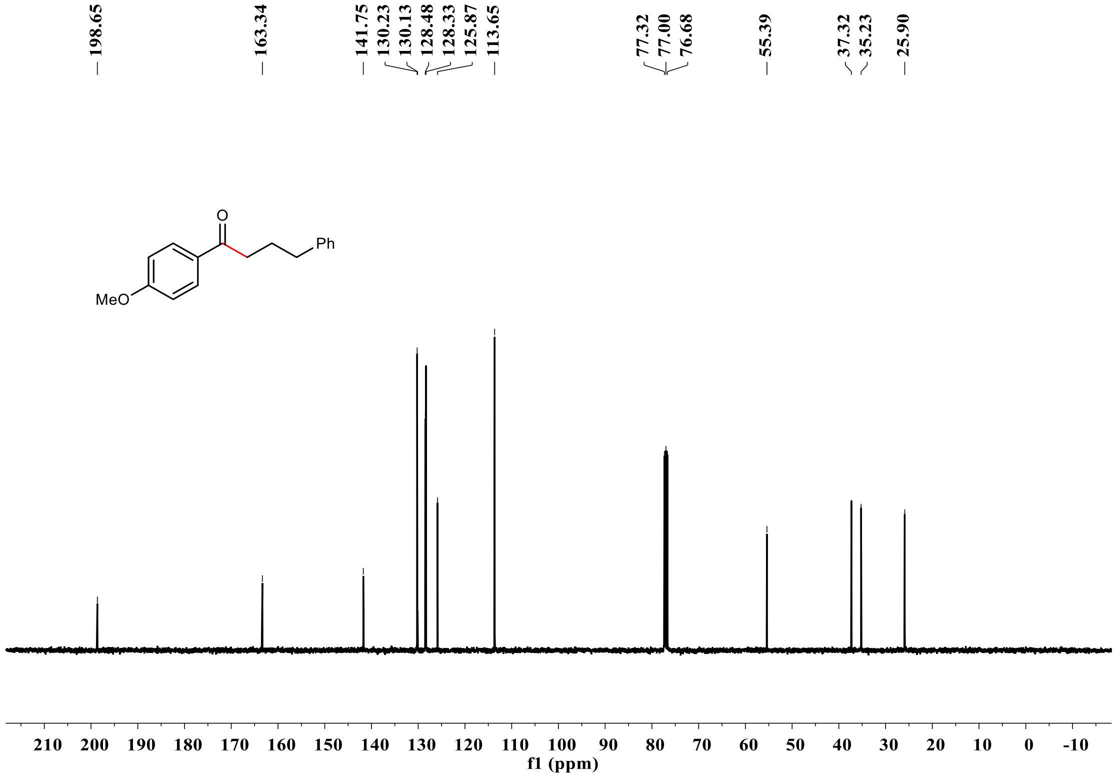

Supplementary Figure 42. ^{13}C NMR spectrum for **K12**

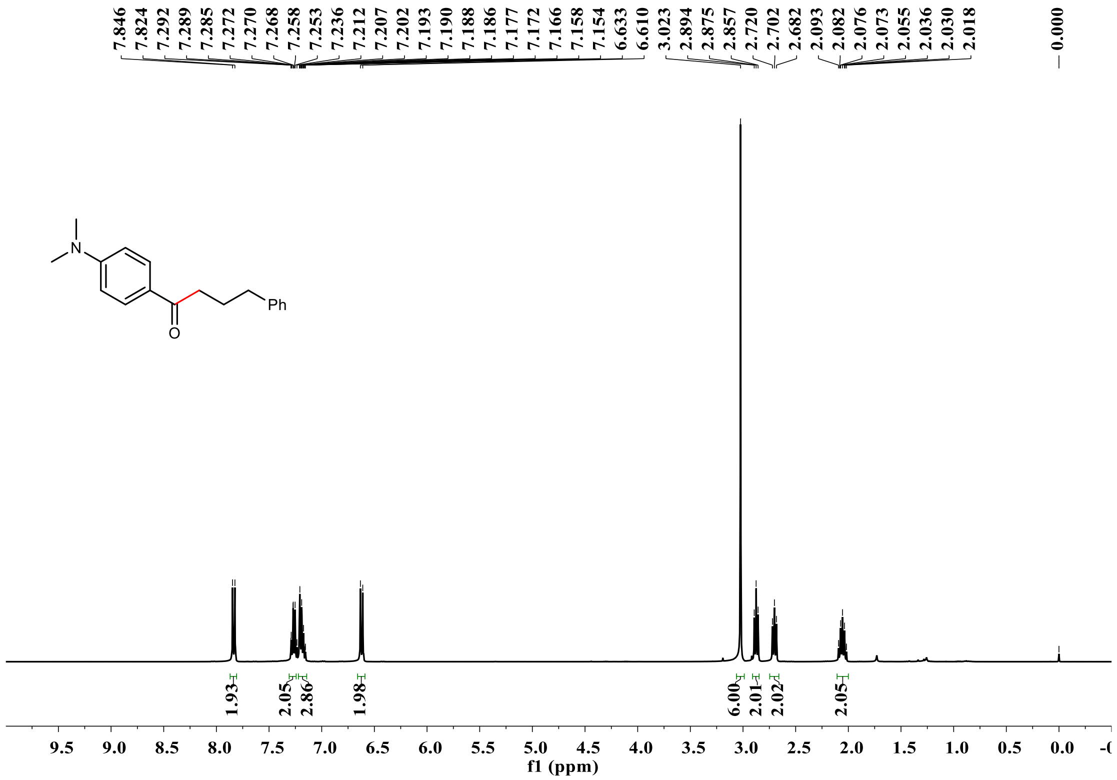

Supplementary Figure 43. ^{1}H NMR spectrum for **K13**

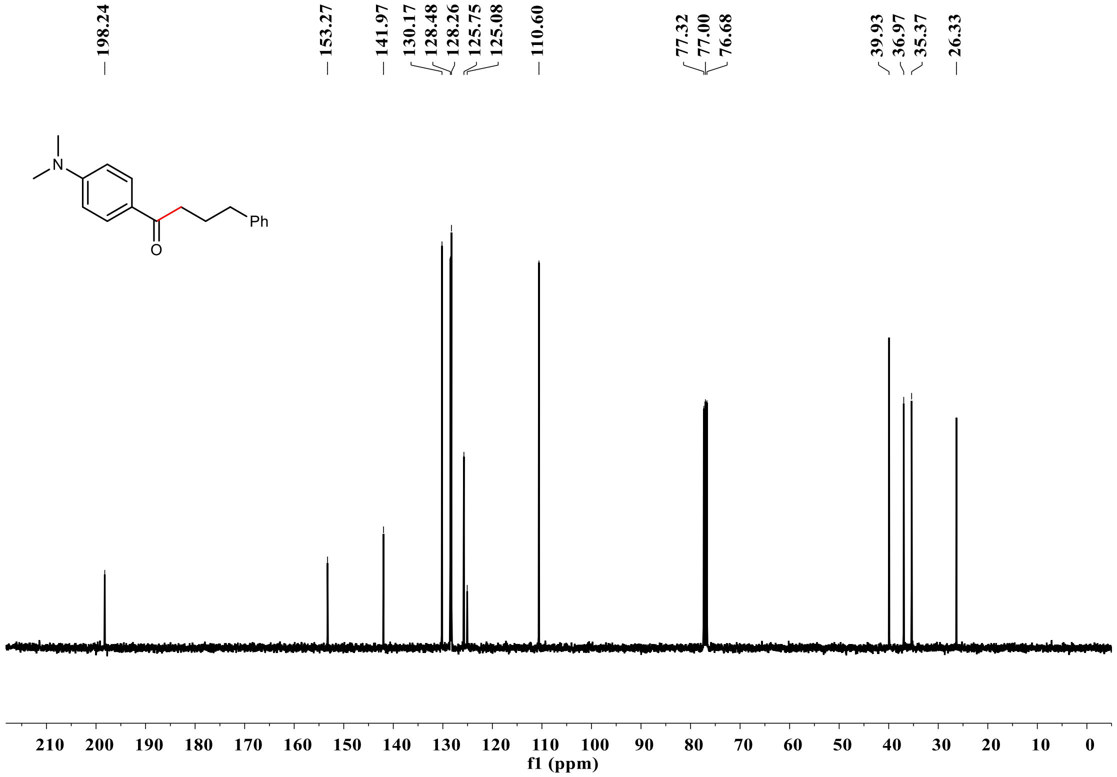

Supplementary Figure 44. ^{13}C NMR spectrum for **K13**

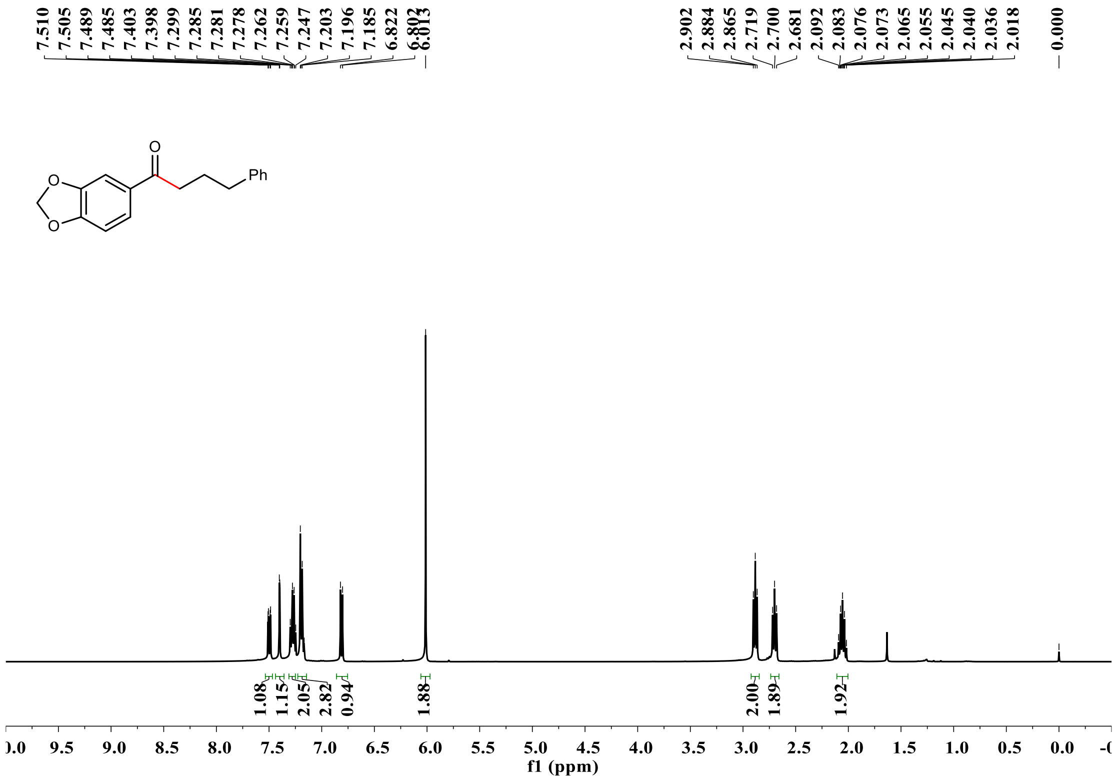

Supplementary Figure 45. ^{1}H NMR spectrum for **K14**

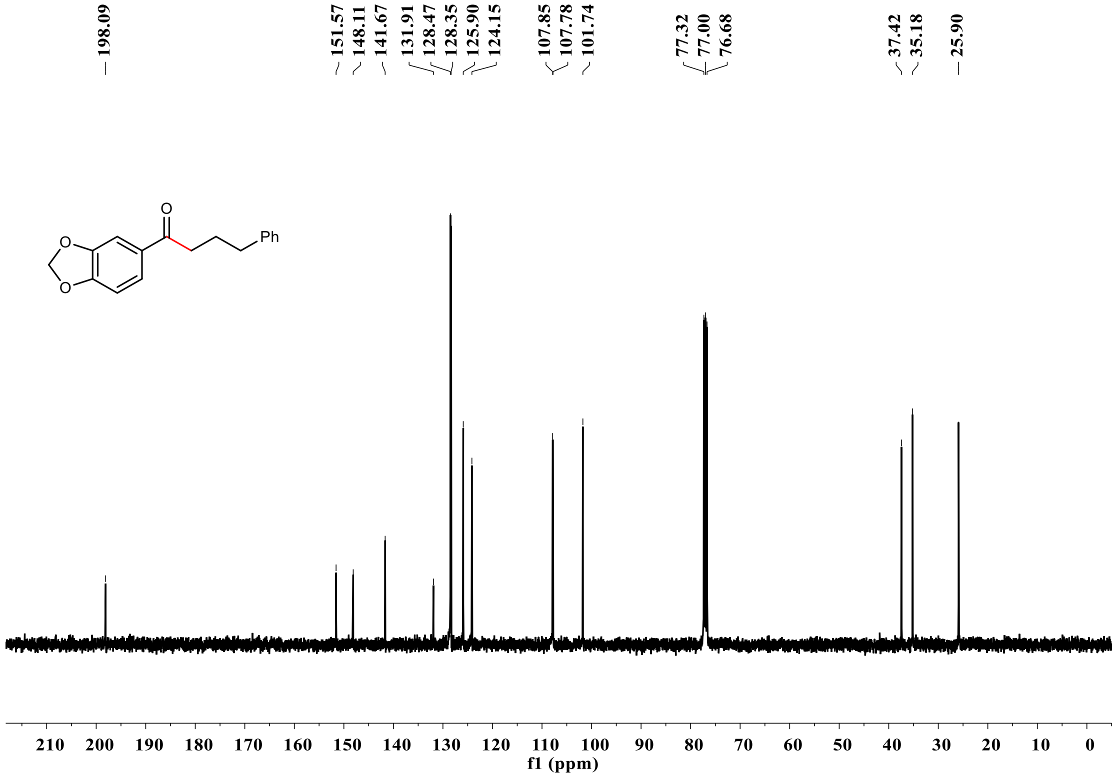

Supplementary Figure 46. ^{13}C NMR spectrum for **K14**

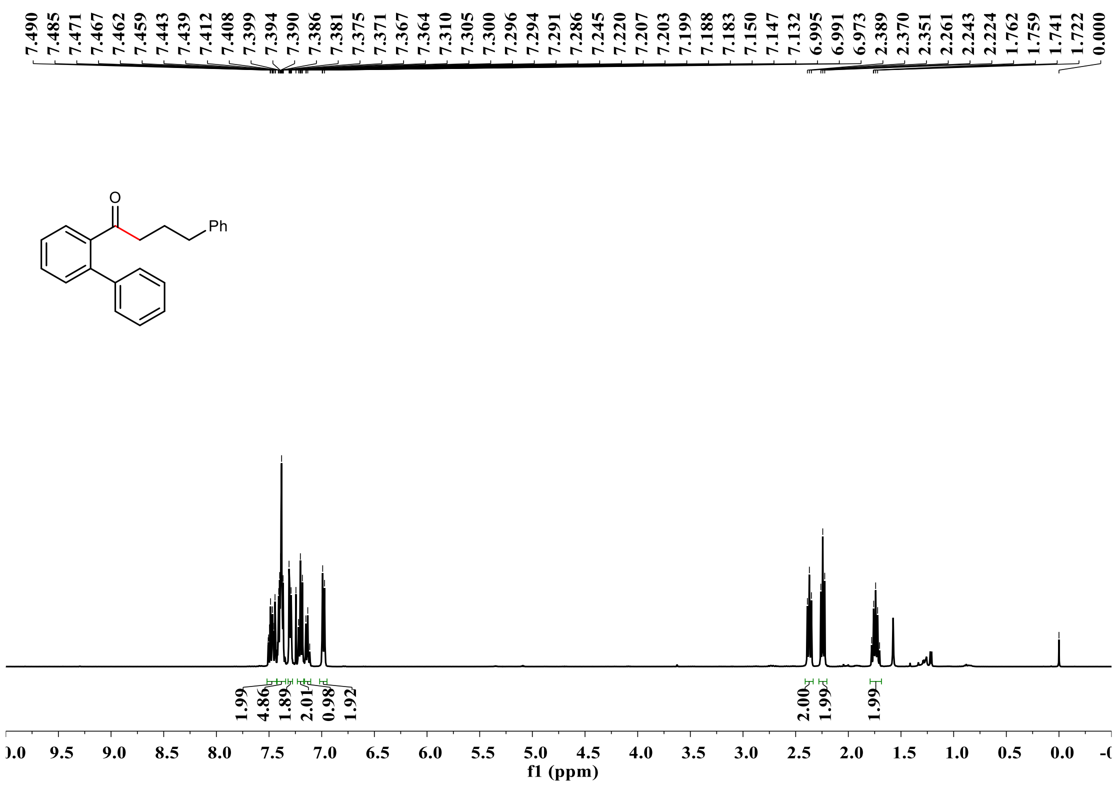

Supplementary Figure 47. ^{1}H NMR spectrum for **K15**

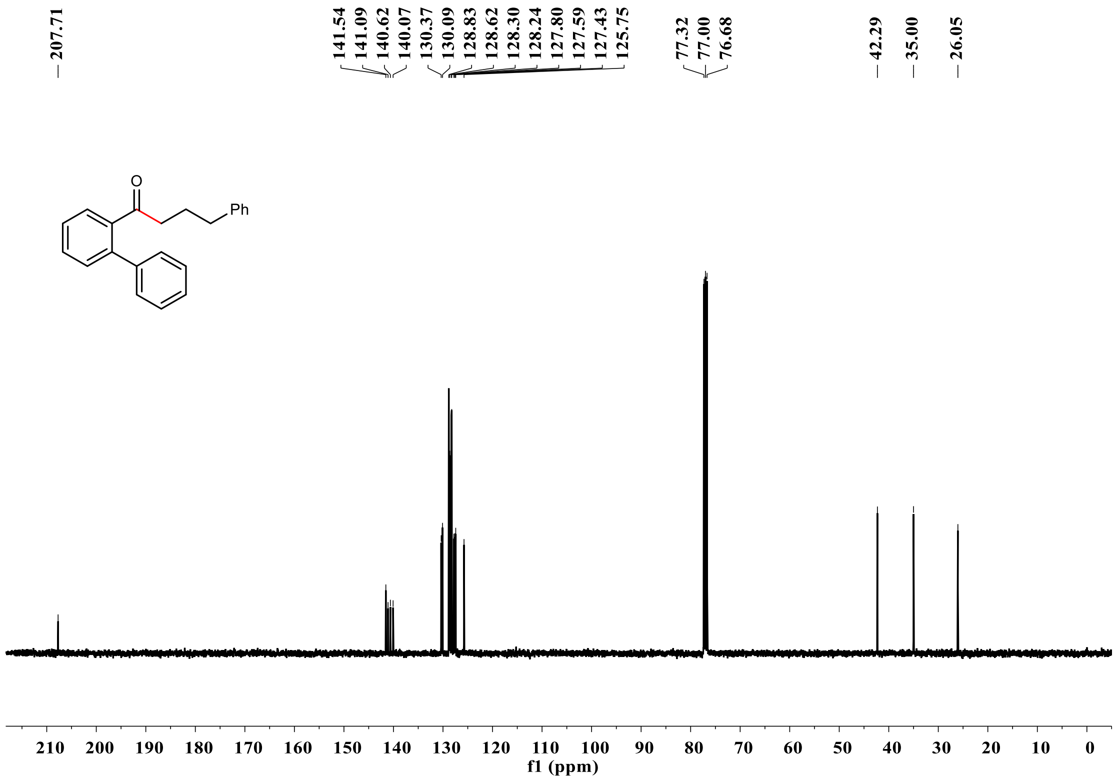

Supplementary Figure 48. ^{13}C NMR spectrum for **K15**

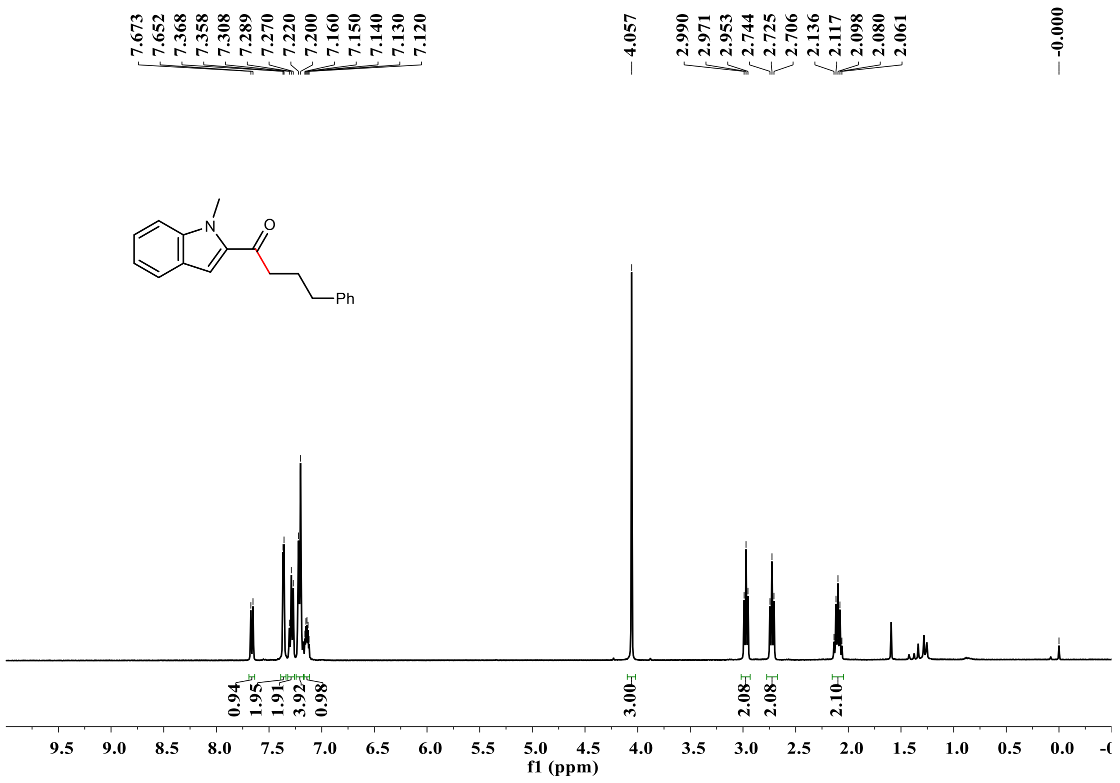

Supplementary Figure 49. ^{1}H NMR spectrum for **K16**

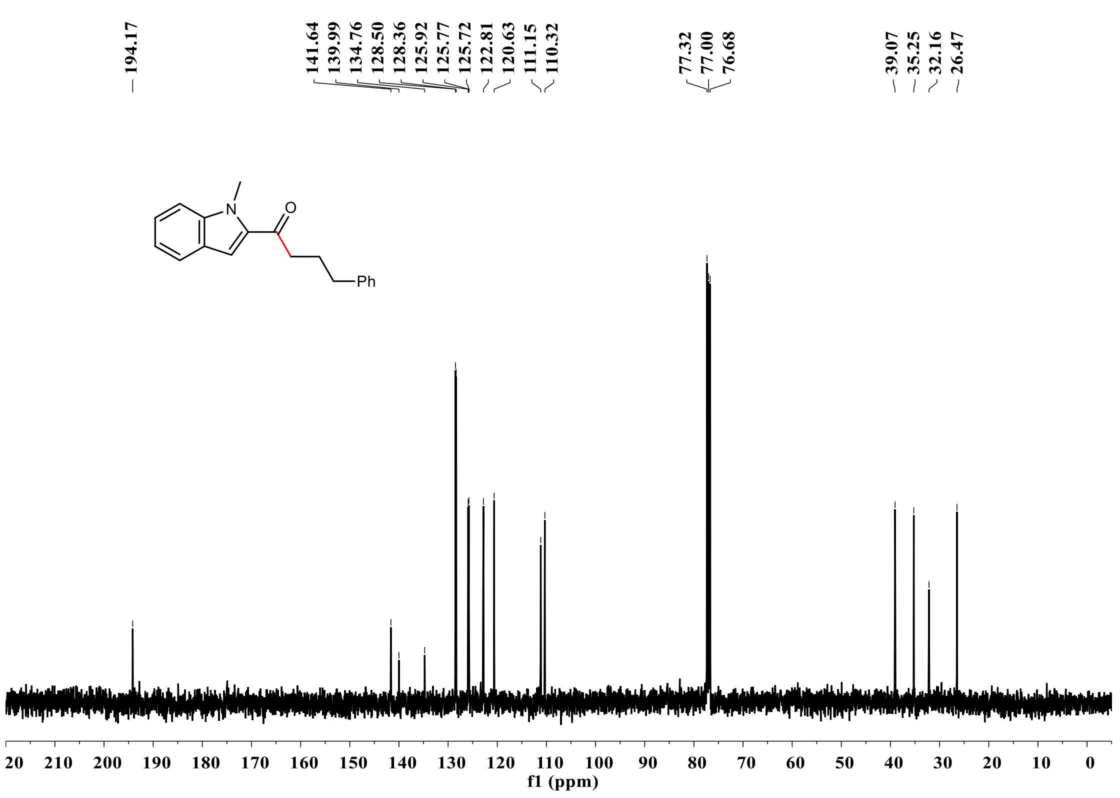

Supplementary Figure 50. ^{13}C NMR spectrum for **K16**

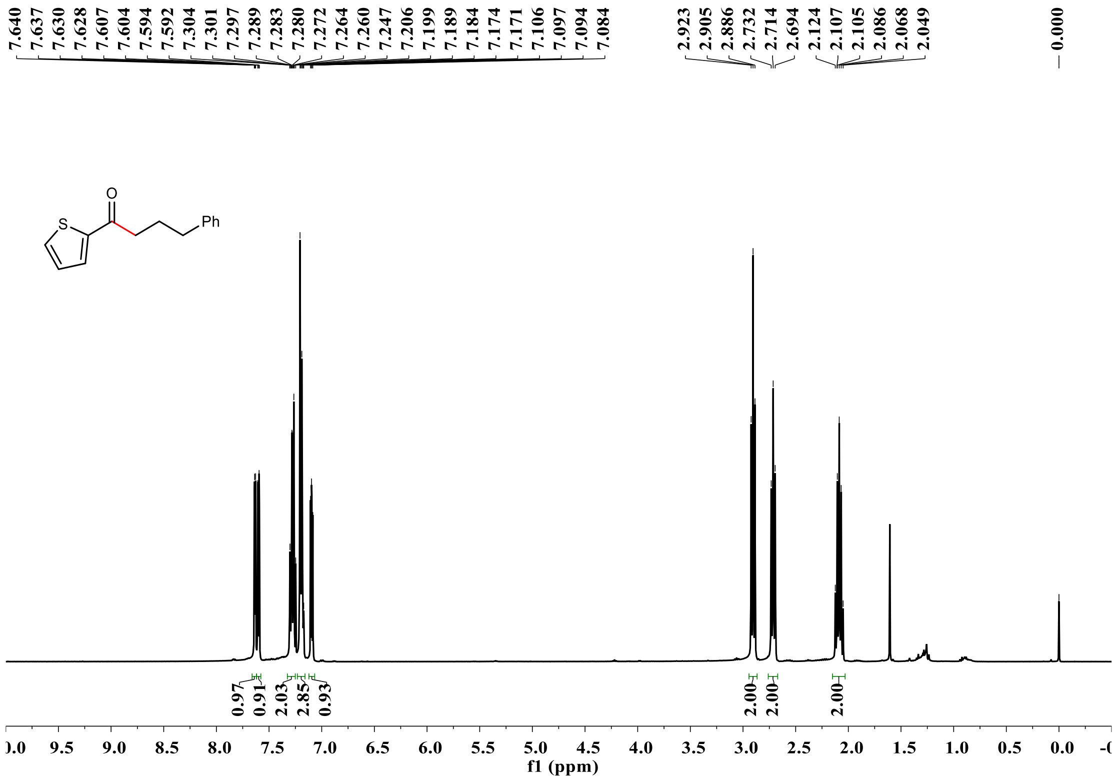

Supplementary Figure 51. ^{1}H NMR spectrum for **K17**

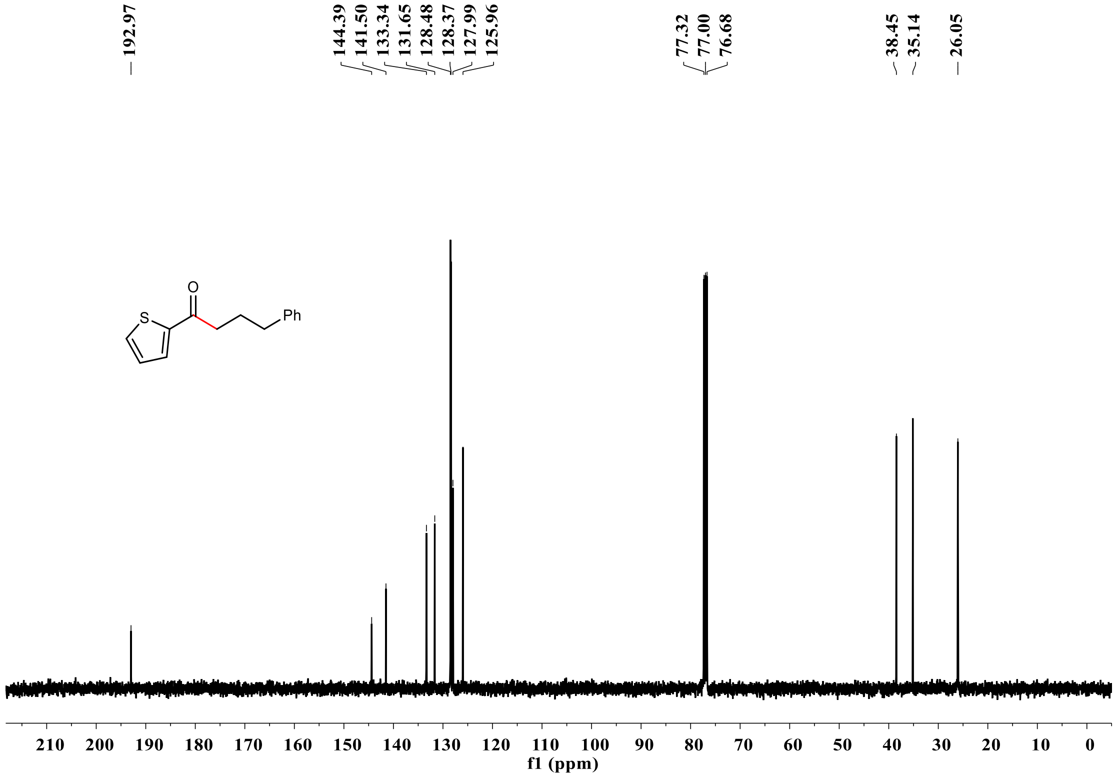

Supplementary Figure 52. ^{13}C NMR spectrum for **K17**

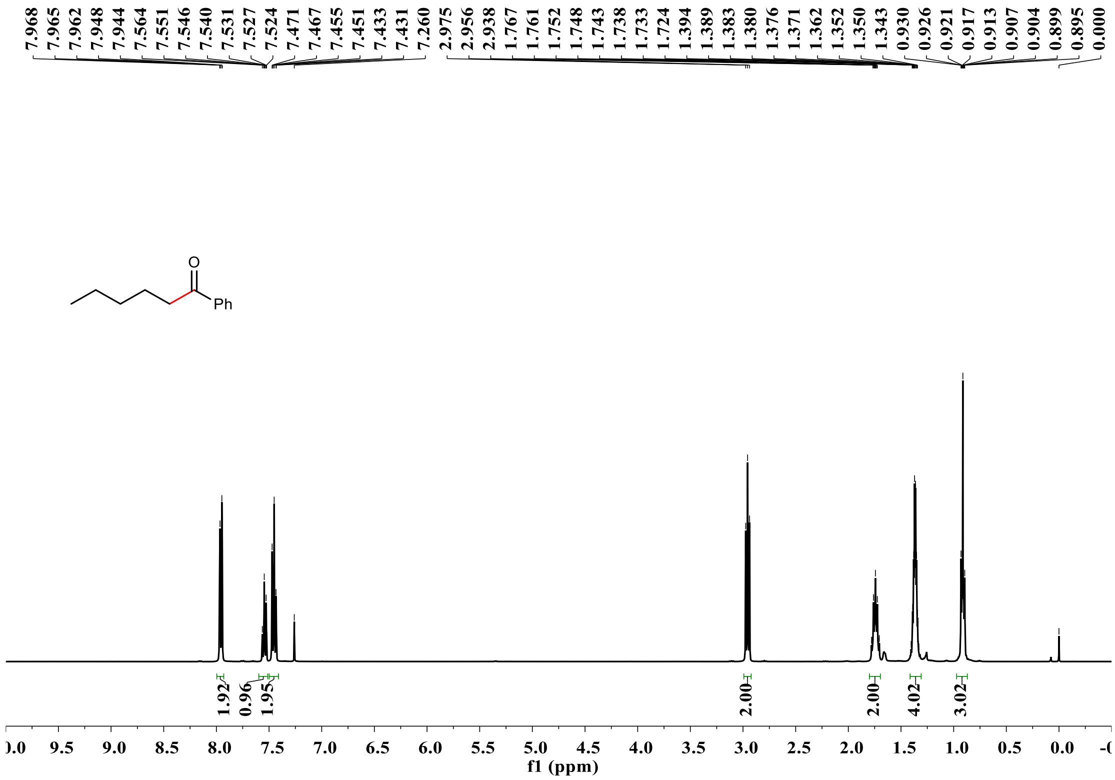

Supplementary Figure 53. ^{1}H NMR spectrum for **K18**

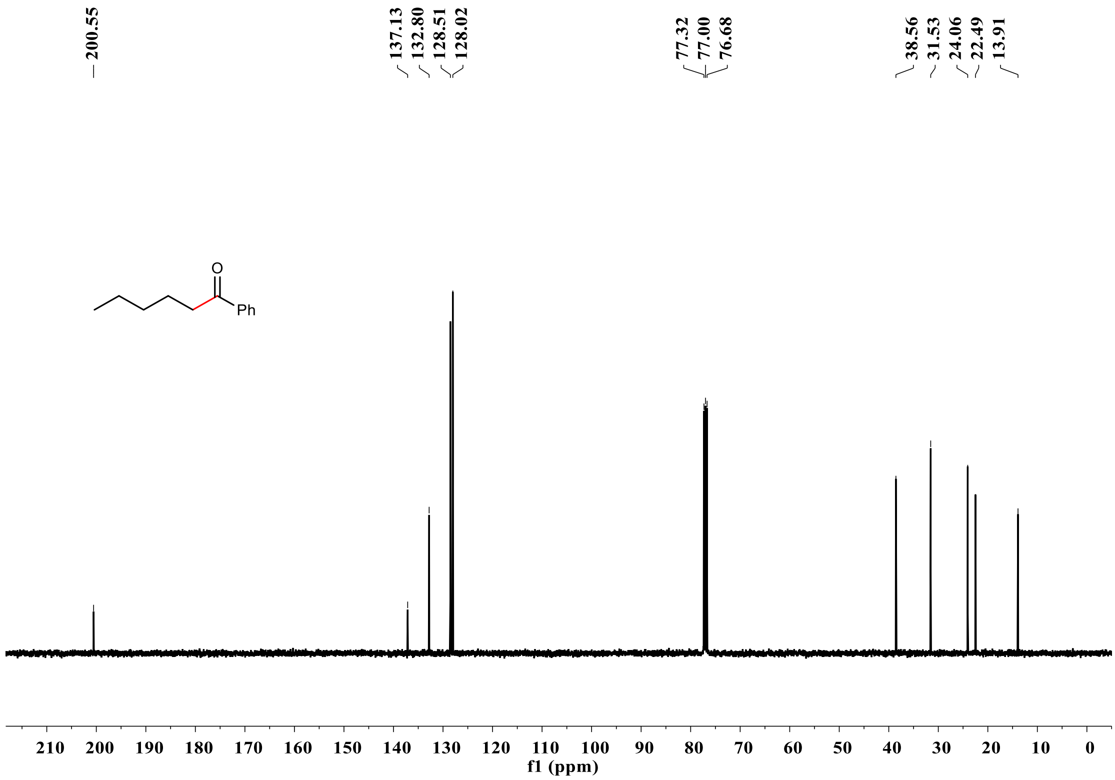

Supplementary Figure 54. ^{13}C NMR spectrum for **K18**

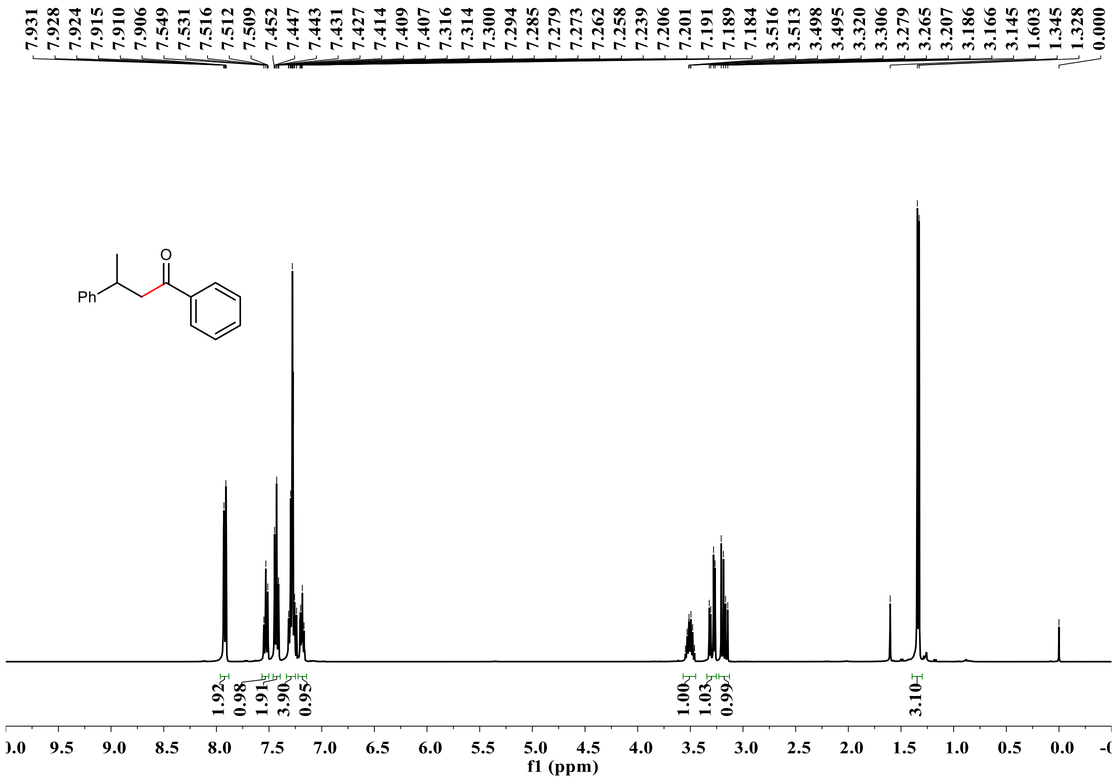

Supplementary Figure 55. ^{1}H NMR spectrum for **K19**

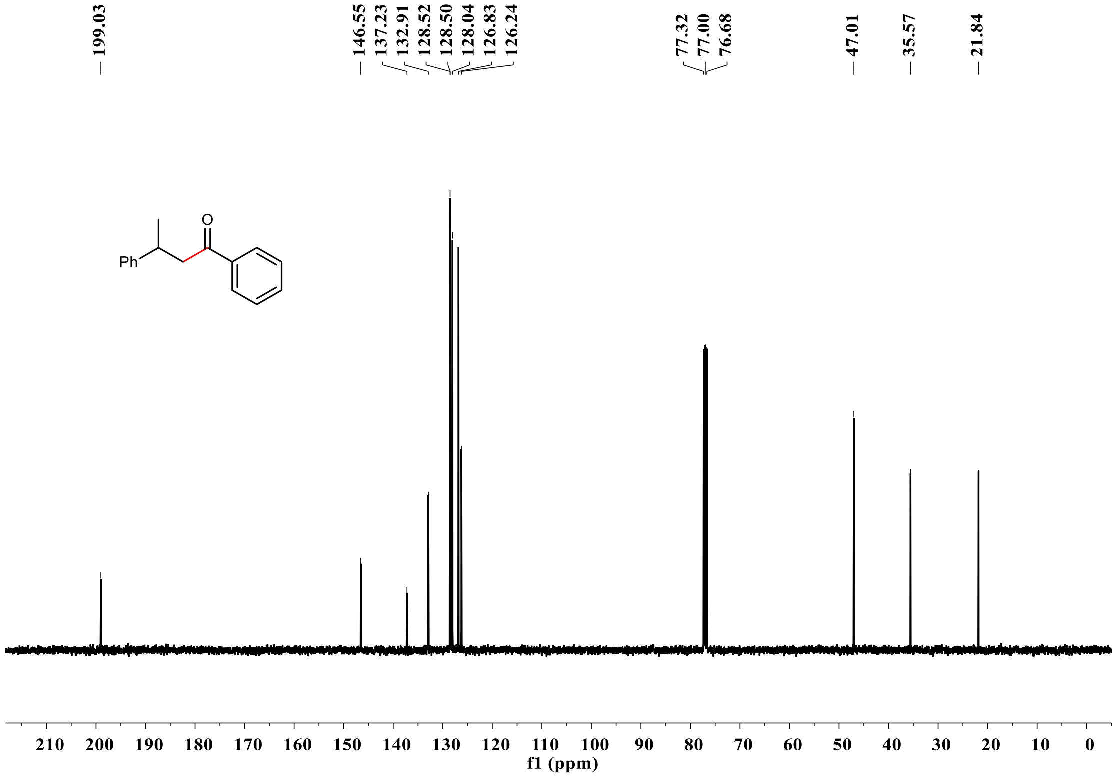

Supplementary Figure 56. ^{13}C NMR spectrum for **K19**

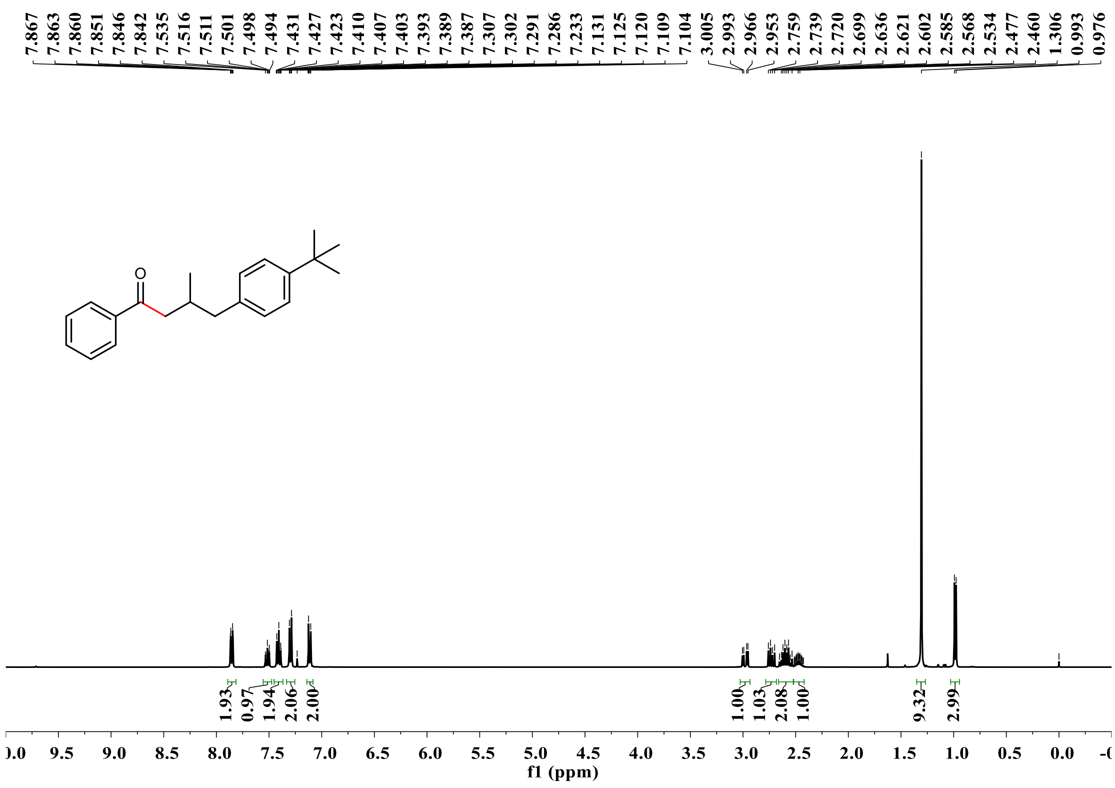

Supplementary Figure 57. ^{1}H NMR spectrum for **K20**

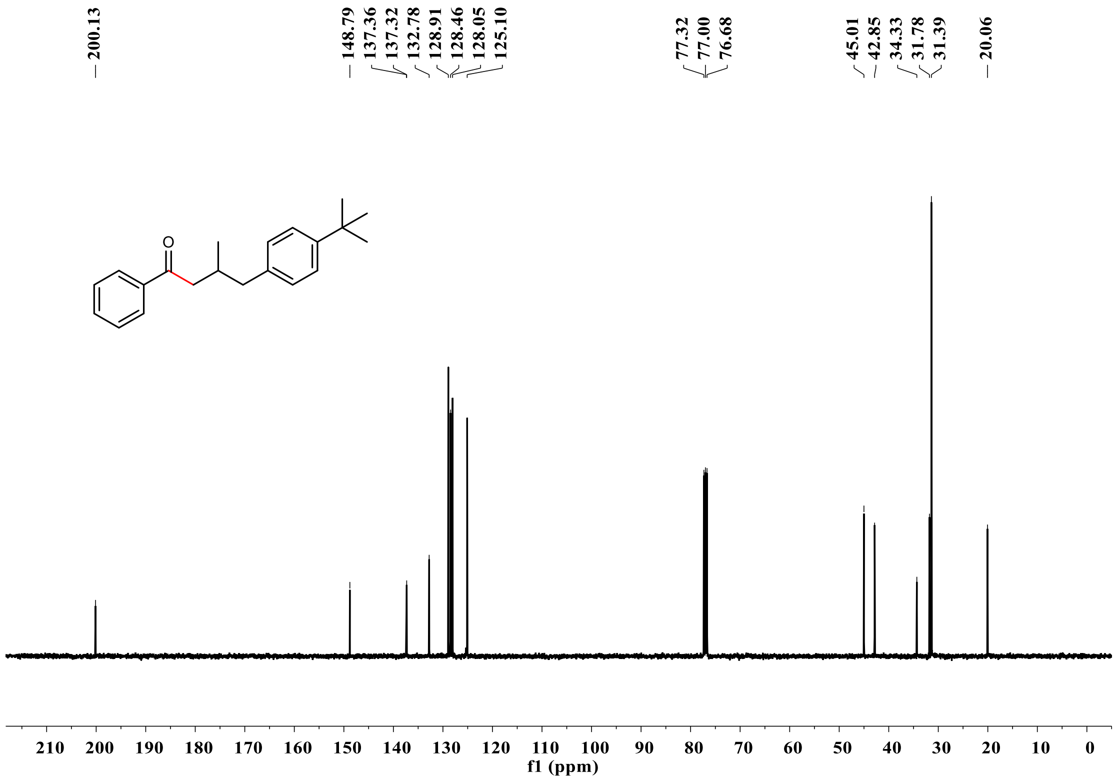

Supplementary Figure 58. ^{13}C NMR spectrum for **K20**

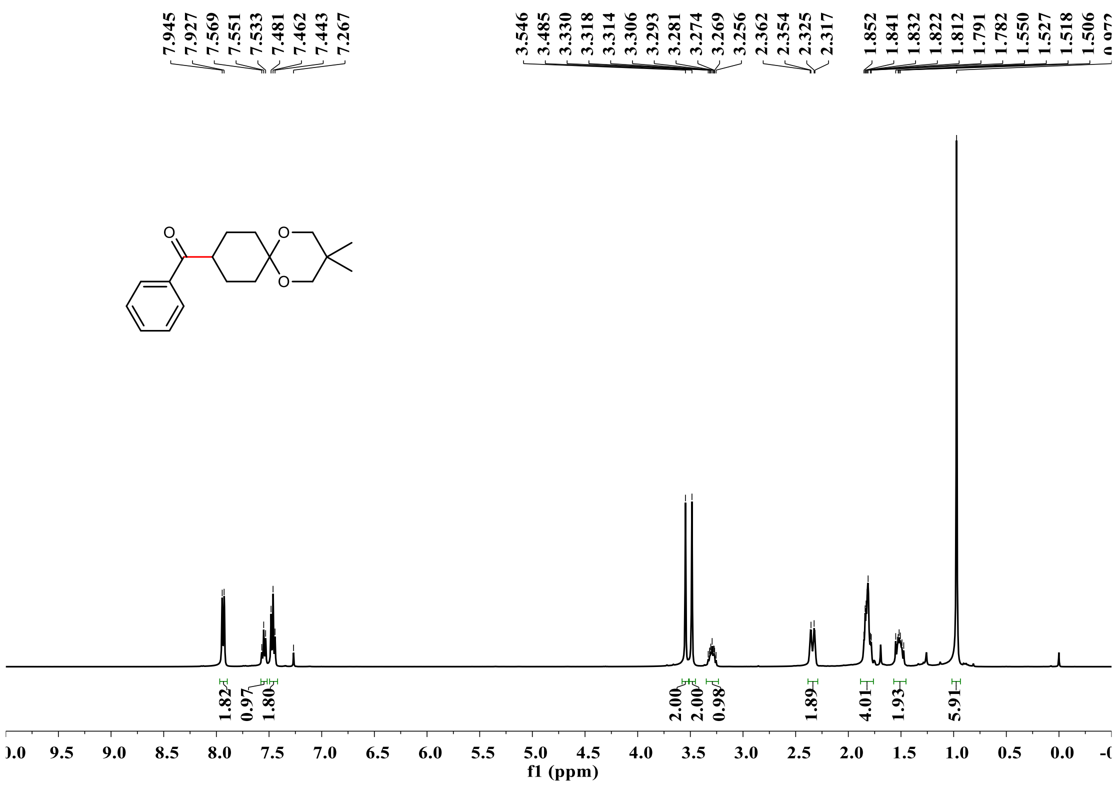

Supplementary Figure 59. ^{1}H NMR spectrum for **K21**

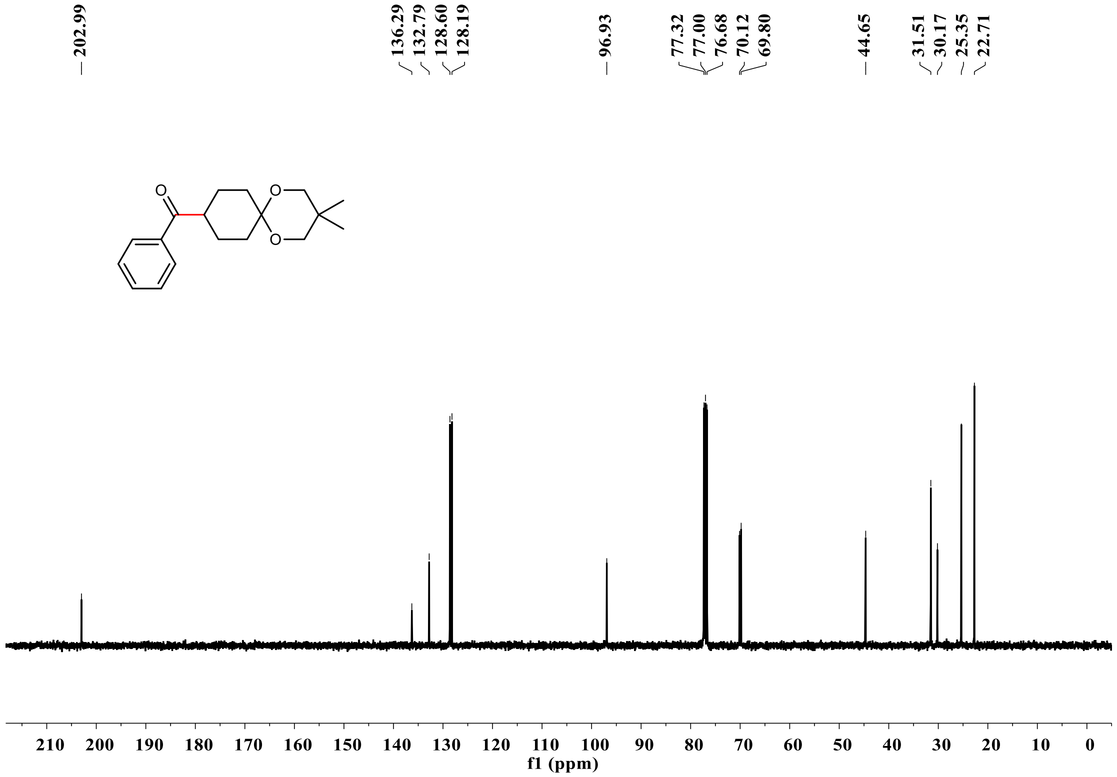

Supplementary Figure 60. ^{13}C NMR spectrum for **K21**

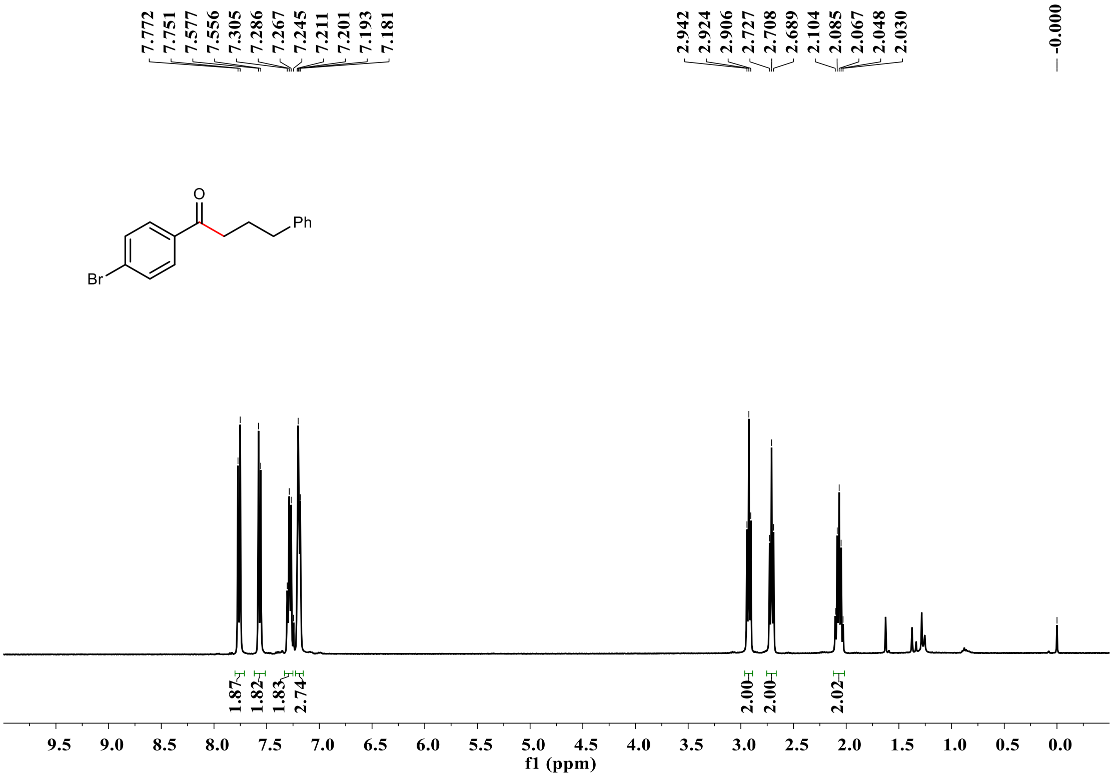

Supplementary Figure 61. ^{1}H NMR spectrum for **K22**

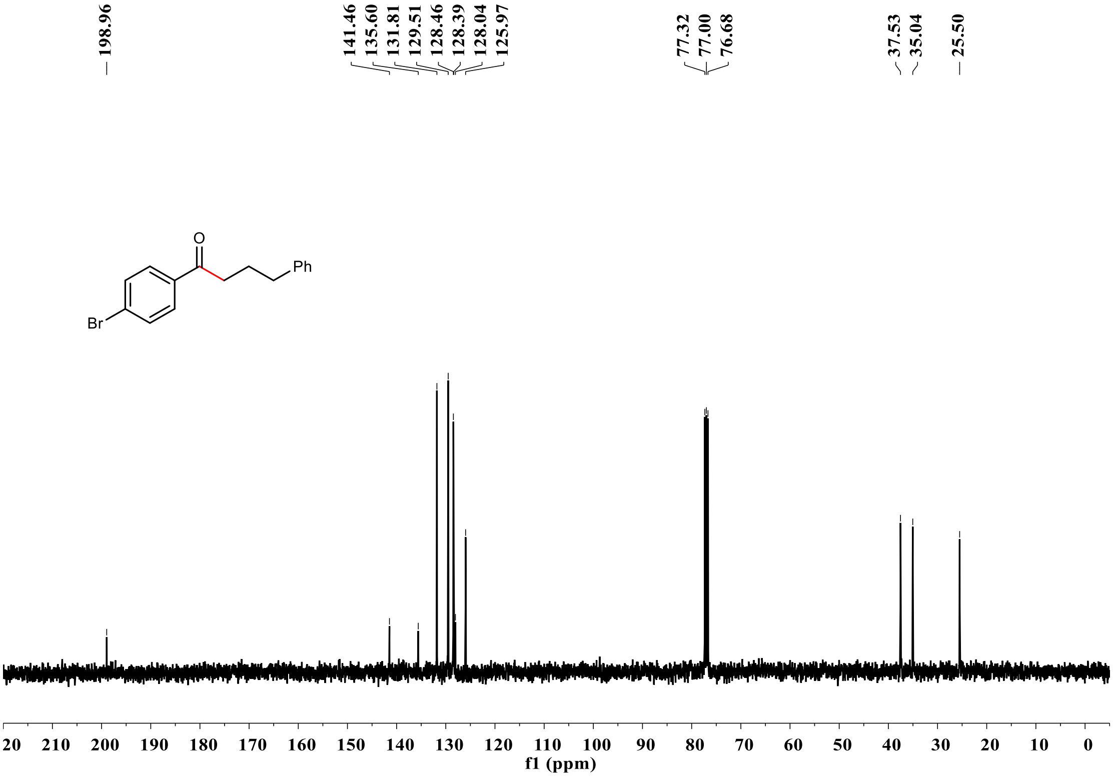

Supplementary Figure 62. ^{13}C NMR spectrum for **K22**

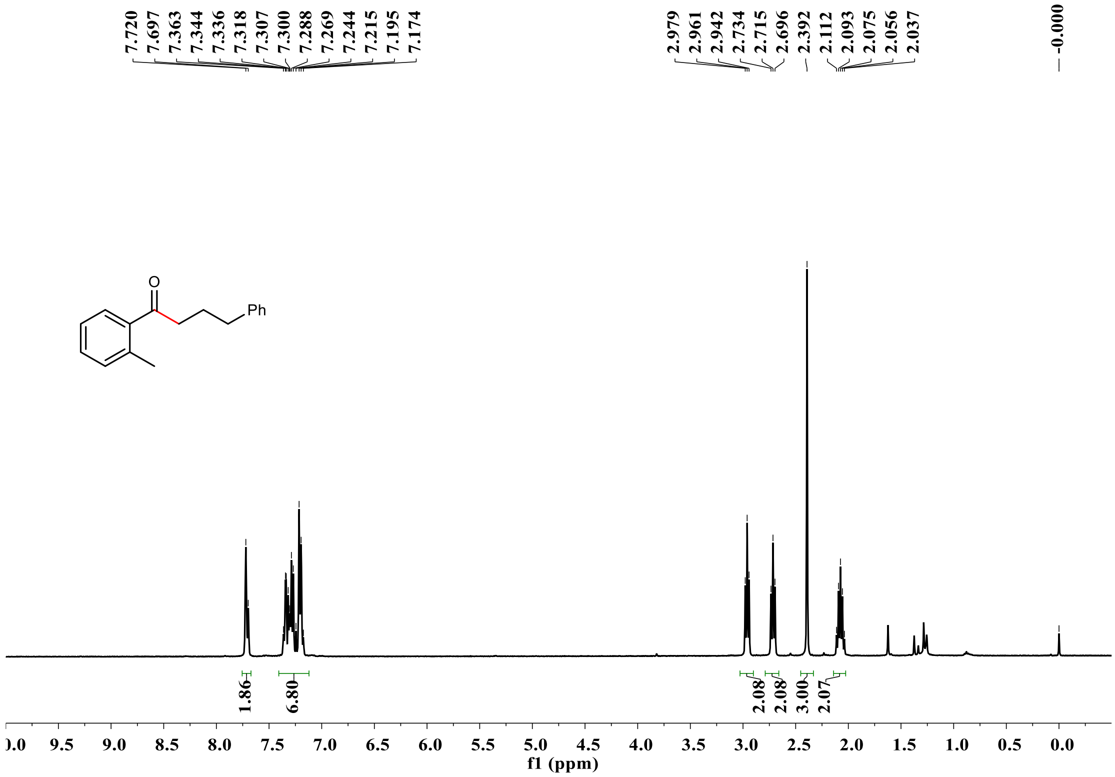

Supplementary Figure 63. ^{1}H NMR spectrum for **K23**

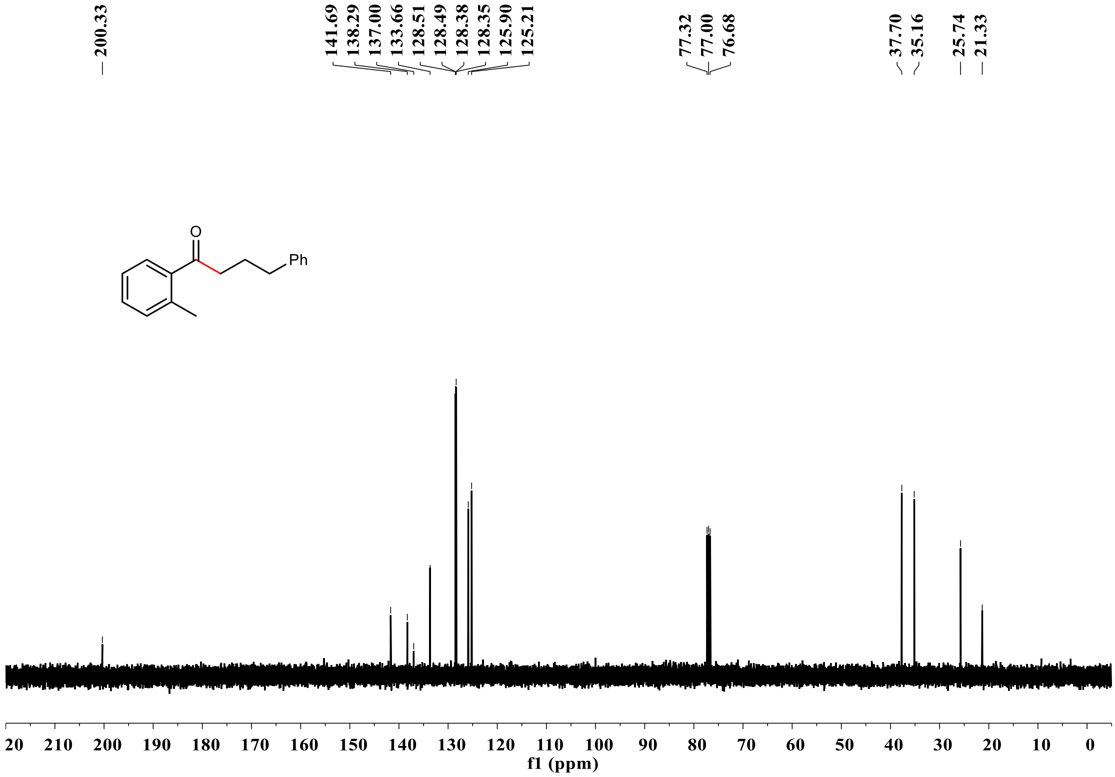

Supplementary Figure 64. ^{13}C NMR spectrum for **K23**

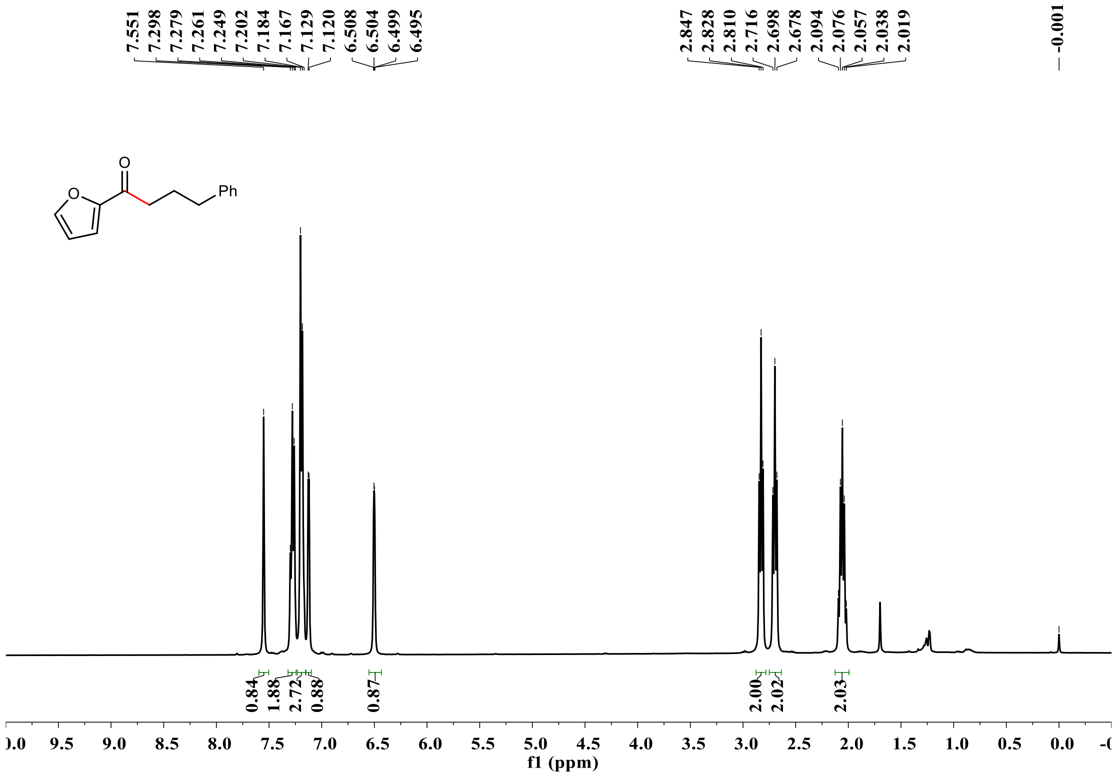

Supplementary Figure 65. ^{1}H NMR spectrum for **K24**

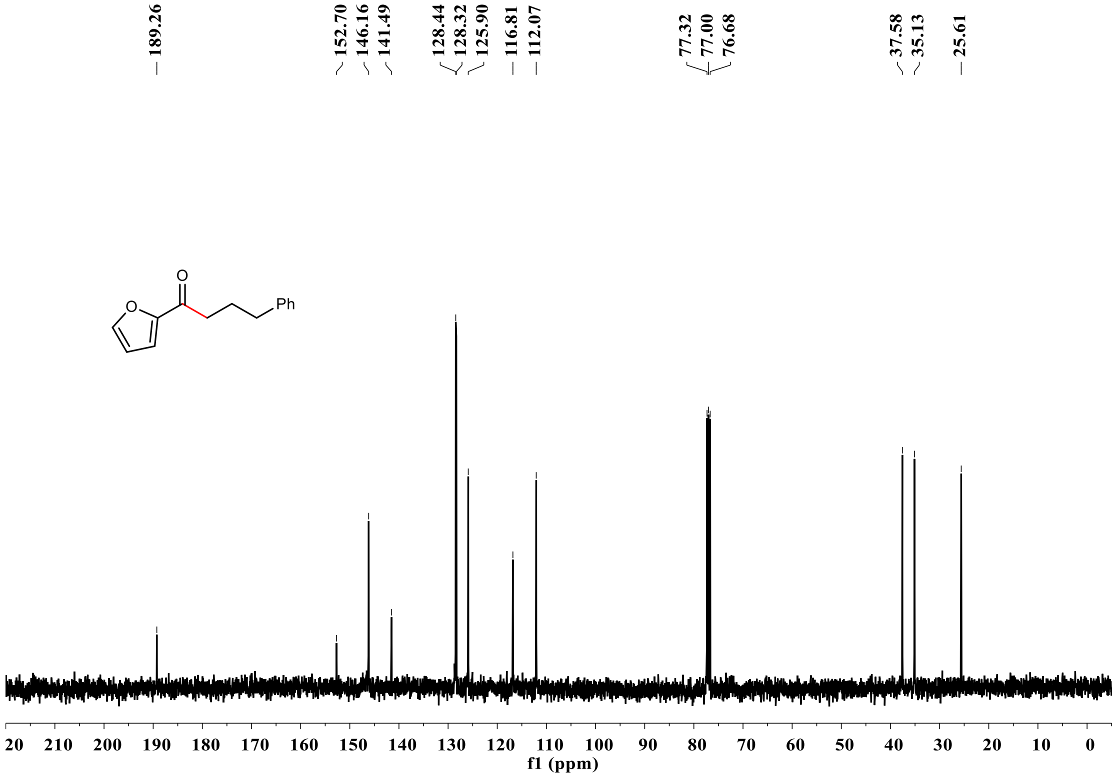

Supplementary Figure 66. ^{13}C NMR spectrum for **K24**

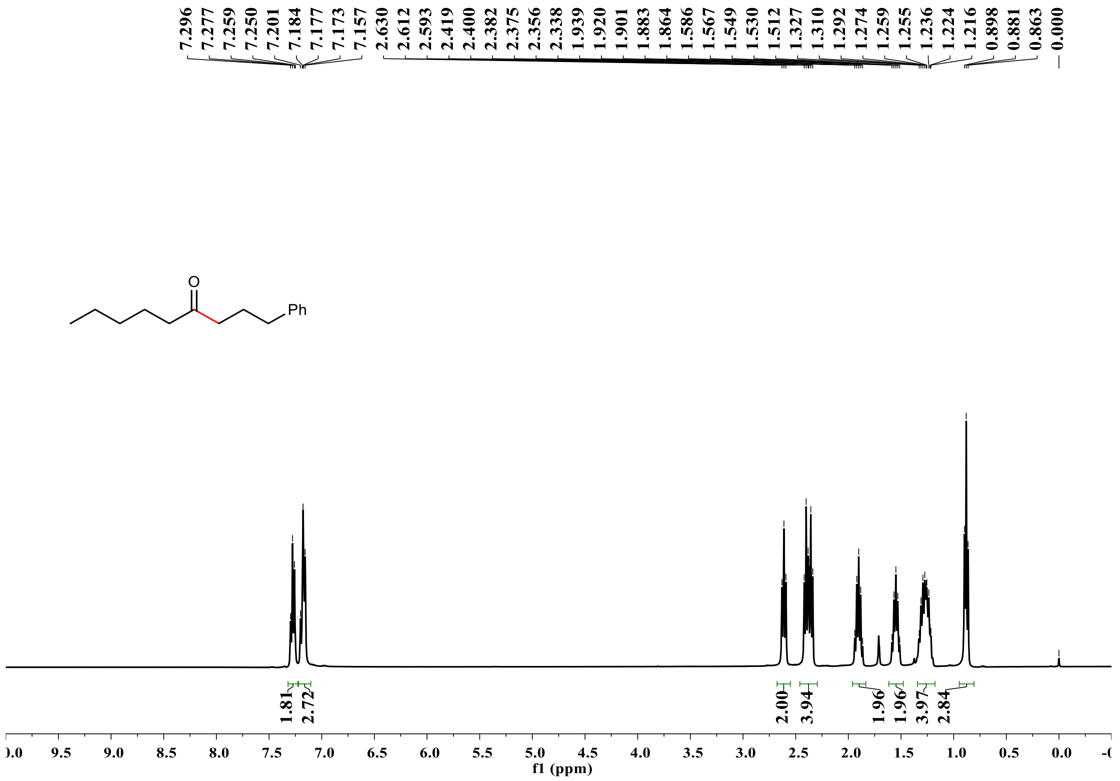

Supplementary Figure 67. ^{1}H NMR spectrum for **K25**

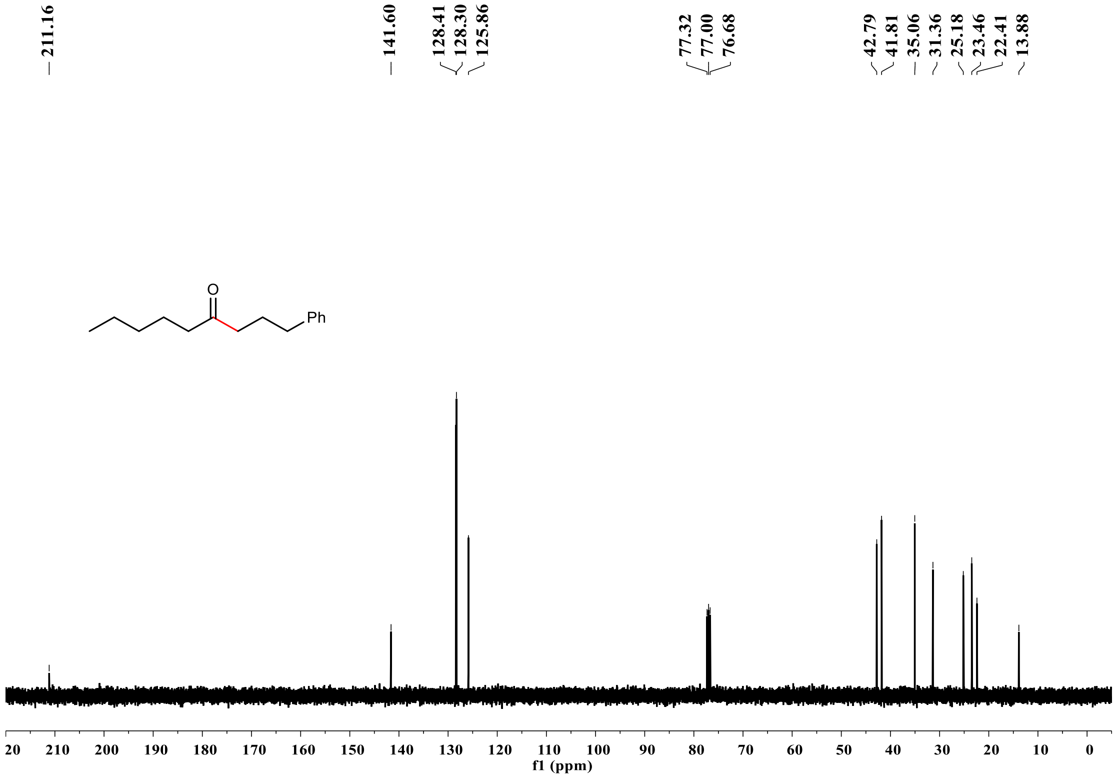

Supplementary Figure 68. ^{13}C NMR spectrum for **K25**

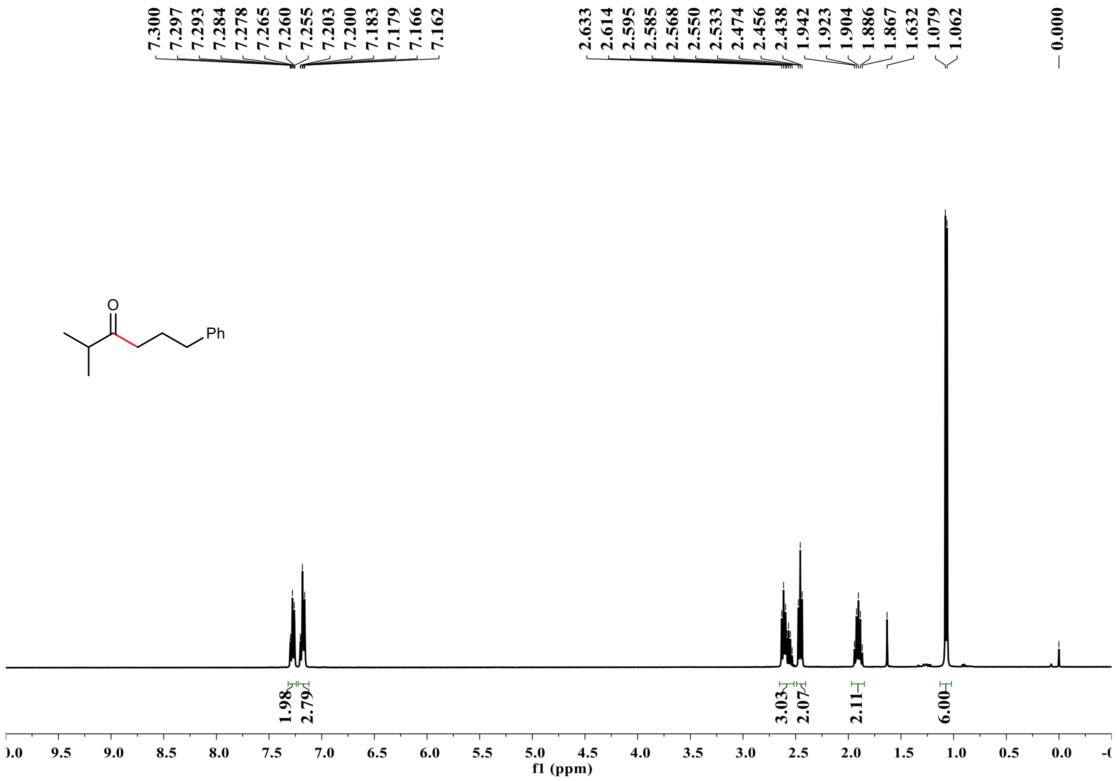

Supplementary Figure 69. ^{1}H NMR spectrum for **K26**

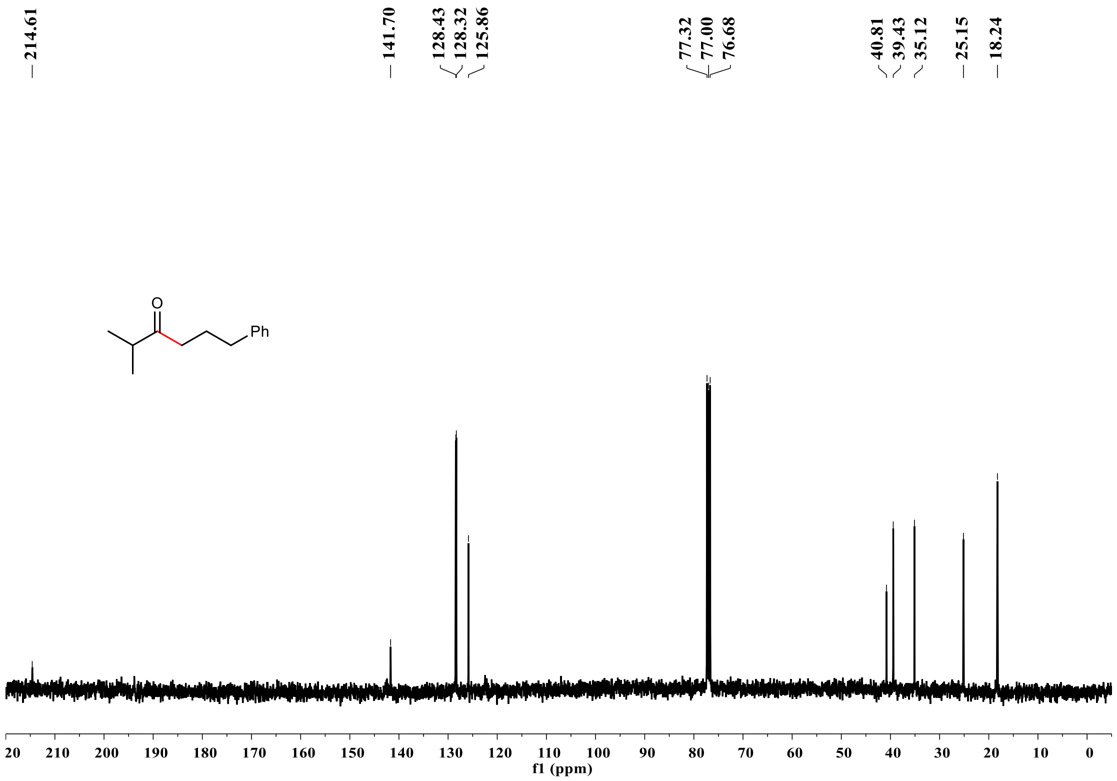

Supplementary Figure 70. ^{13}C NMR spectrum for **K26**

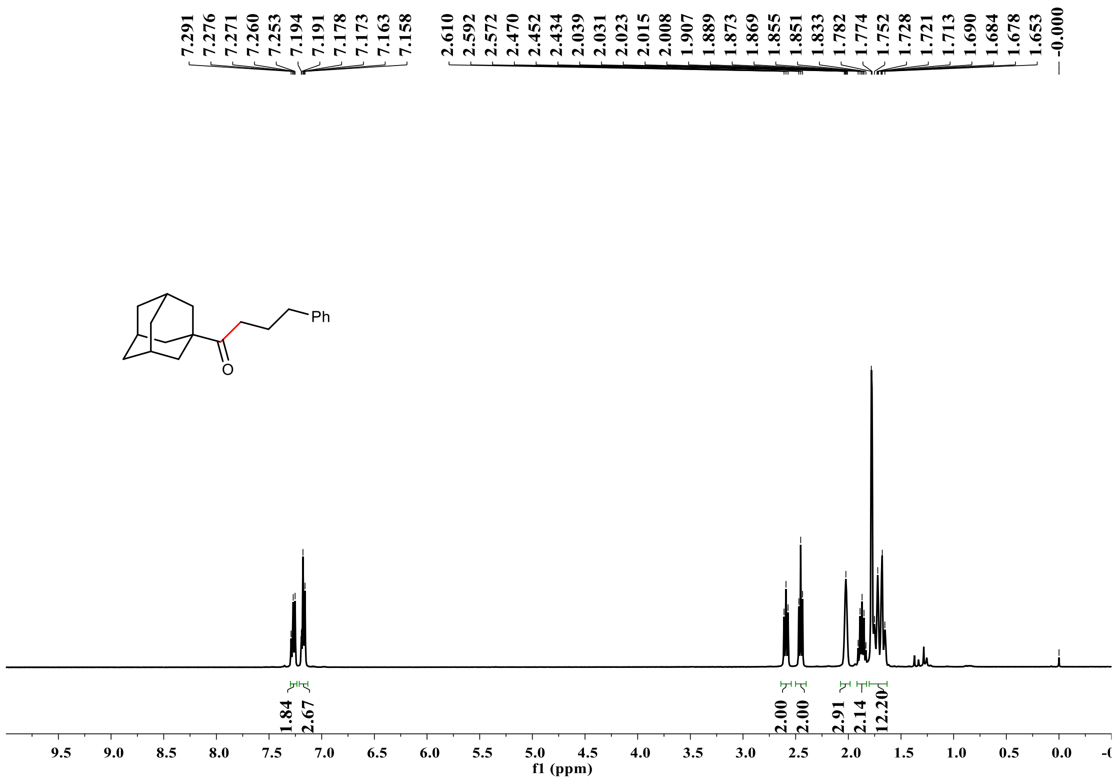

Supplementary Figure 71. ^{1}H NMR spectrum for **K27**

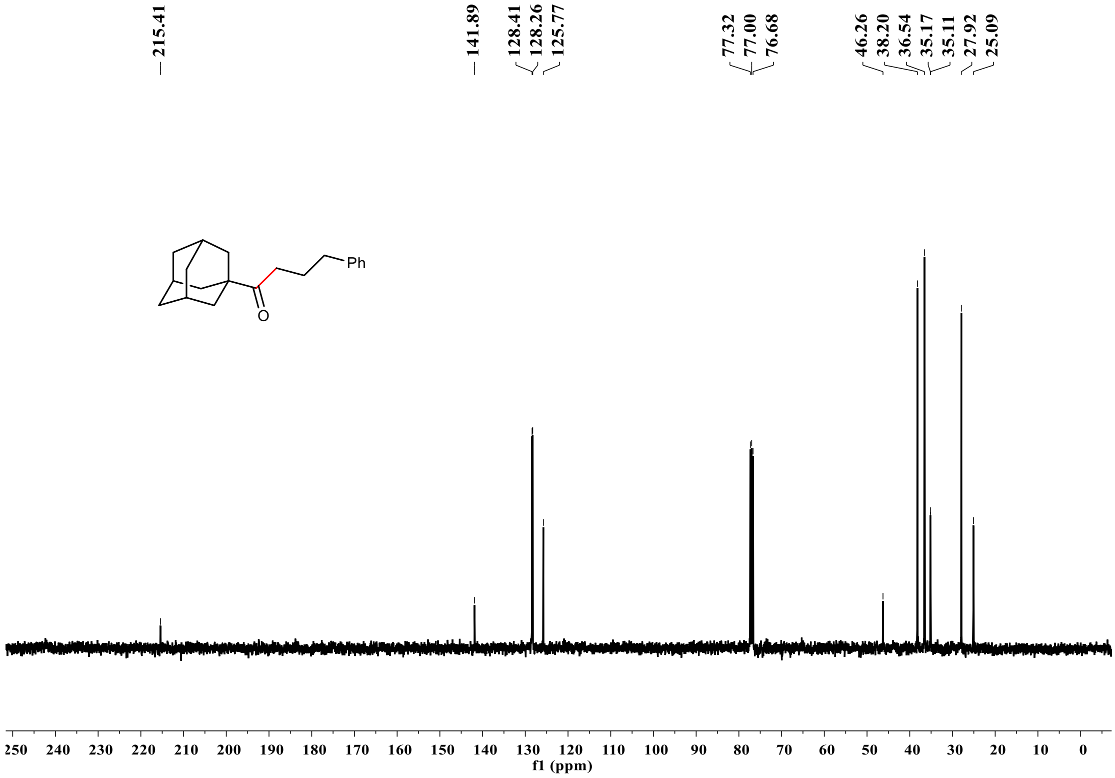

Supplementary Figure 72. ^{13}C NMR spectrum for **K27**

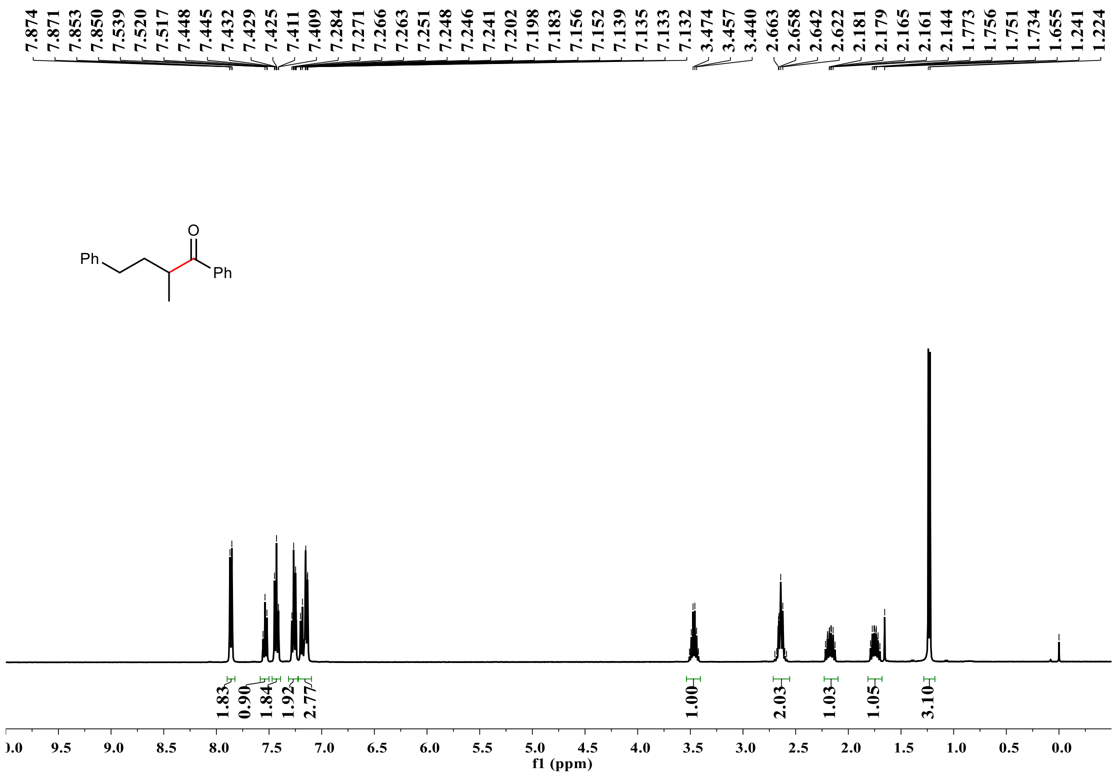

Supplementary Figure 73. ^{1}H NMR spectrum for **K28**

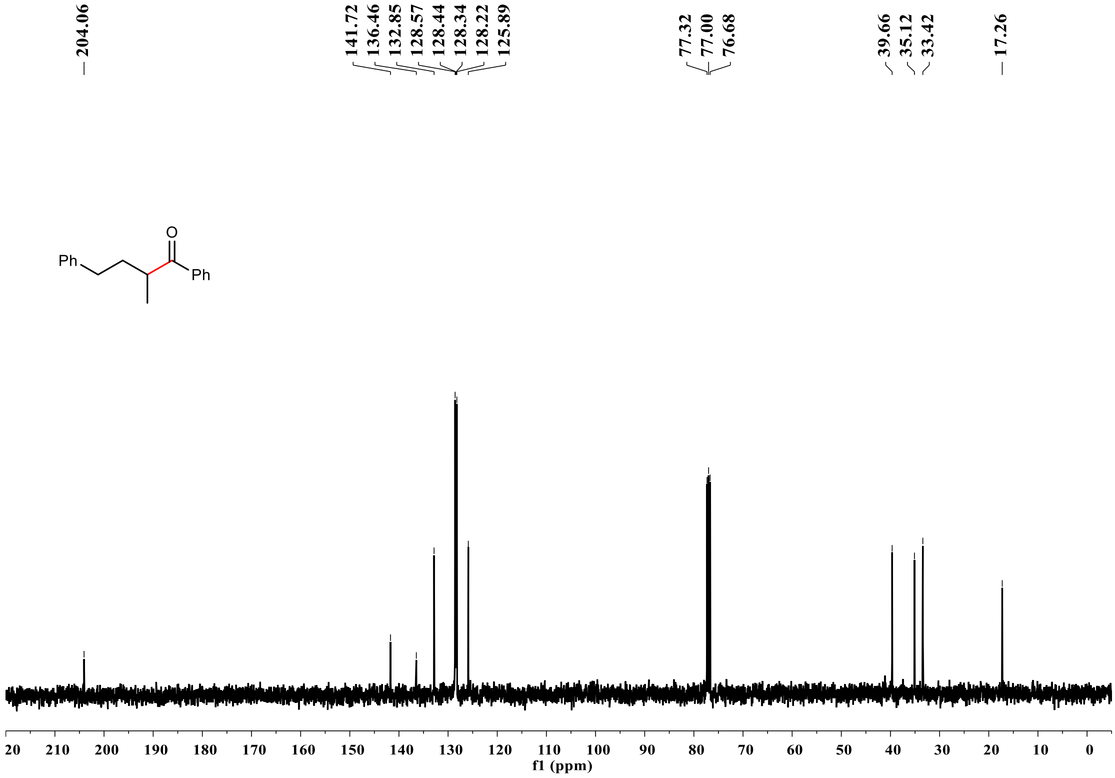

Supplementary Figure 74. ^{13}C NMR spectrum for **K28**

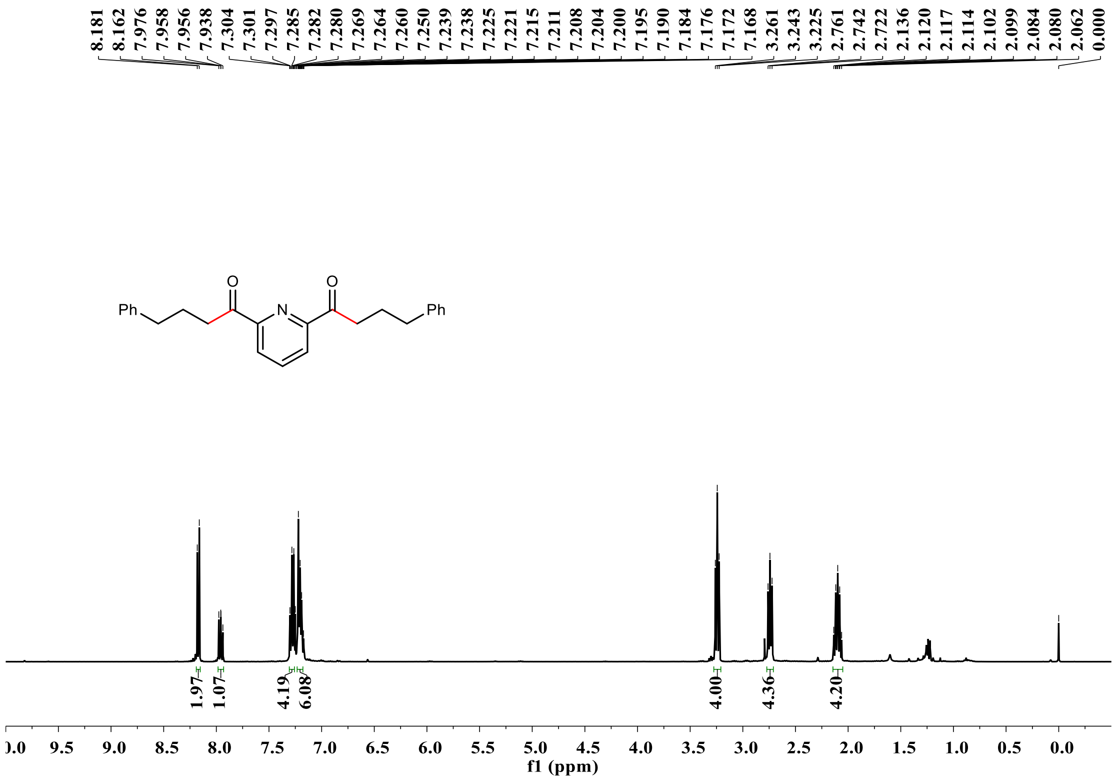

Supplementary Figure 75. ^{1}H NMR spectrum for **K29**

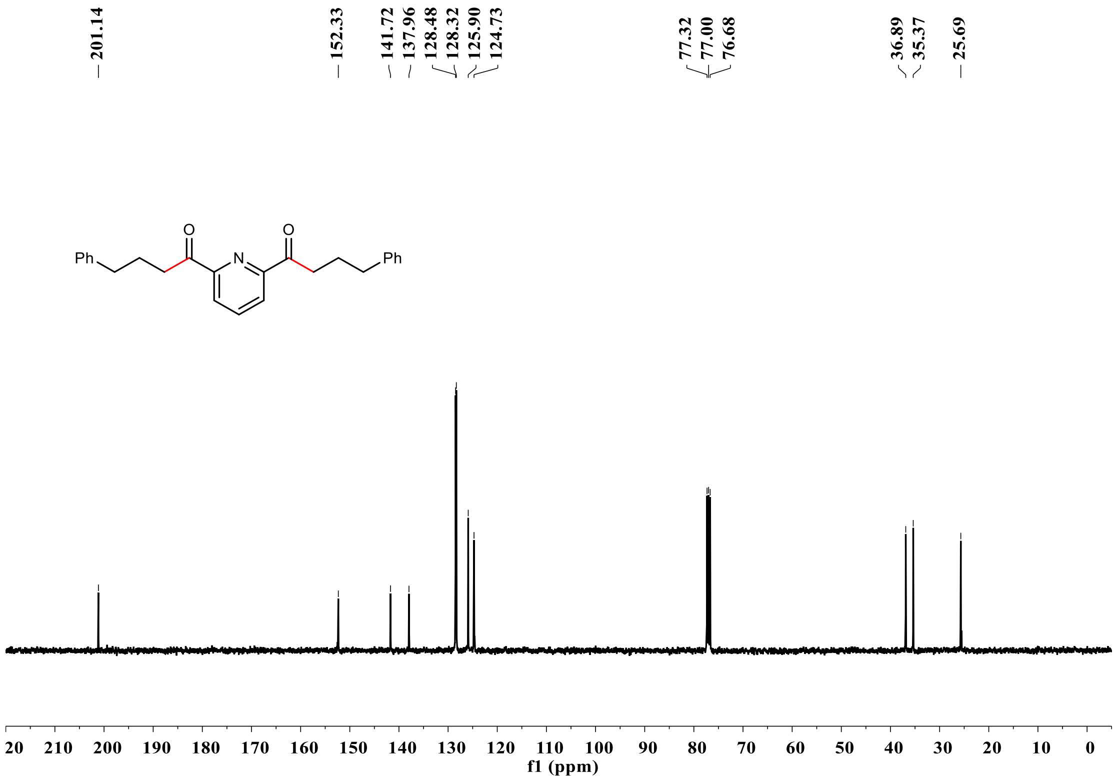

Supplementary Figure 76. ^{13}C NMR spectrum for **K29**

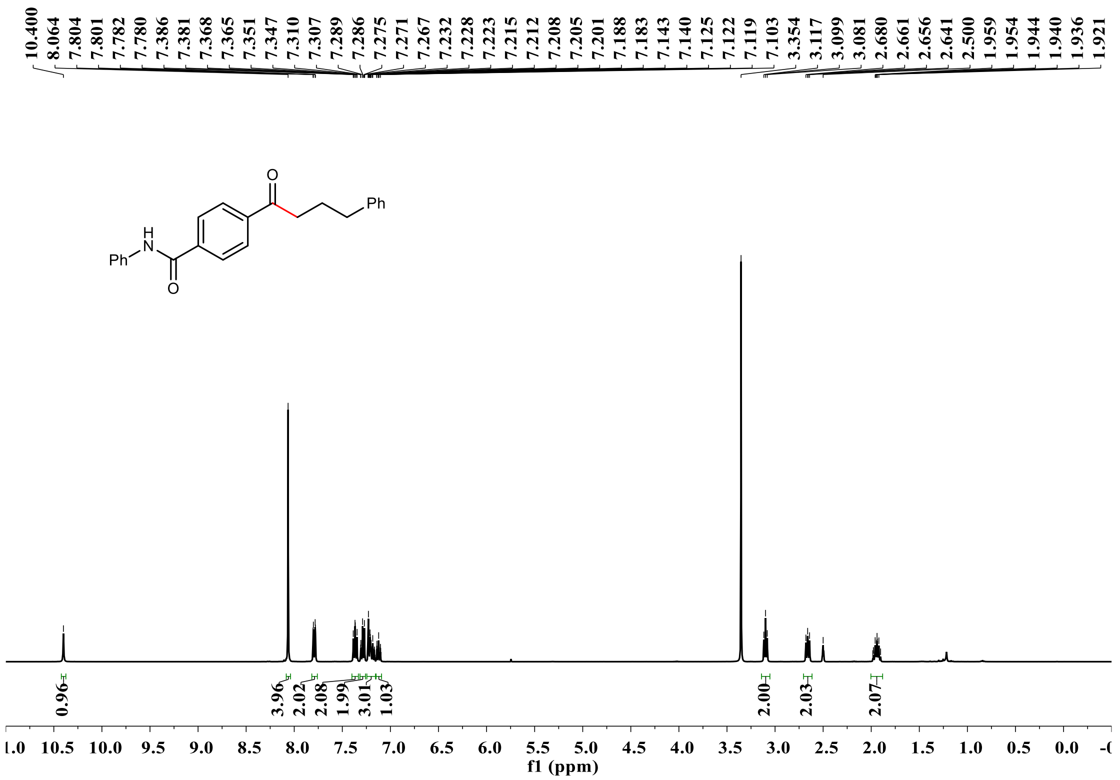

Supplementary Figure 77. ^{1}H NMR spectrum for **K30**

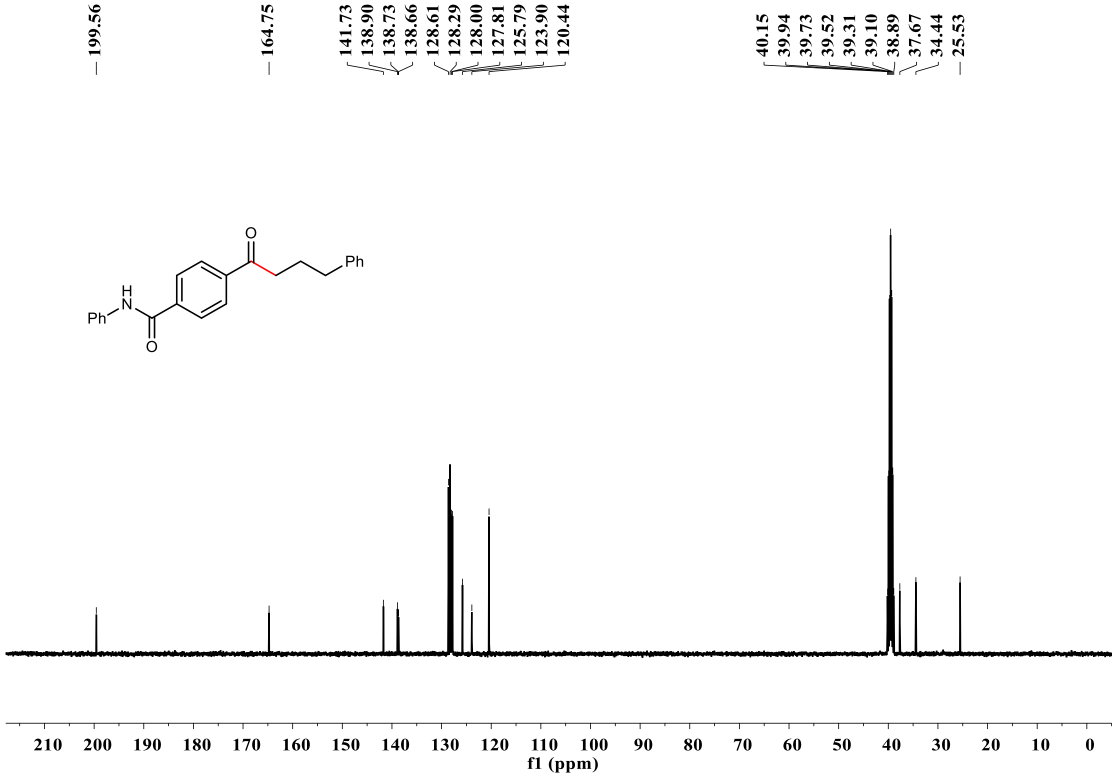

Supplementary Figure 78. ^{13}C NMR spectrum for **K30**

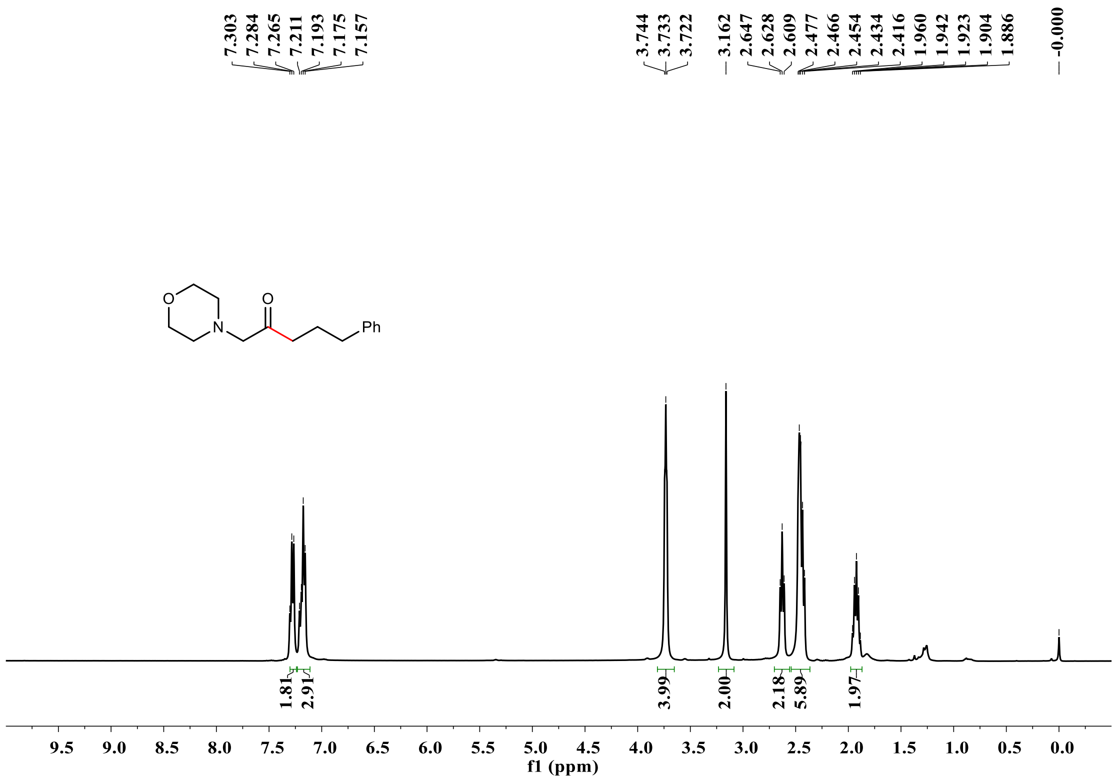

Supplementary Figure 79. ^{1}H NMR spectrum for **K31**

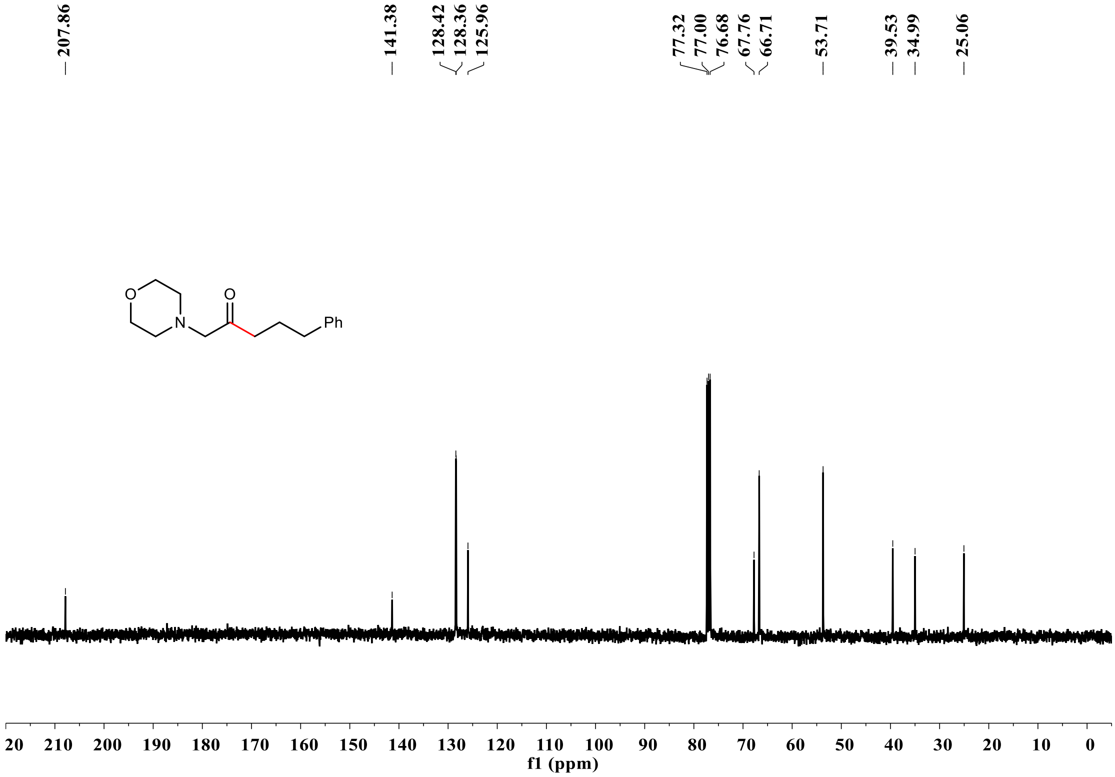

Supplementary Figure 80. ^{13}C NMR spectrum for **K31**

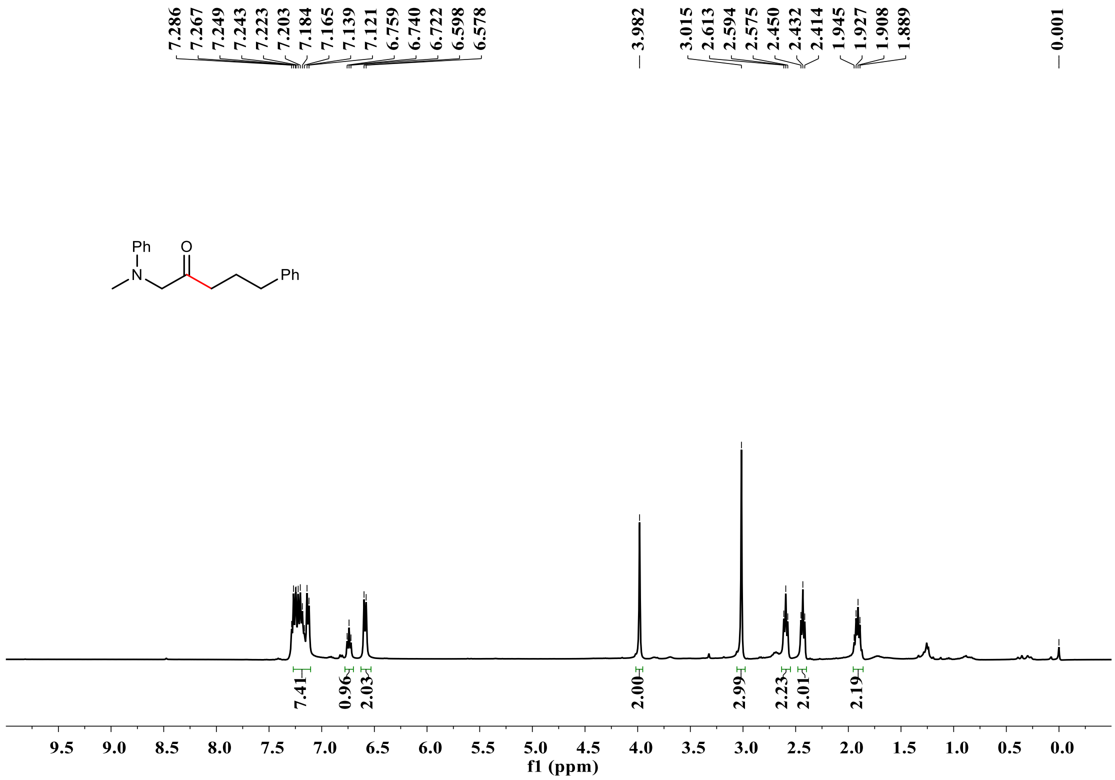

Supplementary Figure 81. ^{1}H NMR spectrum for **K32**

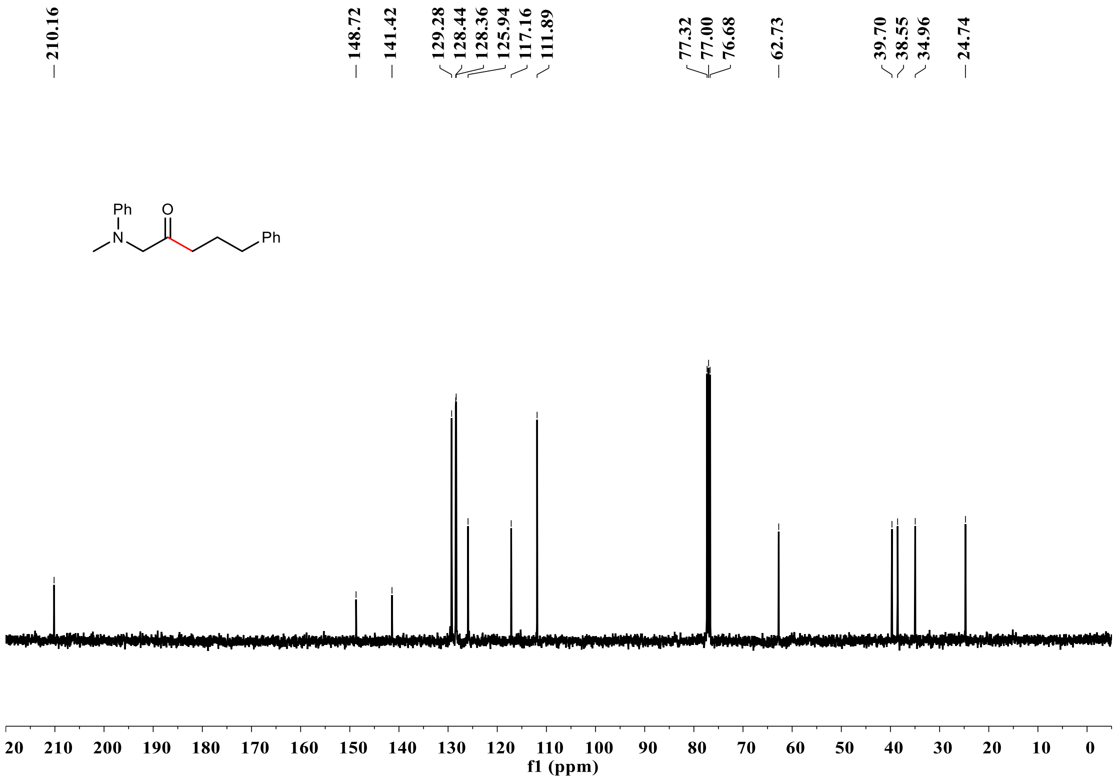

Supplementary Figure 82. ^{13}C NMR spectrum for **K32**

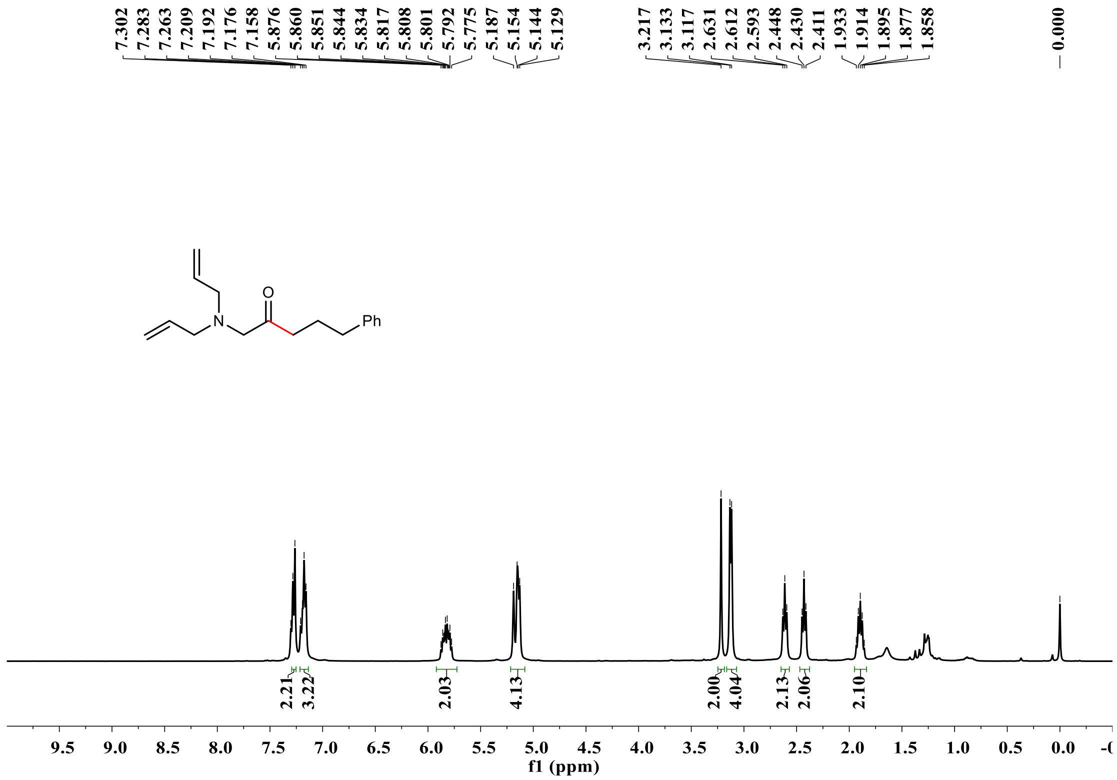

Supplementary Figure 83. ^{1}H NMR spectrum for **K33**

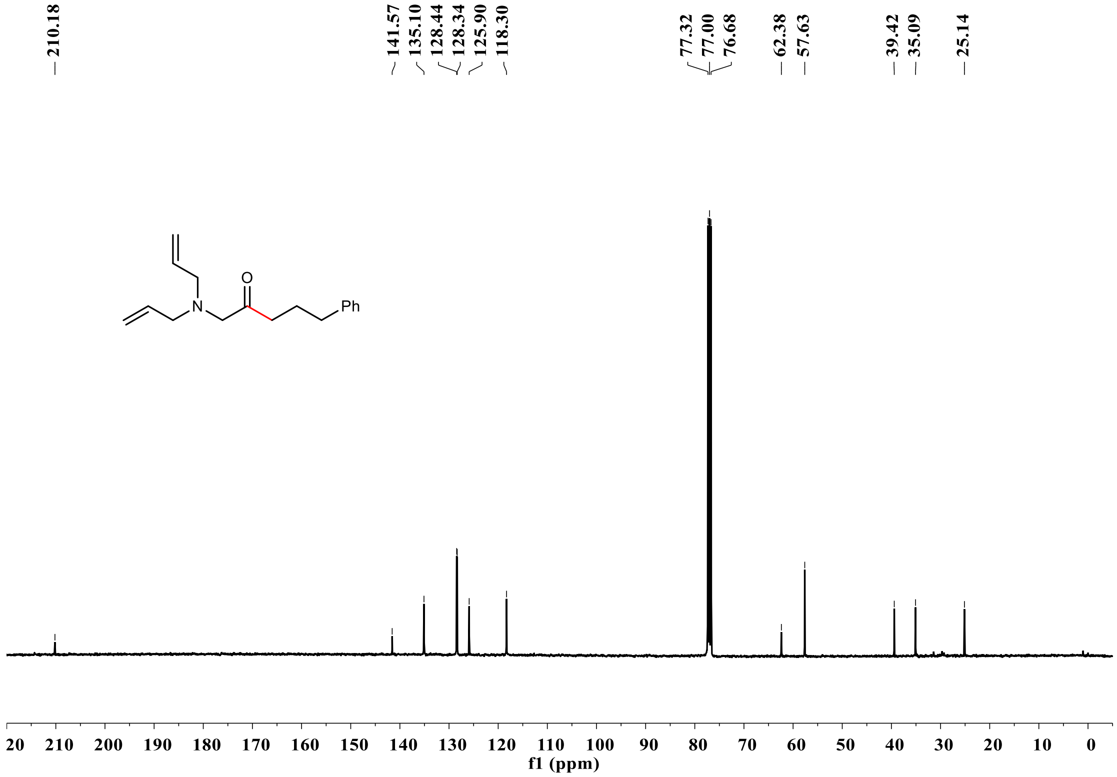

Supplementary Figure 84. ^{13}C NMR spectrum for **K33**

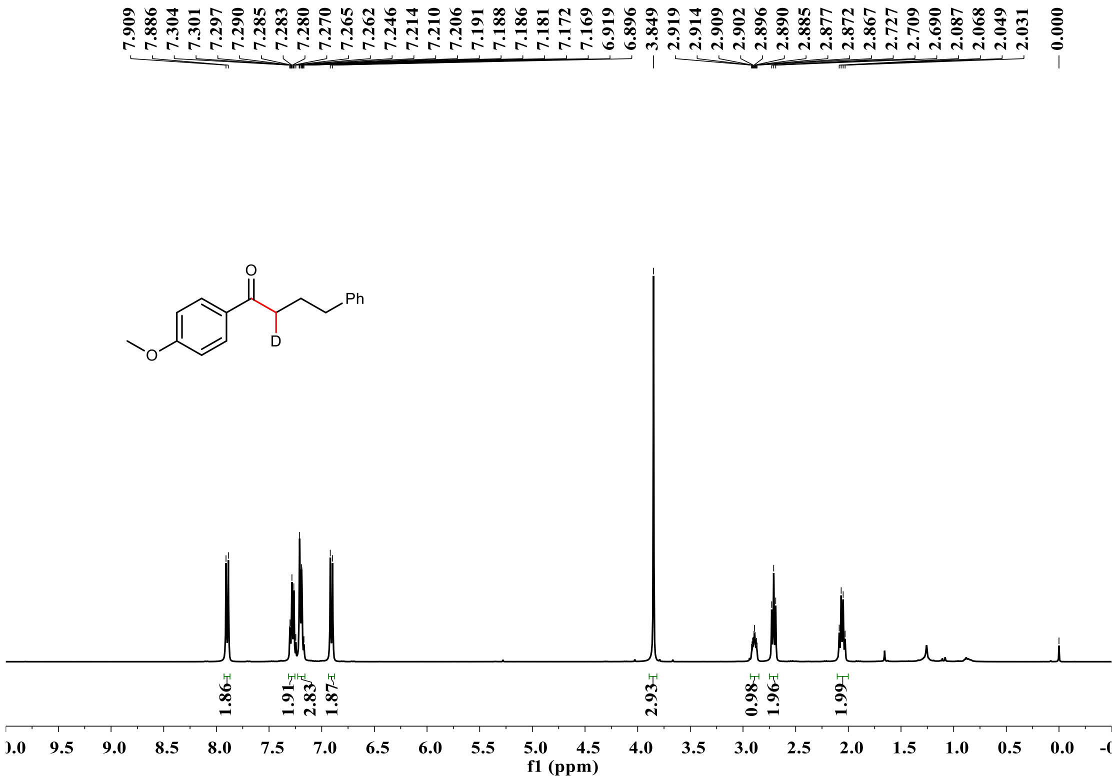

Supplementary Figure 85. ^{1}H NMR spectrum for **K34**

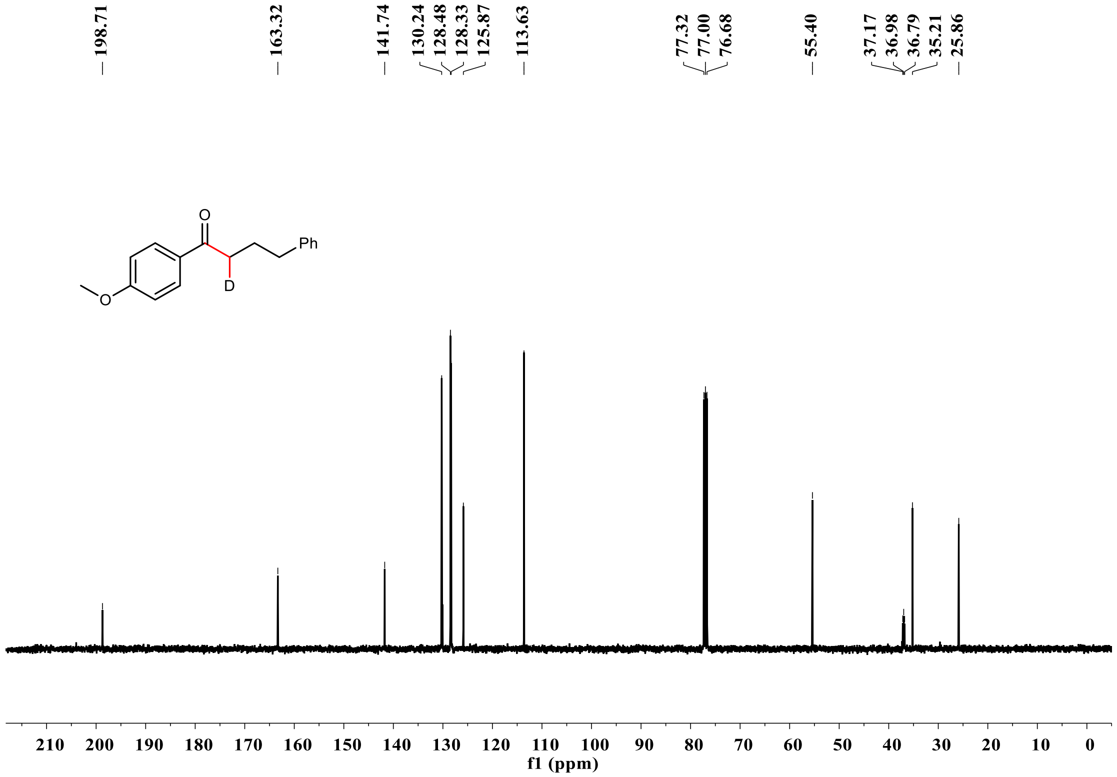

Supplementary Figure 86. ^{13}C NMR spectrum for **K34**

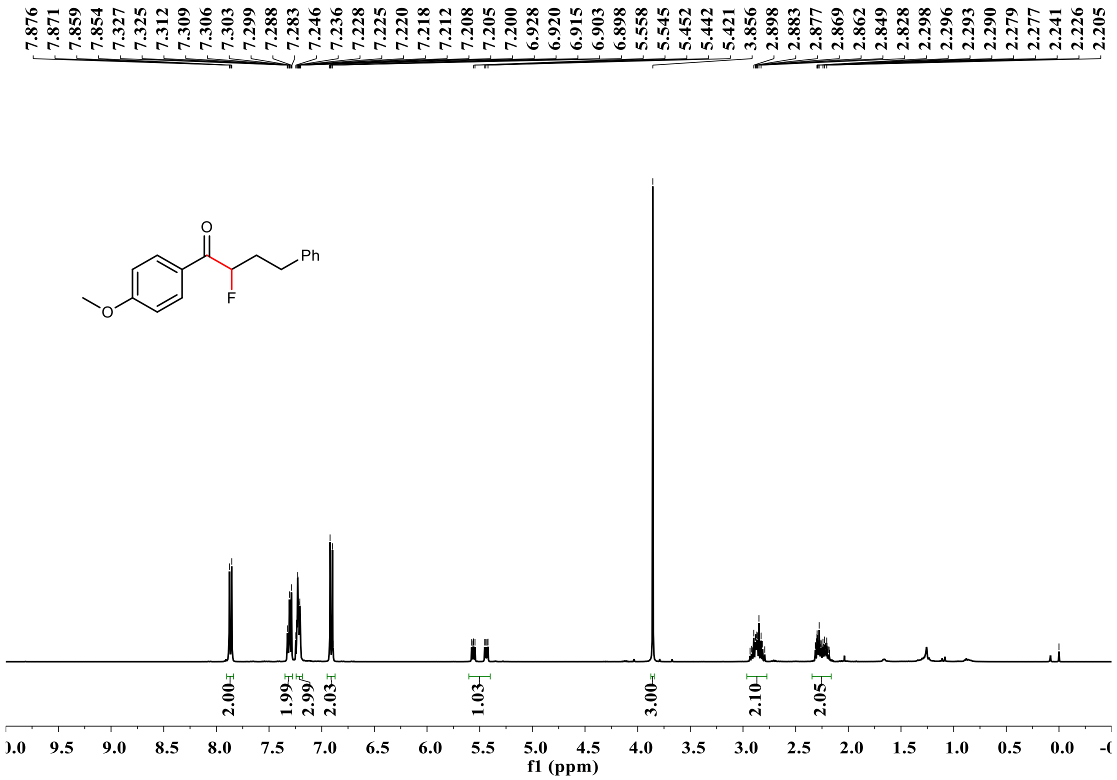

Supplementary Figure 87. ^{1}H NMR spectrum for **K35**

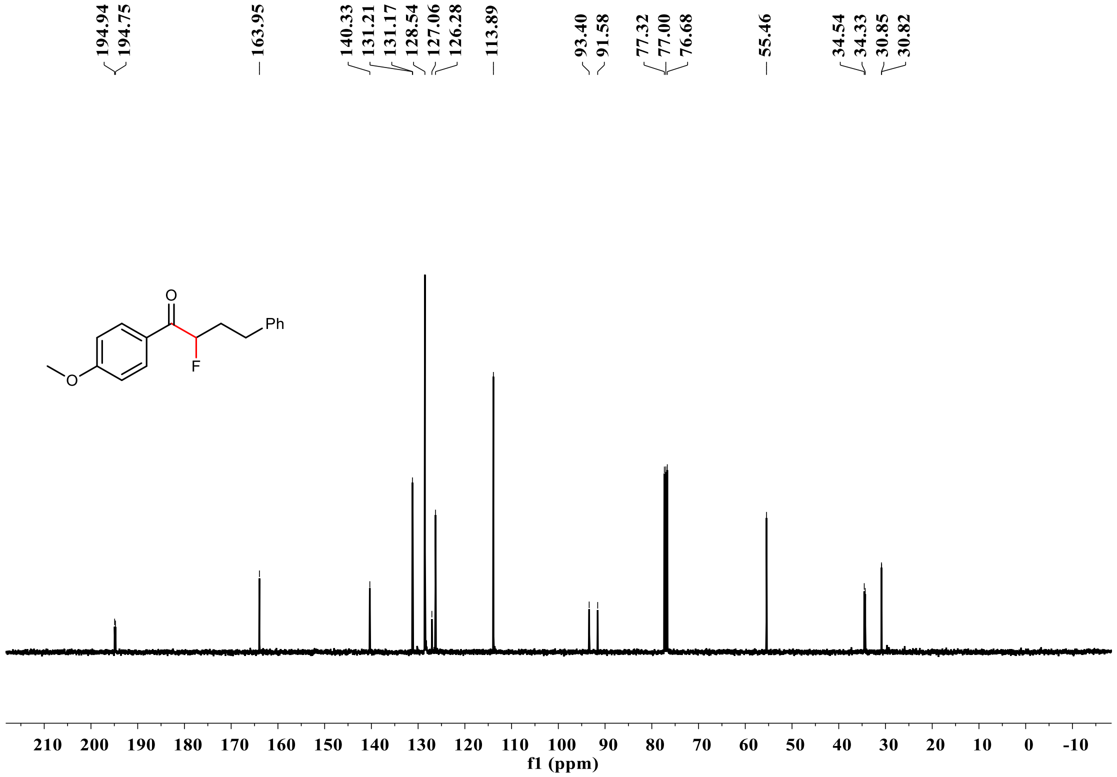

Supplementary Figure 88. ^{13}C NMR spectrum for **K35**

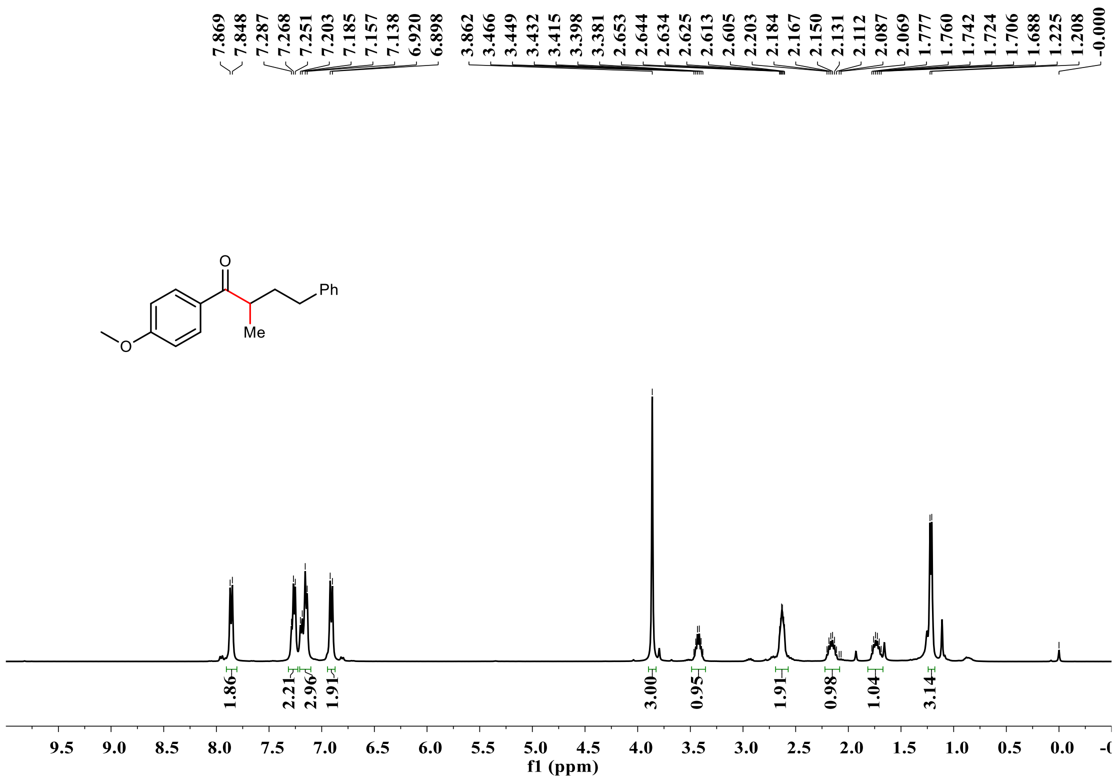

Supplementary Figure 89. ^{1}H NMR spectrum for **K36**

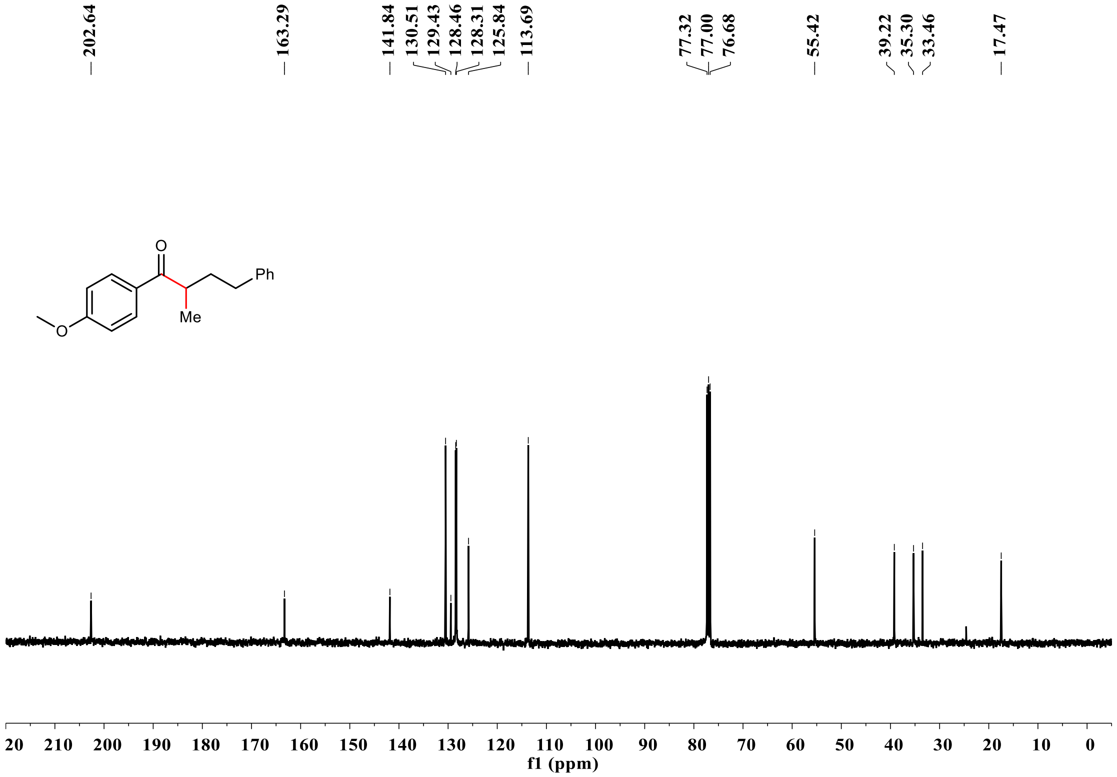

Supplementary Figure 90. ^{13}C NMR spectrum for **K36**

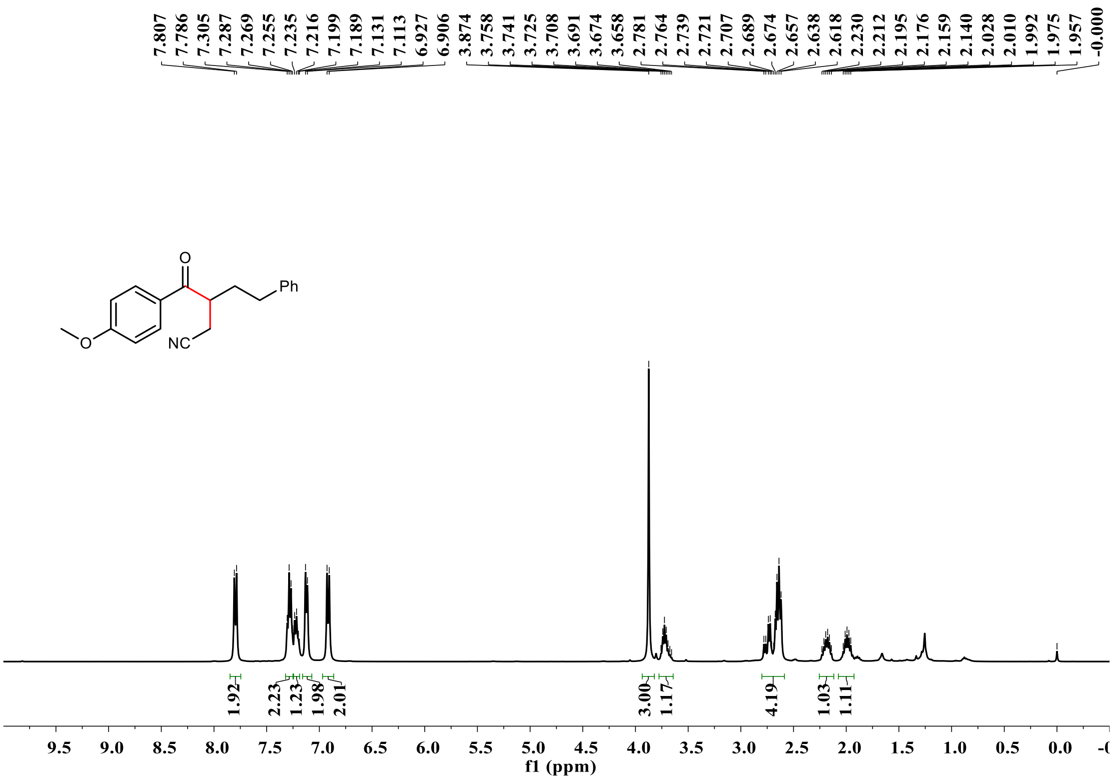

Supplementary Figure 91. ^{1}H NMR spectrum for **K37**

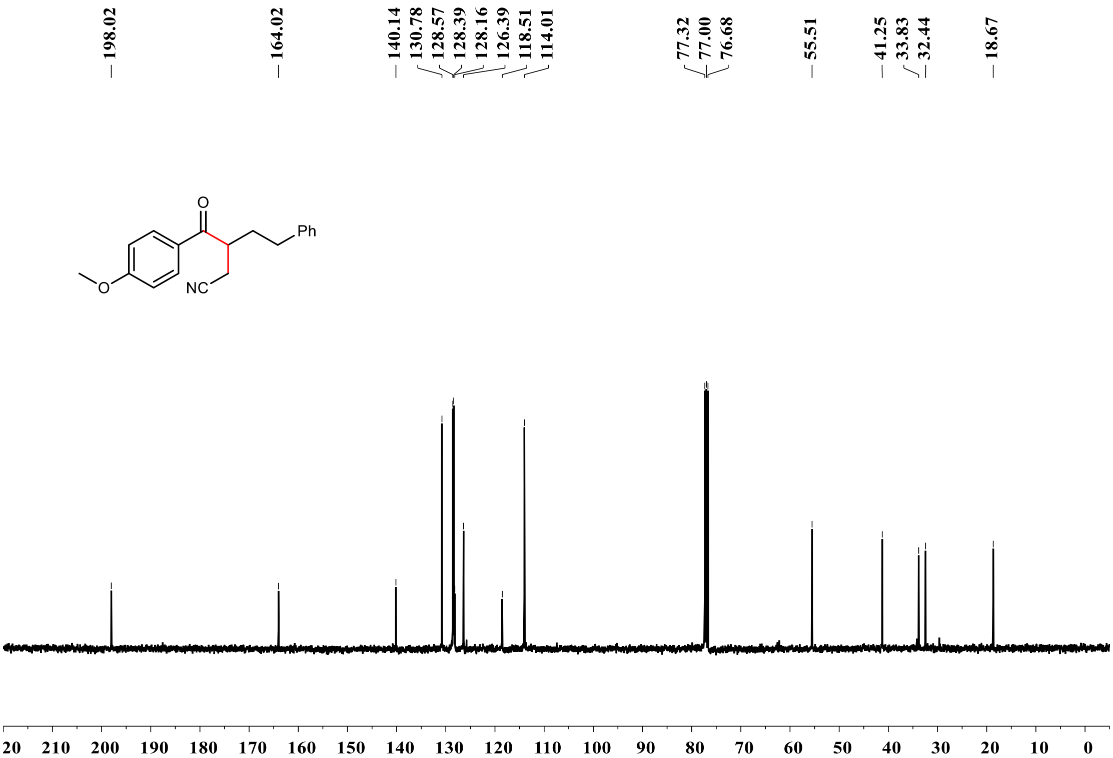

Supplementary Figure 92. ^{13}C NMR spectrum for **K37**

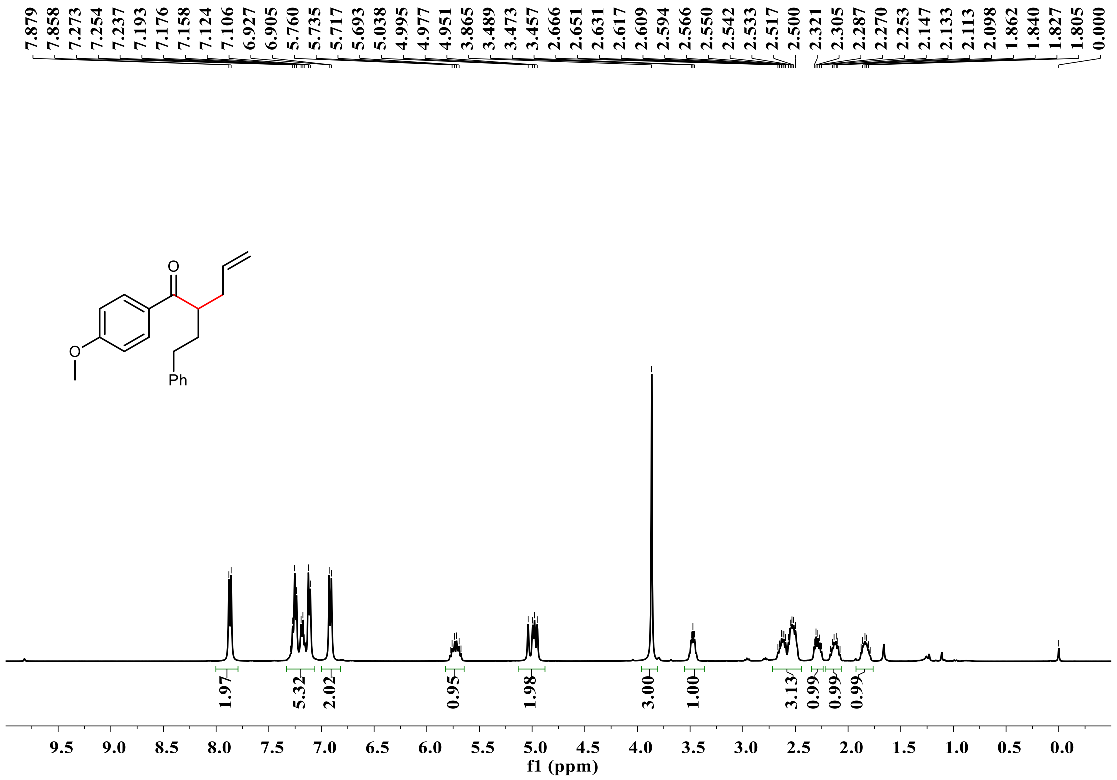

Supplementary Figure 93. ^{1}H NMR spectrum for **K38**

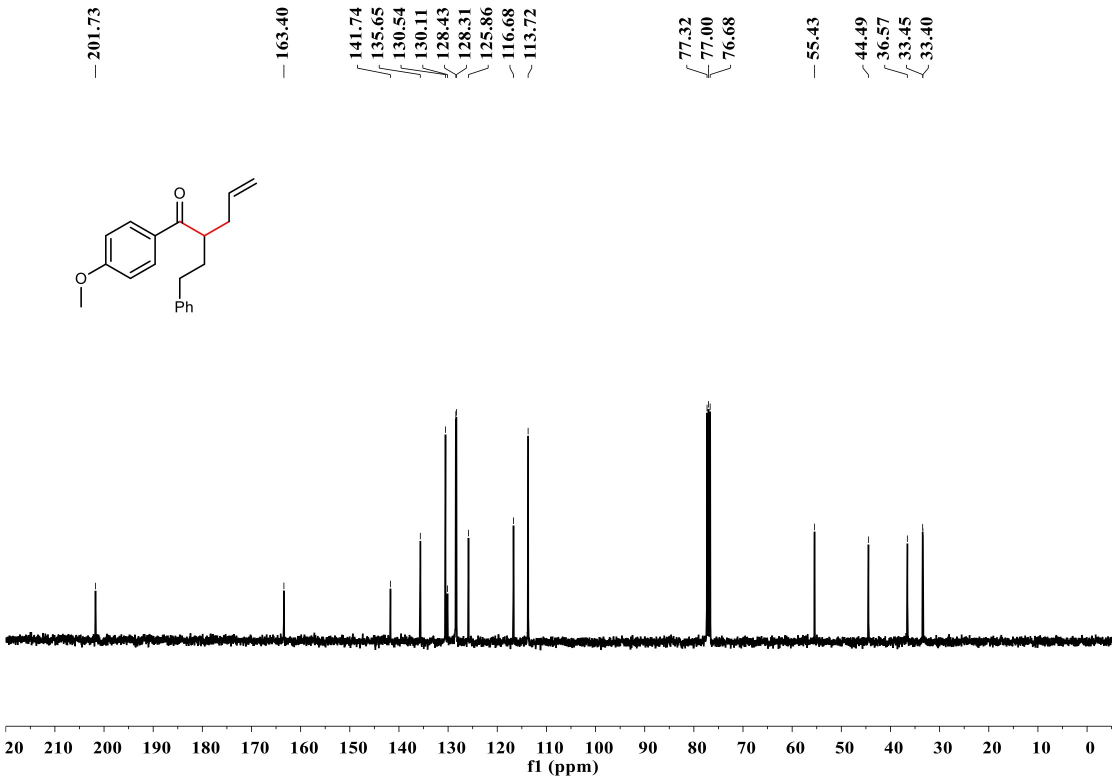

Supplementary Figure 94. ^{13}C NMR spectrum for **K38**

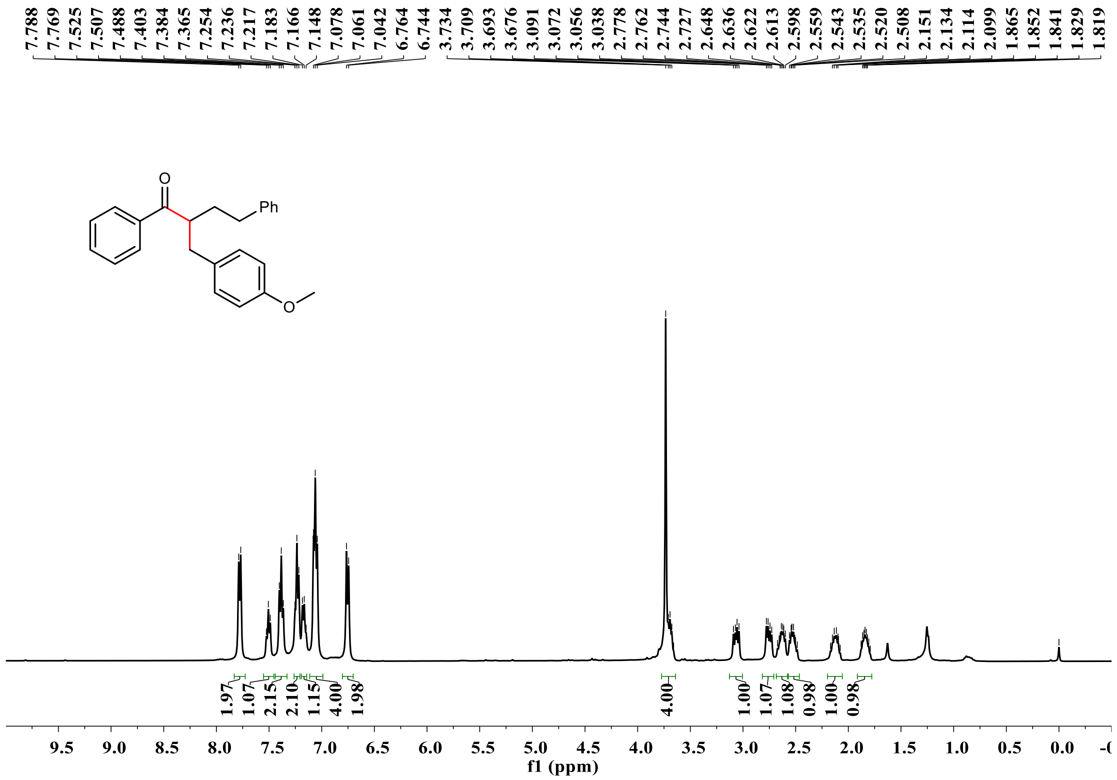

Supplementary Figure 95. ^{1}H NMR spectrum for **K39**

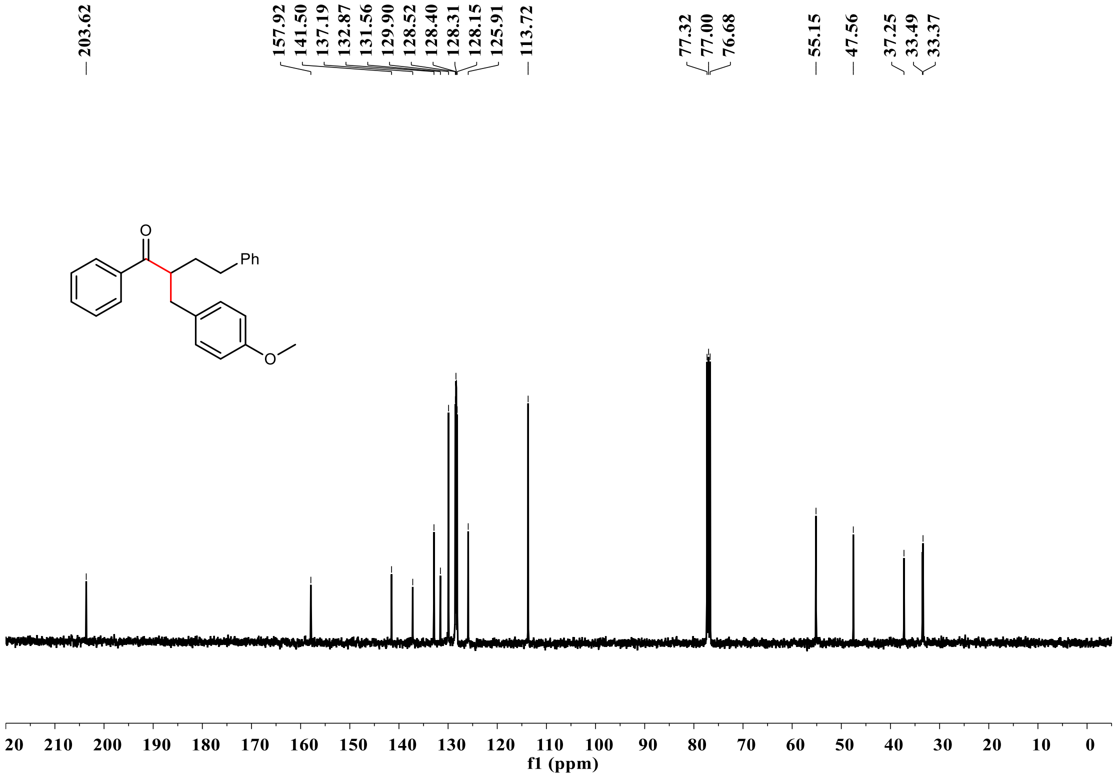

Supplementary Figure 96. ^{13}C NMR spectrum for **K39**

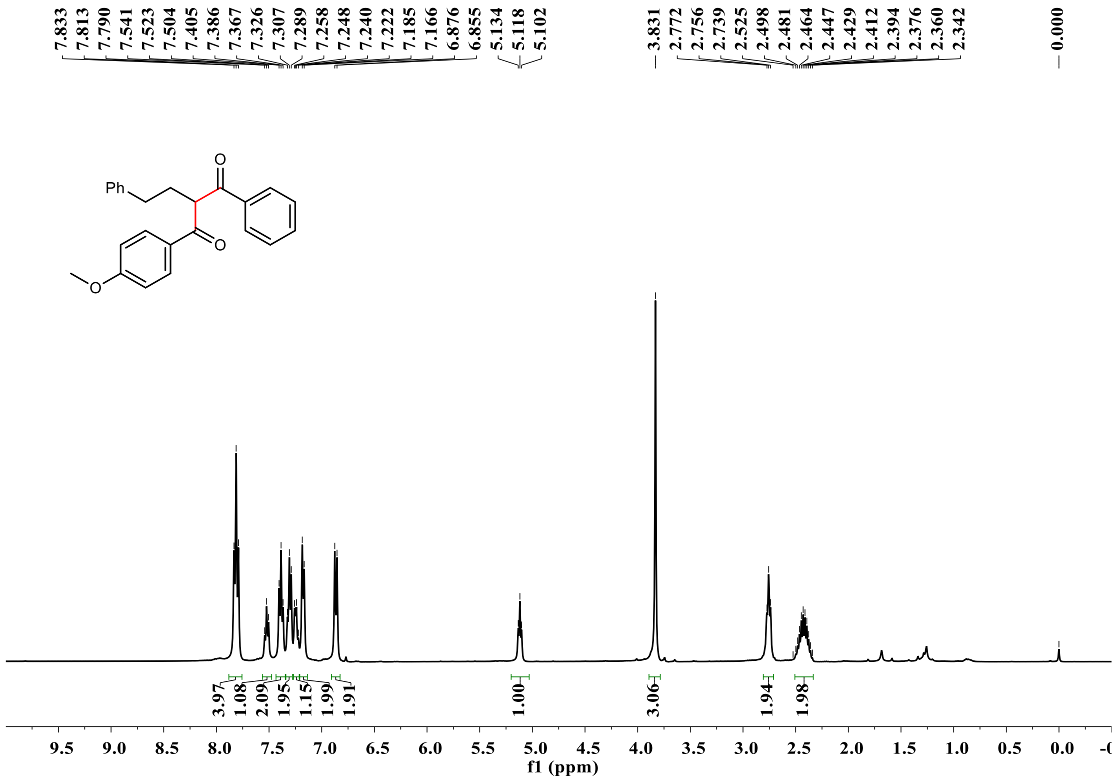

Supplementary Figure 97. ^{1}H NMR spectrum for **K40**

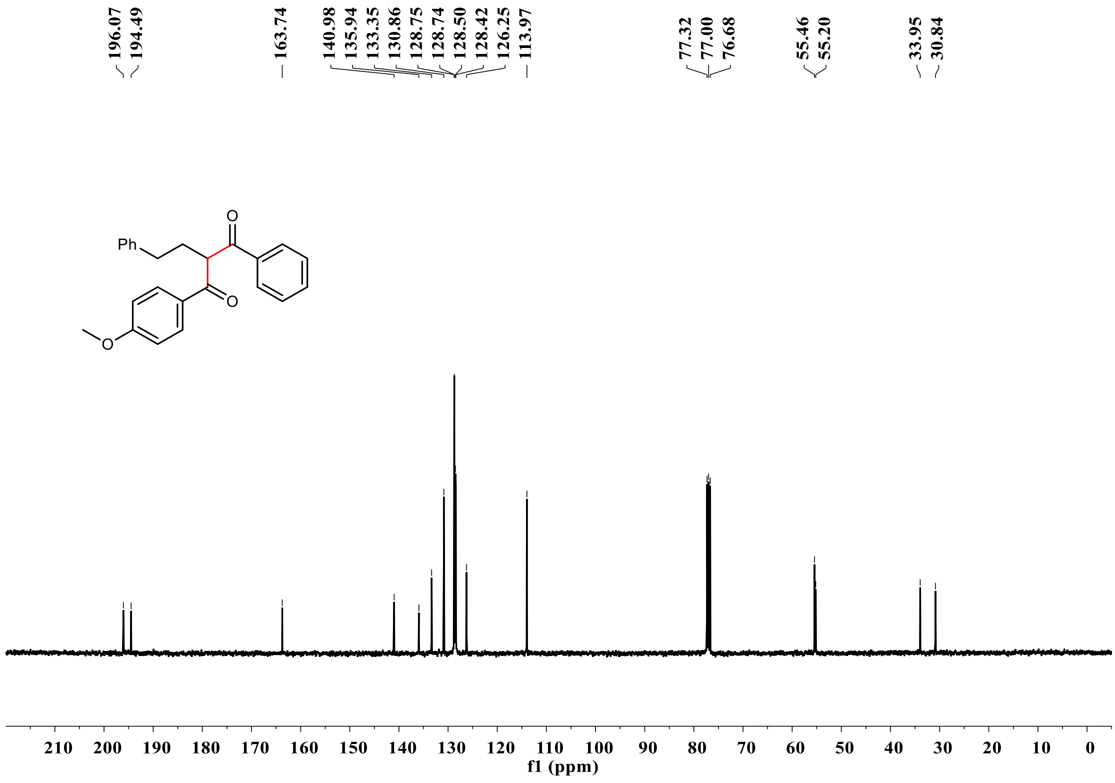

Supplementary Figure 98. ^{13}C NMR spectrum for **K40**

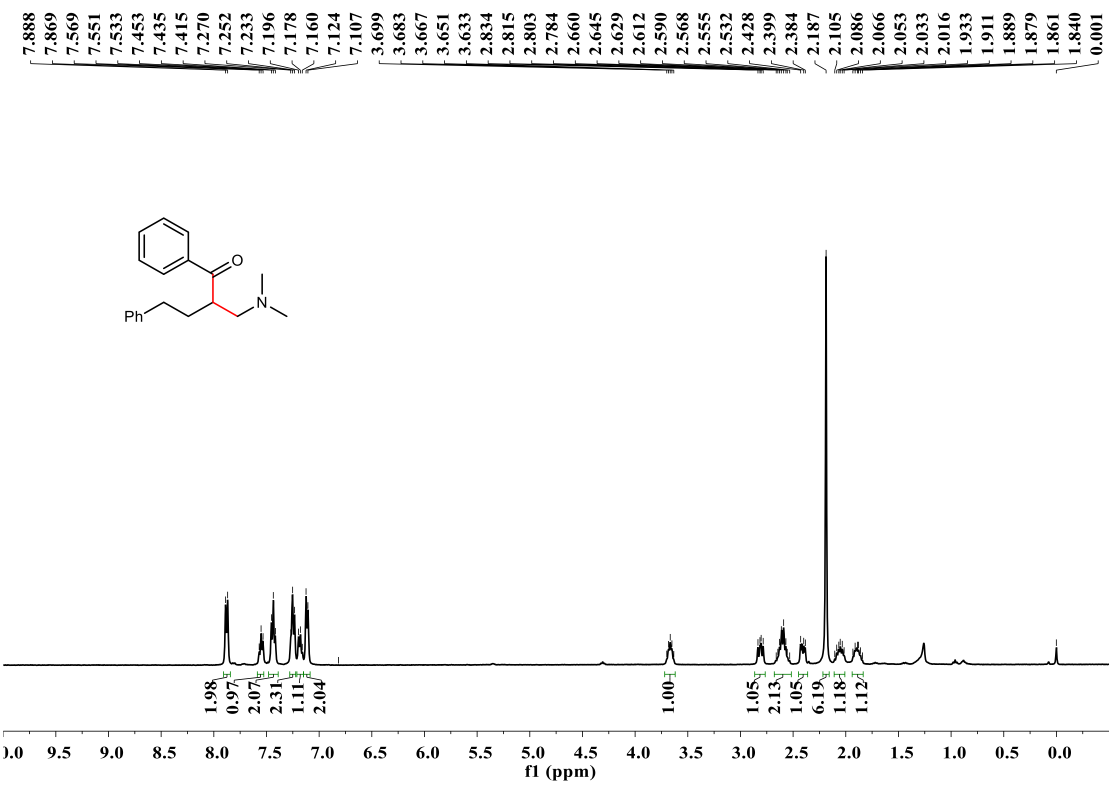

Supplementary Figure 99. ^{1}H NMR spectrum for **K41**

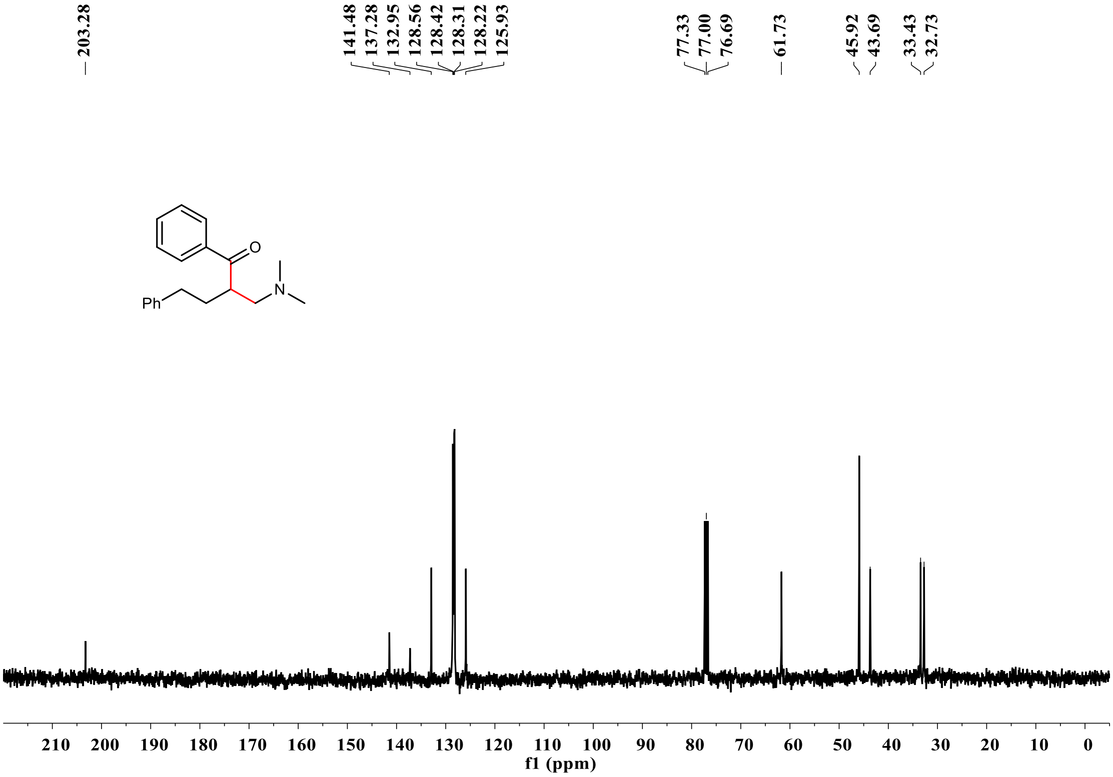

Supplementary Figure 100. ^{13}C NMR spectrum for **K41**

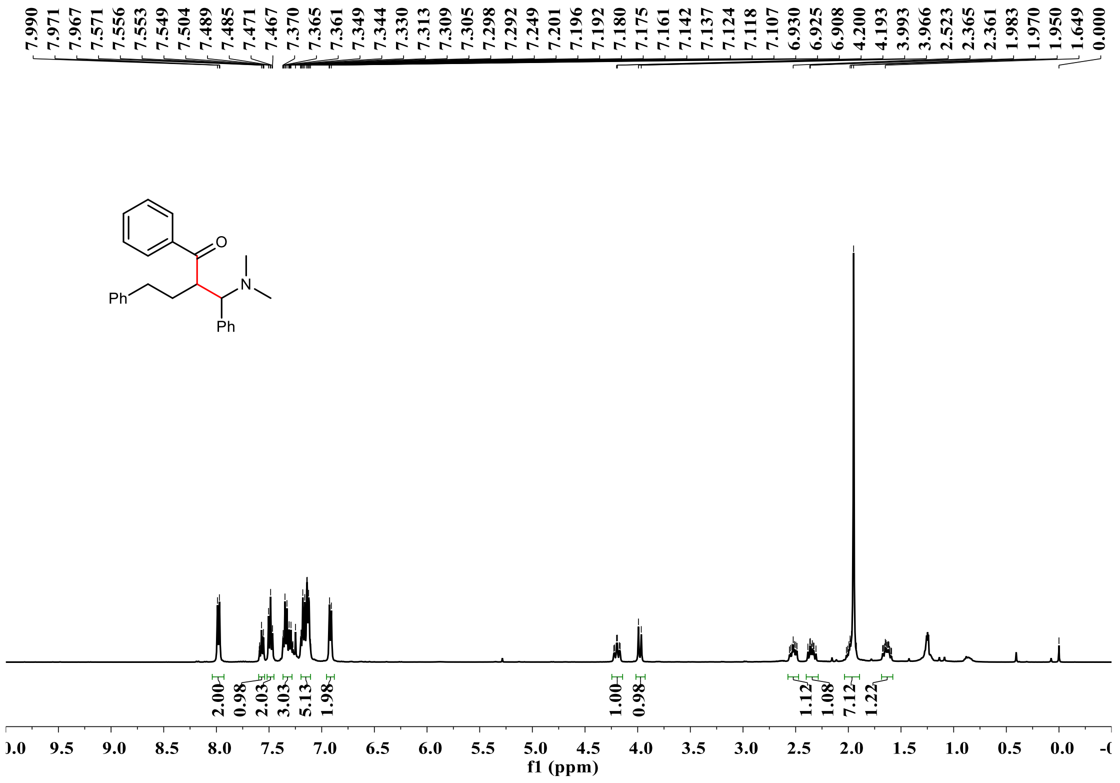

Supplementary Figure 101. ^{1}H NMR spectrum for **K42**

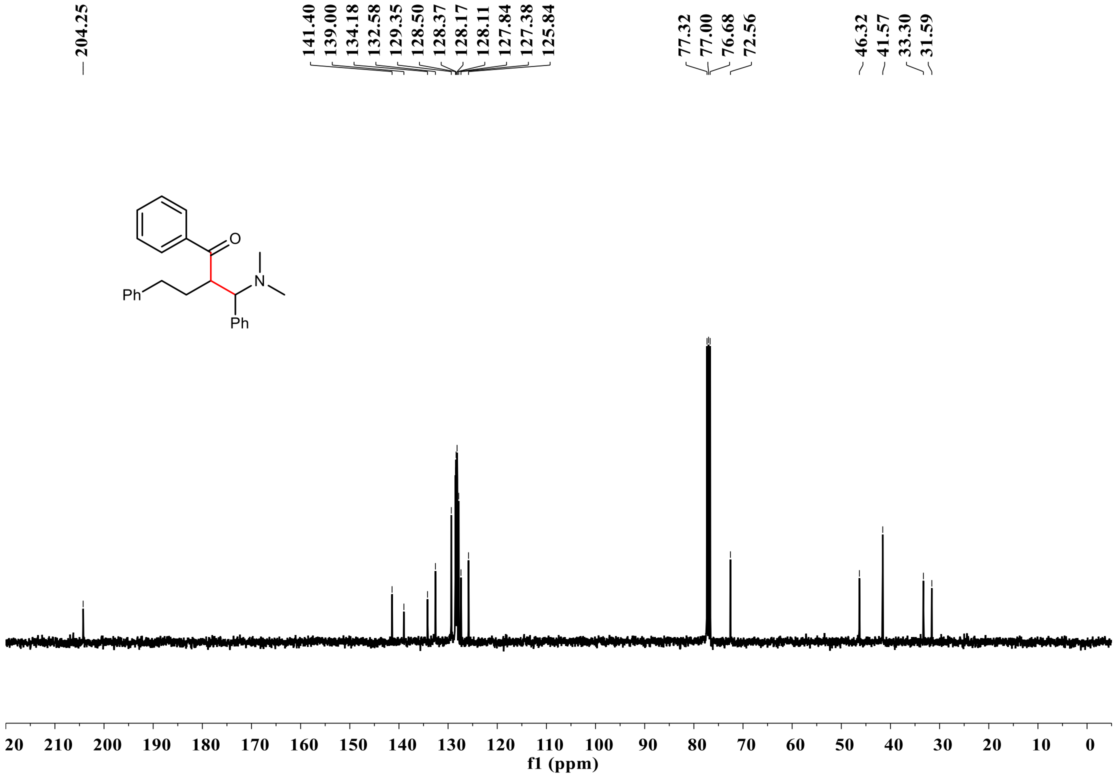

Supplementary Figure 102. ^{13}C NMR spectrum for **K42**

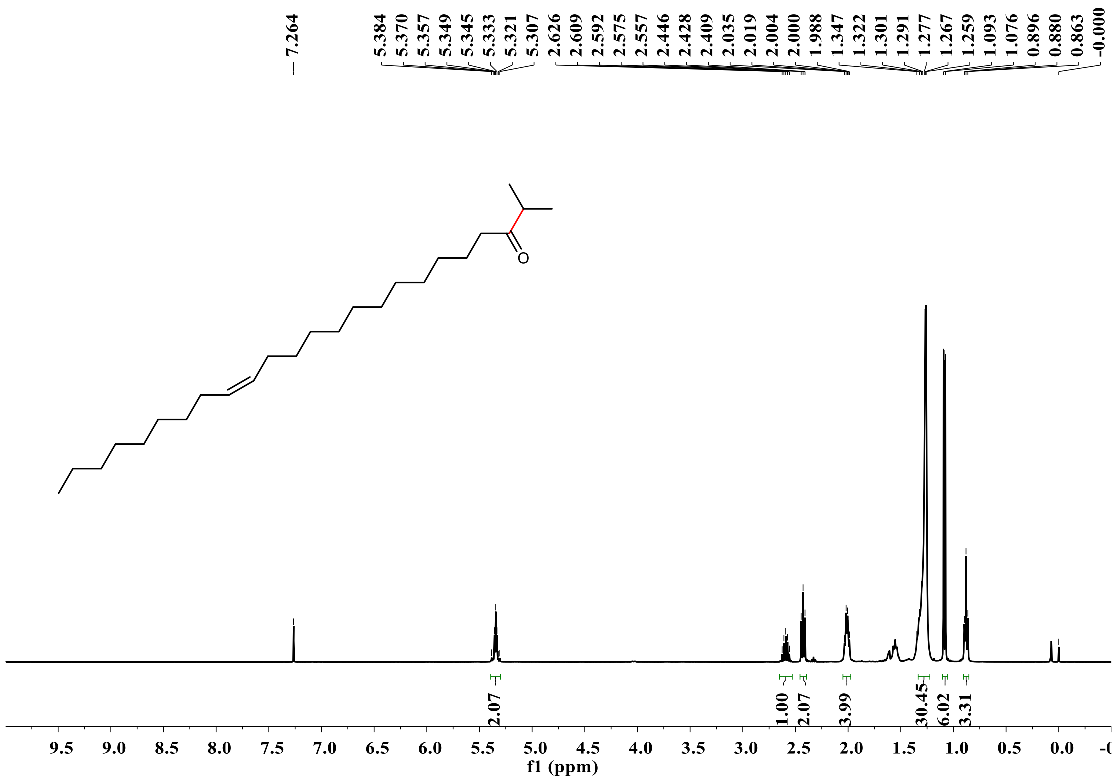

Supplementary Figure 103. ^{1}H NMR spectrum for **K43**

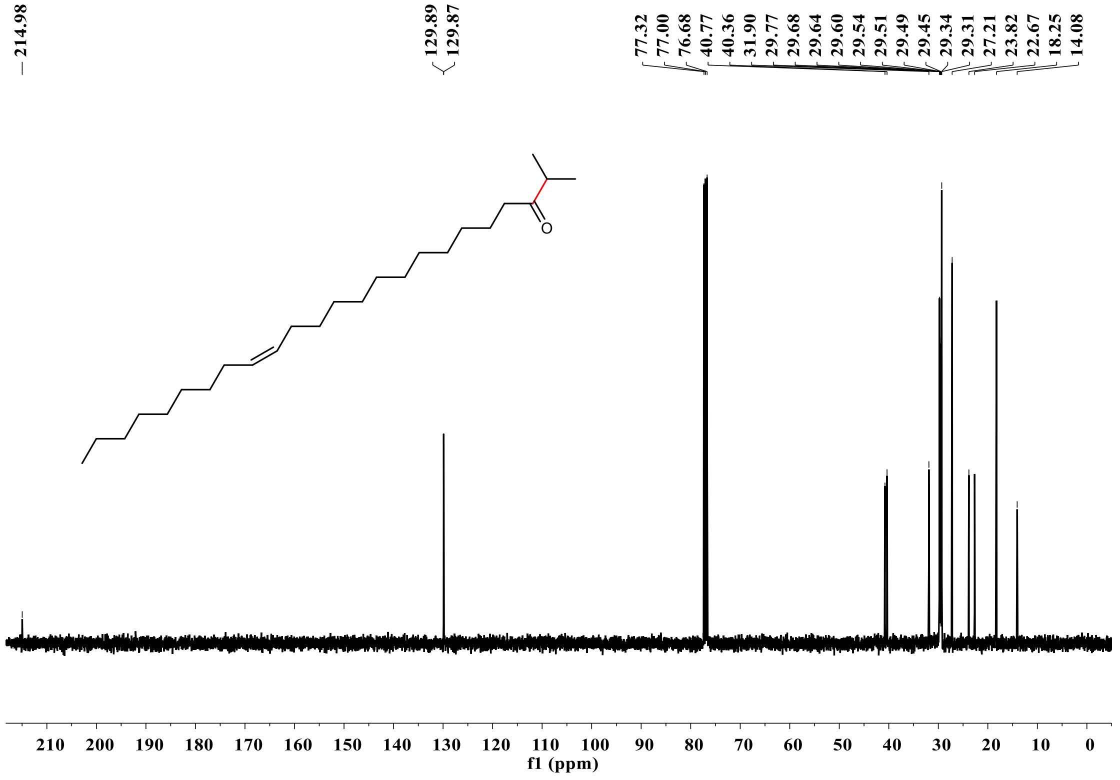

Supplementary Figure 104. ^{13}C NMR spectrum for **K43**

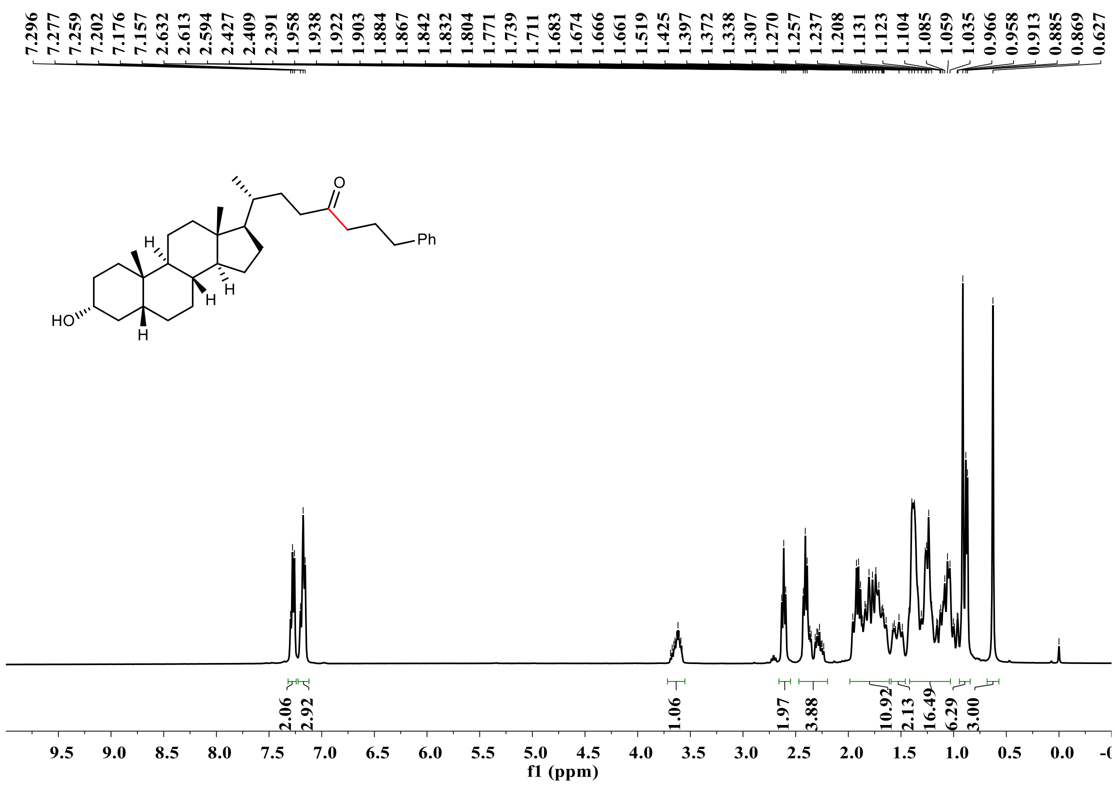

Supplementary Figure 105. ^{1}H NMR spectrum for **K44**

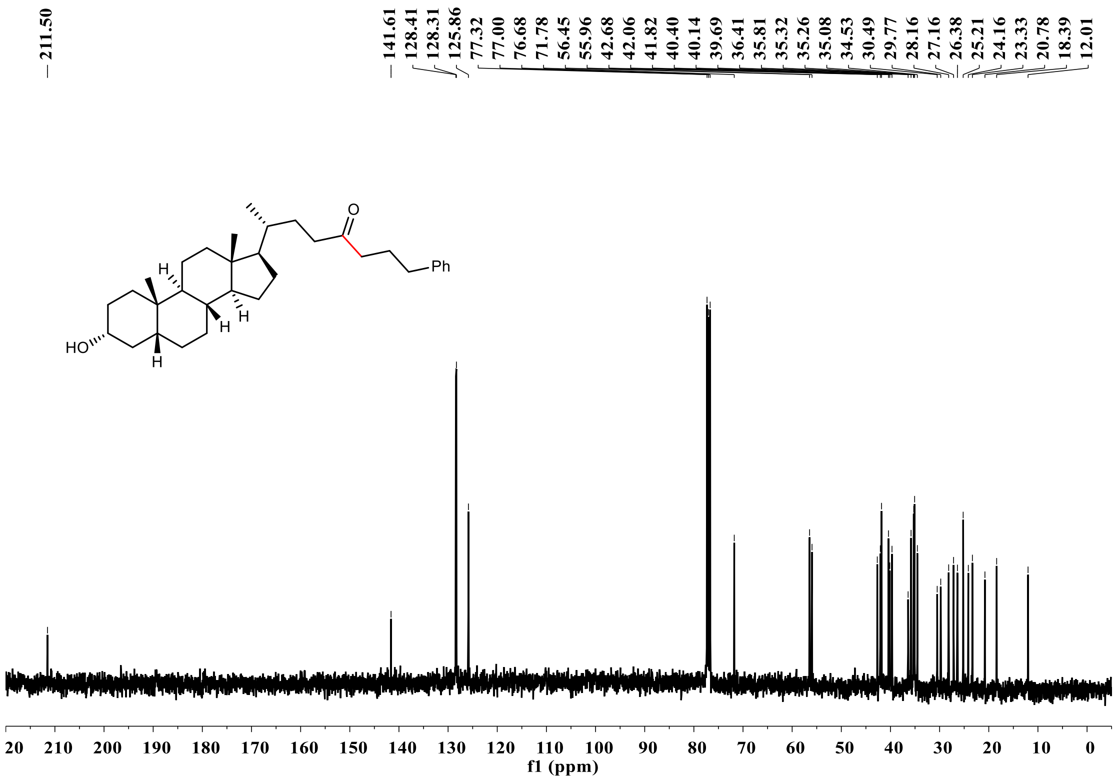

Supplementary Figure 106. ^{13}C NMR spectrum for **K44**

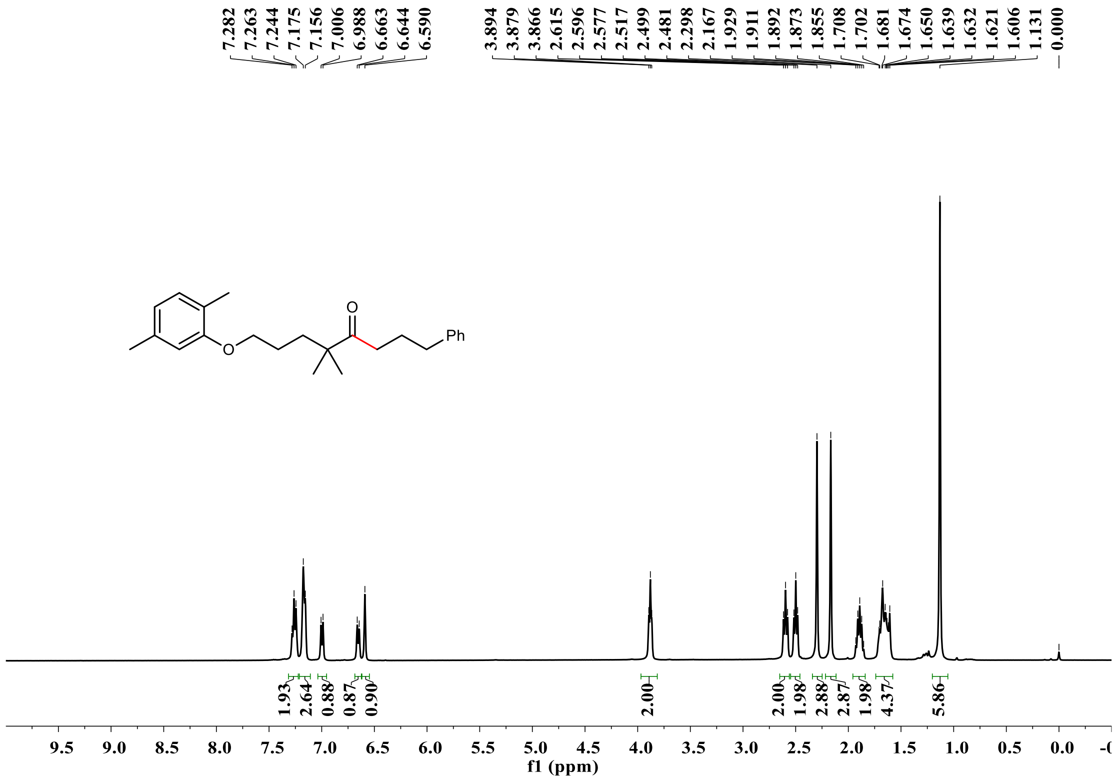

Supplementary Figure 107. ^{1}H NMR spectrum for **K45**

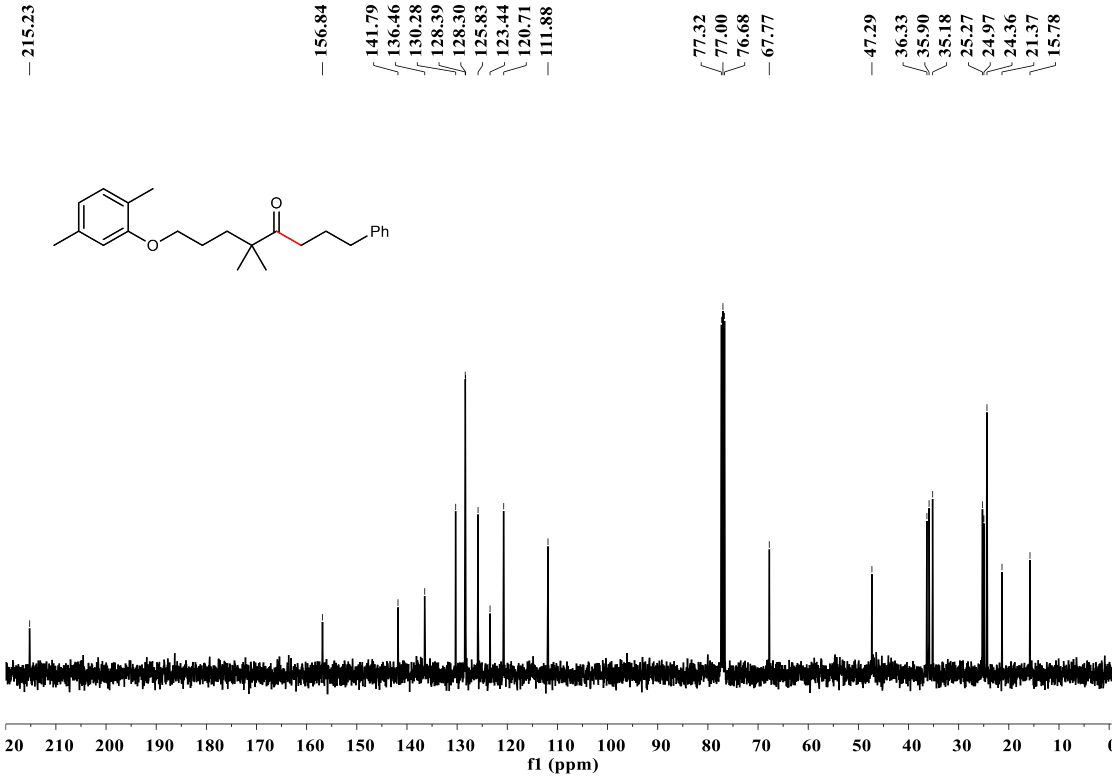

Supplementary Figure 108. ^{13}C NMR spectrum for **K45**

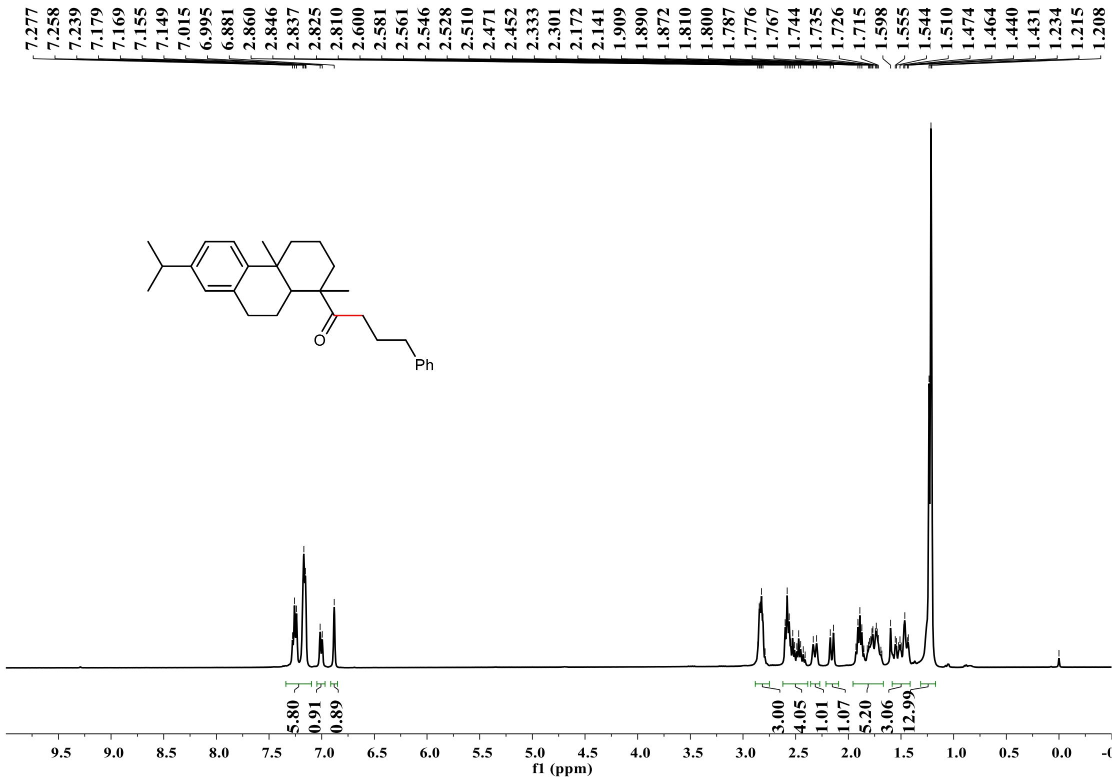

Supplementary Figure 109. ^{1}H NMR spectrum for **K46**

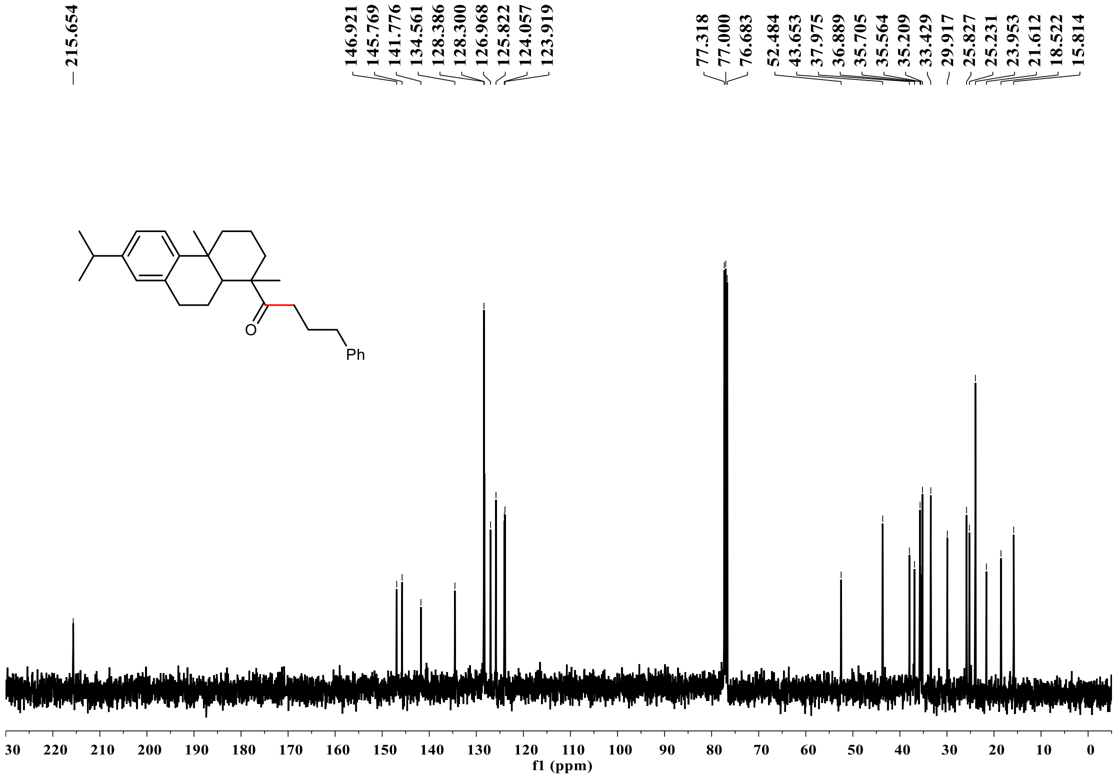

Supplementary Figure 110. ^{13}C NMR spectrum for **K46**

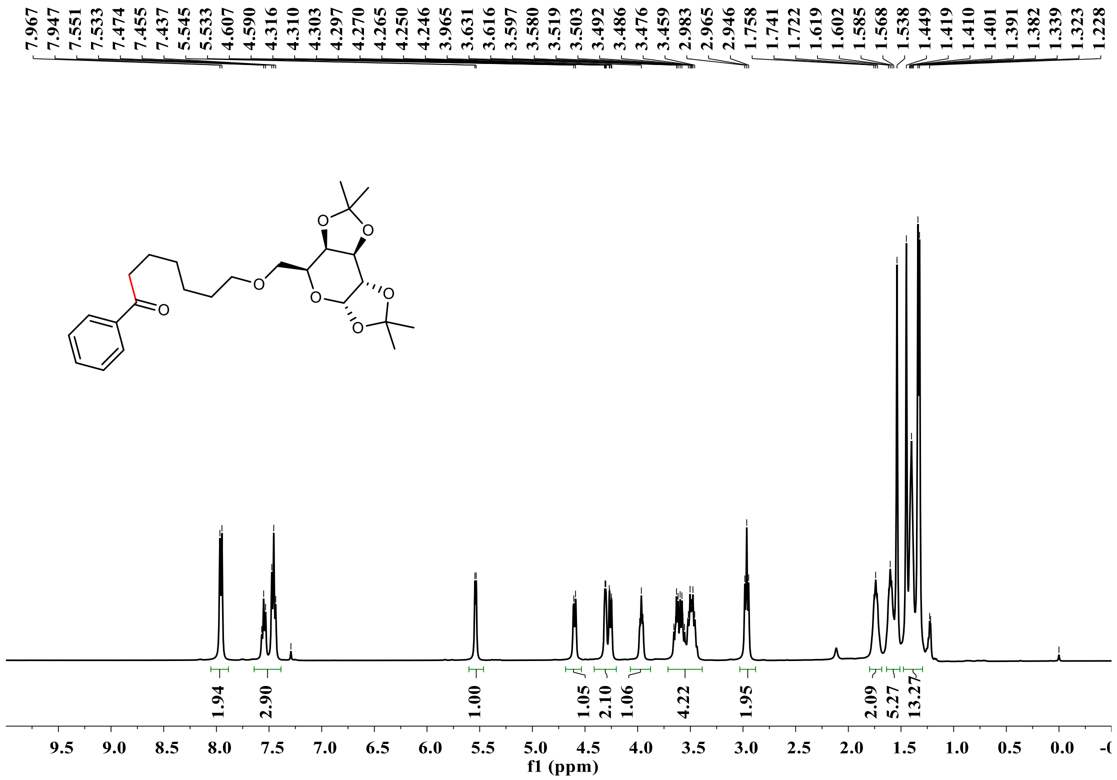

Supplementary Figure 111. ^{1}H NMR spectrum for **K47**

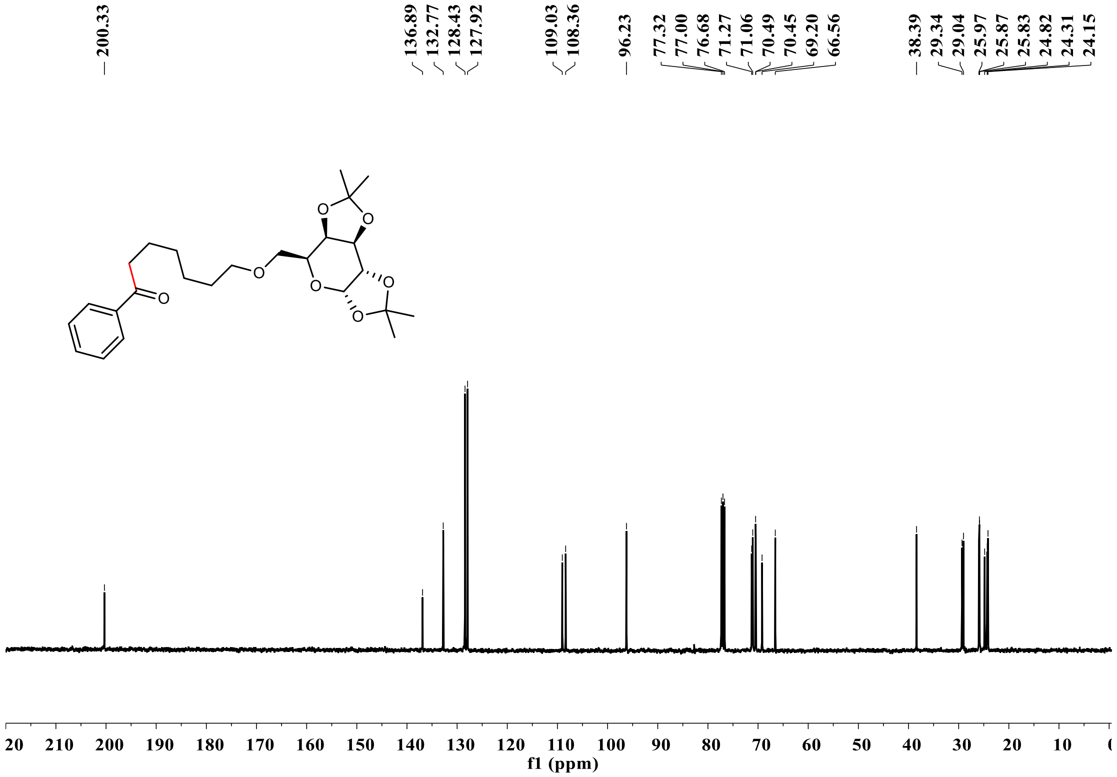

Supplementary Figure 112. ^{13}C NMR spectrum for **K47**

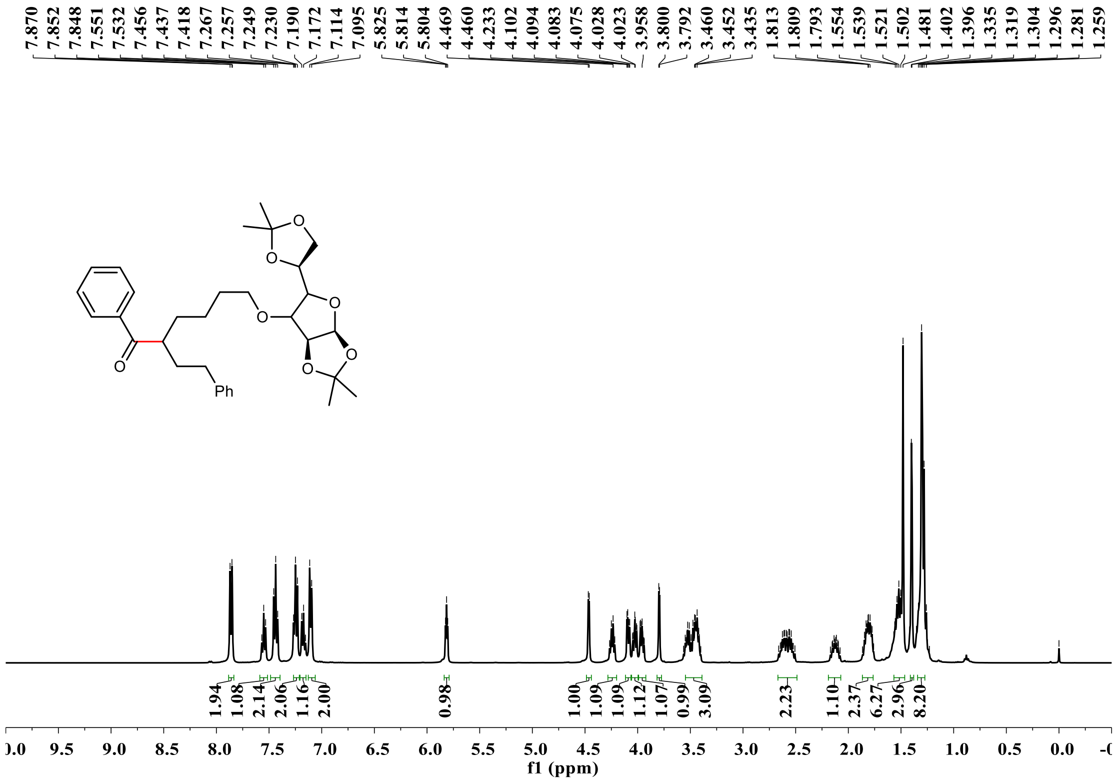

Supplementary Figure 113. ^{1}H NMR spectrum for **K48**

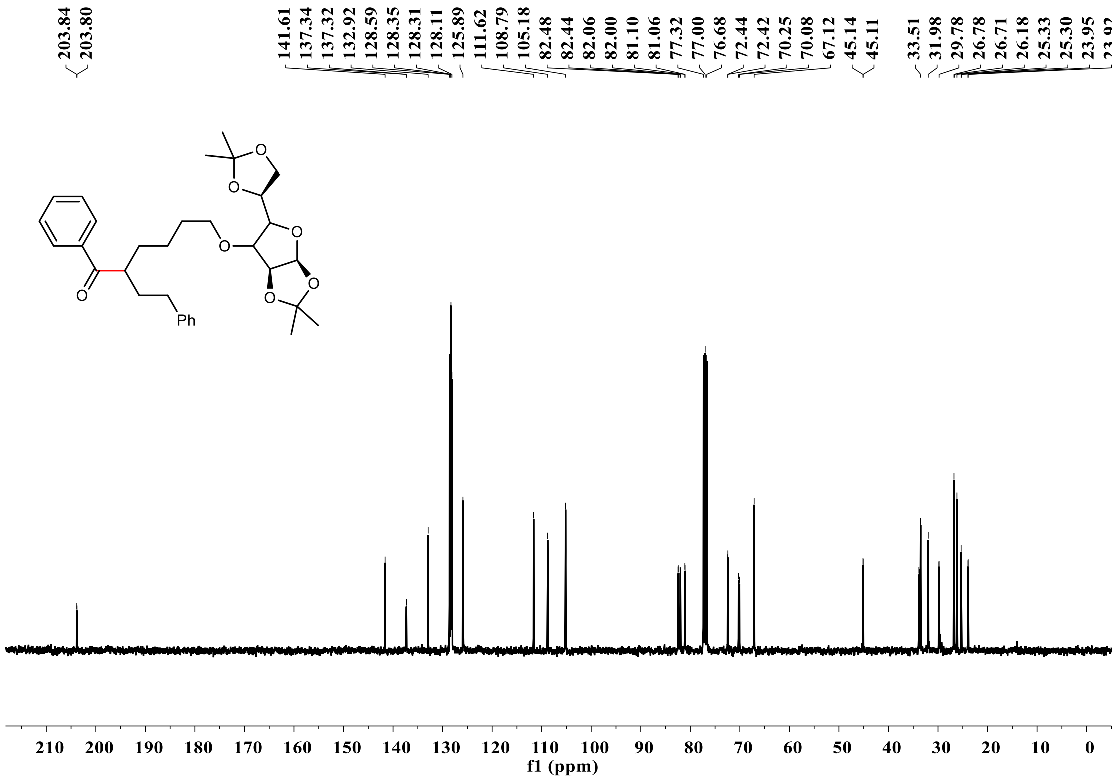

Supplementary Figure 114. ^{13}C NMR spectrum for **K48**

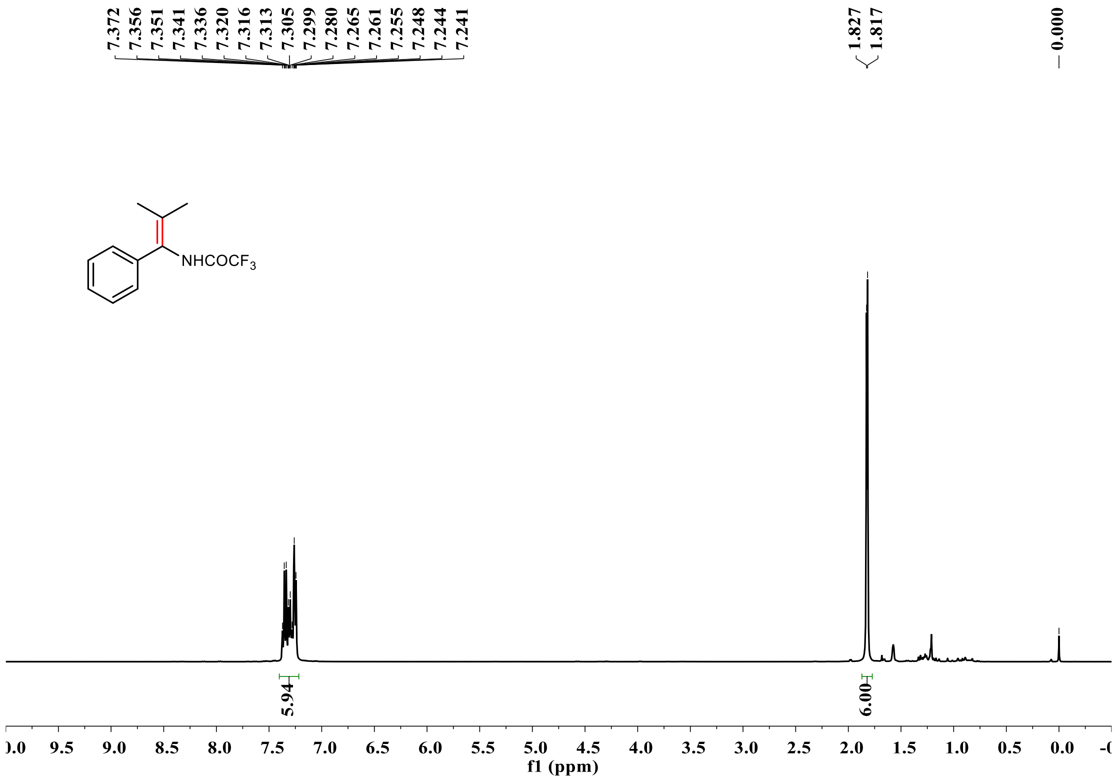

Supplementary Figure 115. ^{1}H NMR spectrum for **EA1**

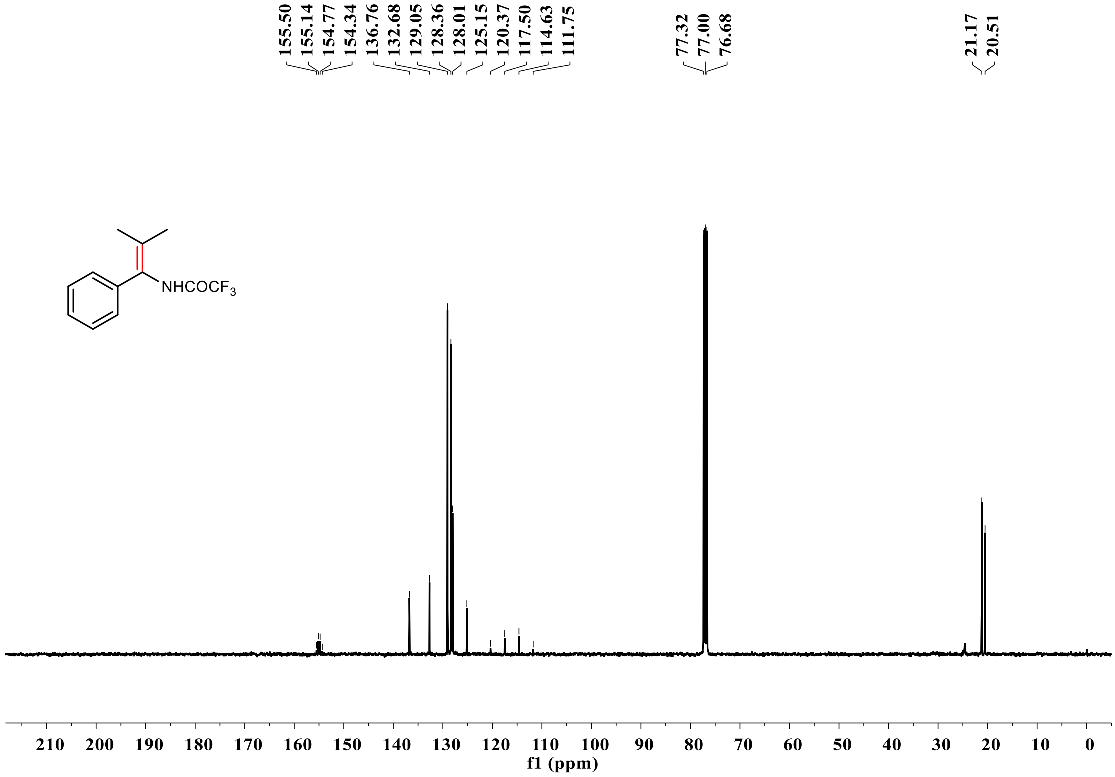

Supplementary Figure 116. ^{13}C NMR spectrum for **EA1**

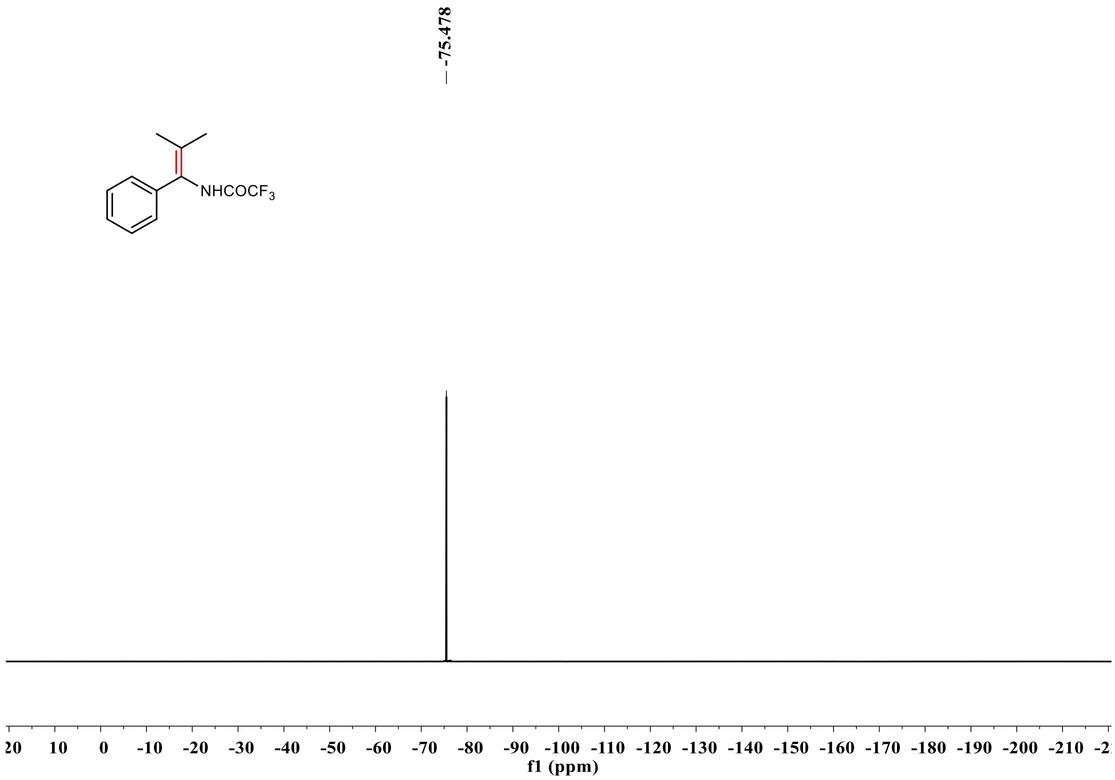

Supplementary Figure 117. ^{19}F NMR spectrum for **EA1**

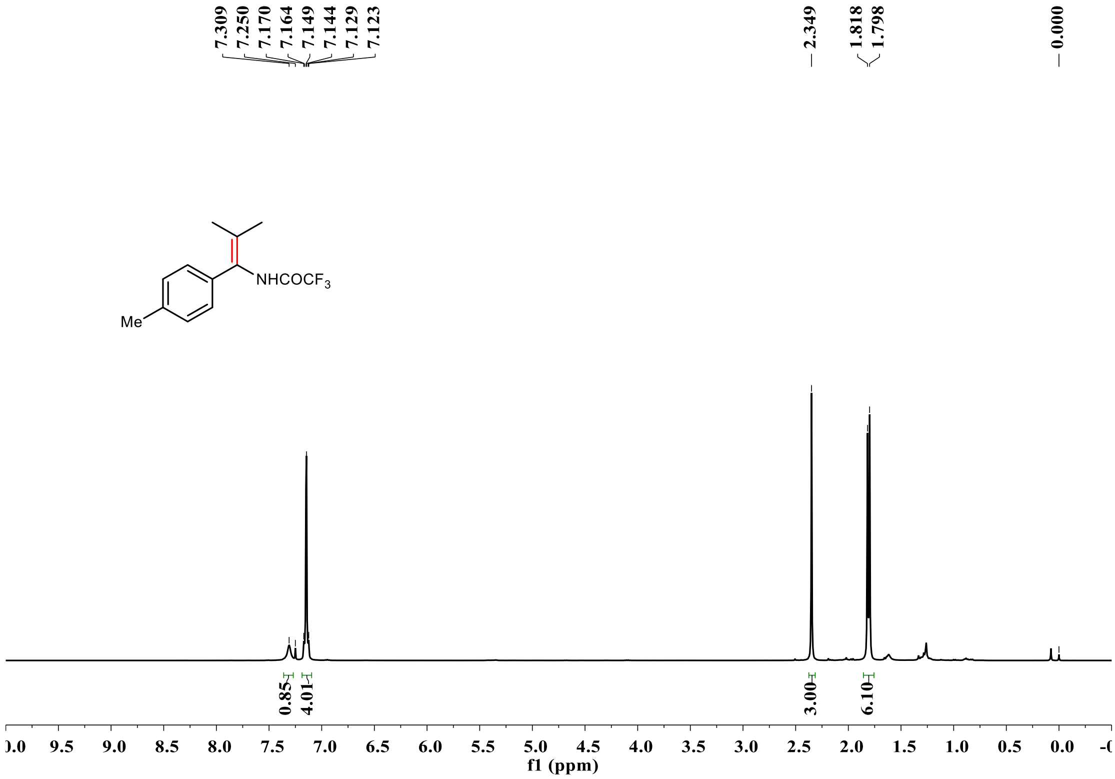

Supplementary Figure 118. ^{1}H NMR spectrum for **EA2**

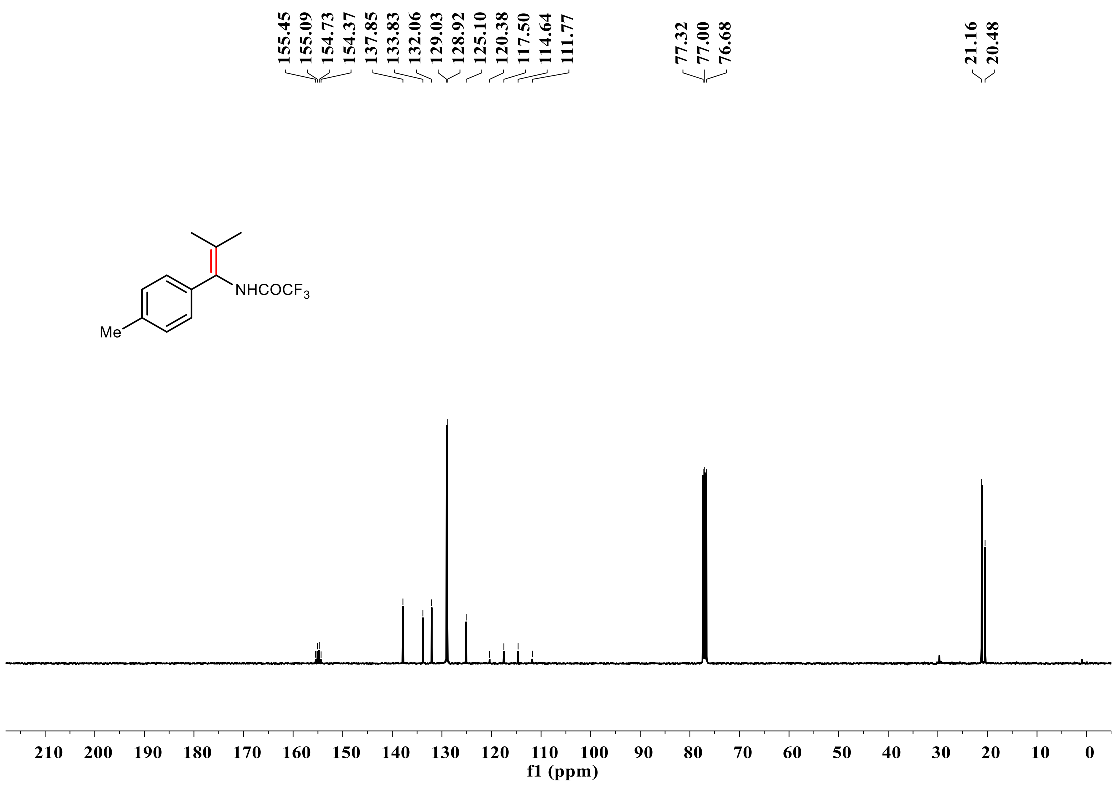

Supplementary Figure 119. ^{13}C NMR spectrum for **EA2**

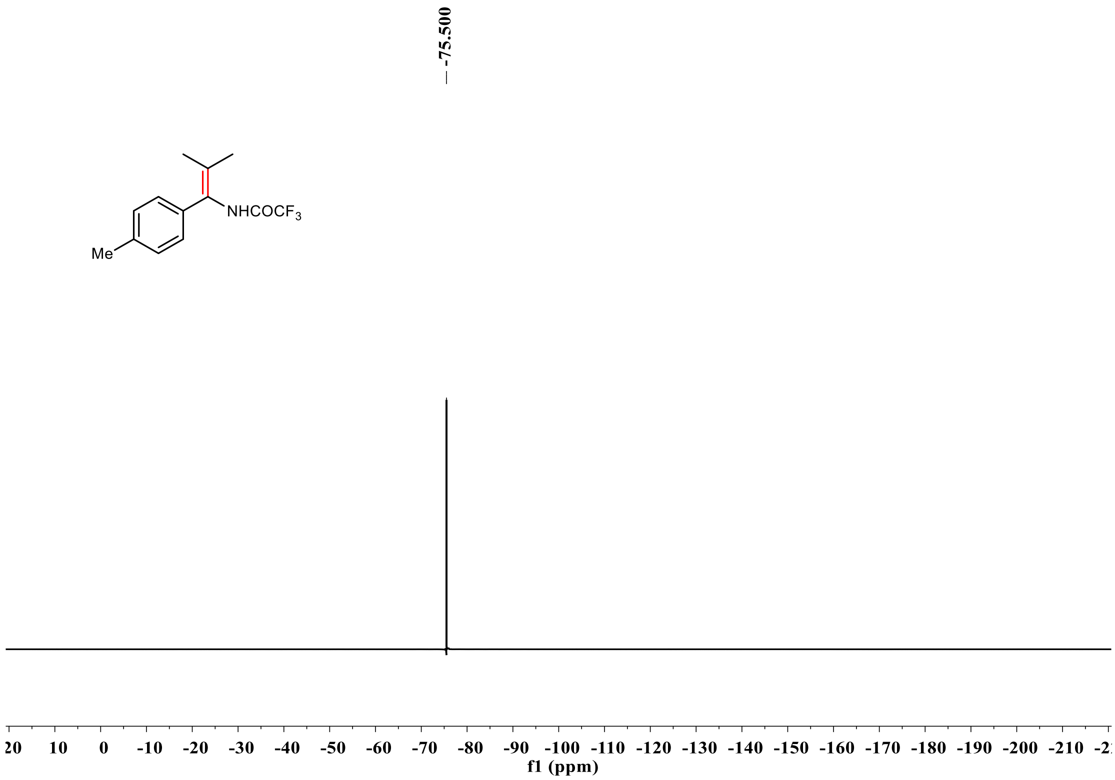

Supplementary Figure 120. ^{19}F NMR spectrum for **EA2**

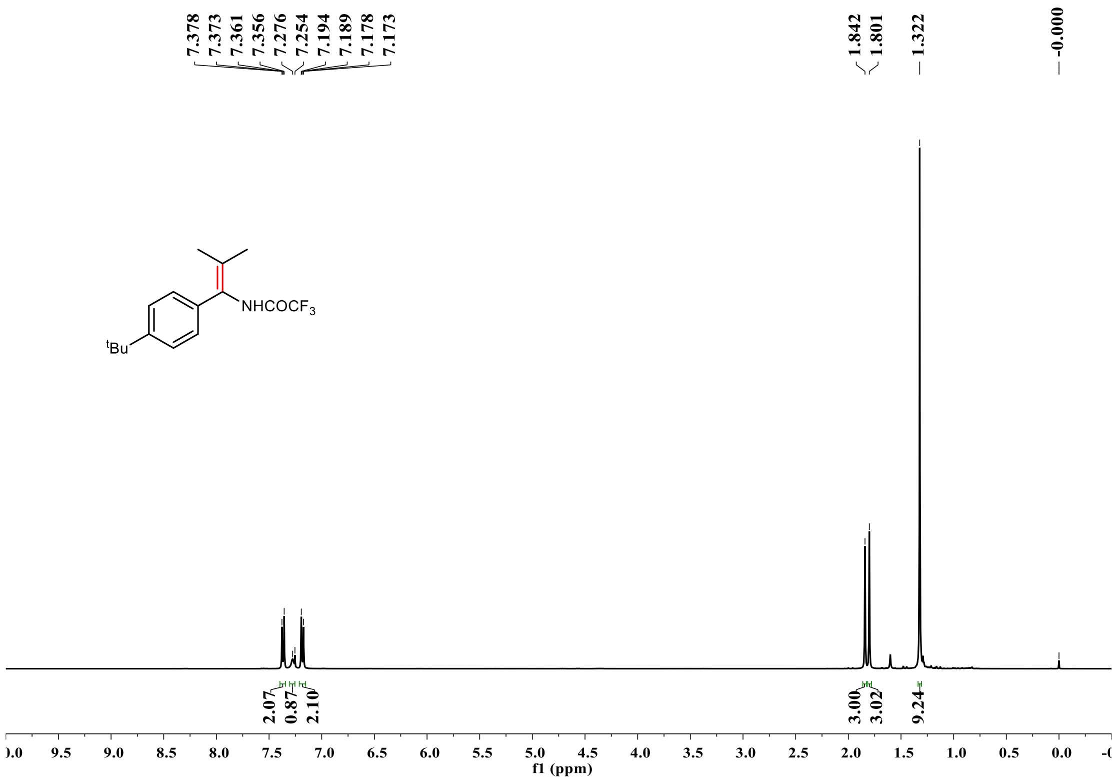

Supplementary Figure 121. ^{1}H NMR spectrum for **EA3**

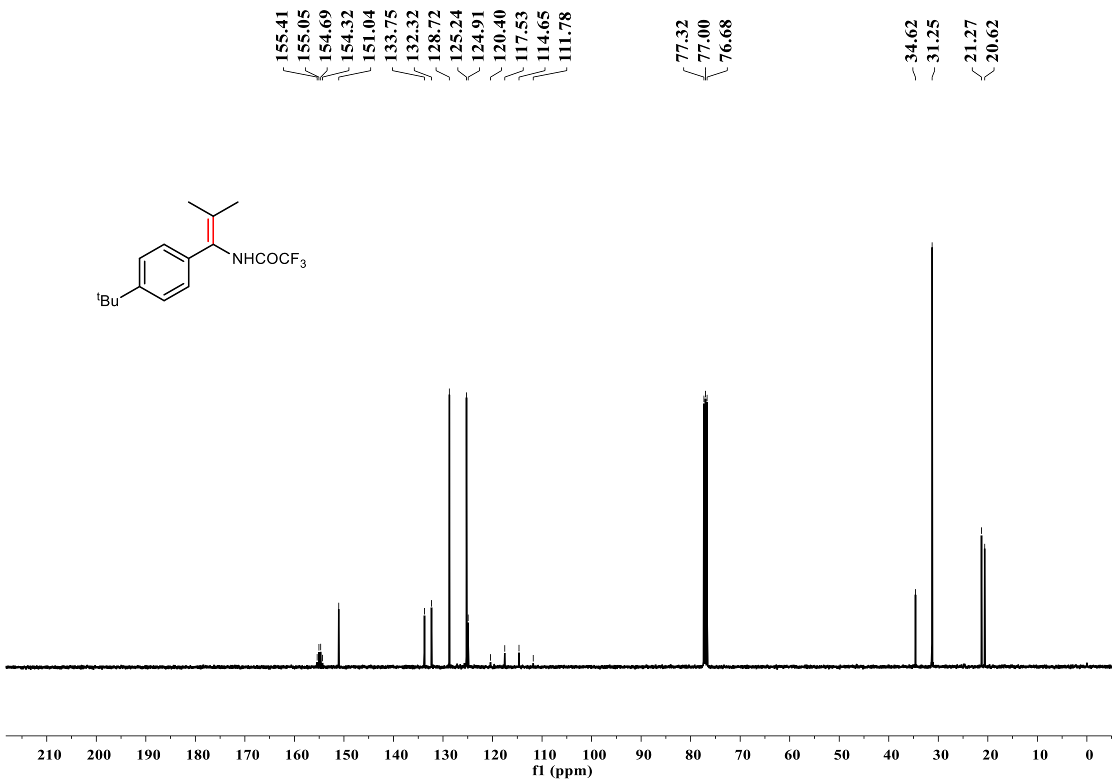

Supplementary Figure 122. ^{13}C NMR spectrum for **EA3**

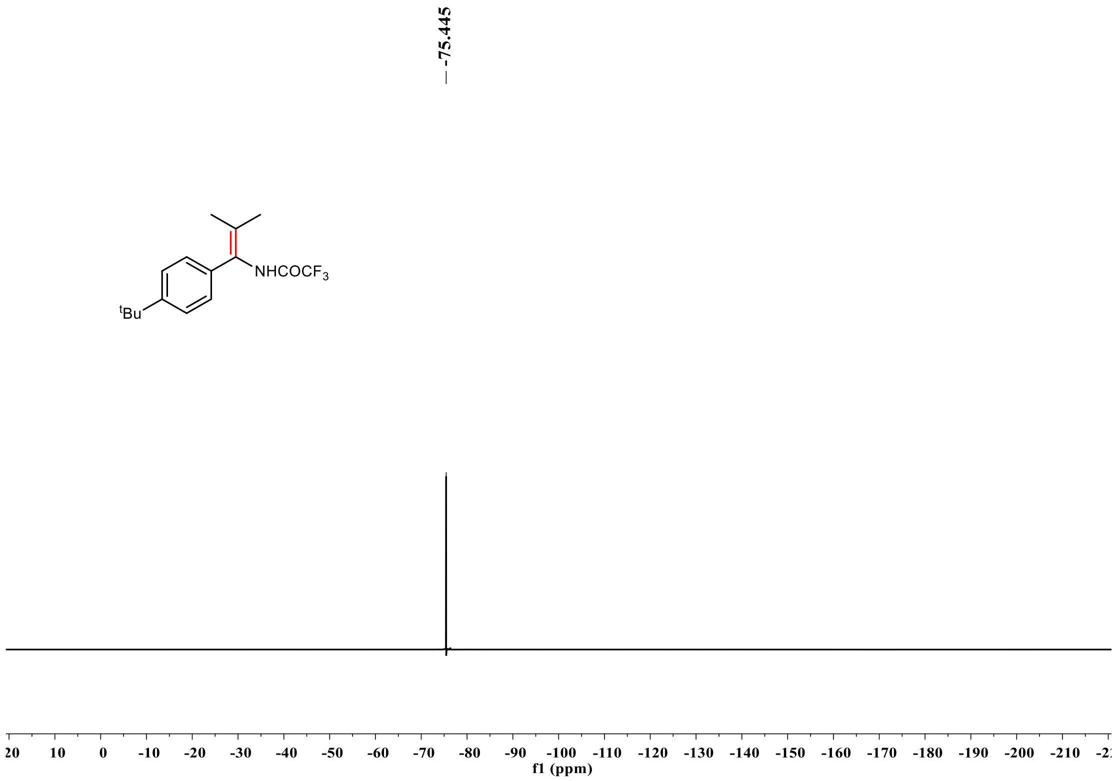

Supplementary Figure 123. ^{19}F NMR spectrum for **EA3**

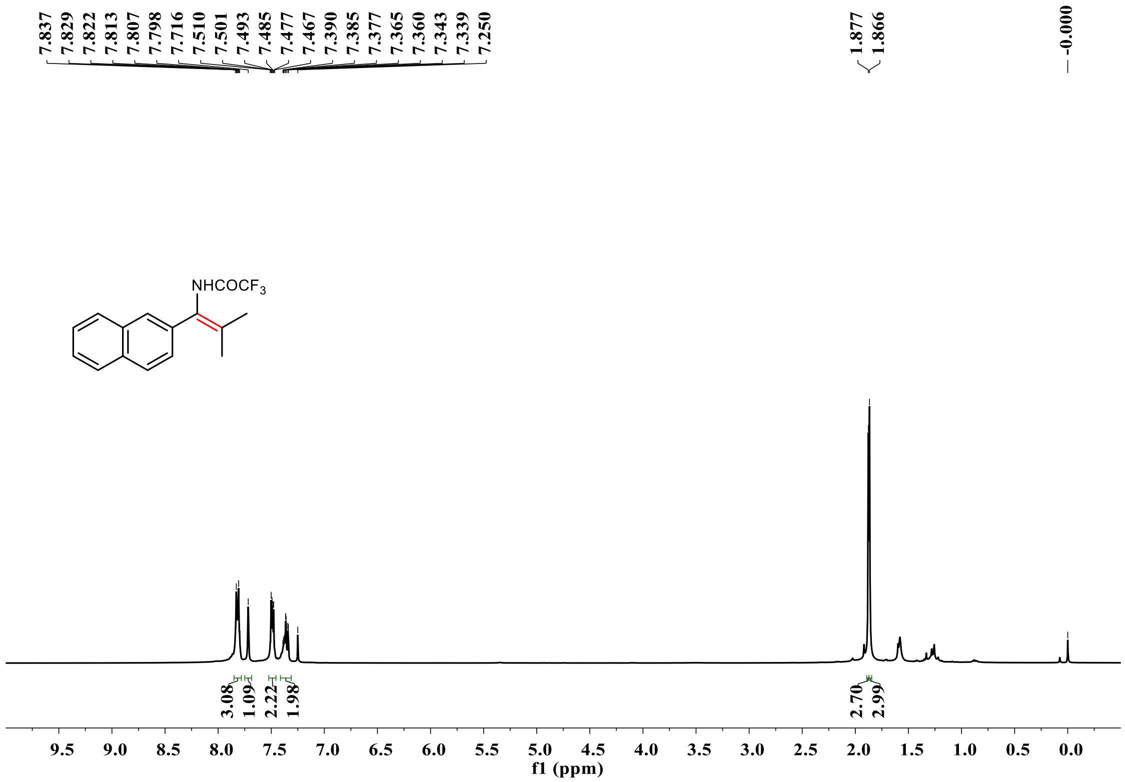

Supplementary Figure 124. ^{1}H NMR spectrum for **EA4**

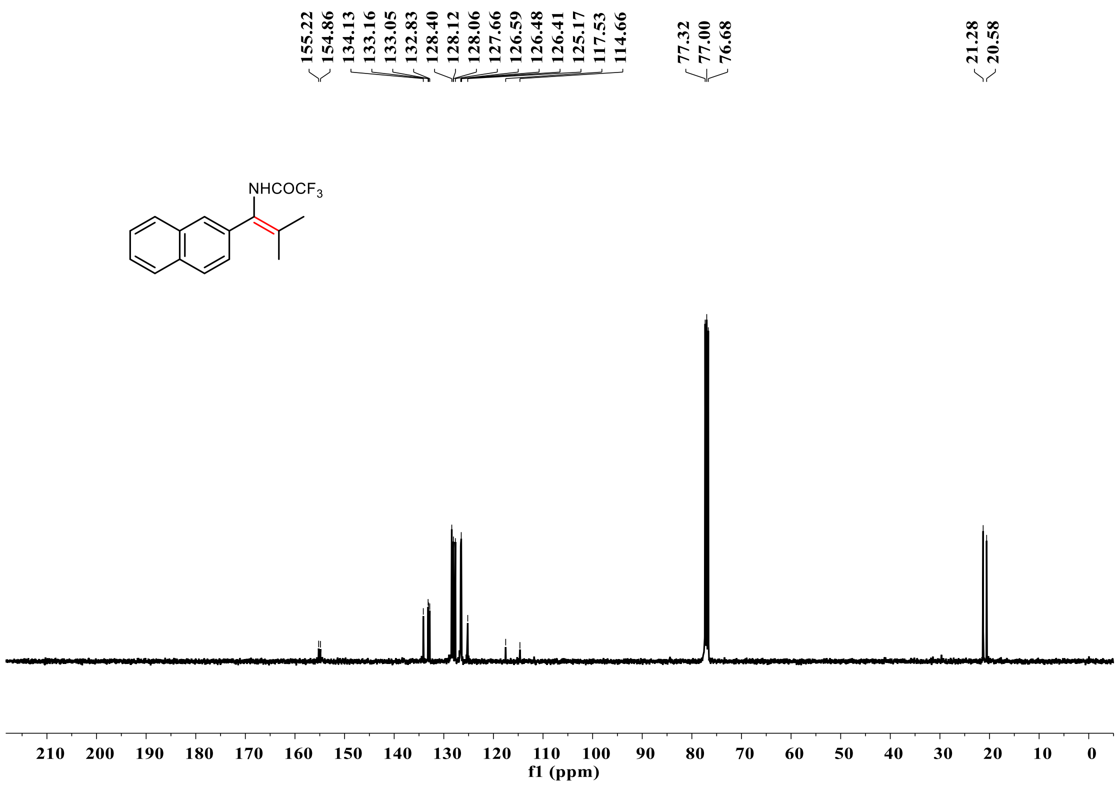

Supplementary Figure 125. ^{13}C NMR spectrum for **EA4**

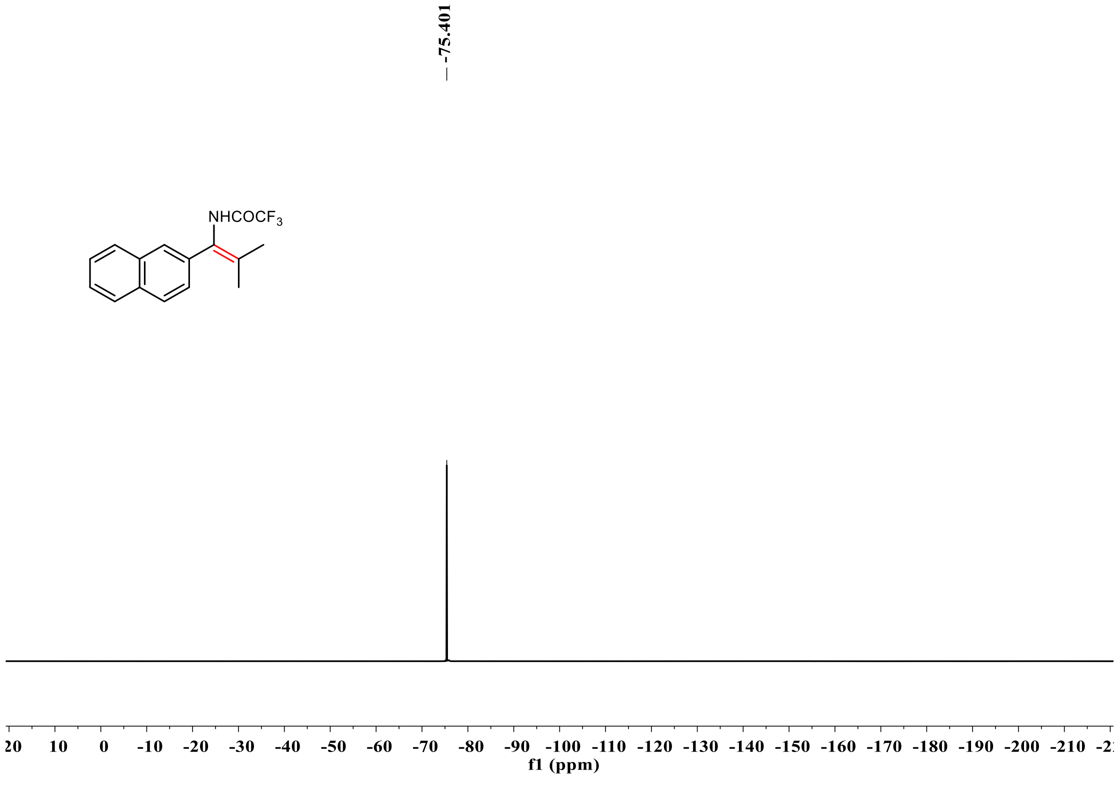

Supplementary Figure 126. ^{19}F NMR spectrum for **EA4**

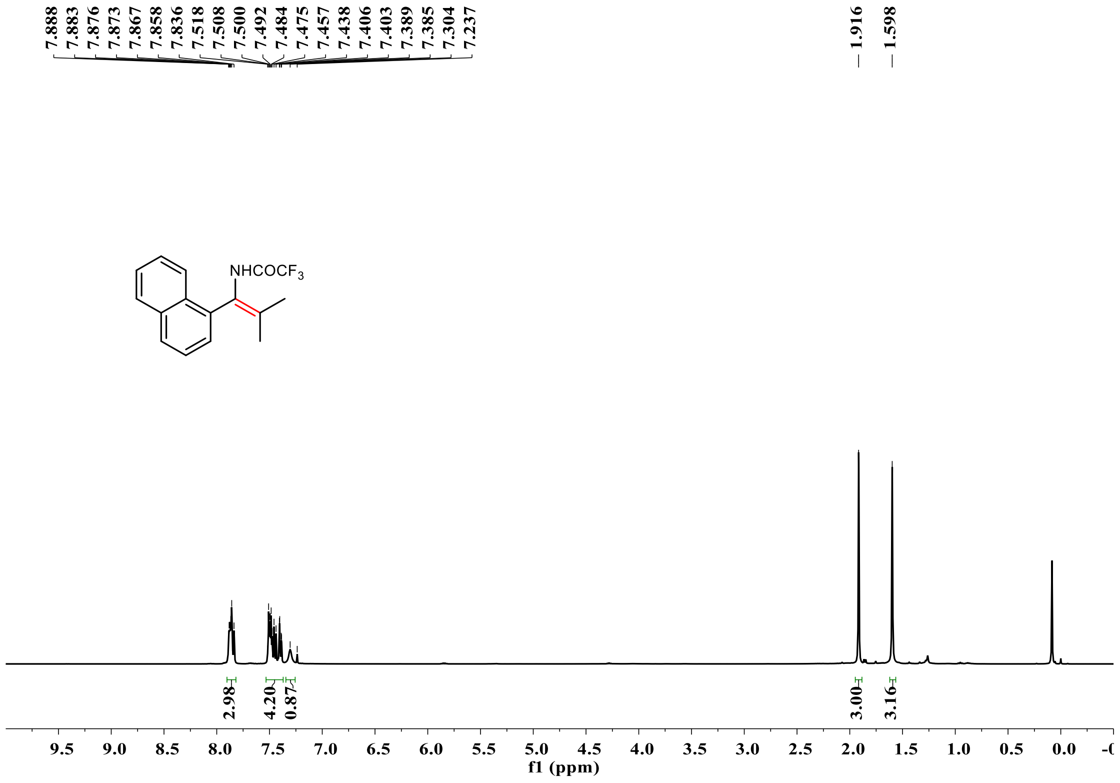

Supplementary Figure 127. ^{1}H NMR spectrum for **EA5**

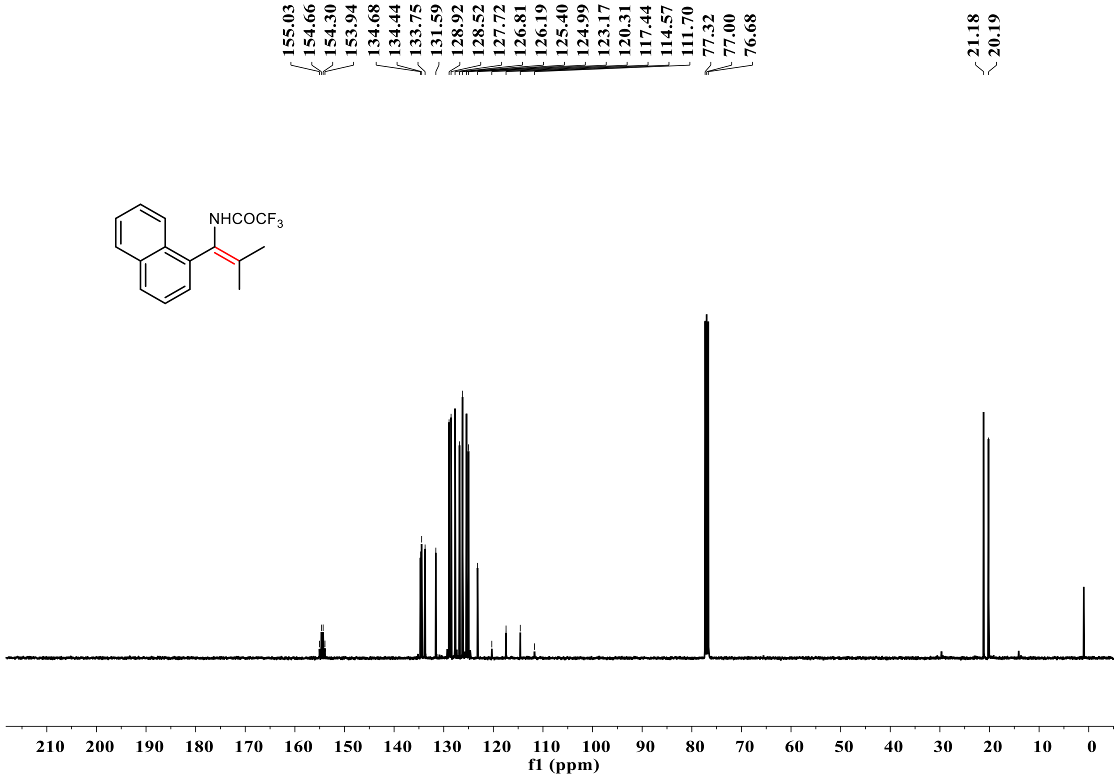

Supplementary Figure 128. ^{13}C NMR spectrum for **EA5**

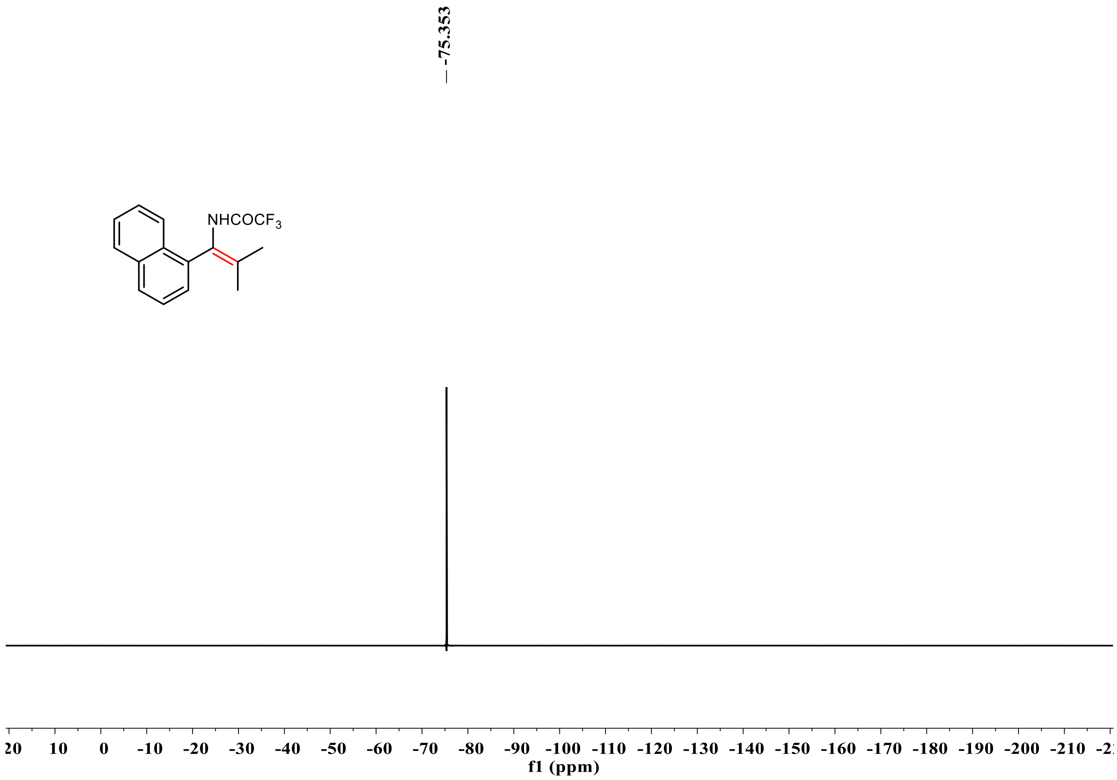

Supplementary Figure 129. ^{19}F NMR spectrum for **EA5**

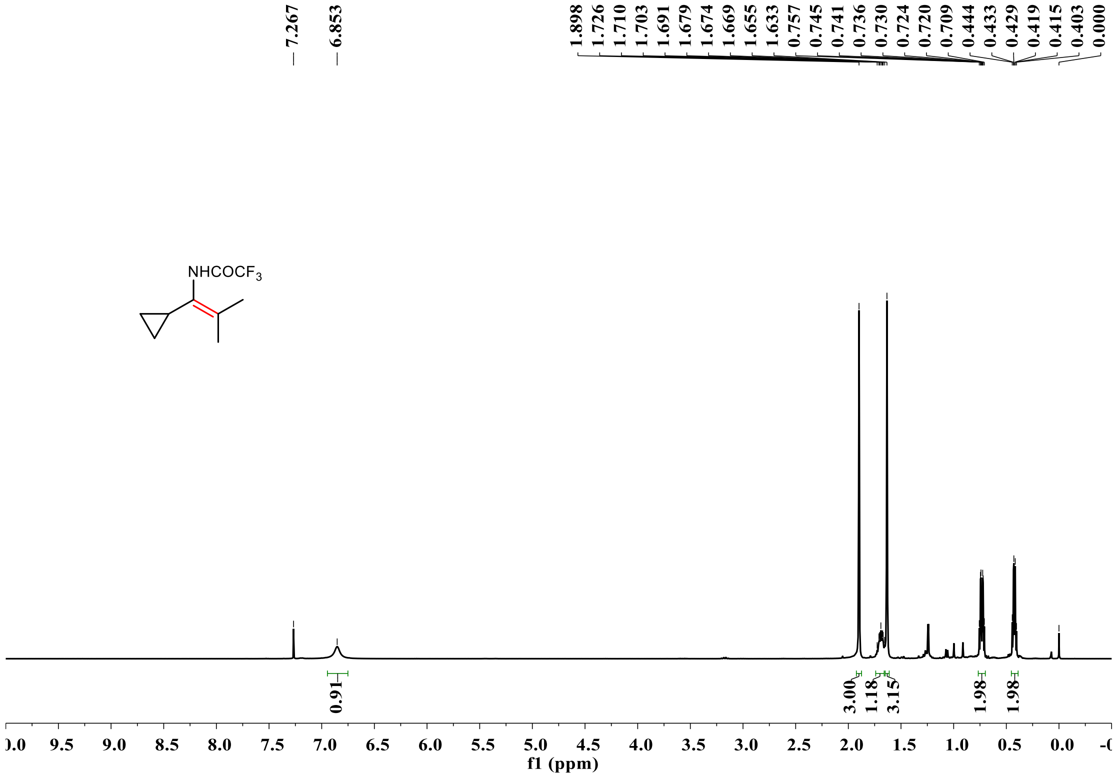

Supplementary Figure 130. ^{1}H NMR spectrum for **EA6**

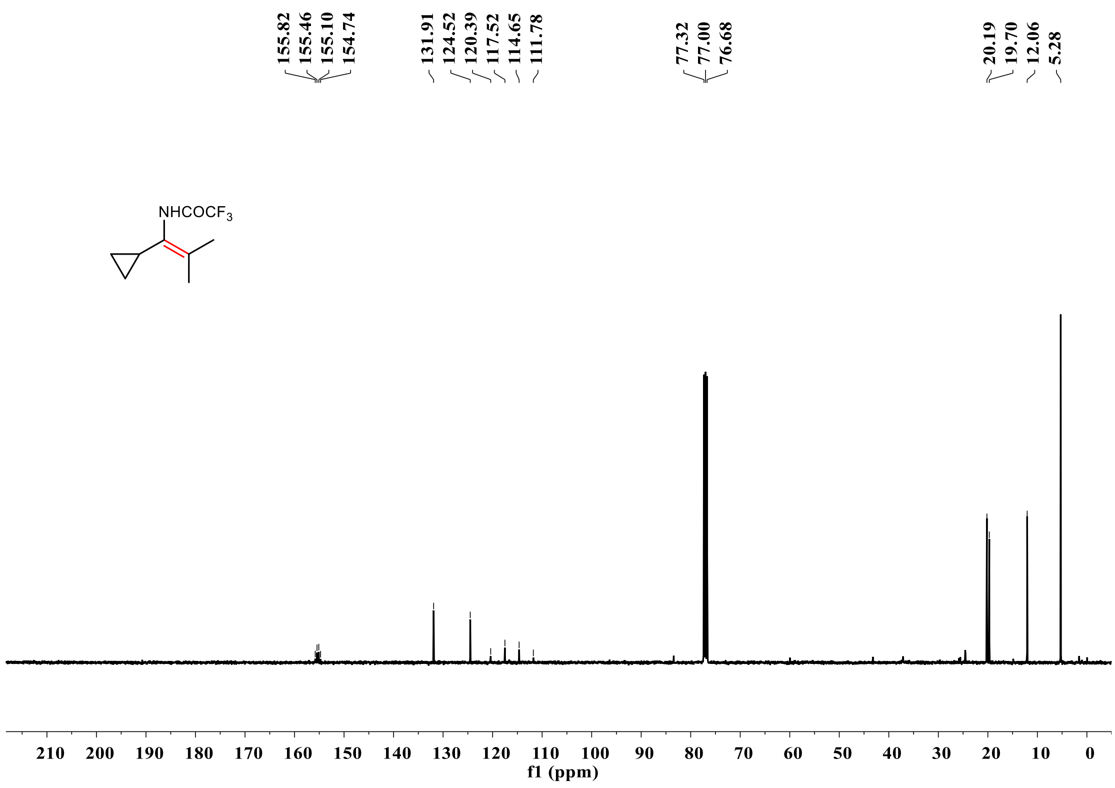

Supplementary Figure 131. ^{13}C NMR spectrum for **EA6**

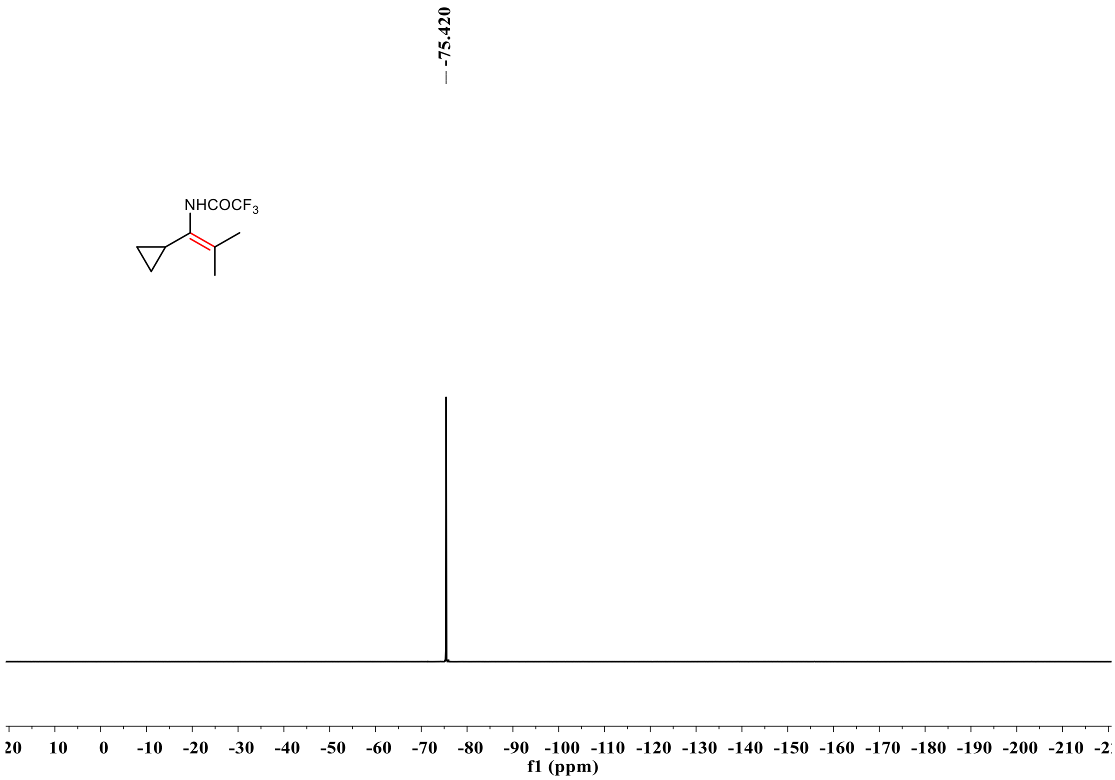

Supplementary Figure 132. ^{19}F NMR spectrum for **EA6**

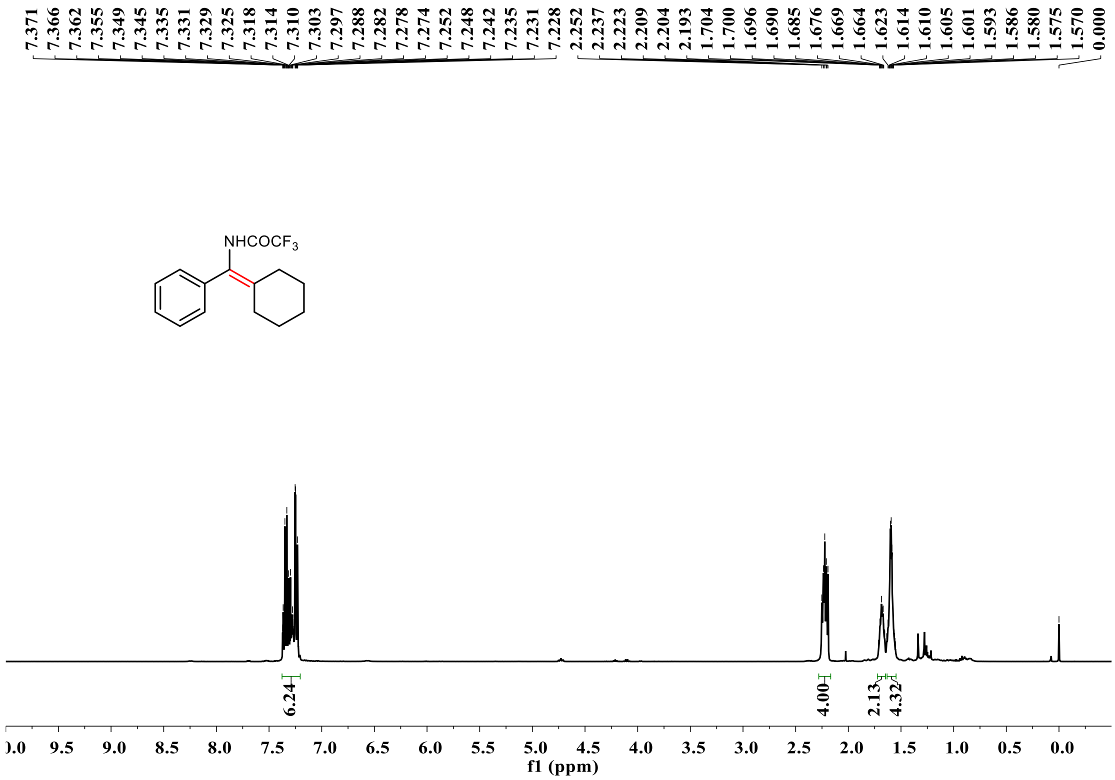

Supplementary Figure 133. ^{1}H NMR spectrum for **EA7**

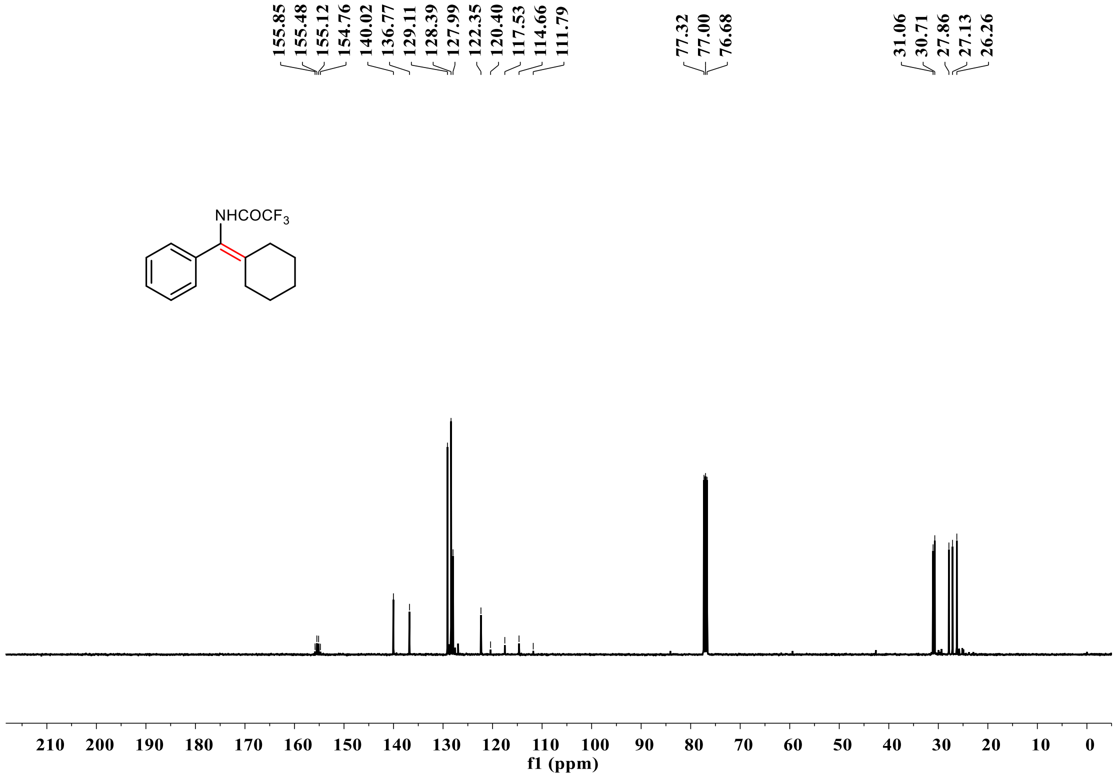

Supplementary Figure 134. ^{13}C NMR spectrum for **EA7**

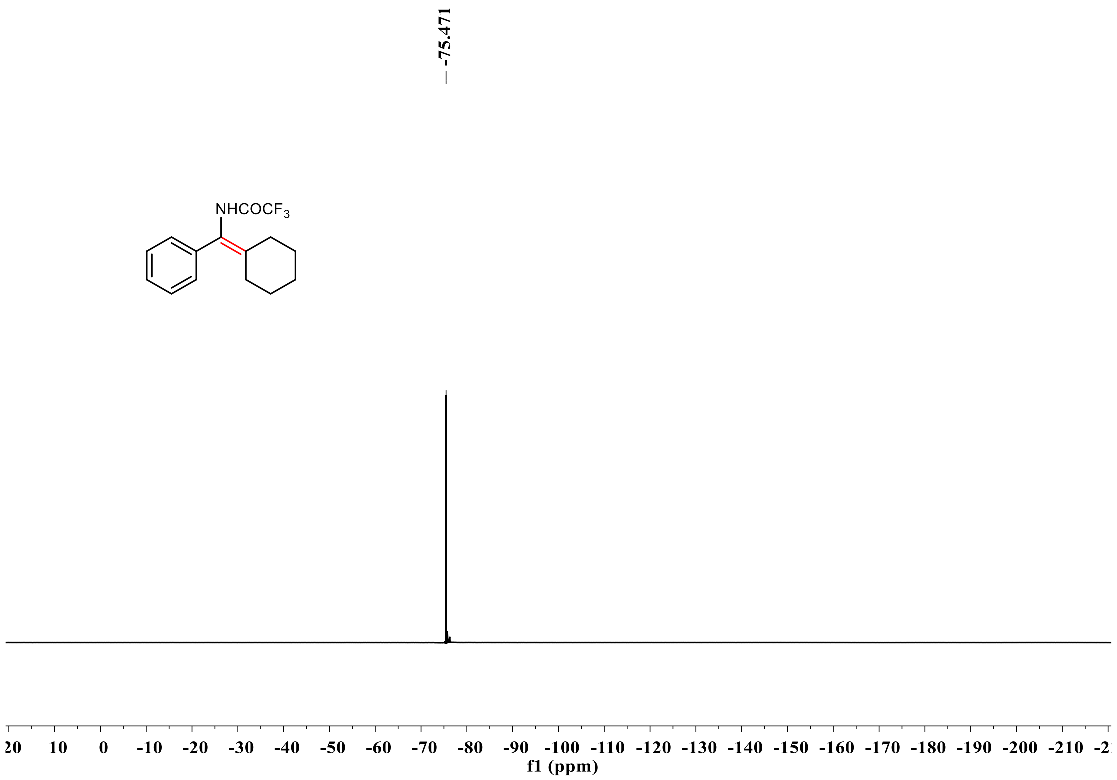

Supplementary Figure 135. ^{19}F NMR spectrum for **EA7**

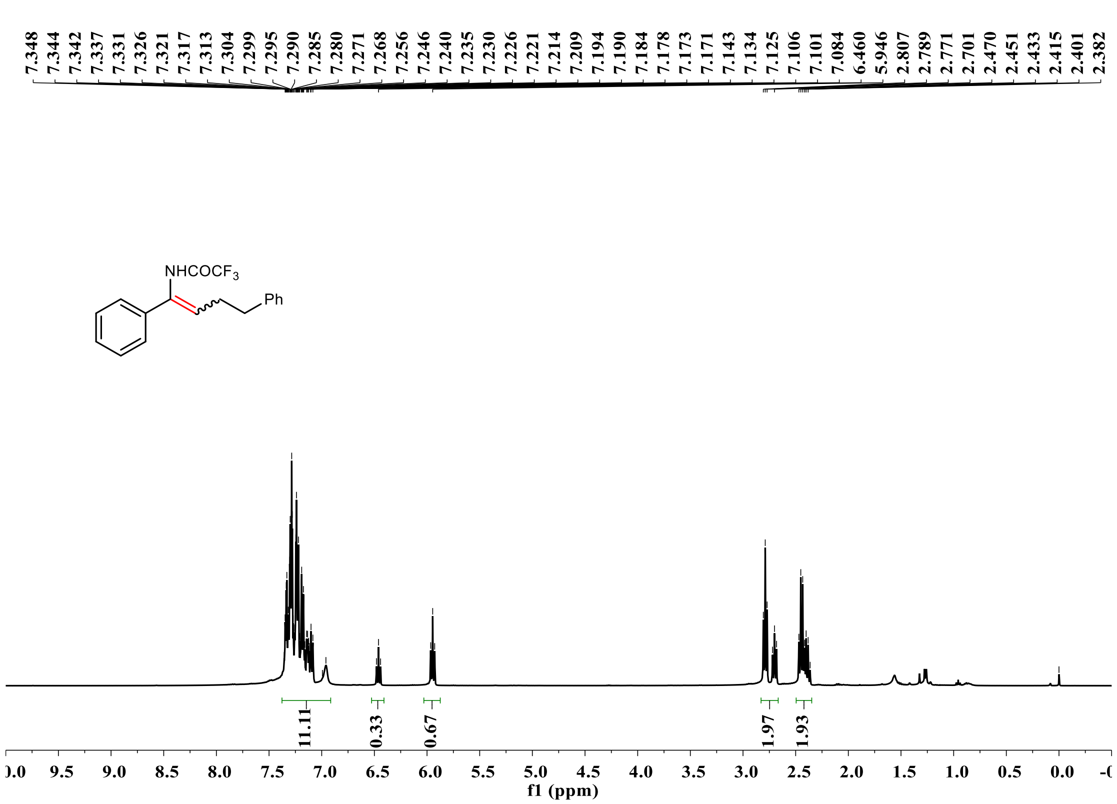

Supplementary Figure 136. ^{1}H NMR spectrum for **EA8**

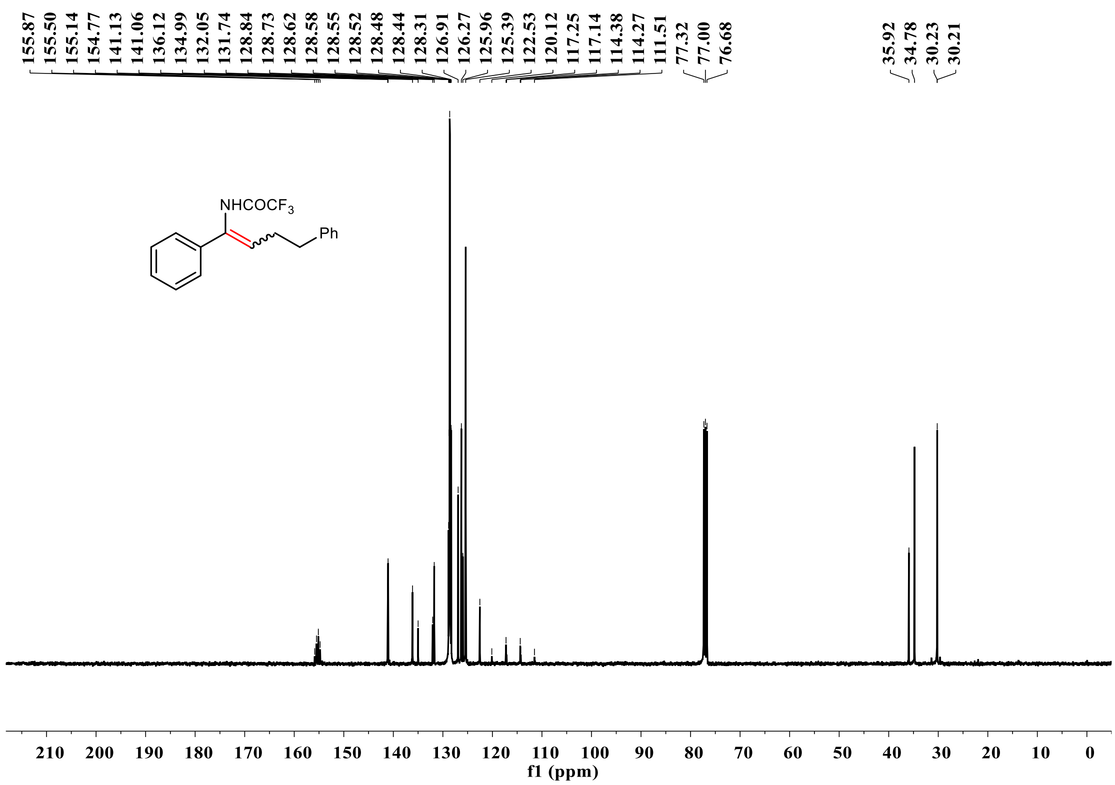

Supplementary Figure 137. ^{13}C NMR spectrum for **EA8**

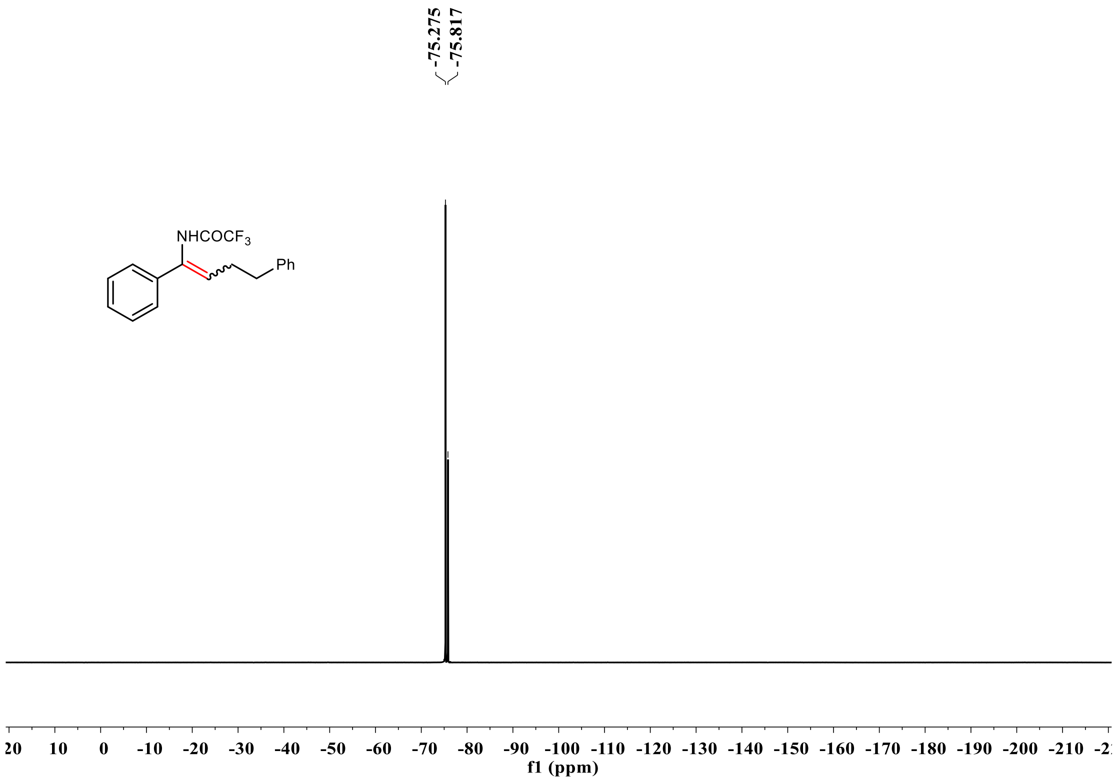

Supplementary Figure 138. ^{19}F NMR spectrum for **EA8**

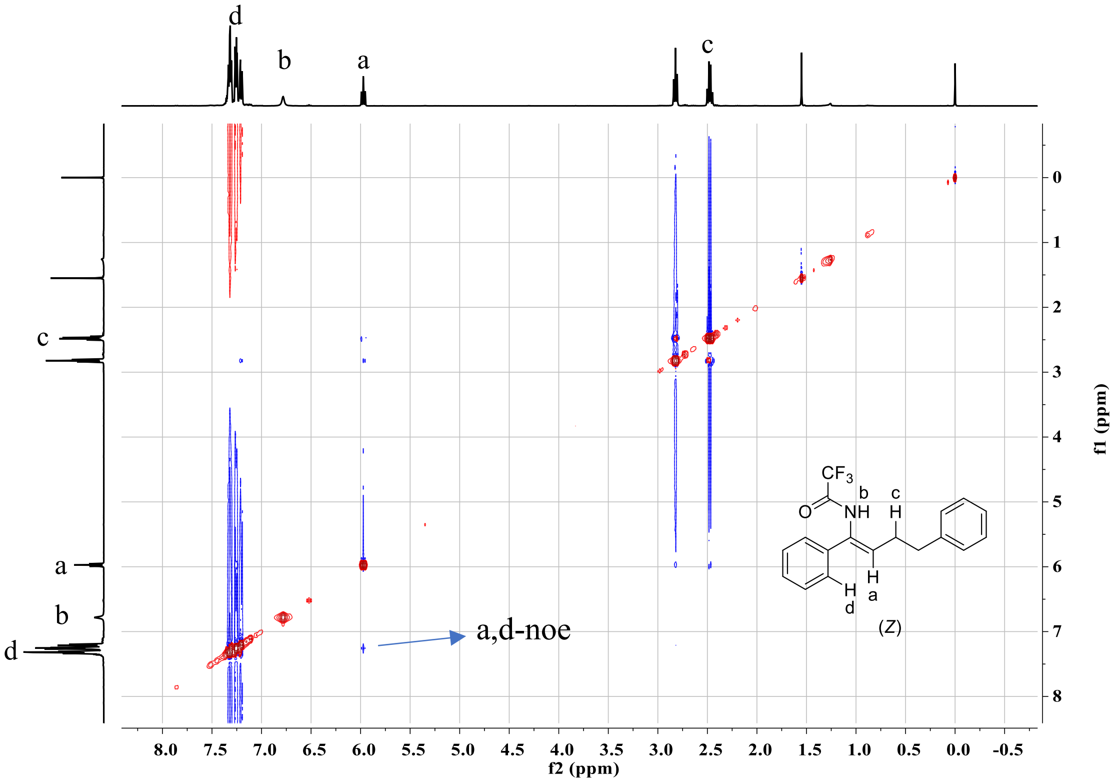

Supplementary Figure 139. NOE spectrum for **EA8**

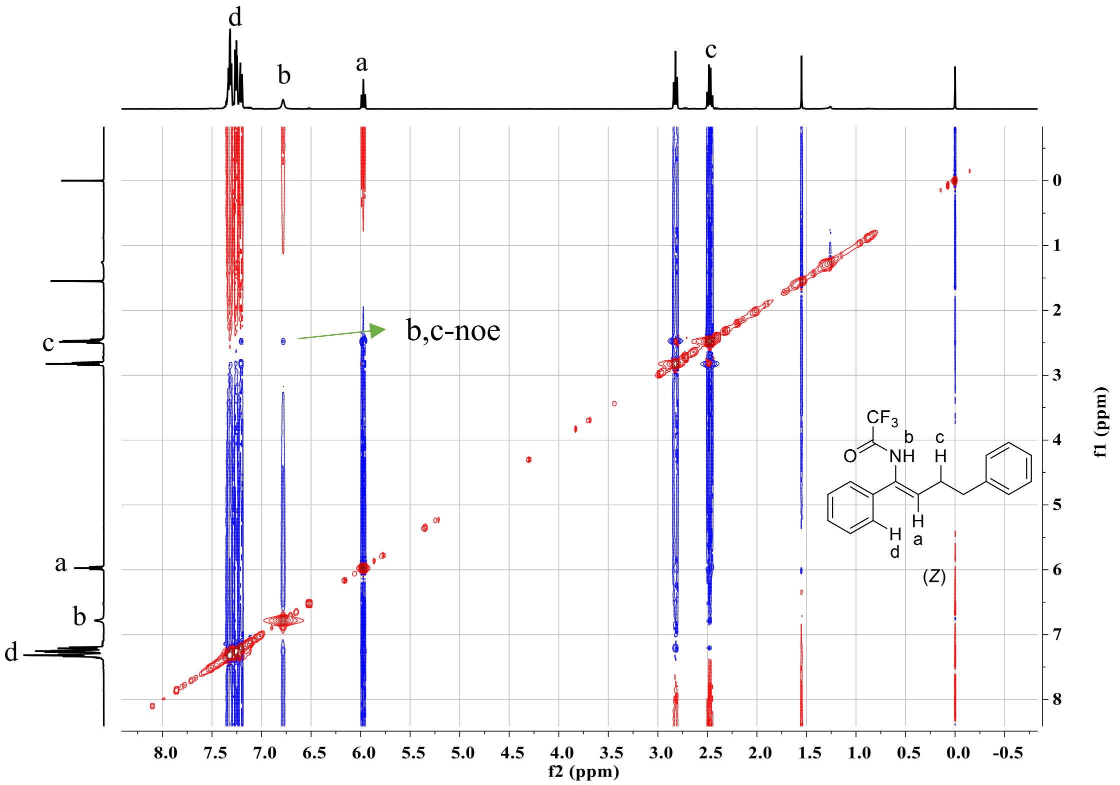

Supplementary Figure 140. NOE spectrum for **EA8**

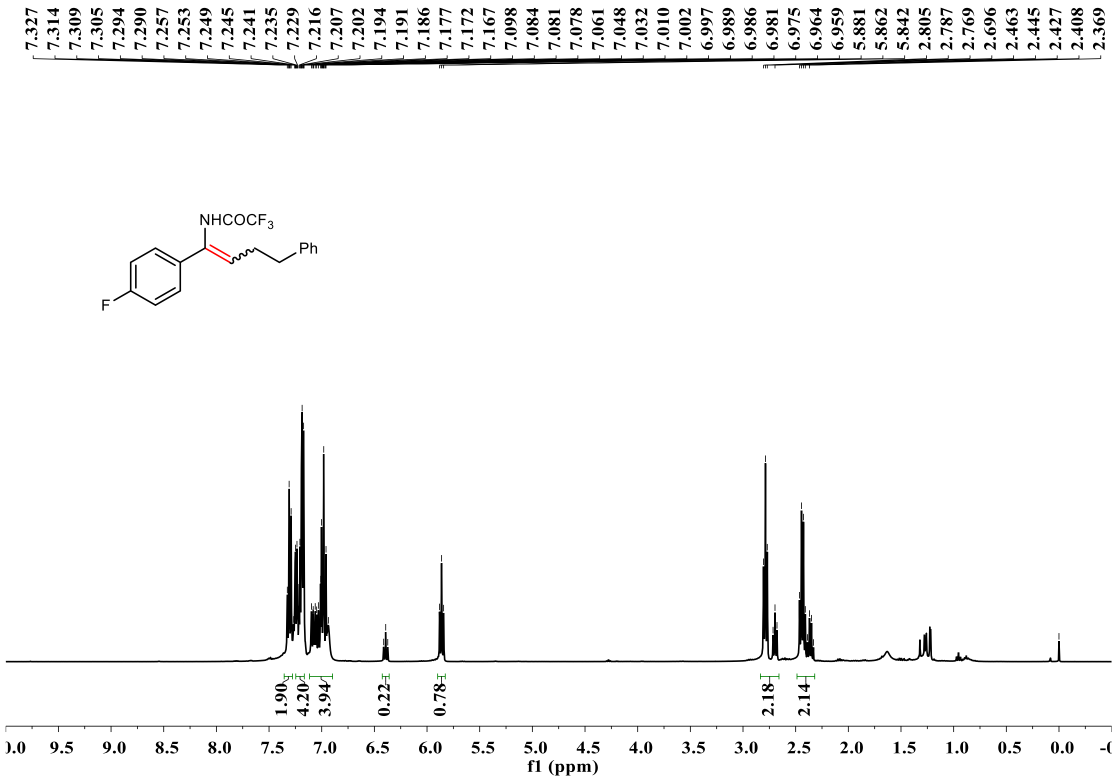

Supplementary Figure 141. ^{1}H NMR spectrum for **EA9**

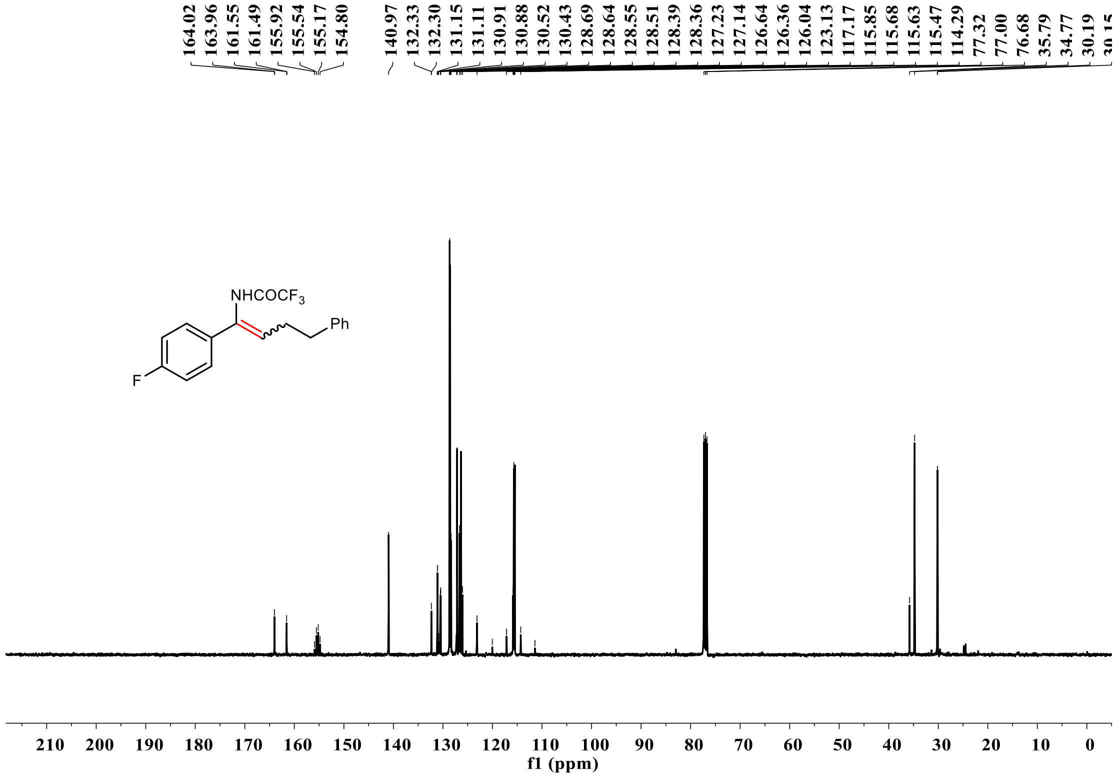

Supplementary Figure 142. ^{13}C NMR spectrum for **EA9**

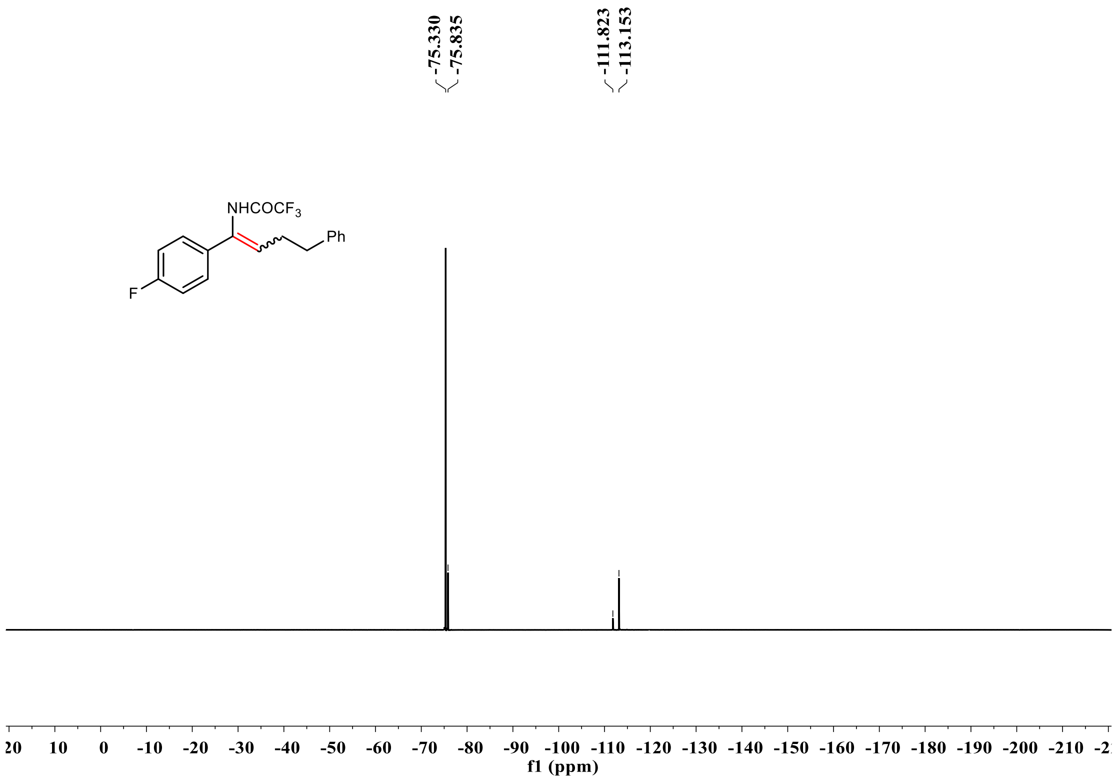

Supplementary Figure 143. ^{19}F NMR spectrum for **EA9**

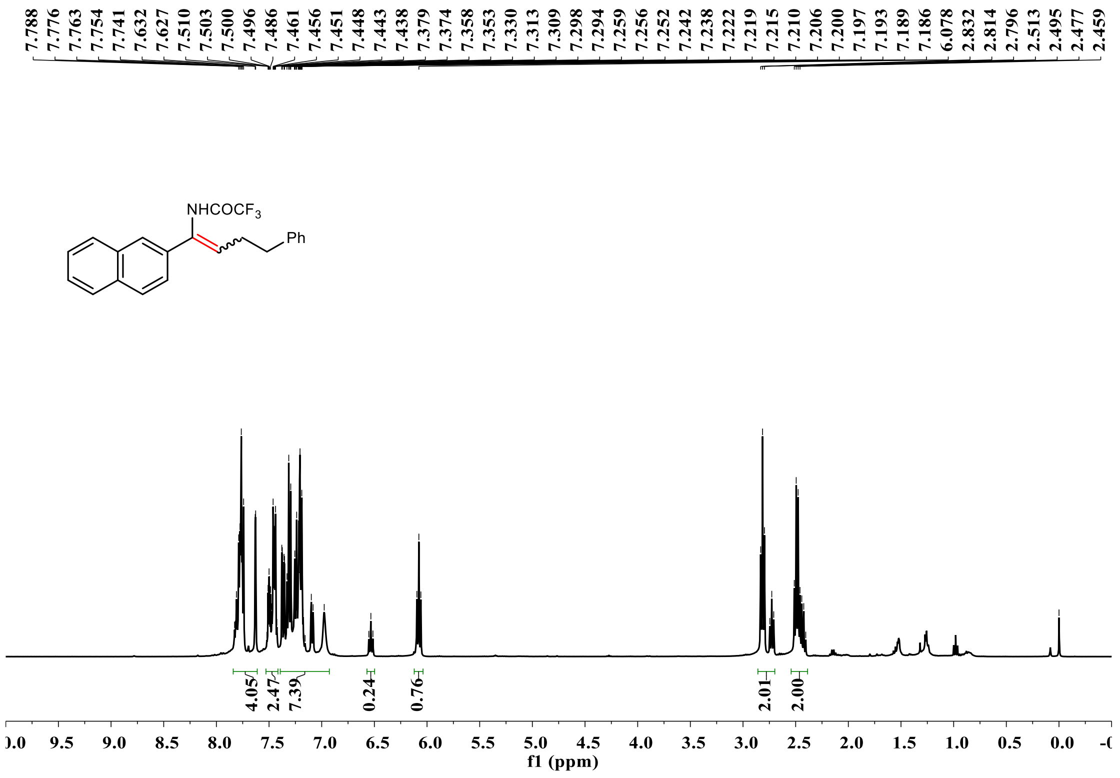

Supplementary Figure 144. ^{1}H NMR spectrum for **EA10**

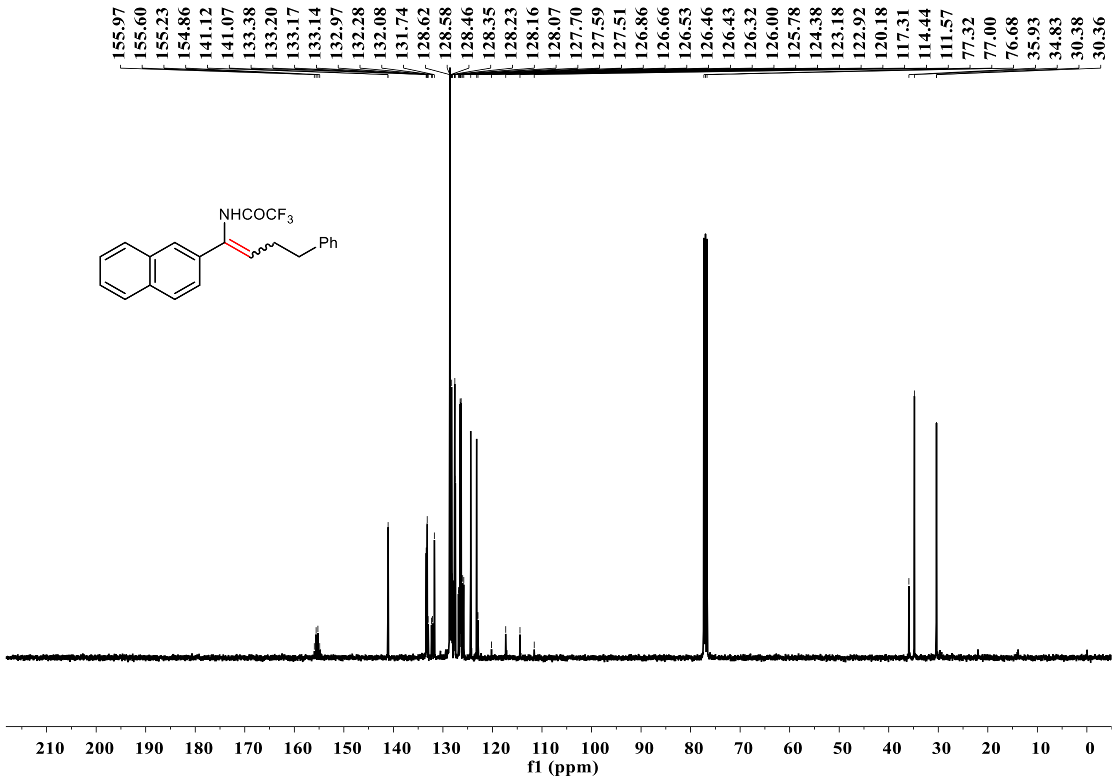

Supplementary Figure 145. ^{13}C NMR spectrum for **EA10**

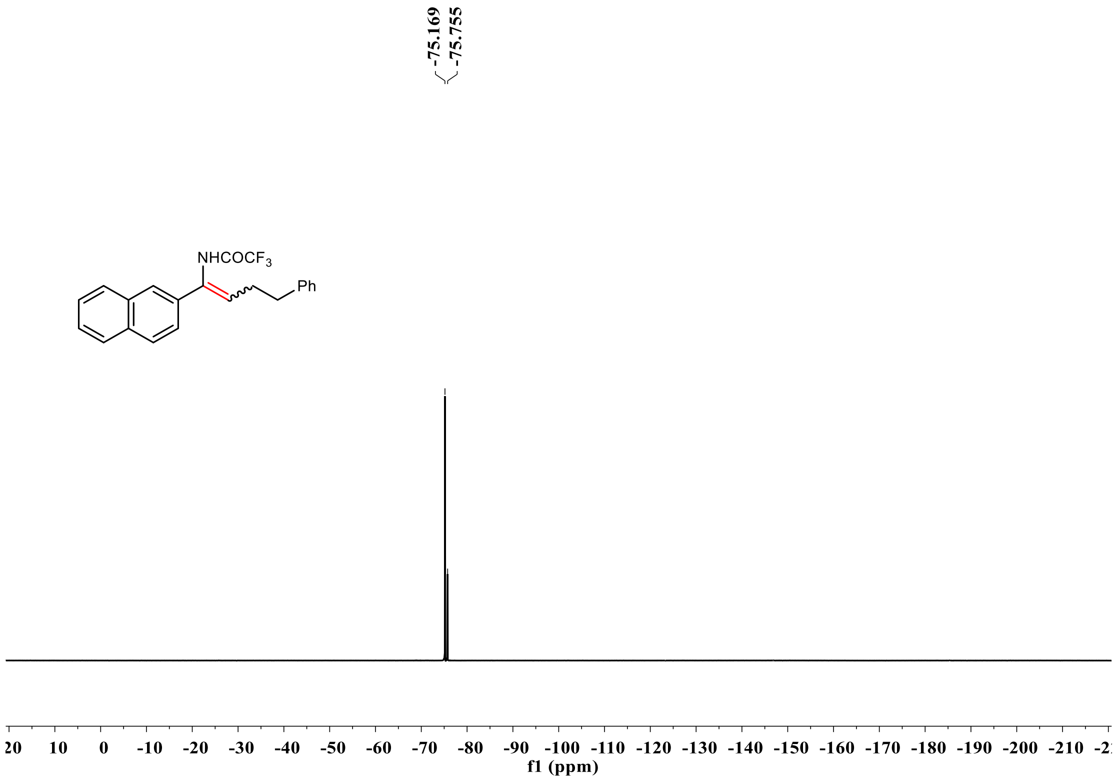

Supplementary Figure 146. ^{19}F NMR spectrum for **EA10**

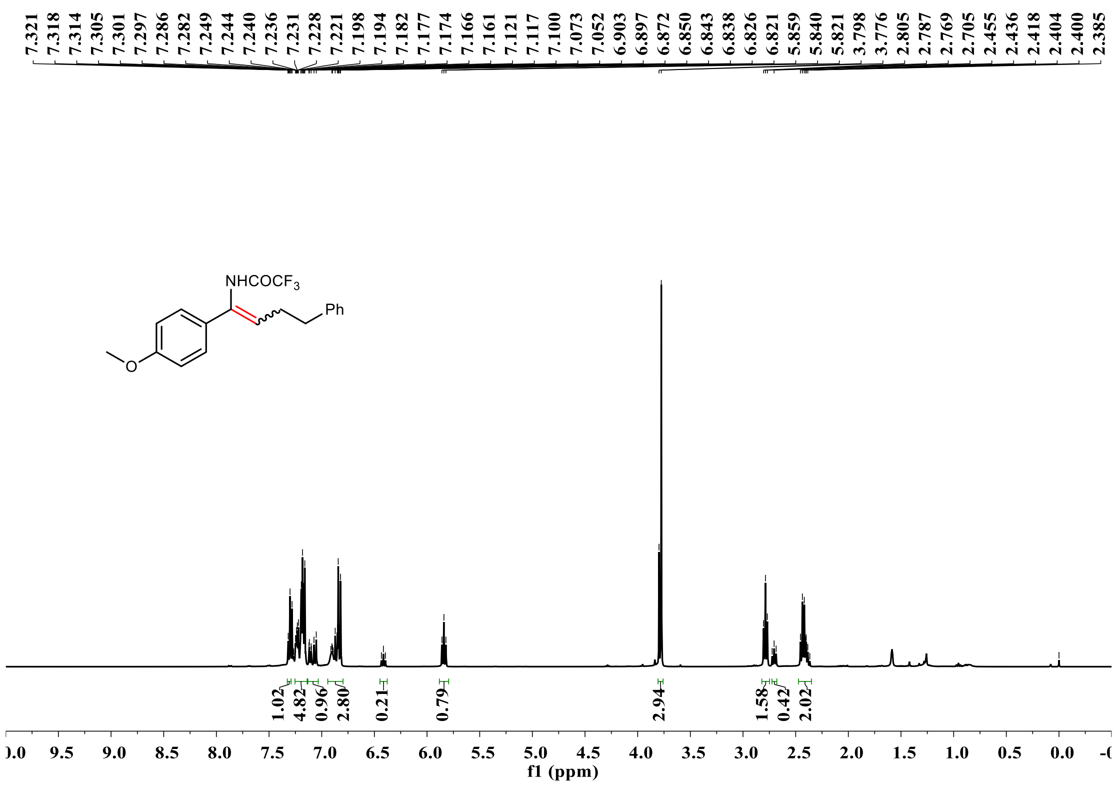

Supplementary Figure 147. ^{1}H NMR spectrum for **EA11**

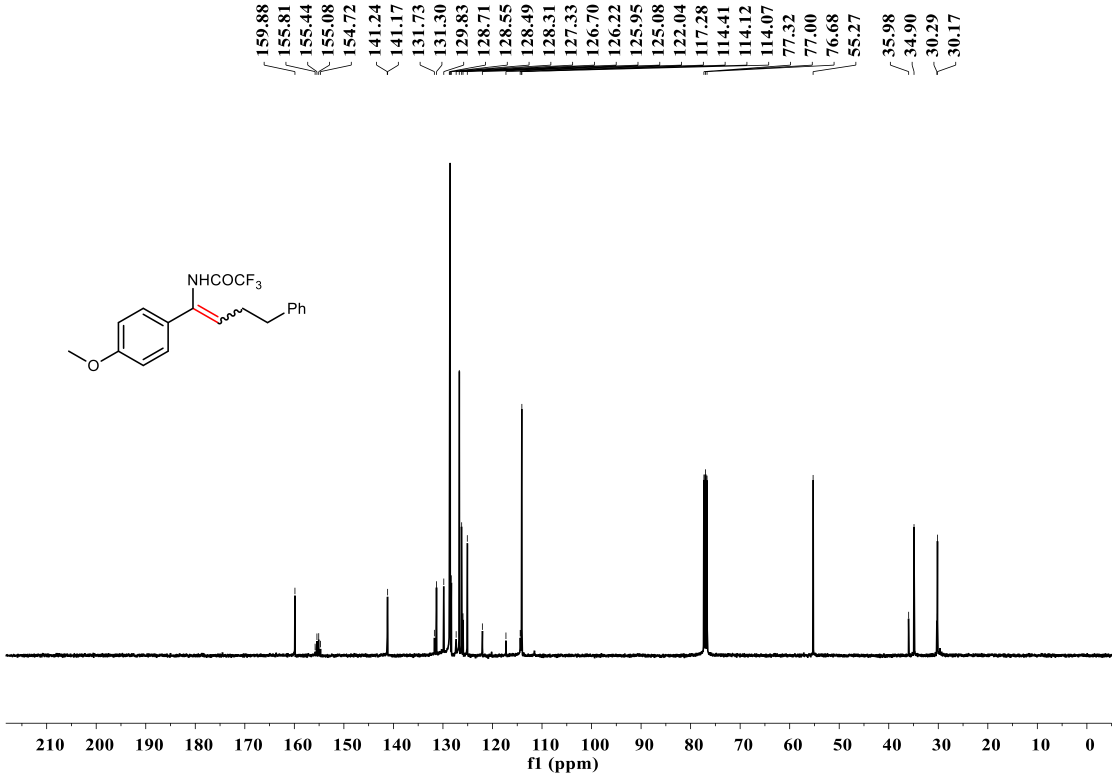

Supplementary Figure 148. ^{13}C NMR spectrum for **EA11**

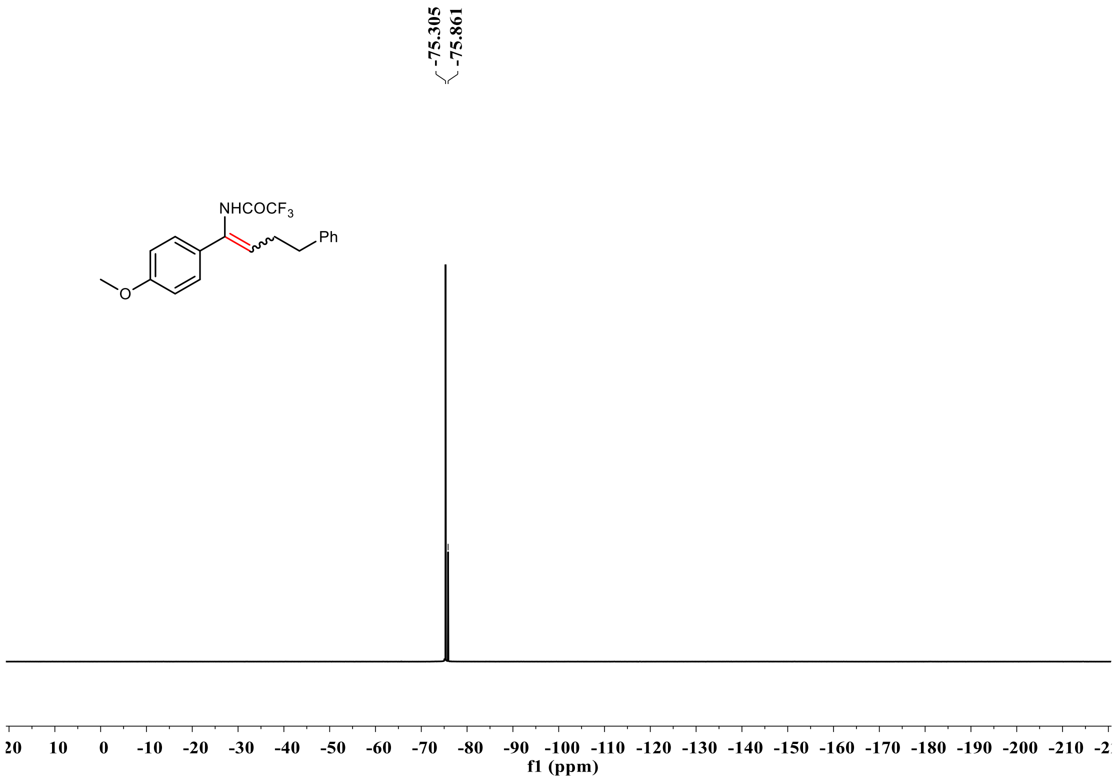

Supplementary Figure 149. ^{19}F NMR spectrum for **EA11**

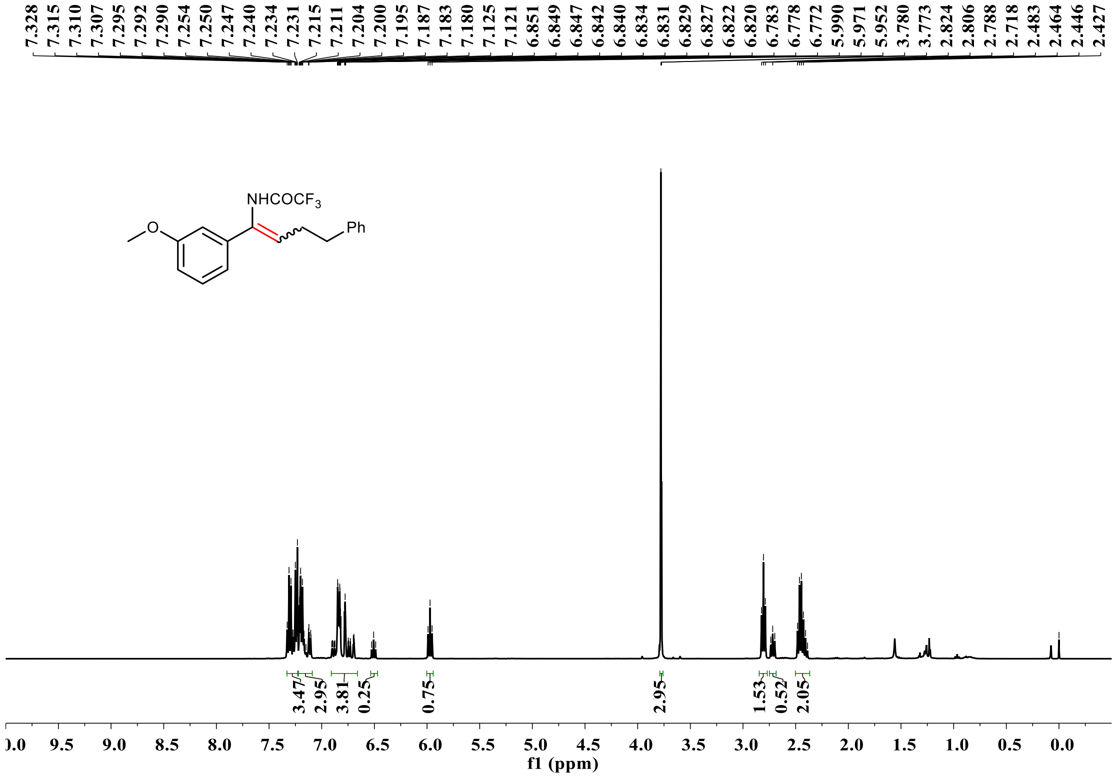

Supplementary Figure 150. ^{1}H NMR spectrum for **EA12**

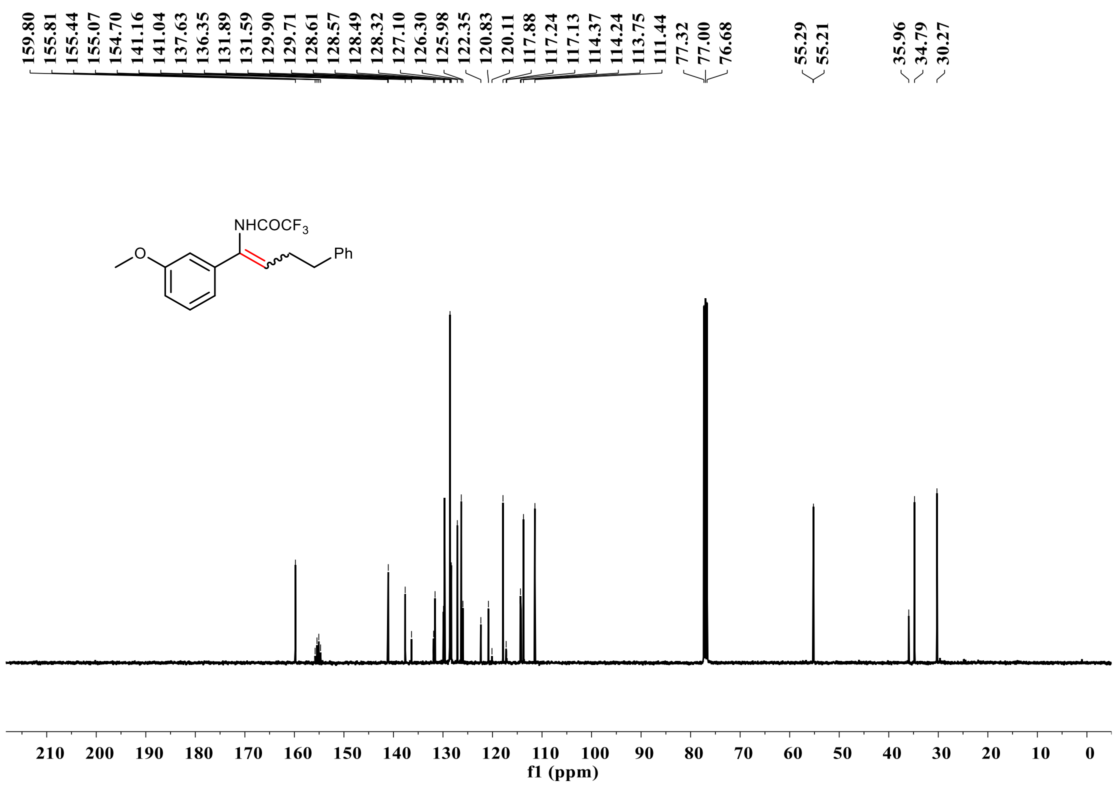

Supplementary Figure 151. ^{13}C NMR spectrum for **EA12**

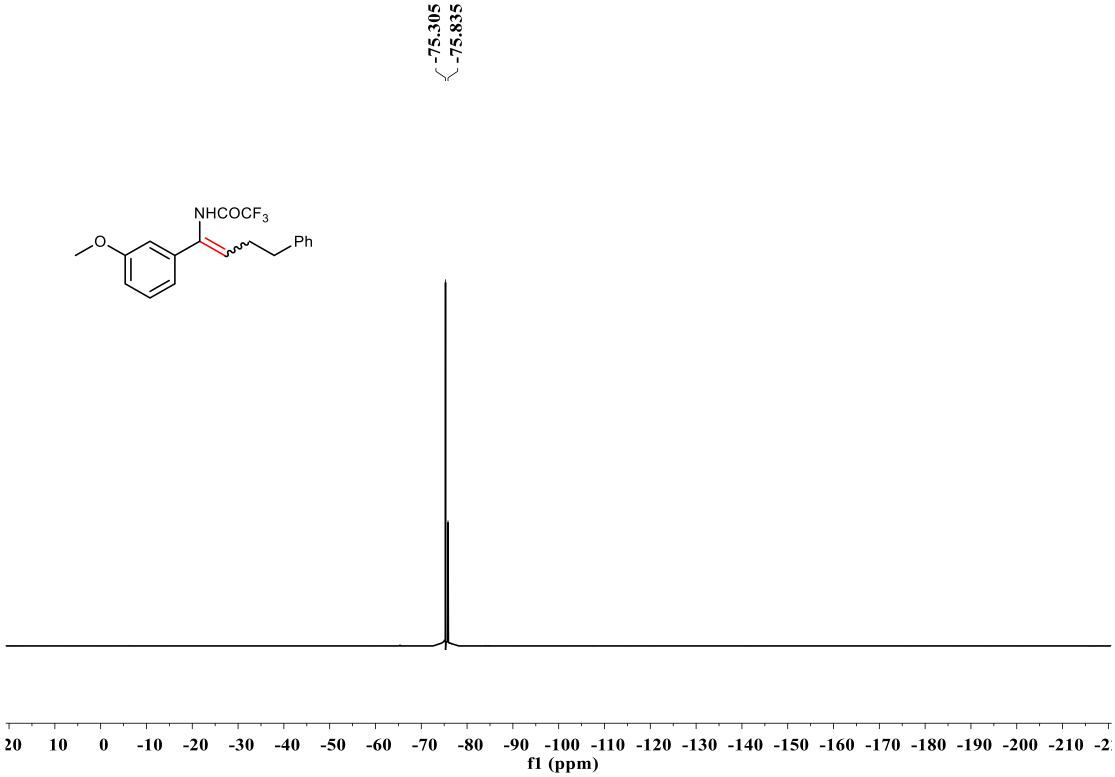

Supplementary Figure 152. ^{19}F NMR spectrum for **EA12**

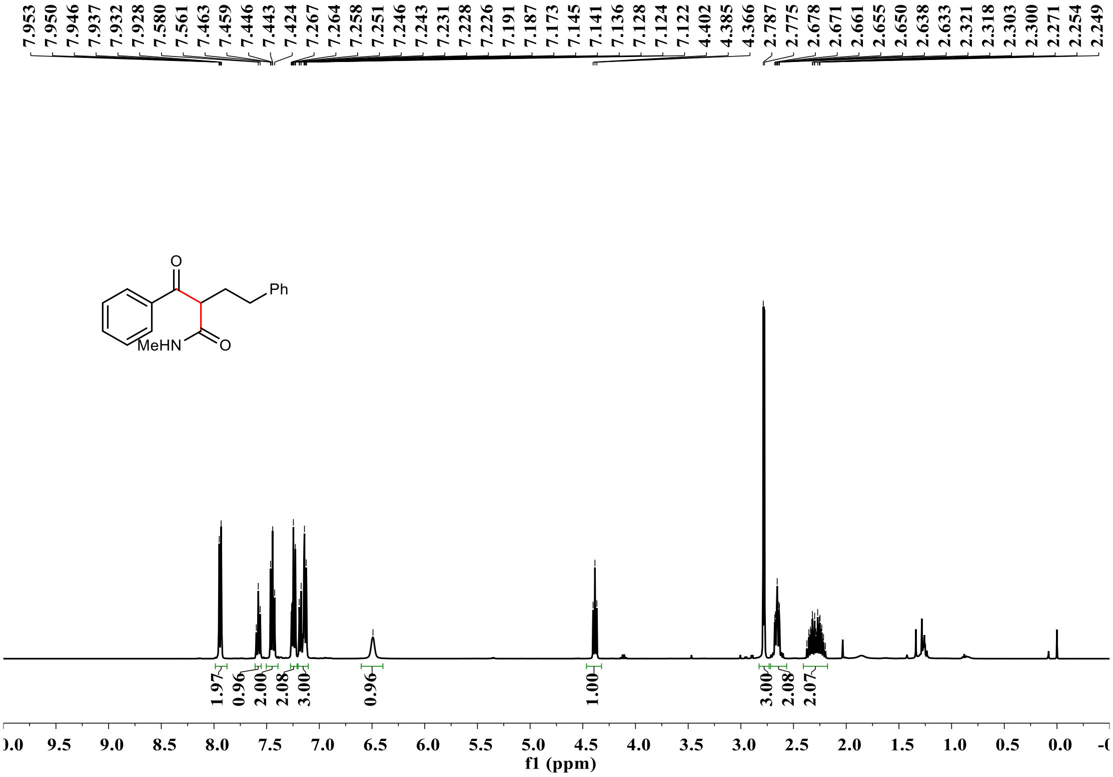

Supplementary Figure 153. ^{1}H NMR spectrum for **KA1**

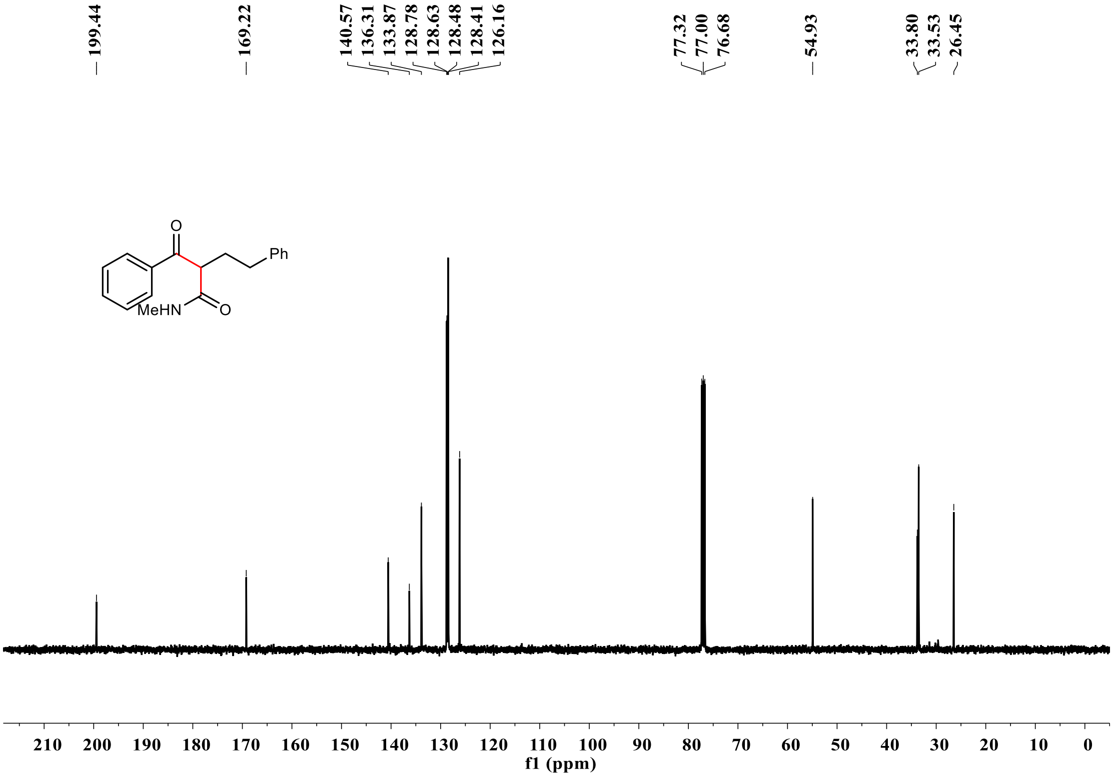

Supplementary Figure 154. ^{13}C NMR spectrum for **KA1**

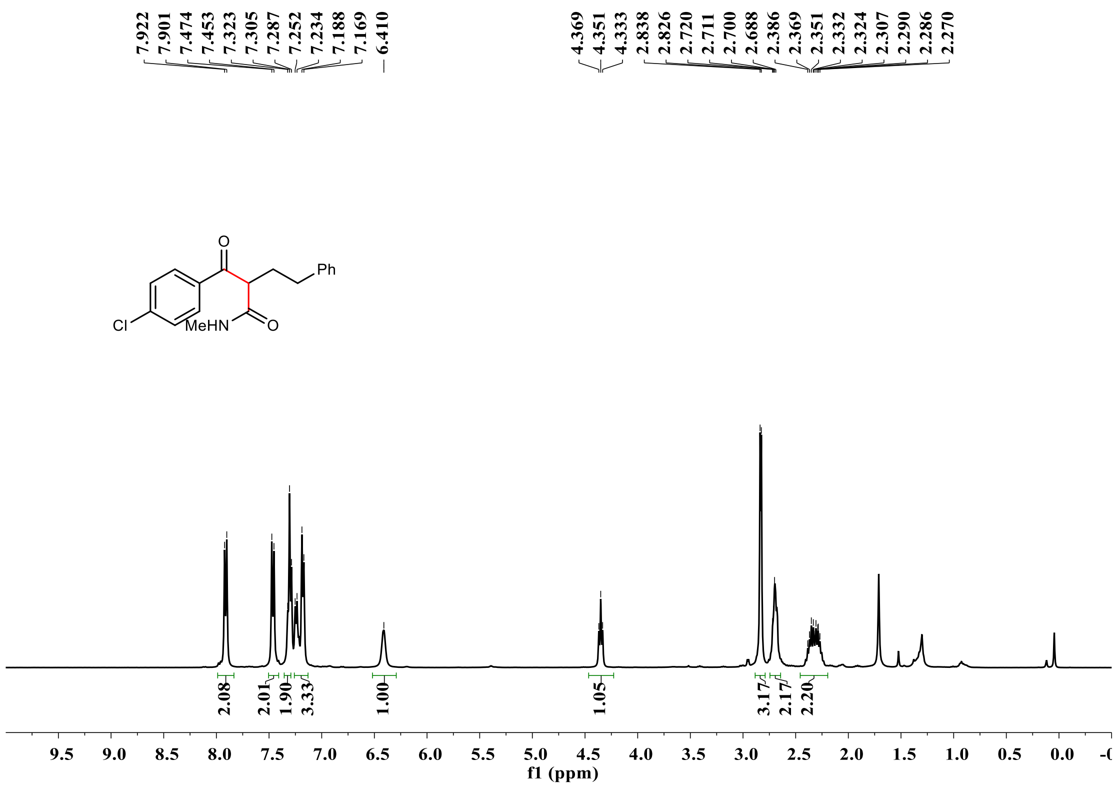

Supplementary Figure 155. ^{1}H NMR spectrum for **KA2**

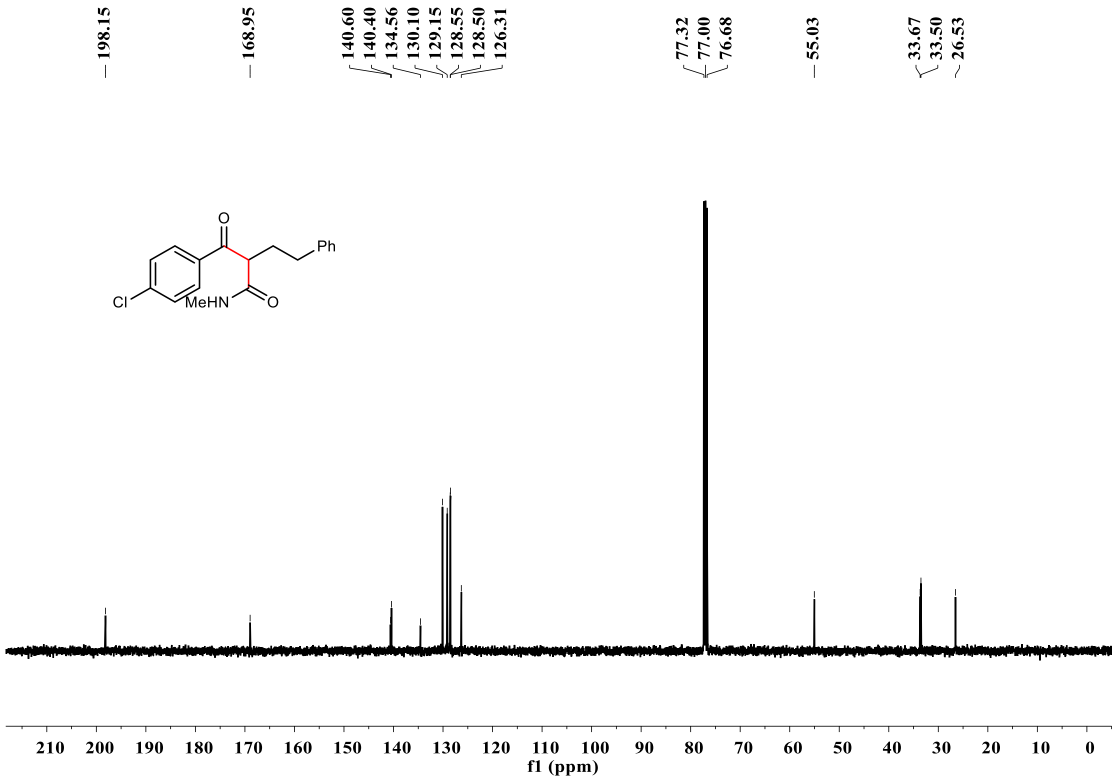

Supplementary Figure 156. ^{13}C NMR spectrum for **KA2**

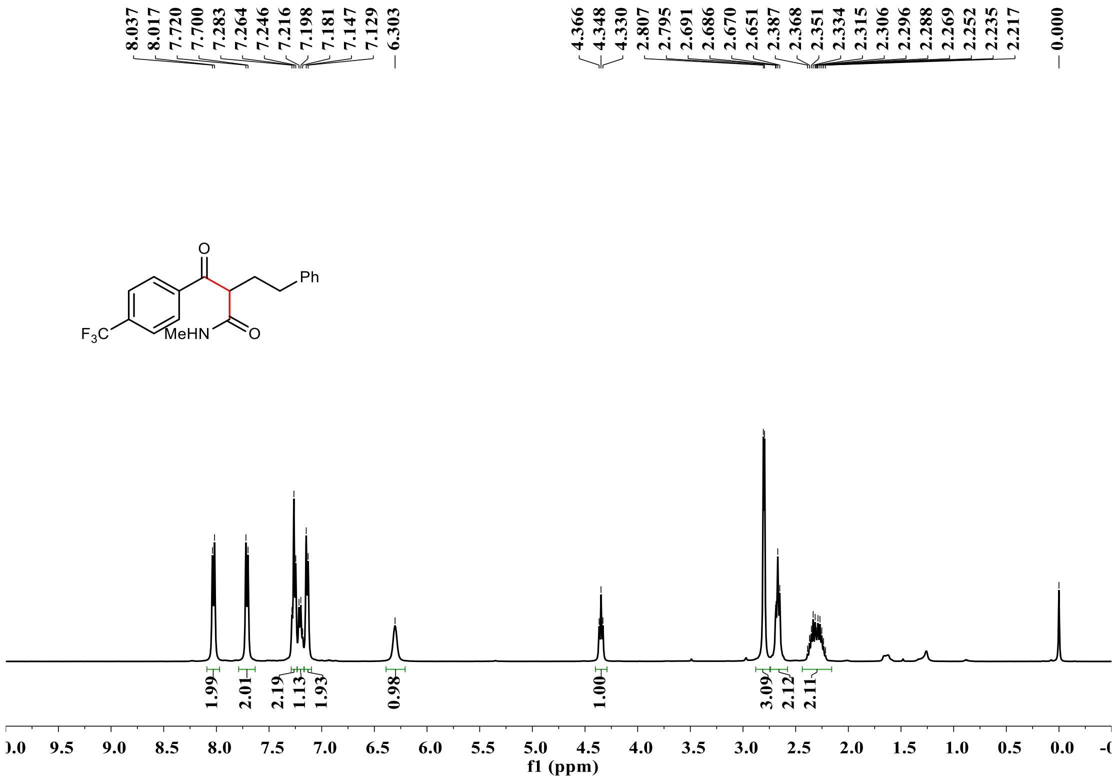

Supplementary Figure 157. ^{1}H NMR spectrum for **KA3**

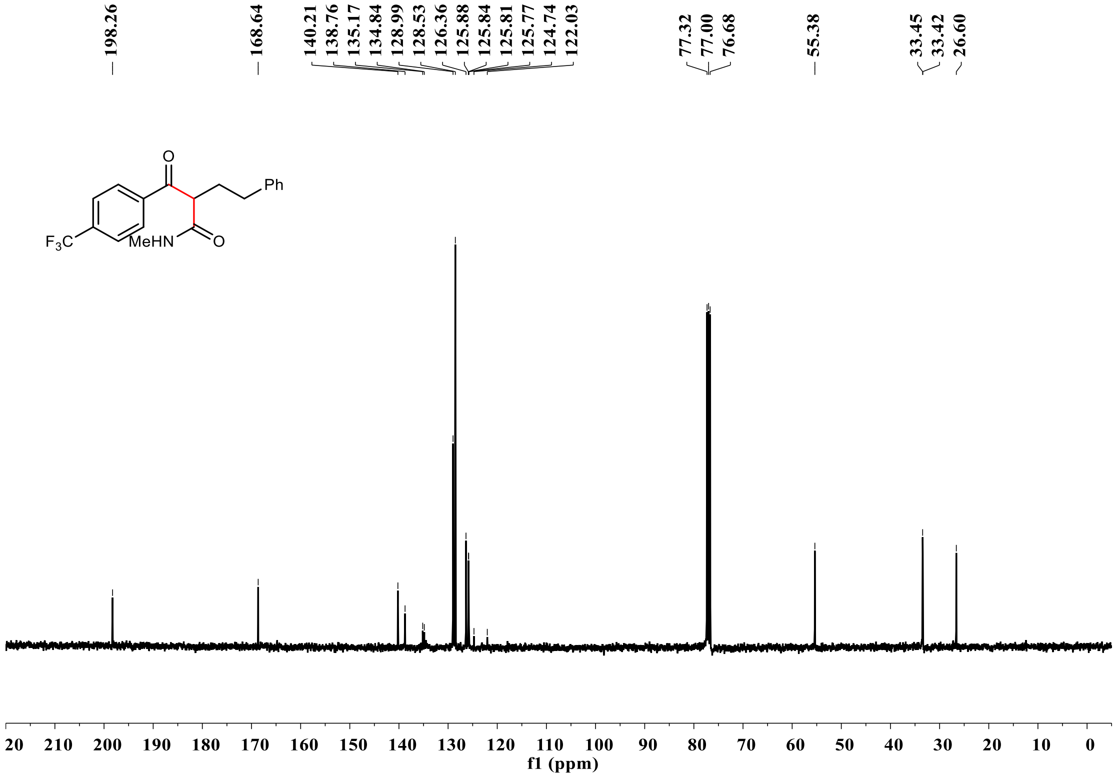

Supplementary Figure 158. ^{13}C NMR spectrum for **KA3**

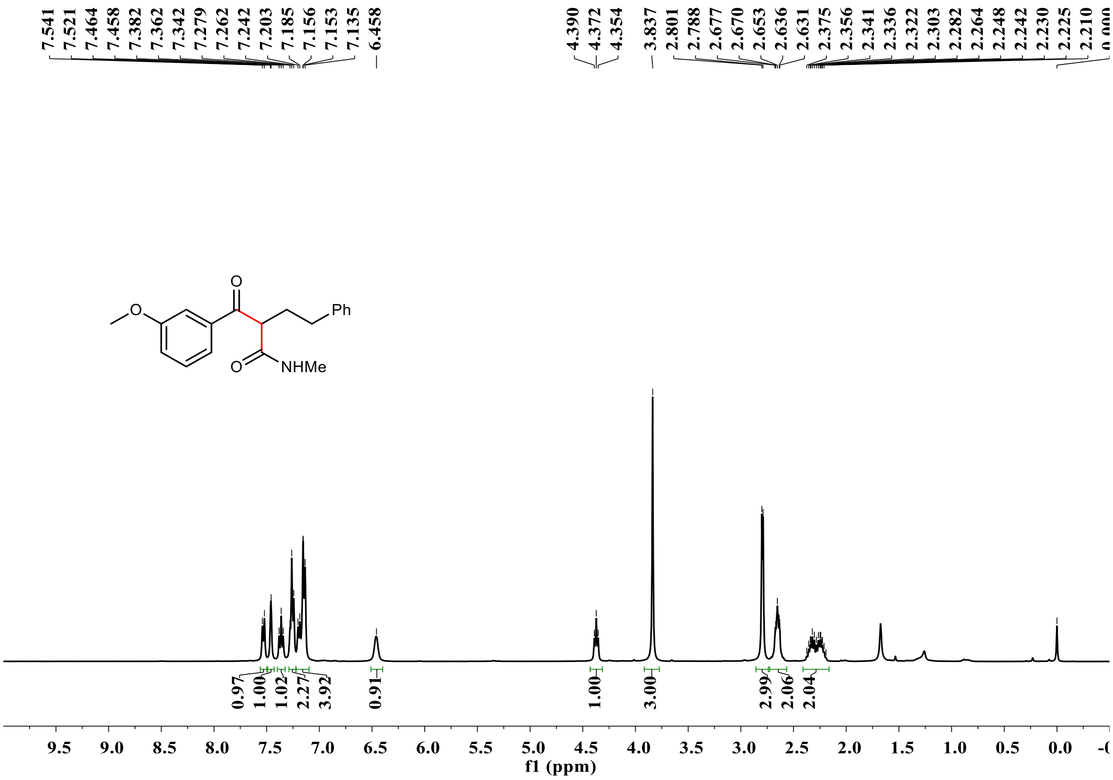

Supplementary Figure 159. ^{1}H NMR spectrum for **KA4**

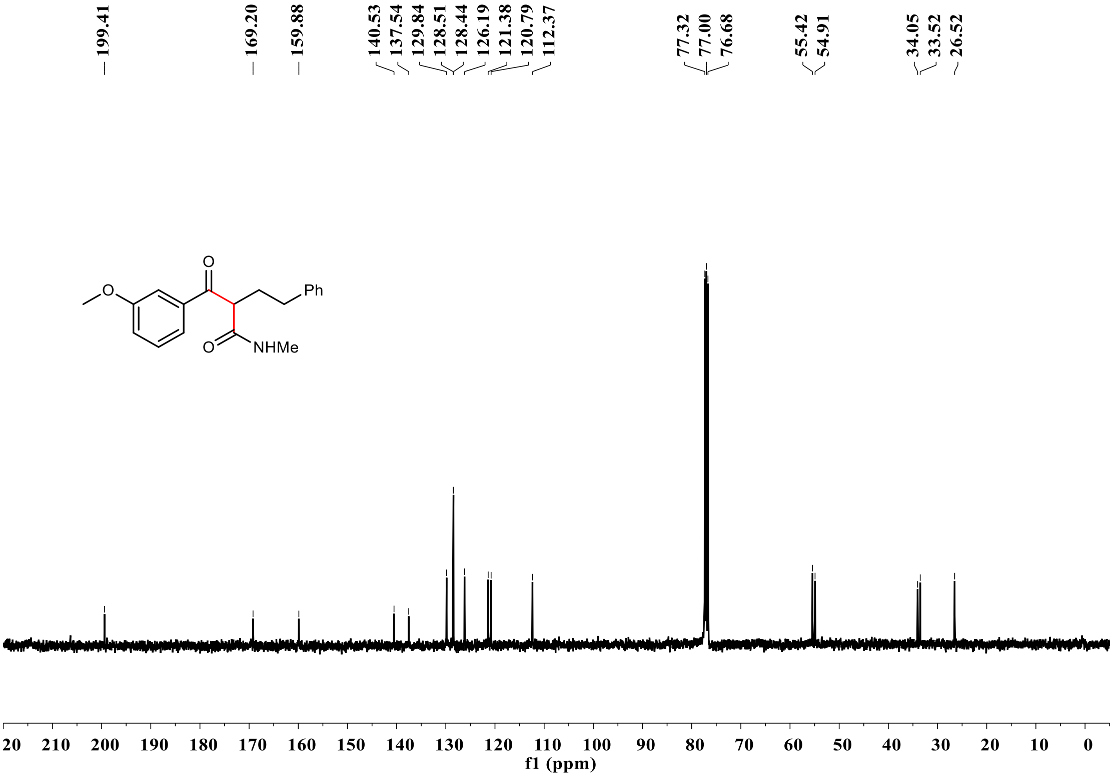

Supplementary Figure 160. ^{13}C NMR spectrum for **KA4**

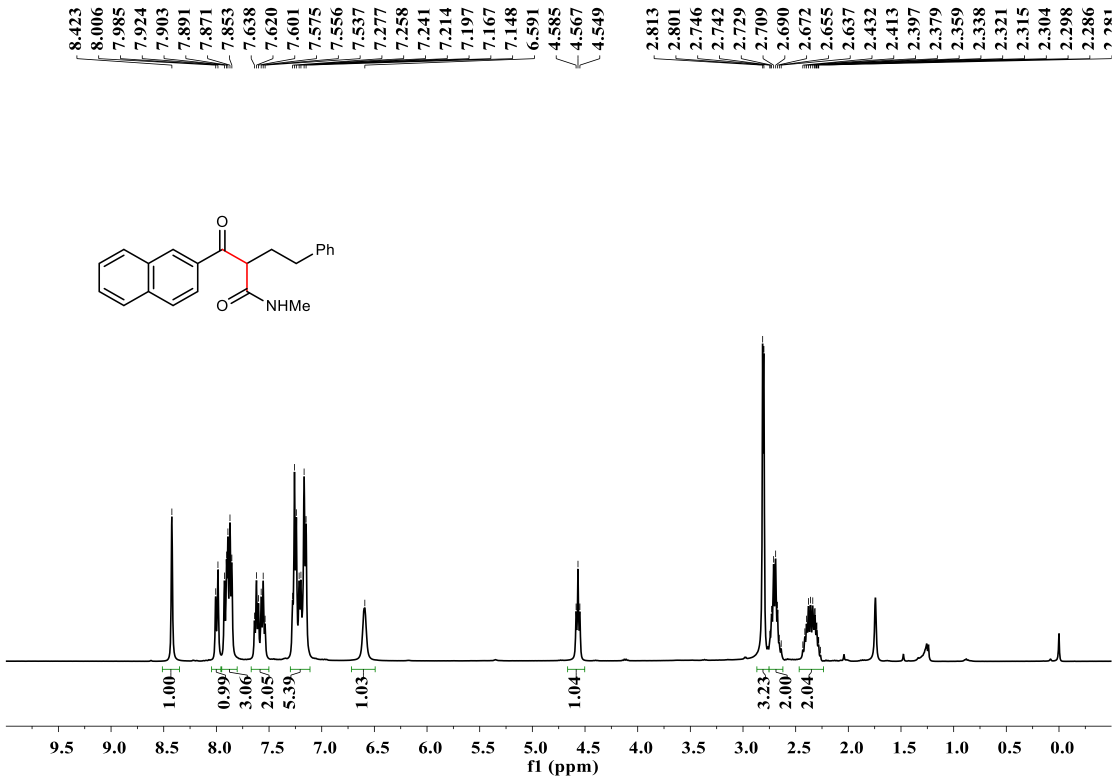

Supplementary Figure 161. ^{1}H NMR spectrum for **KA5**

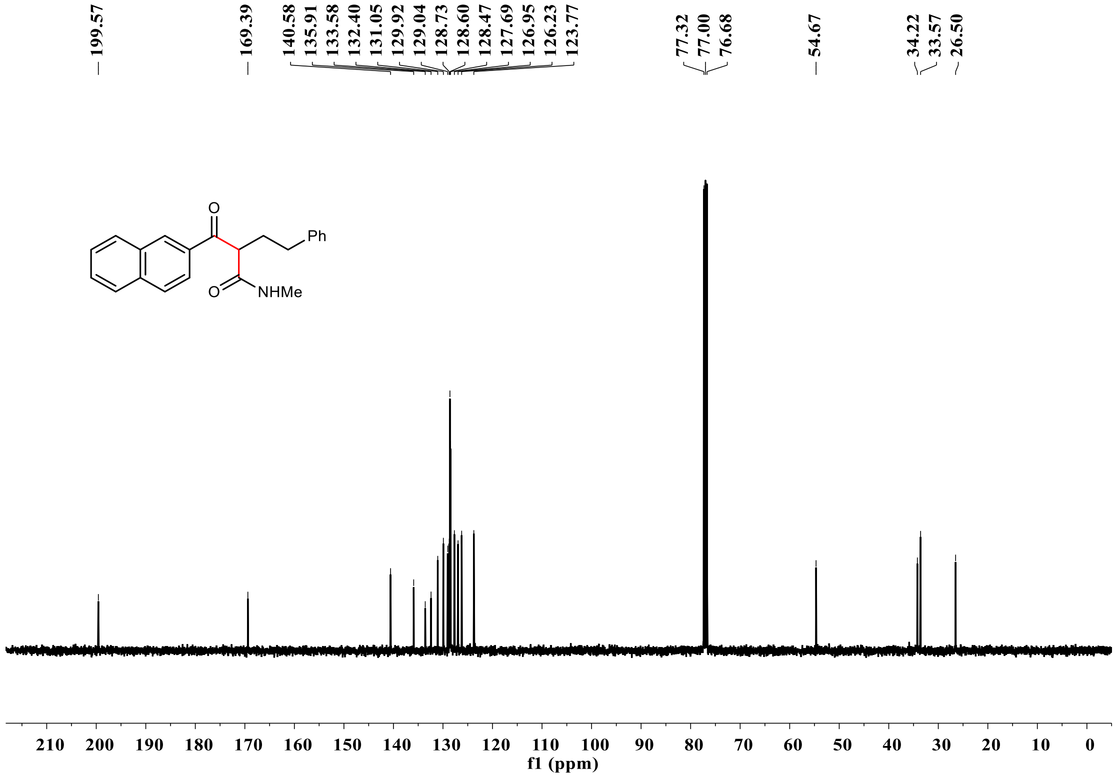

Supplementary Figure 162. ^{13}C NMR spectrum for **KA5**

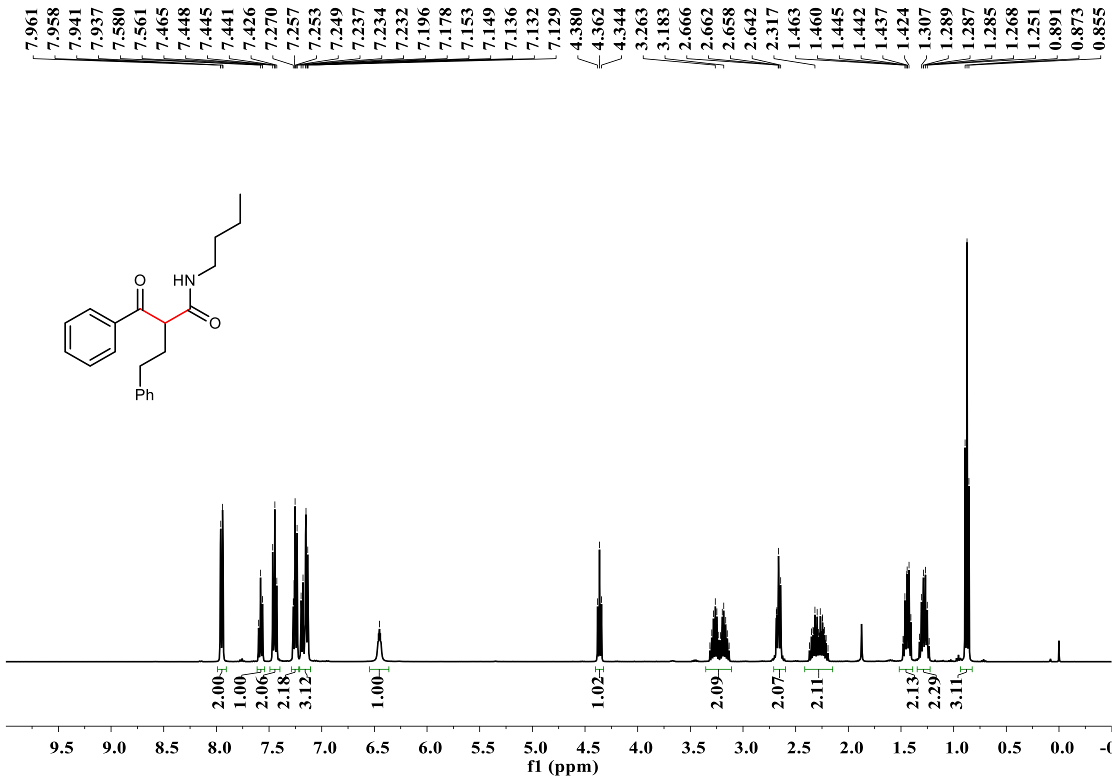

Supplementary Figure 163. ^{1}H NMR spectrum for **KA6**

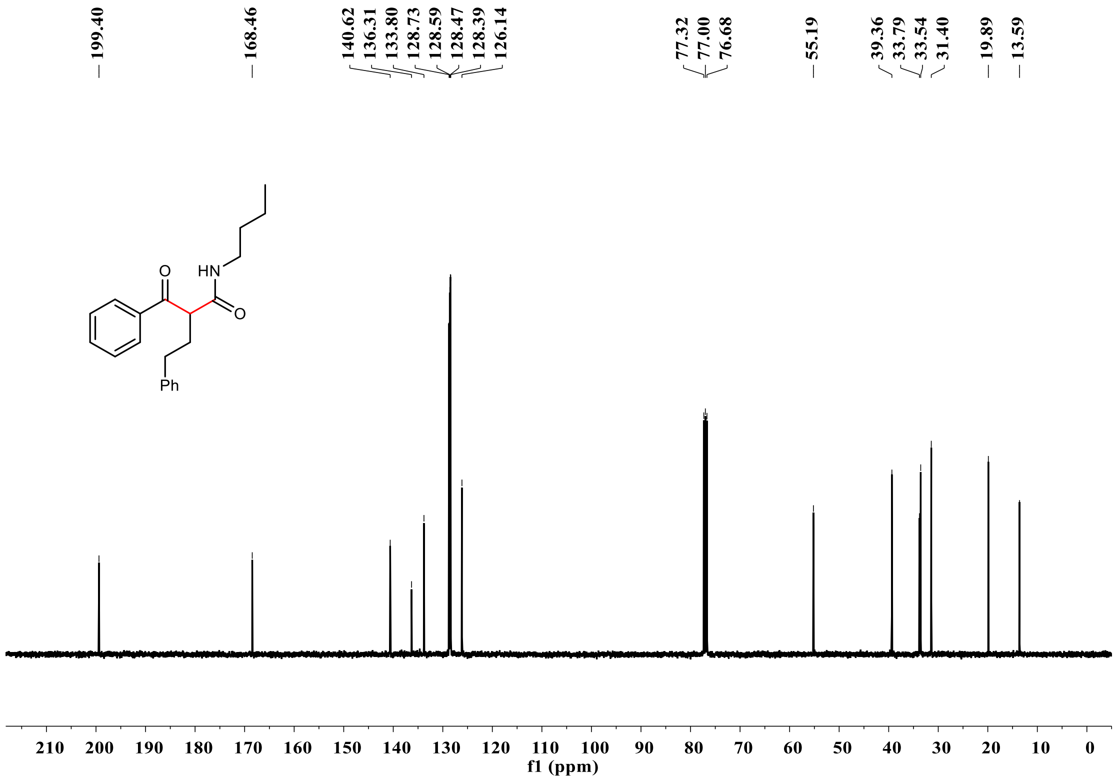

Supplementary Figure 164. ^{13}C NMR spectrum for **KA6**

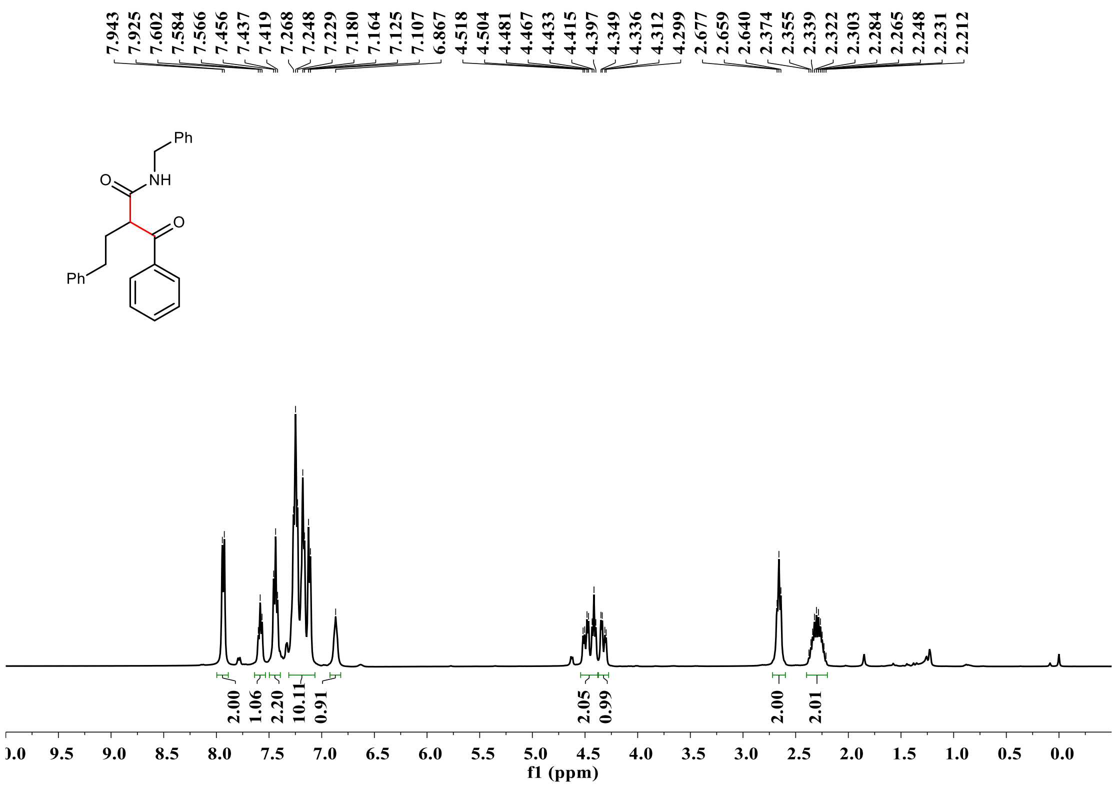

Supplementary Figure 165. ^{1}H NMR spectrum for **KA7**

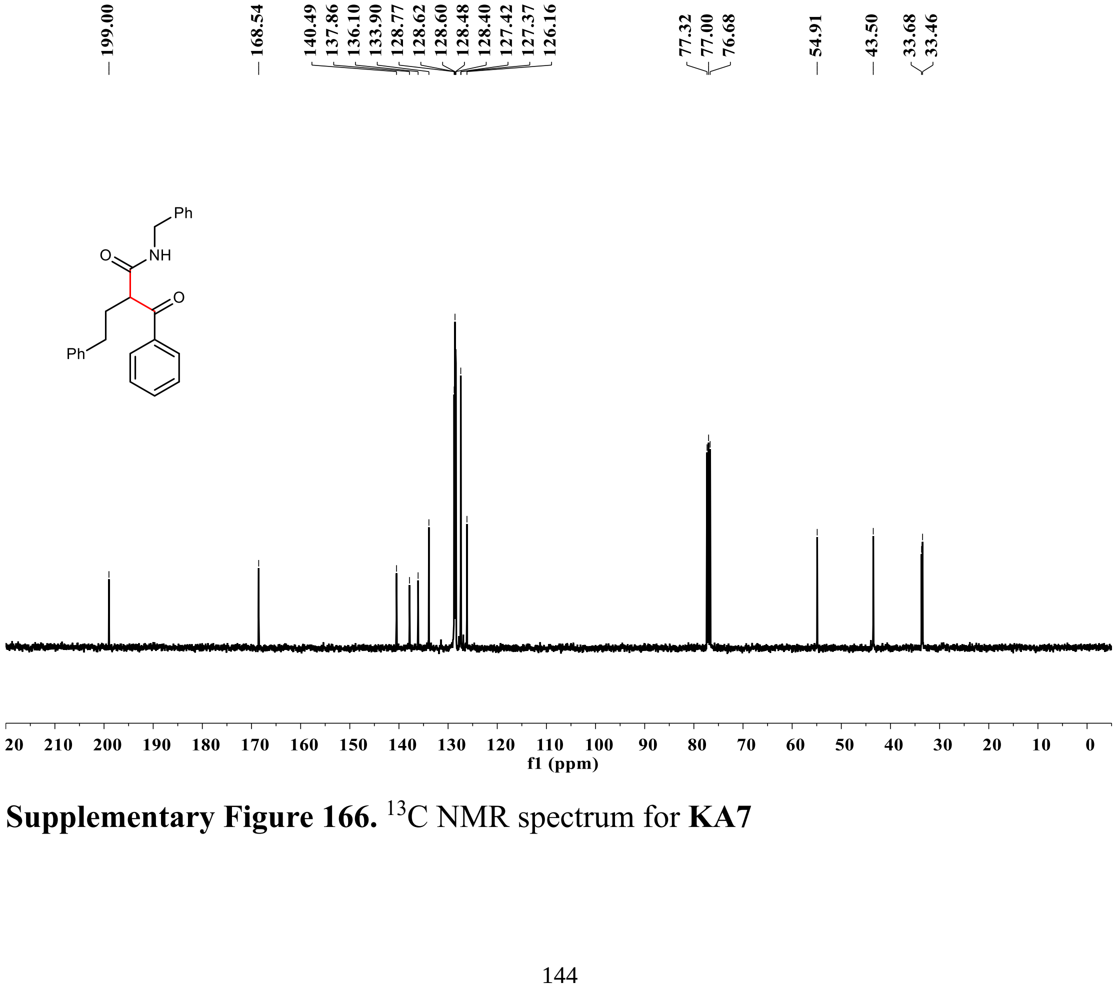

Supplementary Figure 166. ^{13}C NMR spectrum for **KA7**

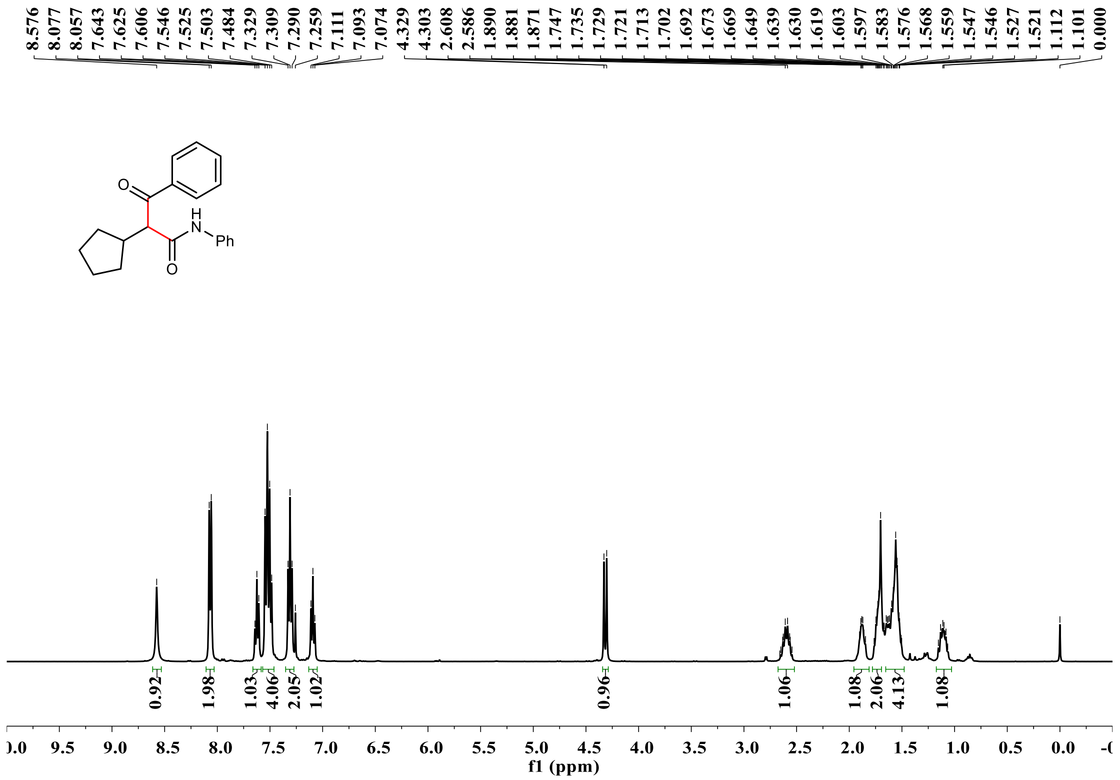

Supplementary Figure 167. ^{1}H NMR spectrum for **KA8**

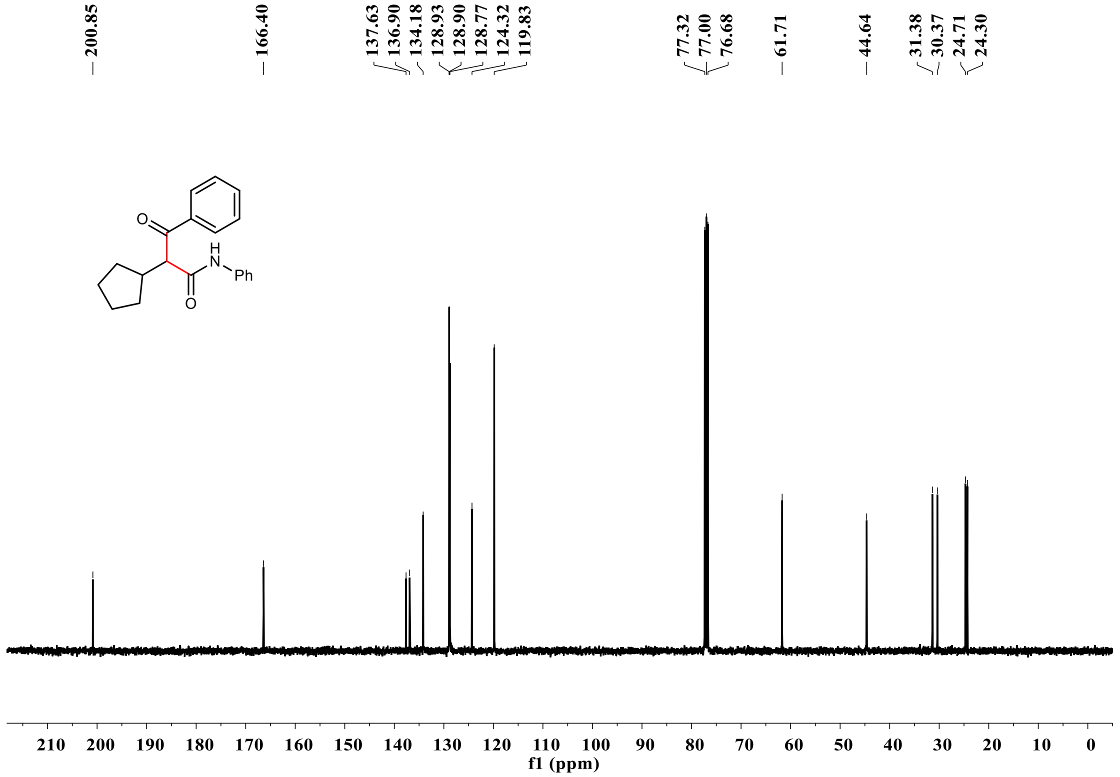

Supplementary Figure 168. ^{13}C NMR spectrum for **KA8**

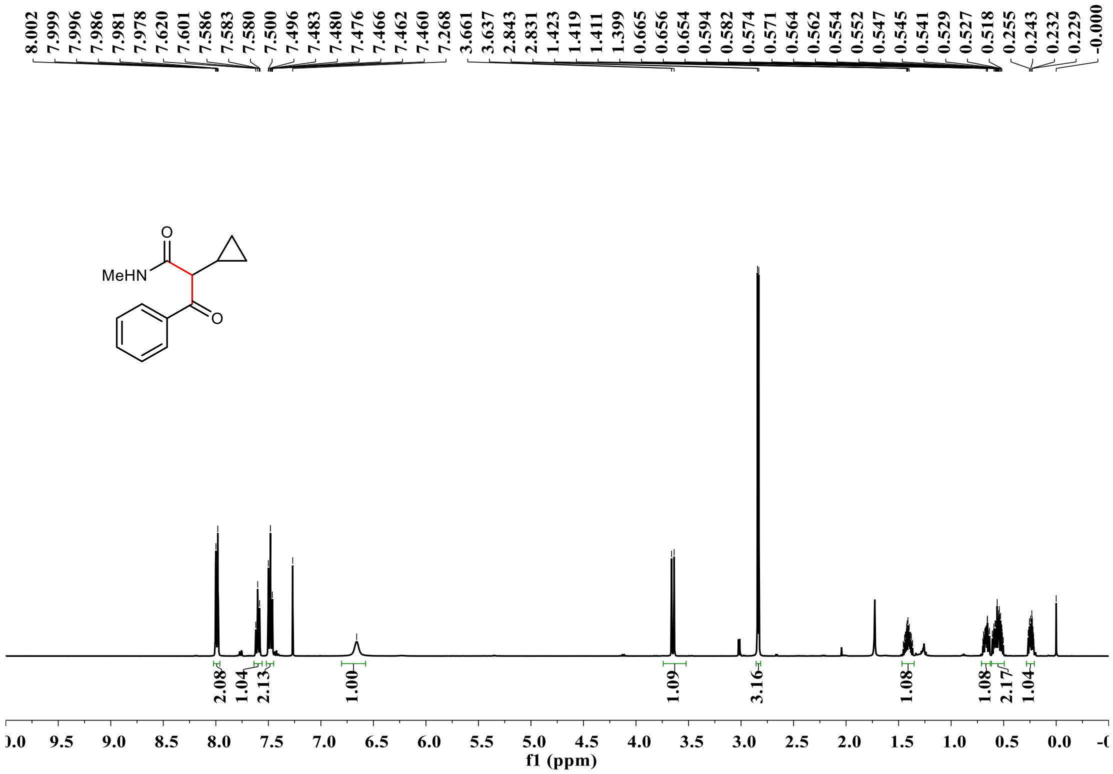

Supplementary Figure 169. ^{1}H NMR spectrum for **KA9**

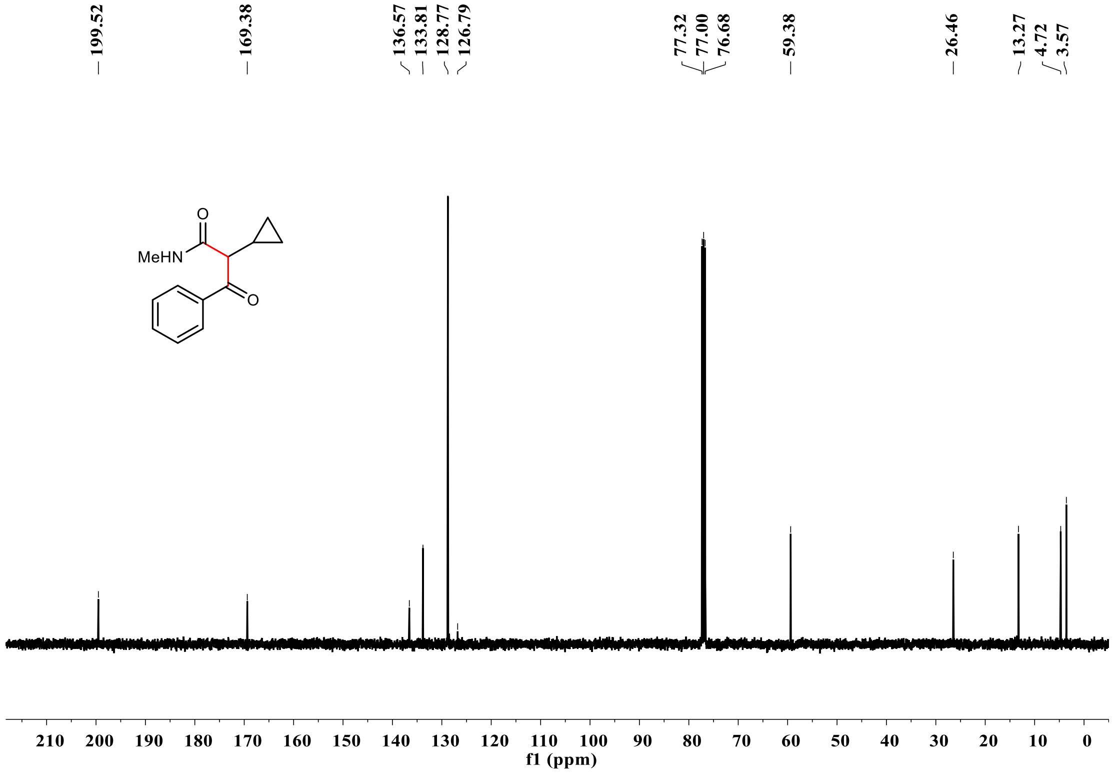

Supplementary Figure 170. ^{13}C NMR spectrum for **KA9**

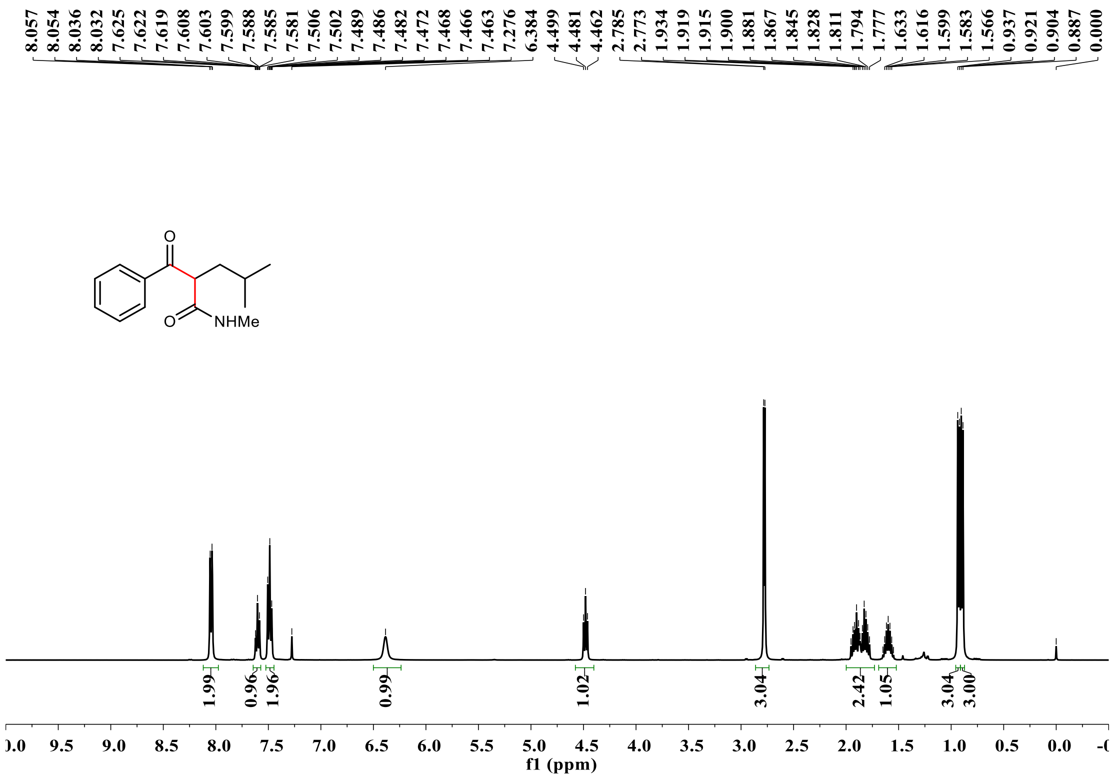

Supplementary Figure 171. ^{1}H NMR spectrum for **KA10**

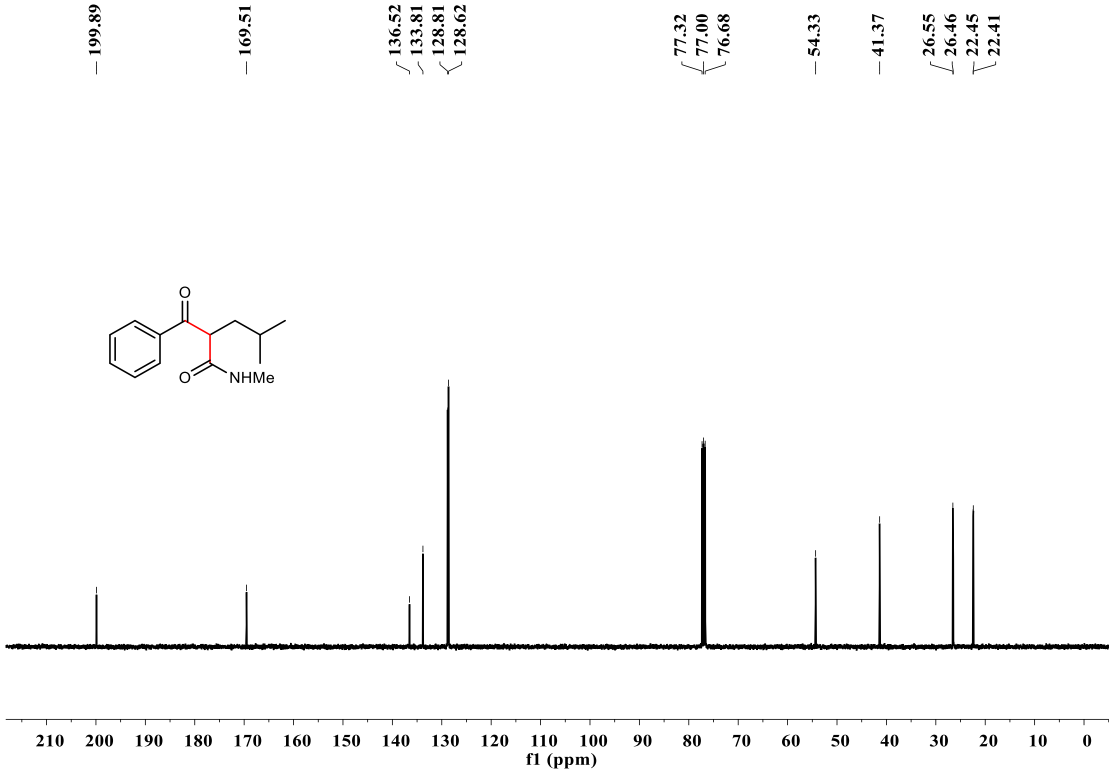

Supplementary Figure 172. ^{13}C NMR spectrum for **KA10**

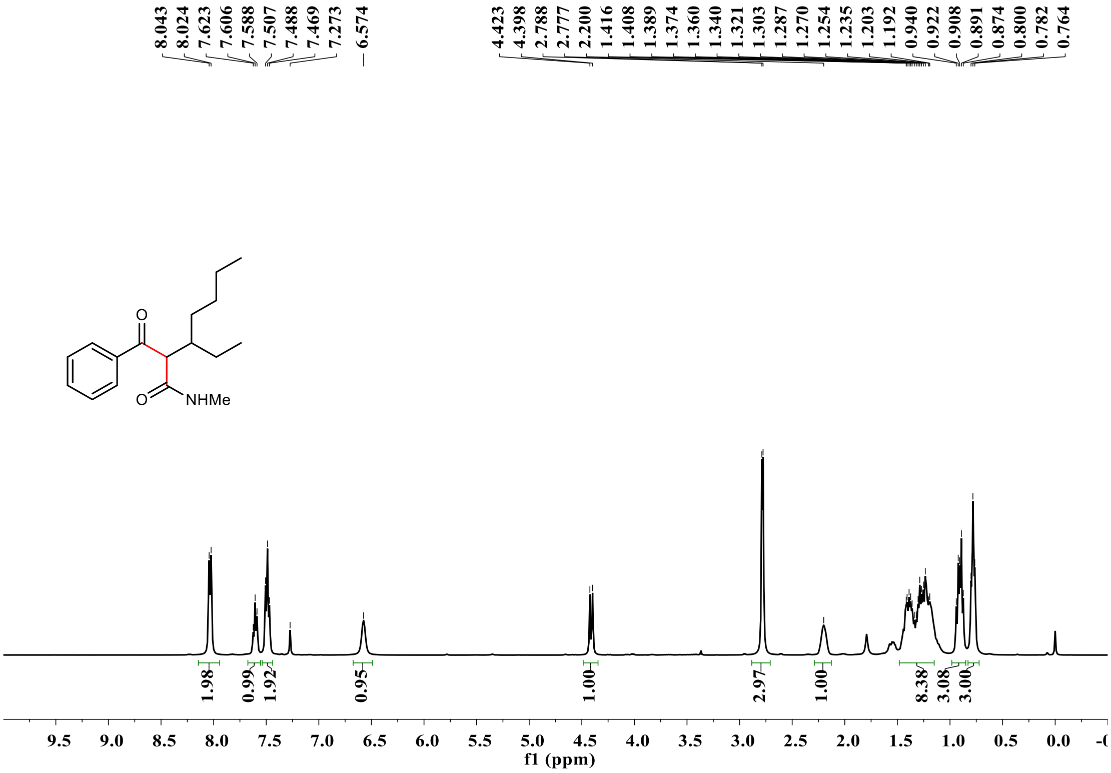

Supplementary Figure 173. ^{1}H NMR spectrum for **KA11**

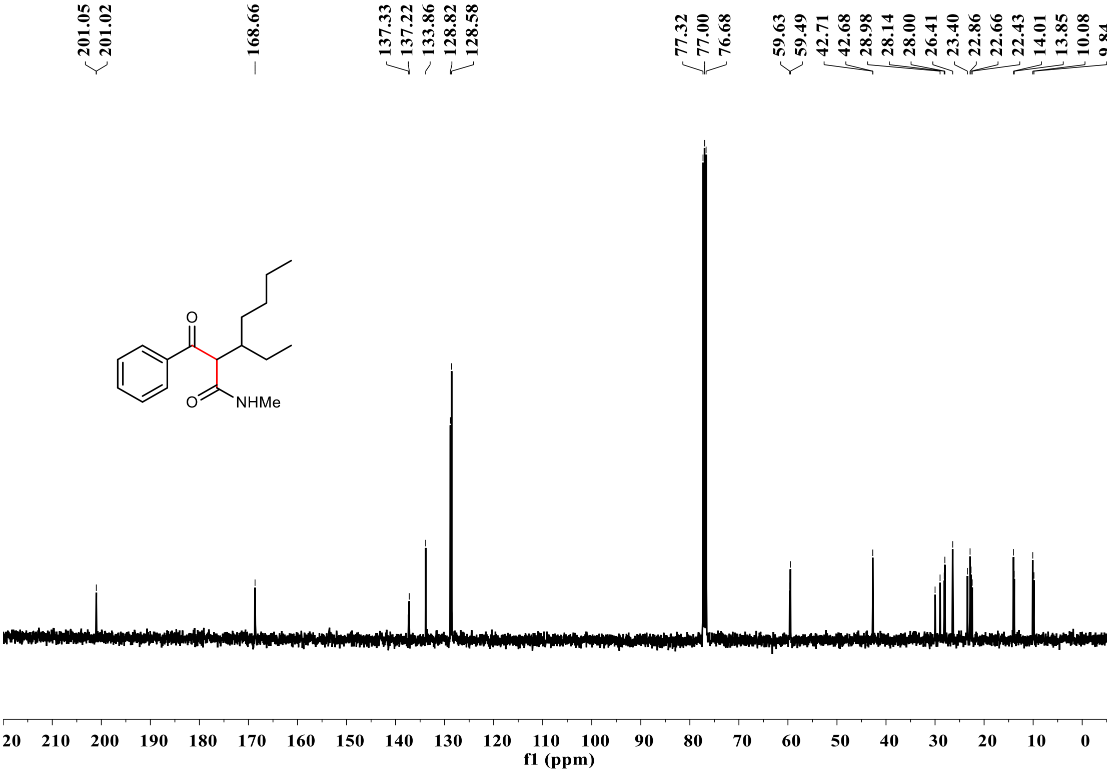

Supplementary Figure 174. ^{13}C NMR spectrum for **KA11**

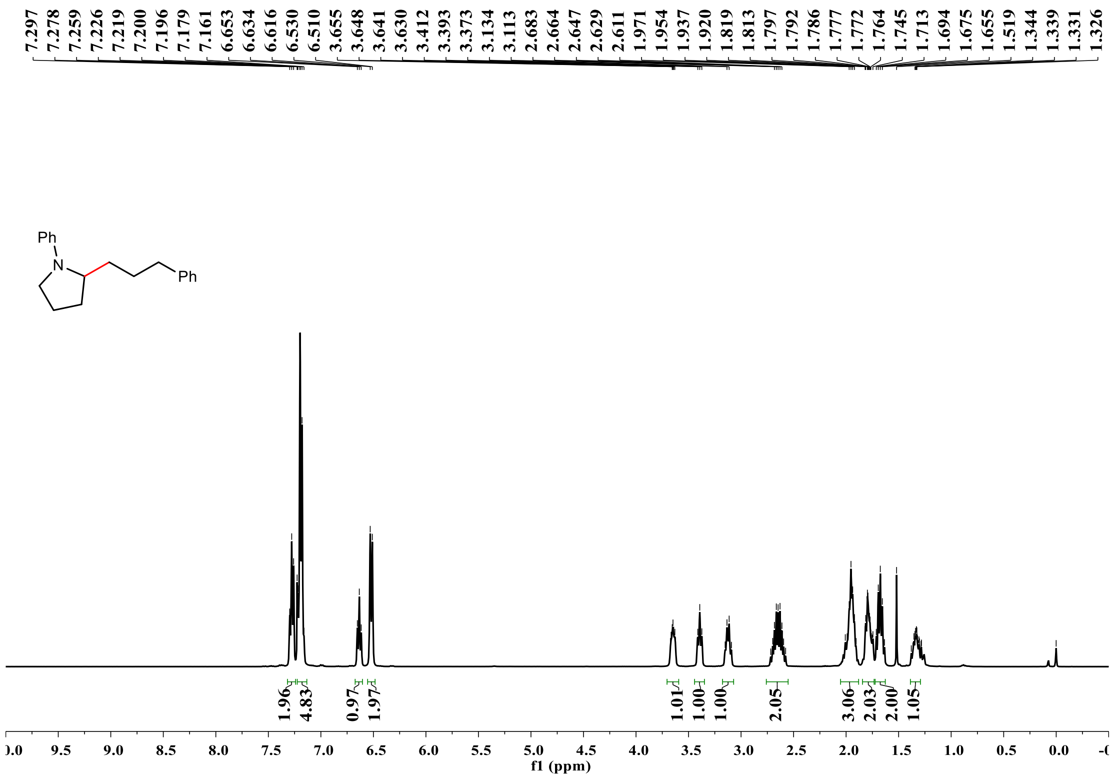

Supplementary Figure 175. ^{1}H NMR spectrum for **A1**

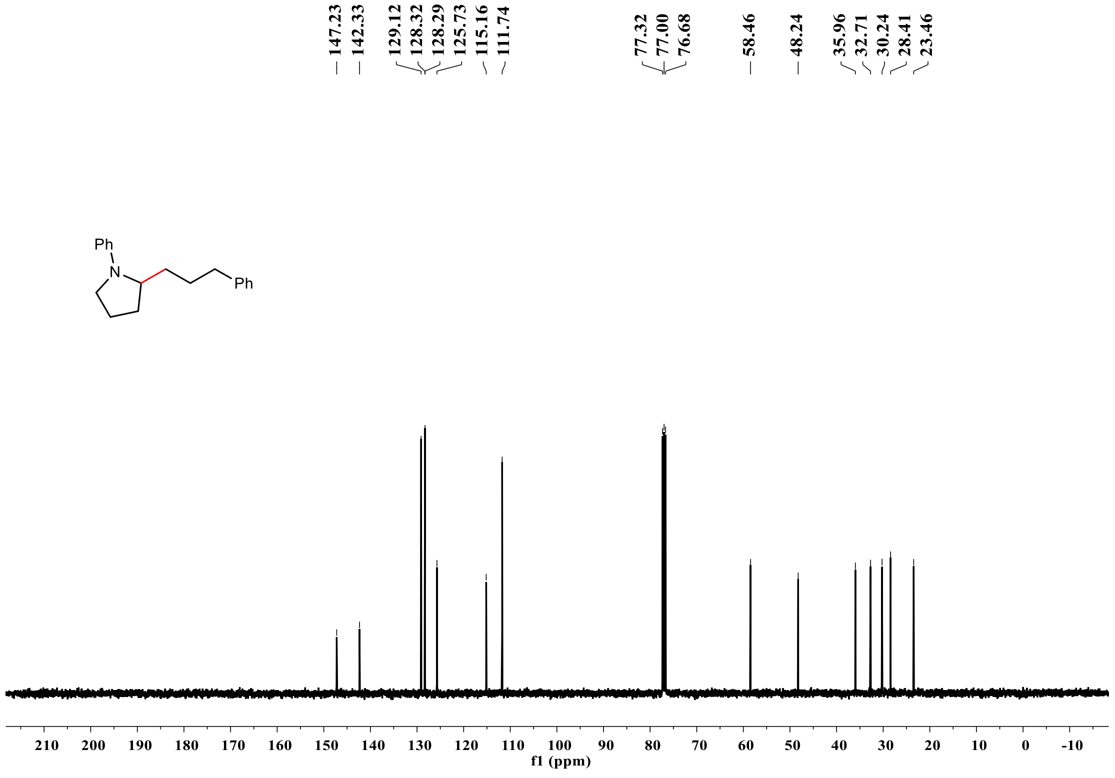

Supplementary Figure 176. ^{13}C NMR spectrum for **A1**

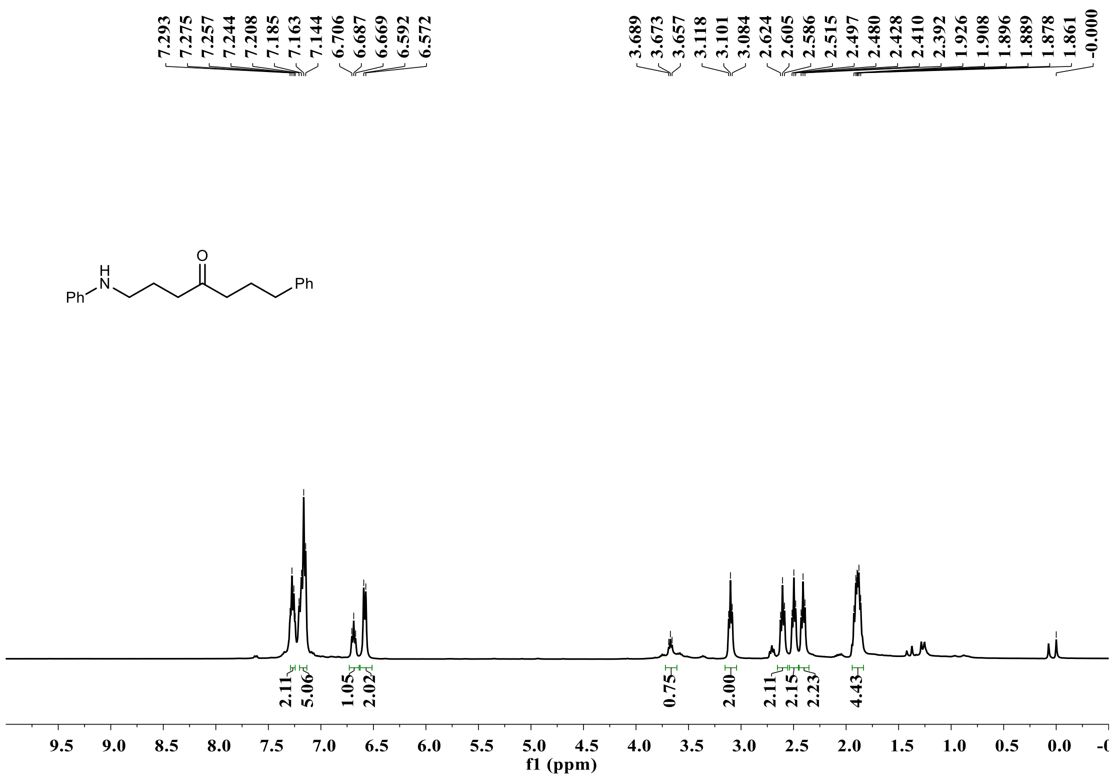

Supplementary Figure 177. ^{1}H NMR spectrum for **A1’**

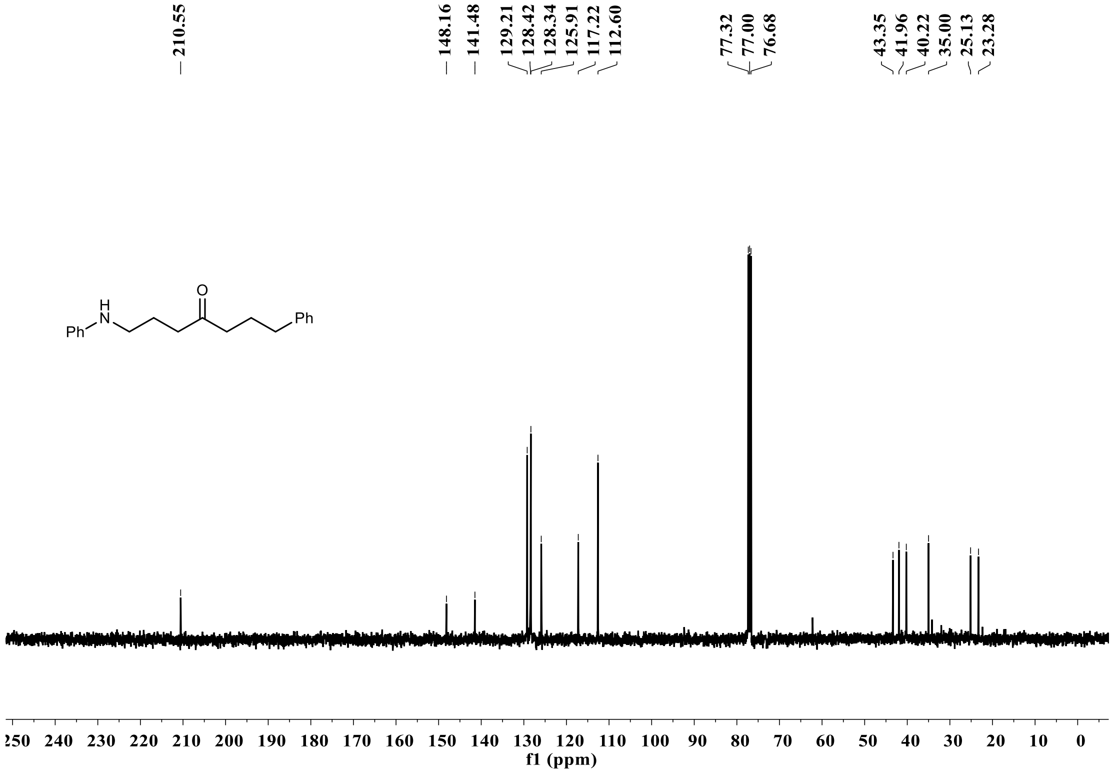

Supplementary Figure 178. ^{13}C NMR spectrum for **A1’**

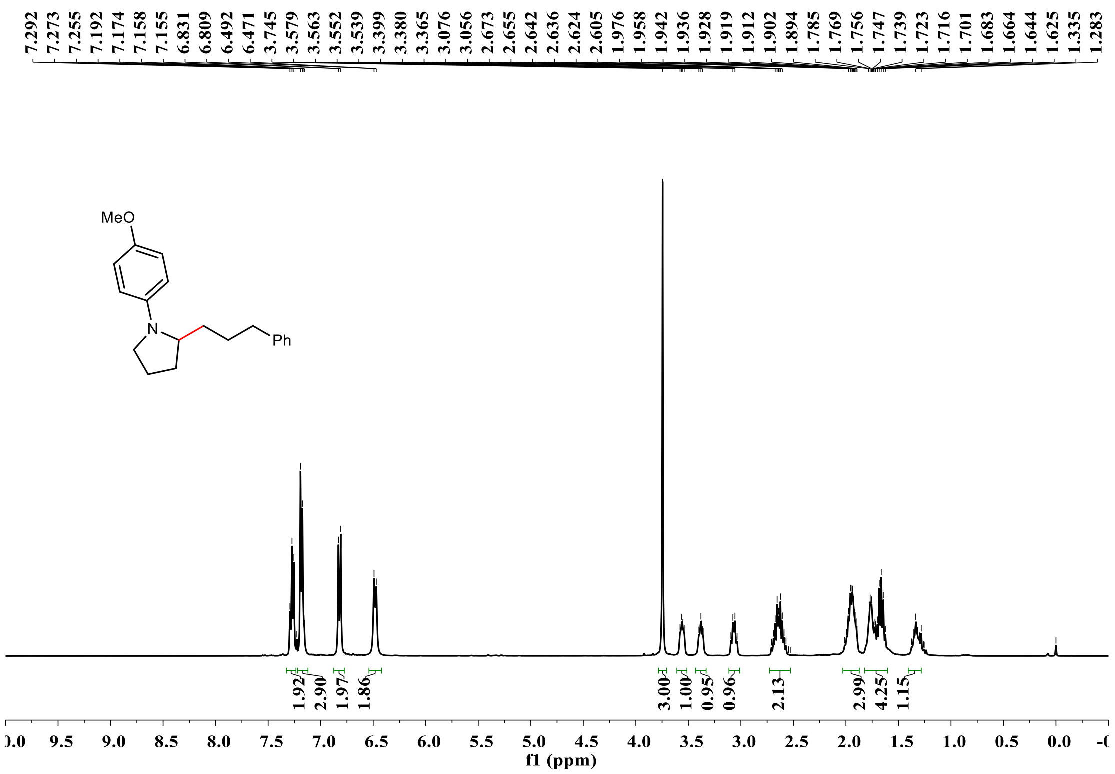

Supplementary Figure 179. ^{1}H NMR spectrum for **A2**

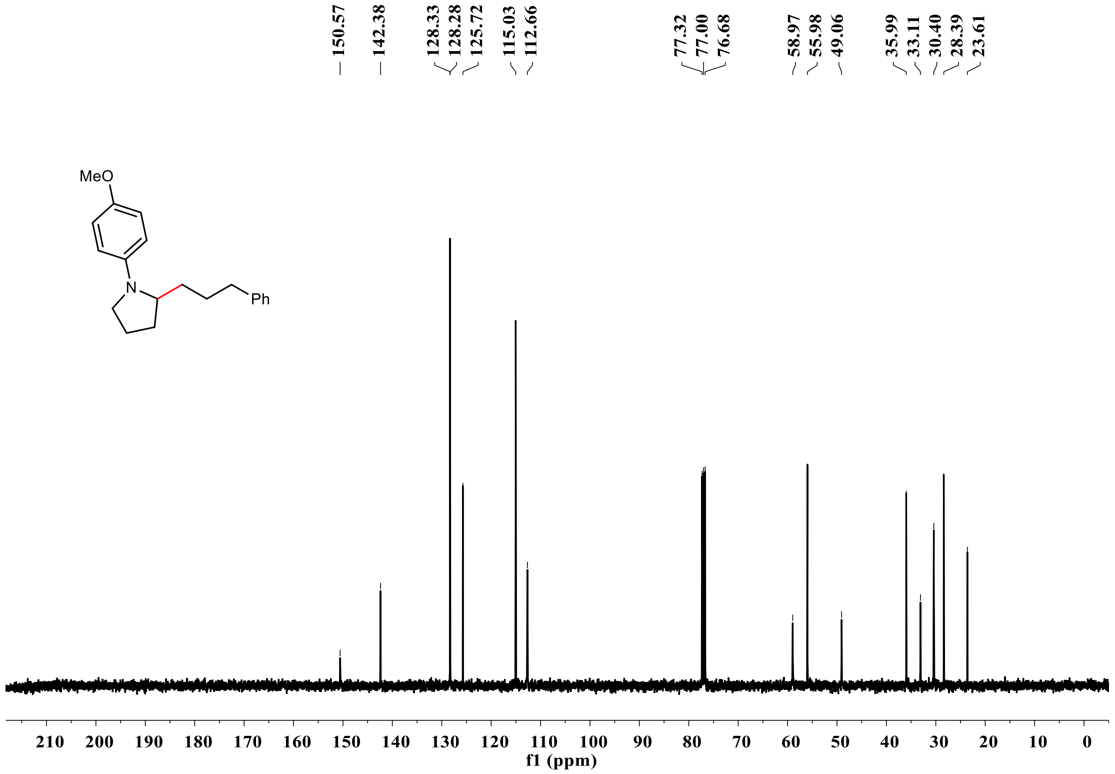

Supplementary Figure 180. ^{13}C NMR spectrum for **A2**

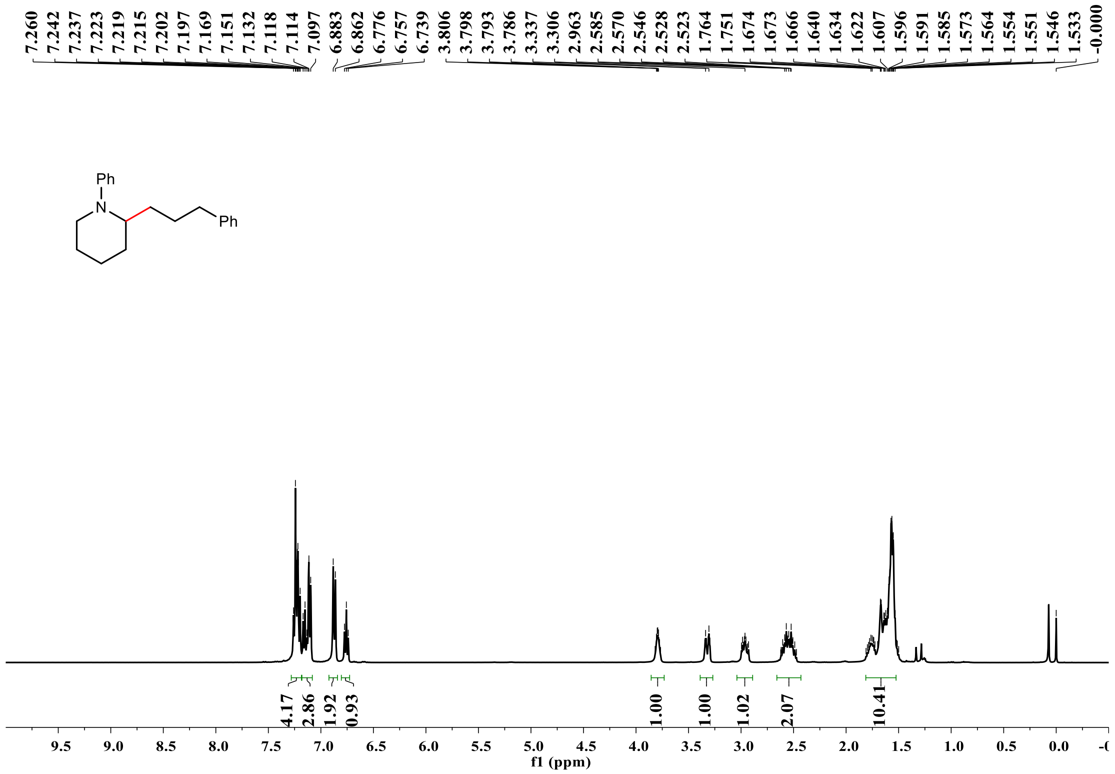

Supplementary Figure 181. ^{1}H NMR spectrum for **A3**

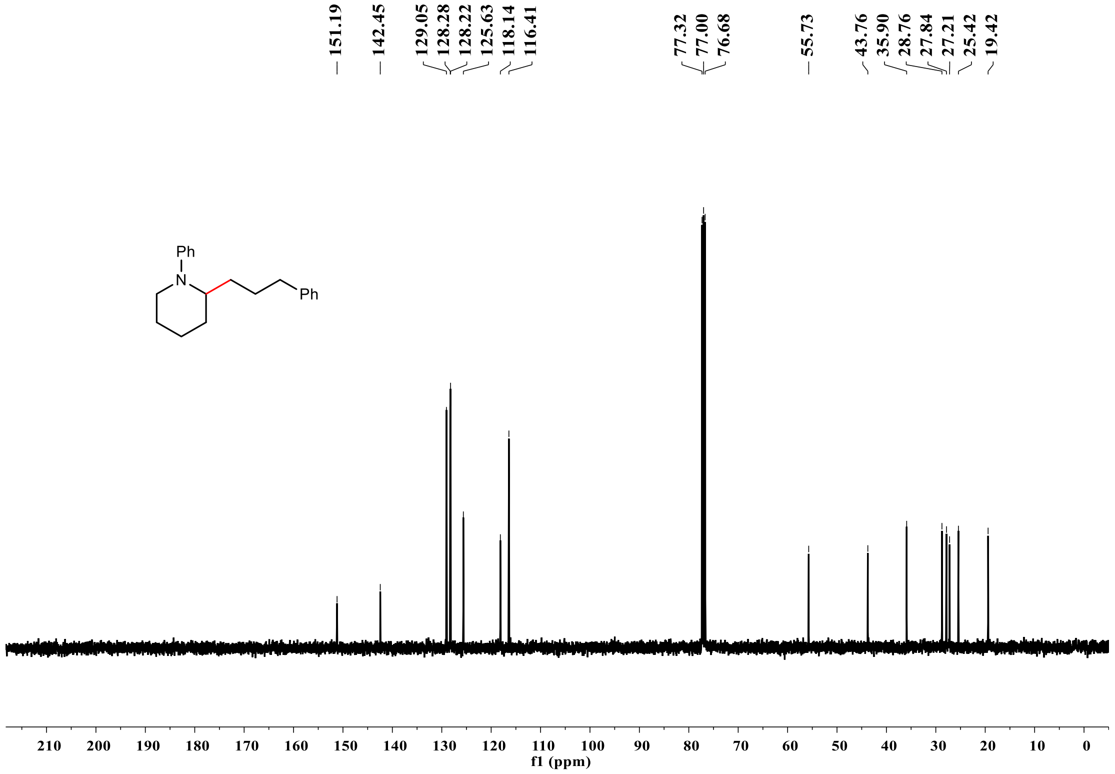

Supplementary Figure 182. ^{13}C NMR spectrum for **A3**

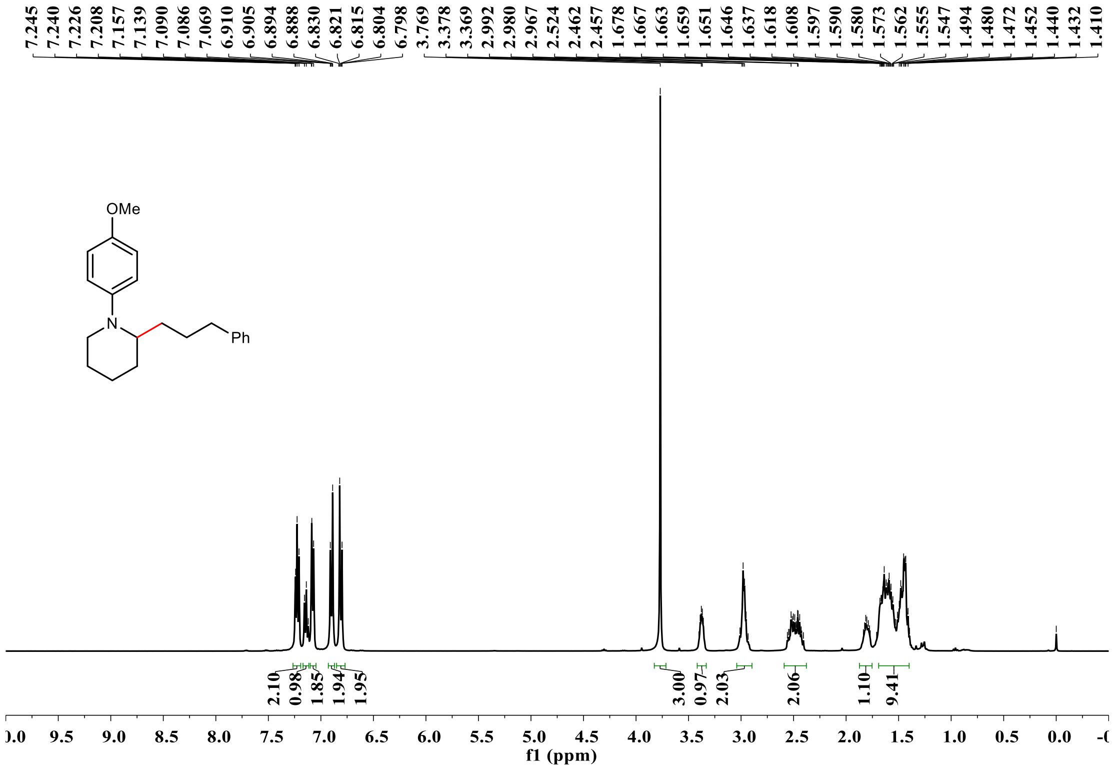

Supplementary Figure 183. ^{1}H NMR spectrum for **A4**

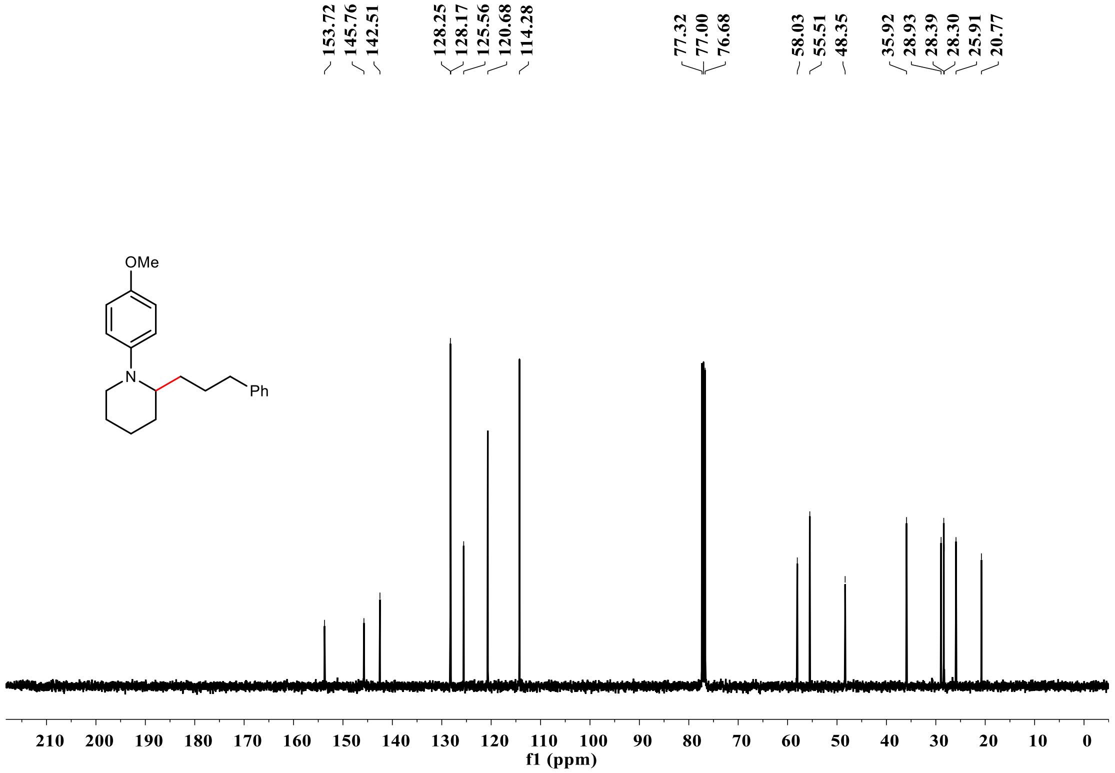

Supplementary Figure 184. ^{13}C NMR spectrum for **A4**

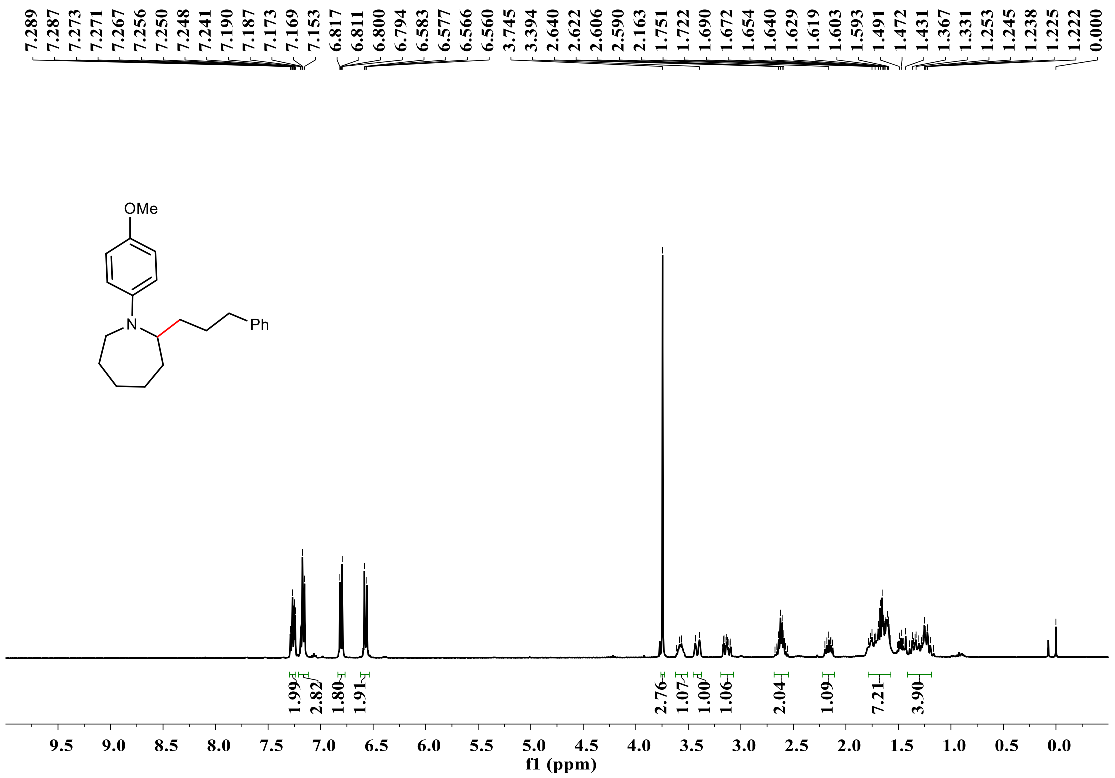

Supplementary Figure 185. ^{1}H NMR spectrum for **A5**

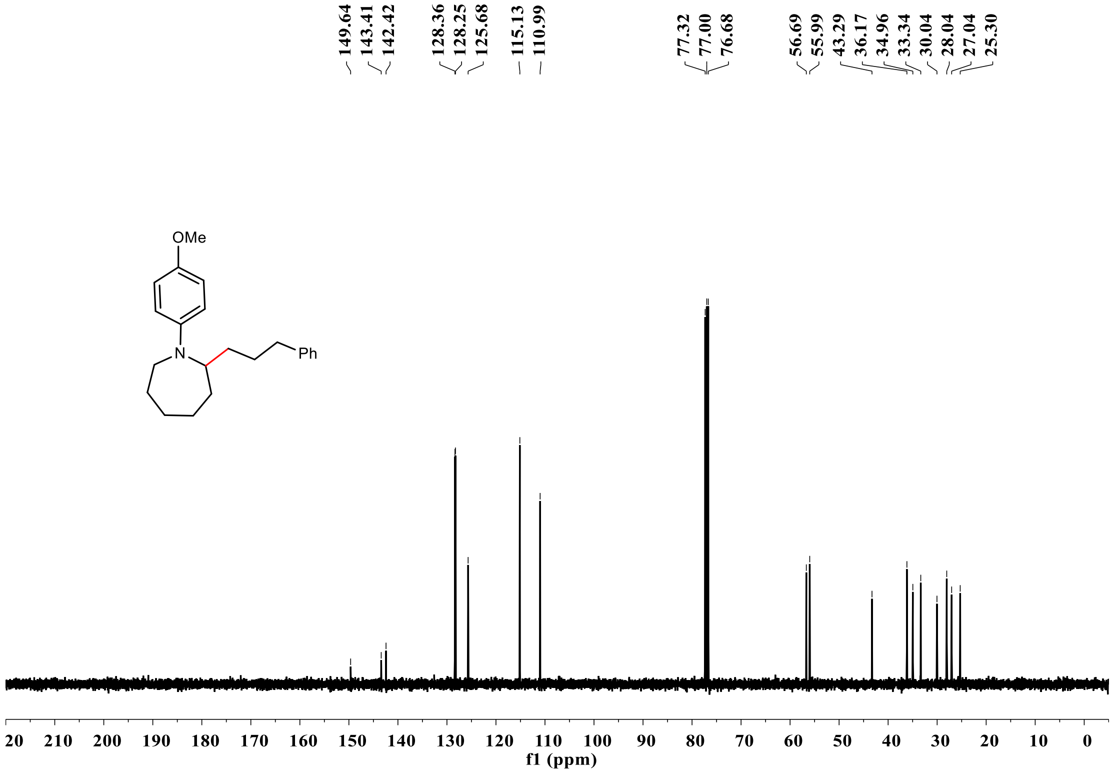

Supplementary Figure 186. ^{13}C NMR spectrum for **A5**

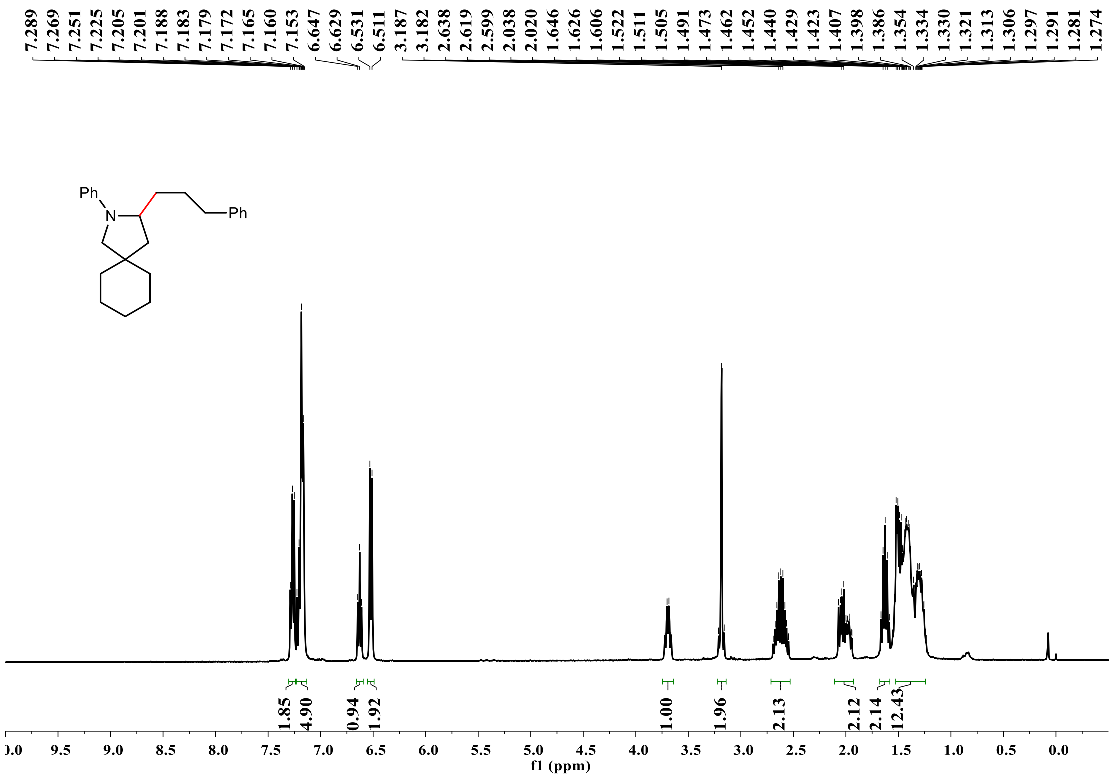

Supplementary Figure 187. ^{1}H NMR spectrum for **A6**

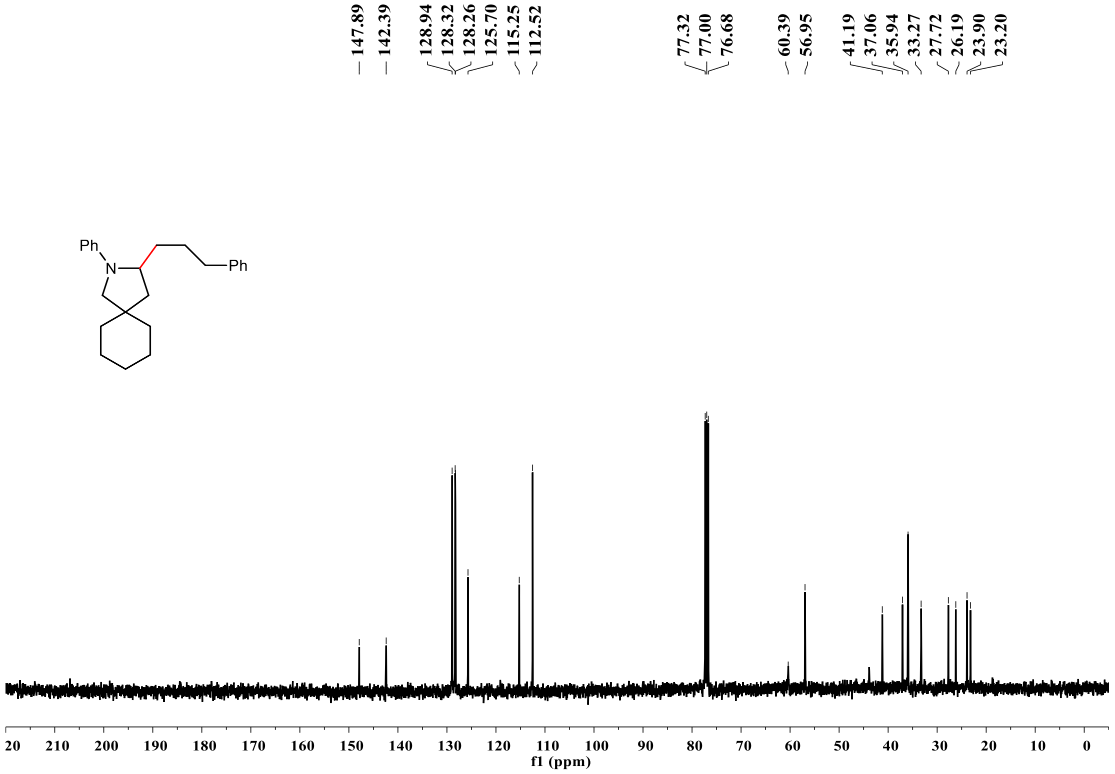

Supplementary Figure 188. ^{13}C NMR spectrum for **A6**

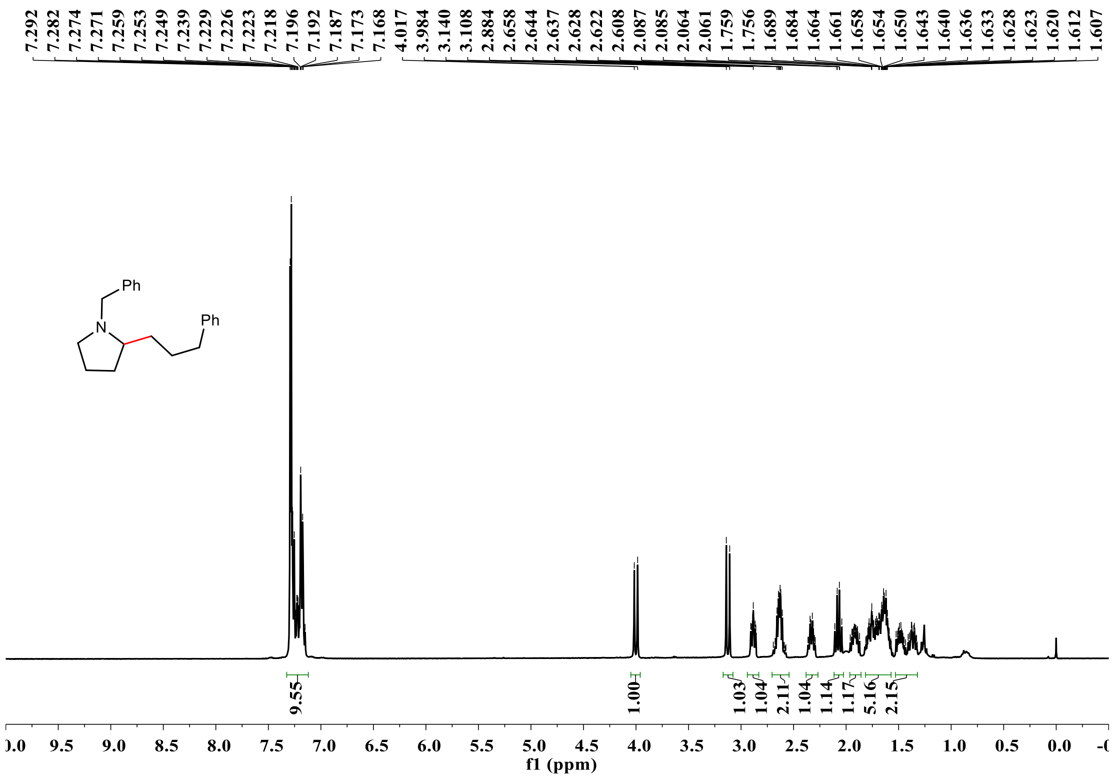

Supplementary Figure 189. ^{1}H NMR spectrum for **A7**

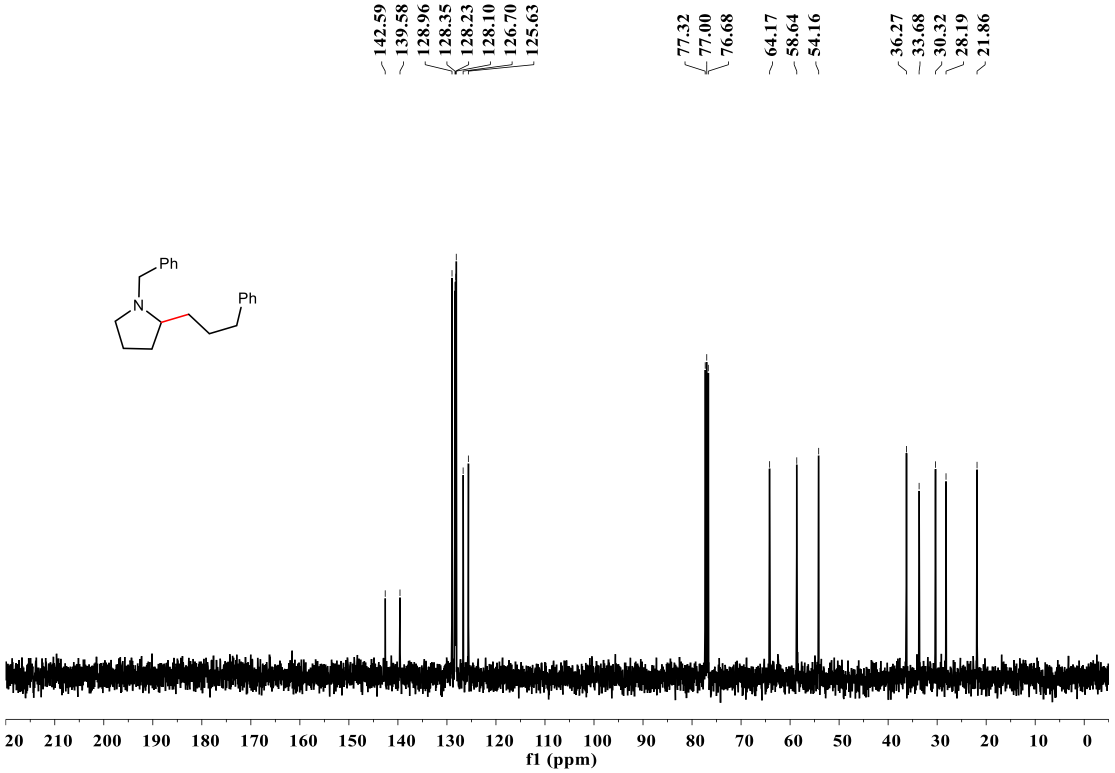

Supplementary Figure 190. ^{13}C NMR spectrum for **A7**

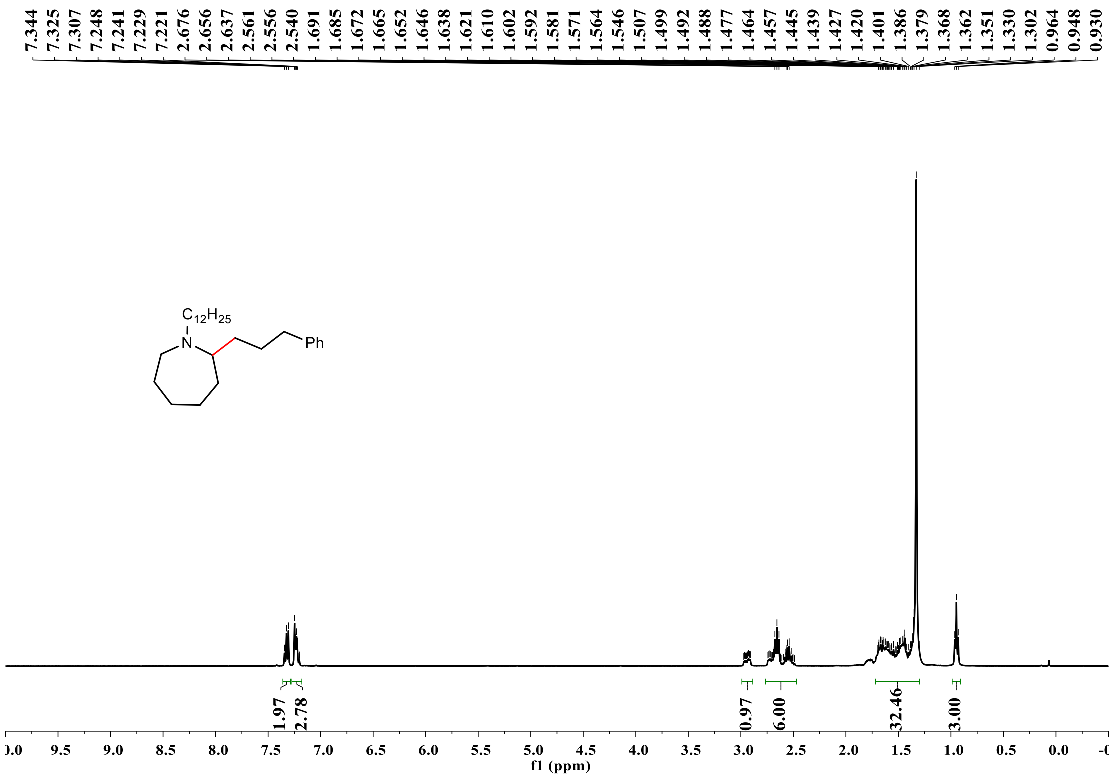

Supplementary Figure 191. ^{1}H NMR spectrum for **A8**

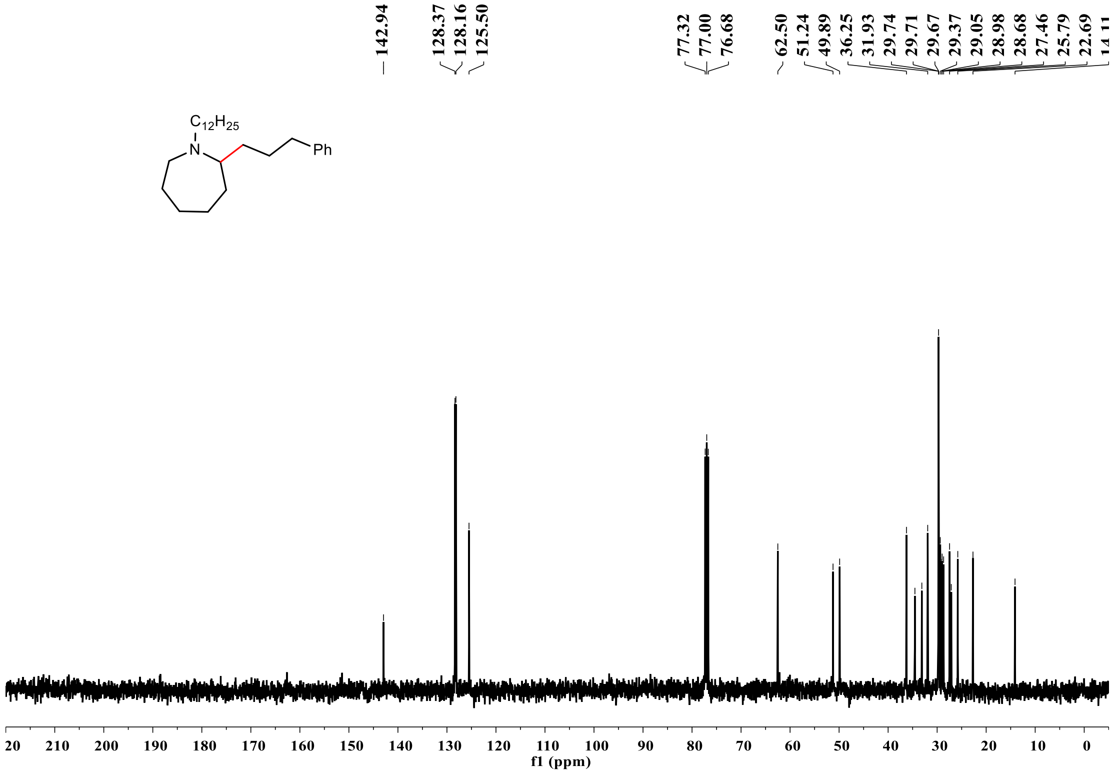

Supplementary Figure 192. ^{13}C NMR spectrum for **A8**

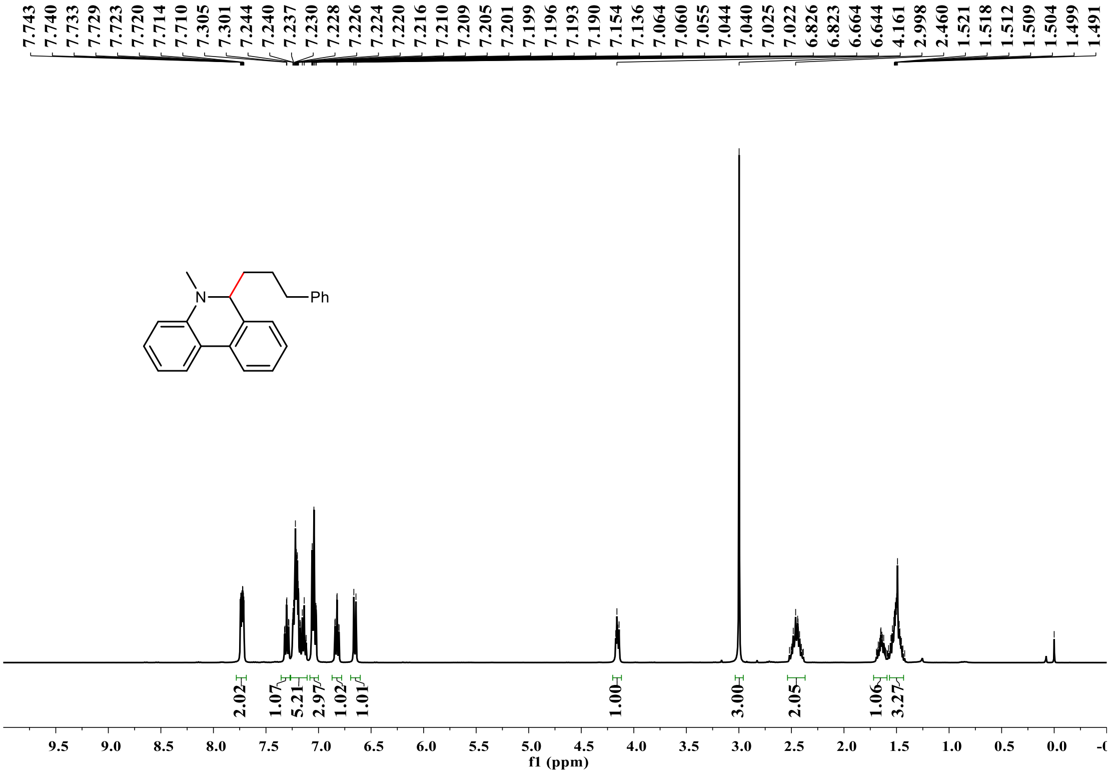

Supplementary Figure 193. ^{1}H NMR spectrum for **A9**

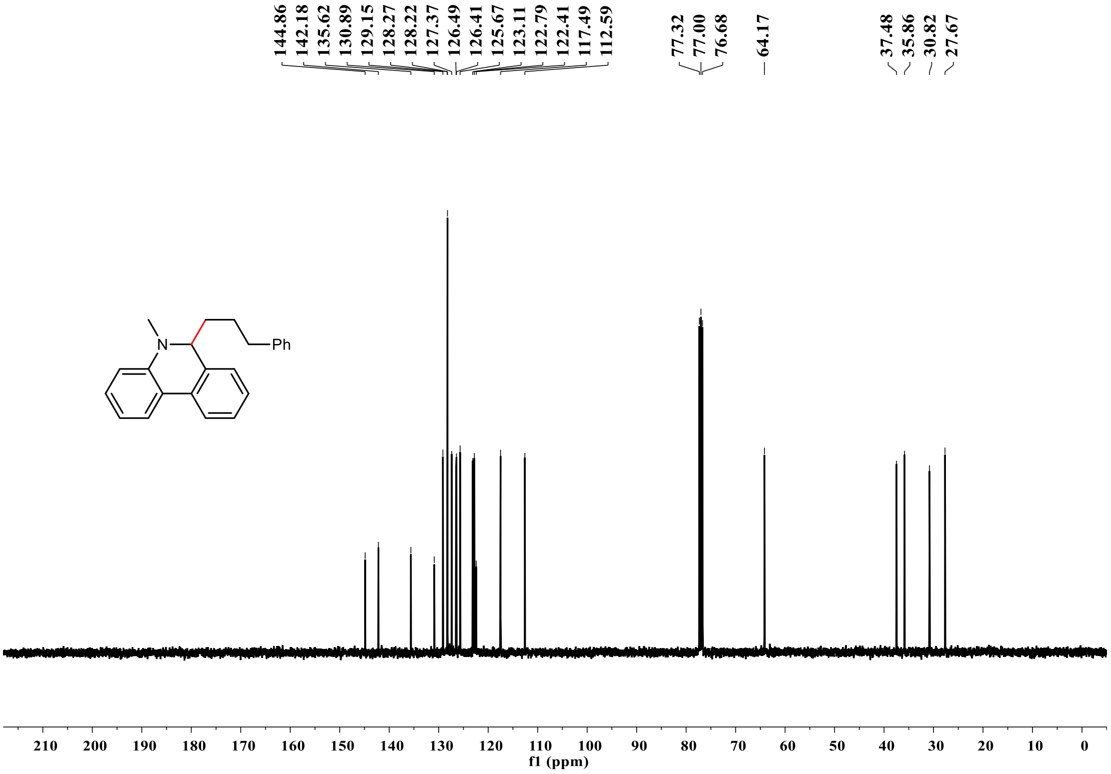

Supplementary Figure 194. ^{13}C NMR spectrum for **A9**

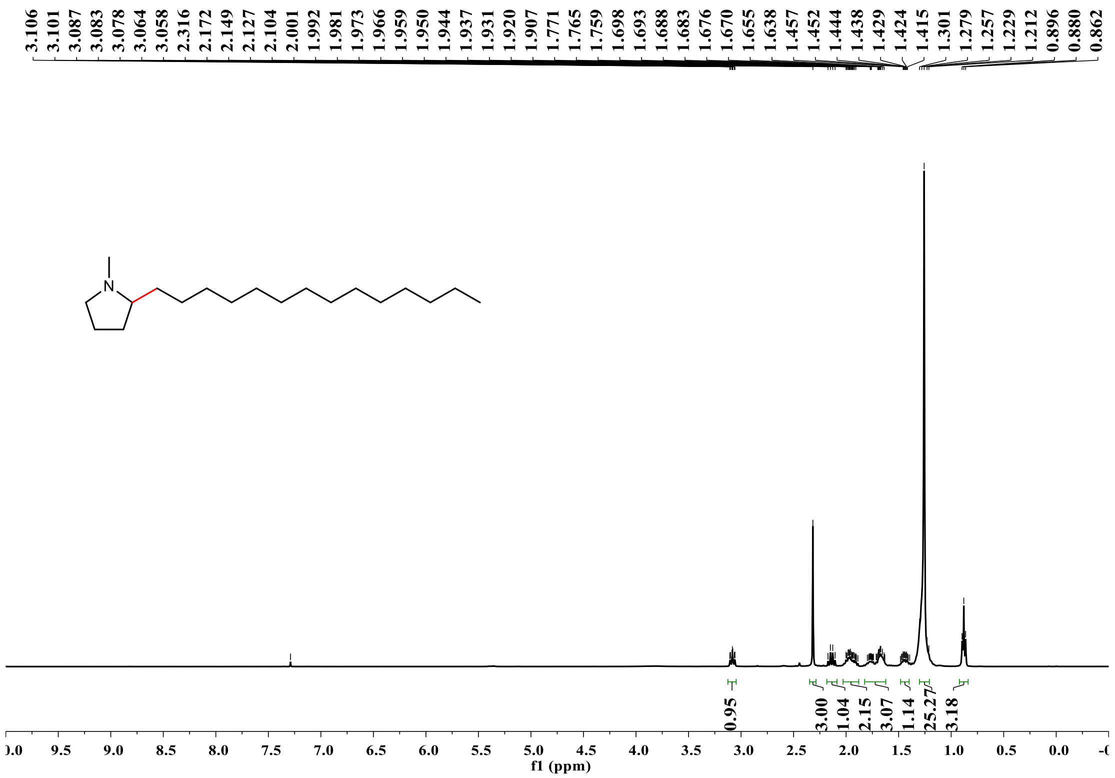

Supplementary Figure 195. ^{1}H NMR spectrum for **A10**

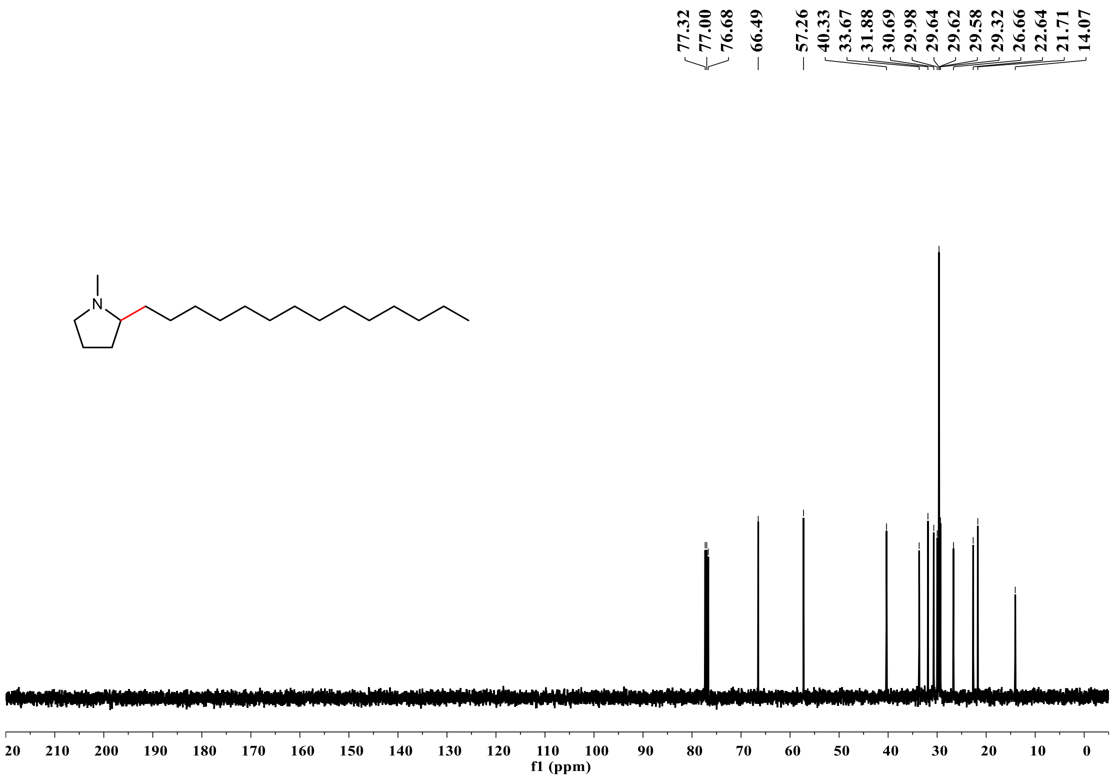

Supplementary Figure 196. ^{13}C NMR spectrum for **A10**

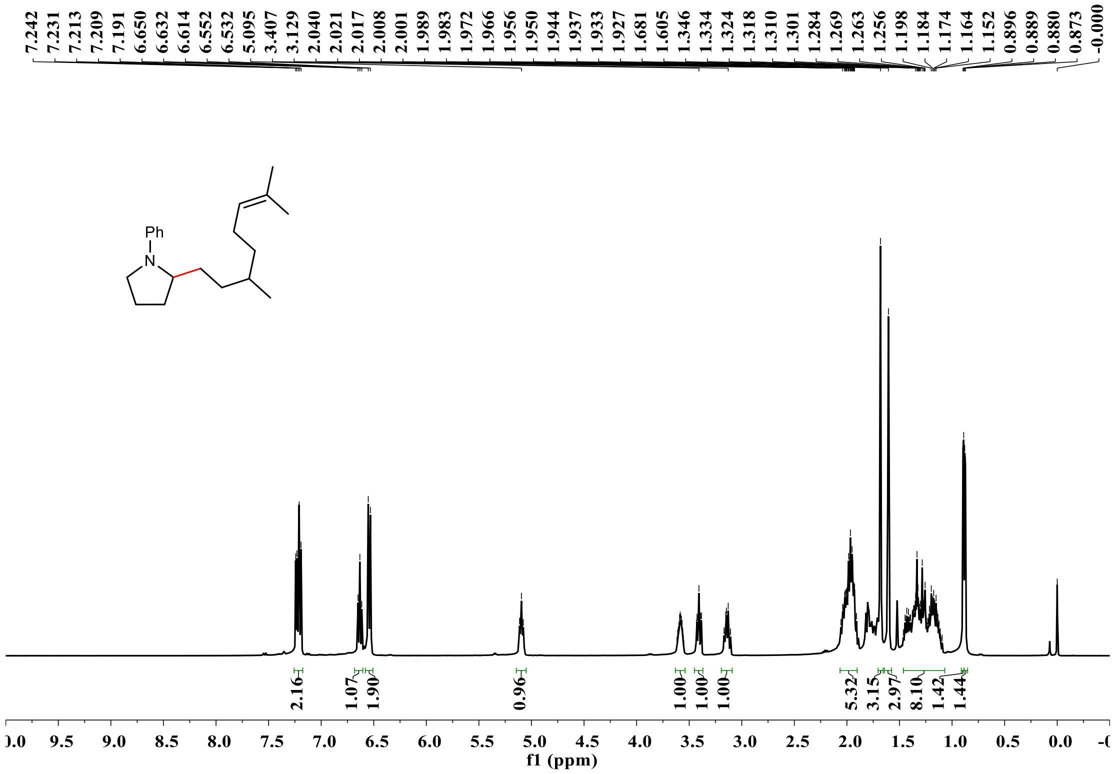

Supplementary Figure 197. ^{1}H NMR spectrum for **A11**

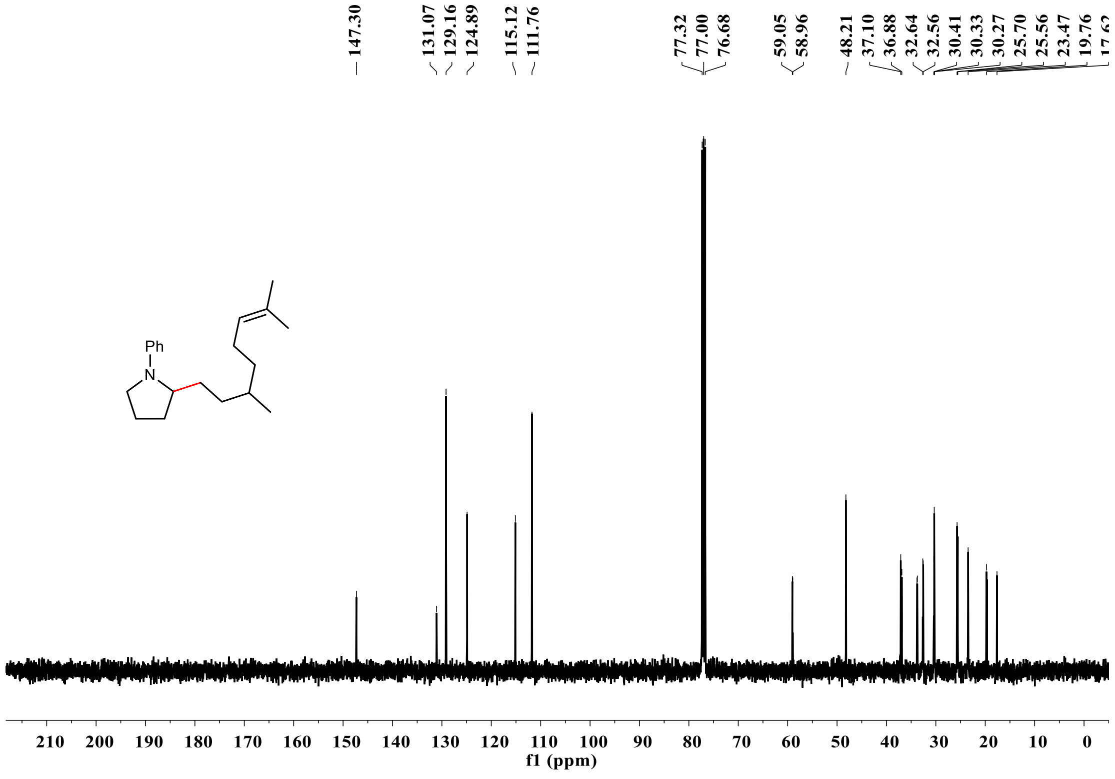

Supplementary Figure 198. ^{13}C NMR spectrum for **A11**

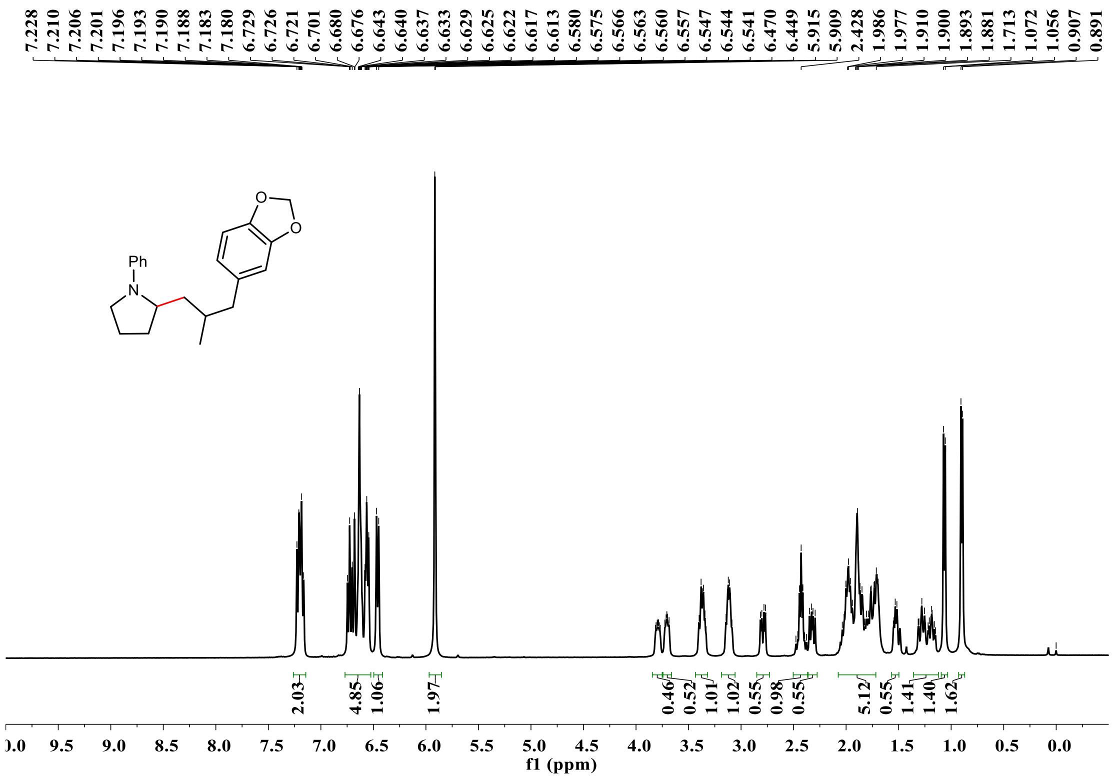

Supplementary Figure 199. ^{1}H NMR spectrum for **A12**

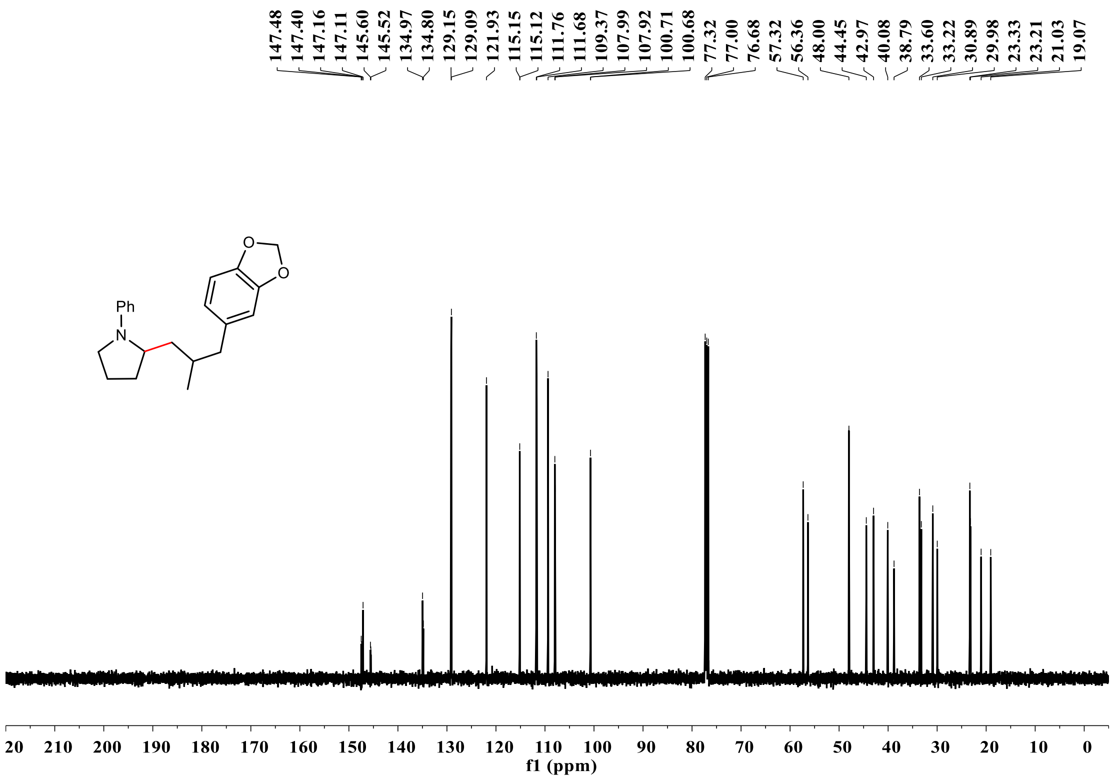

Supplementary Figure 200. ^{13}C NMR spectrum for **A12**

Supplementary References

1 Wang, L., Zhang, T., Sun, W., He, Z., Xia, C., Lan, Y. & Liu, C. C–O Functionalization of α-Oxyboronates: A Deoxygenative gem-Diborylation and gem-Silylborylation of Aldehydes and Ketones. *J. Am. Chem. Soc.* **139**, 5257-5264, (2017).

2 He, Z., Zhu, Q., Hu, X., Wang, L., Xia, C. & Liu, C. Cooperation between an alcoholic proton and boryl species in the catalytic gem-hydrodiborylation of carboxylic esters to access 1,1-diborylalkanes. *Org. Chem. Front.* **6**, 900-907, (2019).

3 Liu, X., Deaton, T. M., Haeffner, F. & Morken, J. P. A Boron Alkylidene–Alkene Cycloaddition Reaction: Application to the Synthesis of Aphanamal. *Angew. Chem. Int. Ed.* **56**, 11485-11489, (2017).

4 Song, L., Cao, G.-M., Zhou, W.-J., Ye, J.-H., Zhang, Z., Tian, X.-Y., Li, J. & Yu, D.-G. Pd-catalyzed carbonylation of aryl C–H bonds in benzamides with CO_2. *Org. Chem. Front.* **5**, 2086-2090, (2018).

5 Bisht, R., Hoque, M. E. & Chattopadhyay, B. Amide Effects in C−H Activation: Noncovalent Interactions with L-Shaped Ligand for meta Borylation of Aromatic Amides. *Angew. Chem. Int. Ed.* **57**, 15762-15766, (2018).

6 Sureshbabu, P., Azeez, S., Muniyappan, N., Sabiah, S. & Kandasamy, J. Chemoselective Synthesis of Aryl Ketones from Amides and Grignard Reagents via C(O)–N Bond Cleavage under Catalyst-Free Conditions. *J. Org. Chem.* **84**, 11823-11838, (2019).

7 Yin, J. & Buchwald, S. L. Pd-Catalyzed Intermolecular Amidation of Aryl Halides: The Discovery that Xantphos Can Be Trans-Chelating in a Palladium Complex. *J. Am. Chem. Soc.* **124**, 6043-6048, (2002).

8 Xing, X., O'Connor, N. R. & Stoltz, B. M. Palladium(II)-Catalyzed Allylic C H Oxidation of Hindered Substrates Featuring Tunable Selectivity Over Extent of Oxidation. *Angew. Chem. Int. Ed.* **54**, 11186-11190, (2015).

9 Das, D. & Samanta, R. Iridium(III)-Catalyzed Regiocontrolled Direct Amidation of Isoquinolones and Pyridones. *Adv. Synth. Catal.* **360**, 379-384, (2018).

10 Okamoto, I., Terashima, M., Masu, H., Nabeta, M., Ono, K., Morita, N., Katagiri, K., Azumaya, I. & Tamura, O. Acid-induced conformational alteration of cis-preferential aromatic amides bearing N-methyl-N-(2-pyridyl) moiety. *Tetrahedron* **67**, 8536-8543, (2011).

11 Liu, Z., Zhang, J., Chen, S., Shi, E., Xu, Y. & Wan, X. Cross Coupling of Acyl and Aminyl Radicals: Direct Synthesis of Amides Catalyzed by Bu4NI with TBHP as an Oxidant. *Angew. Chem. Int. Ed.* **51**, 3231-3235, (2012).

12 Paul, B., Maji, M. & Kundu, S. Atom-Economical and Tandem Conversion of Nitriles to N-Methylated Amides Using Methanol and Water. *ACS Catal.* **9**, 10469-10476, (2019).

13 Sklyaruk, J., Borghs, J. C., El-Sepelgy, O. & Rueping, M. Catalytic C1 Alkylation with Methanol and Isotope-Labeled Methanol. *Angew. Chem. Int. Ed.* **58**, 775-779, (2019).

14 Kolsi, L. E., Krogerus, S., Brito, V., Rüffer, T., Lang, H., Yli-Kauhaluoma, J., Silvestre, S. M. & Moreira, V. M. Regioselective Benzylic Oxidation of Aromatic Abietanes: Application to the Semisynthesis of the Naturally Occurring Picealactones A, B and C. *ChemistrySelect* **2**, 7008-7012, (2017).

15 Sun, W., Wang, L., Xia, C. & Liu, C. Dual Functionalization of α-Monoboryl Carbanions through Deoxygenative Enolization with Carboxylic Acids. *Angew. Chem. Int. Ed.* **57**, 5501-5505, (2018).

16 Xia, Z.-H., Zhang, C.-L., Gao, Z.-H. & Ye, S. Switchable Decarboxylative Heck-Type Reaction and

Oxo-alkylation of Styrenes with N-Hydroxyphthalimide Esters under Photocatalysis. *Org. Lett.* **20**, 3496-3499, (2018).

17 Li, L., Cai, P., Guo, Q. & Xue, S. Et2Zn-Mediated Rearrangement of Bromohydrins. *J. Org. Chem.* **73**, 3516-3522, (2008).

18 Kwon, M. S., Kim, N., Seo, S. H., Park, I. S., Cheedrala, R. K. & Park, J. Recyclable Palladium Catalyst for Highly Selective α Alkylation of Ketones with Alcohols. *Angew. Chem. Int. Ed.* **44**, 6913-6915, (2005).

19 Biosca, M., Pàmies, O. & Diéguez, M. Giving a Second Chance to Ir/Sulfoximine-Based Catalysts for the Asymmetric Hydrogenation of Olefins Containing Poorly Coordinative Groups. *J. Org. Chem.* **84**, 8259-8266, (2019).

20 Stephens, T. C. & Pattison, G. Transition-Metal-Free Homologative Cross-Coupling of Aldehydes and Ketones with Geminal Bis(boron) Compounds. *Org. Lett.* **19**, 3498-3501, (2017).

21 Moorthy, J. N., Samanta, S., Koner, A. L., Saha, S. & Nau, W. M. Intramolecular O-H...O hydrogen-bond-mediated reversal in the partitioning of conformationally restricted triplet 1,4-biradicals and amplification of diastereodifferentiation in their lifetimes. *J. Am. Chem. Soc.* **130**, 13608-13617, (2008).

22 Xiao, K. J., Wang, Y., Ye, K. Y. & Huang, P. Q. Versatile one-pot reductive alkylation of lactams/amides via amide activation: application to the concise syntheses of bioactive alkaloids (+/-)-bgugaine, (+/-)-coniine, (+)-preussin, and (-)-cassine. *Chem. Eur. J.* **16**, 12792-12796, (2010).
